# Supplementary figures and images for: Anatomical and Micro-CT measurement analysis of ocular volume and intraocular volume in adult Bama Miniature pigs, New Zealand rabbits, and Sprague-Dawley rats (part 1 of 2)
Source: PLoS One. 2024 Sep 20;19(9):e0310830. doi: 10.1371/journal.pone.0310830 (PMC11414937; doi:10.1371/journal.pone.0310830)

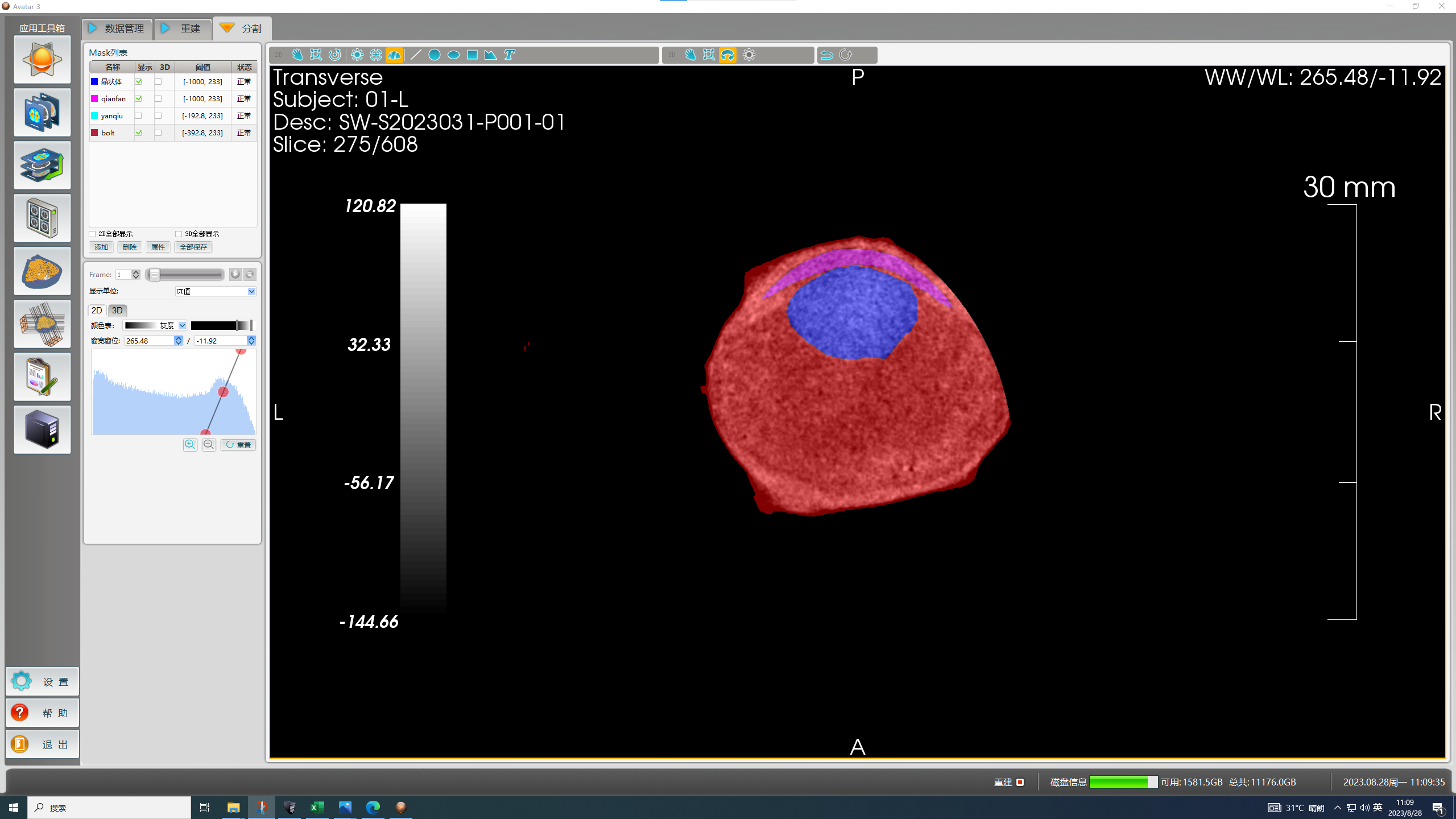

Supplement: S2 Data — (ZIP) [file pone.0310830.s002.zip › CT_pigs/01-L.png]

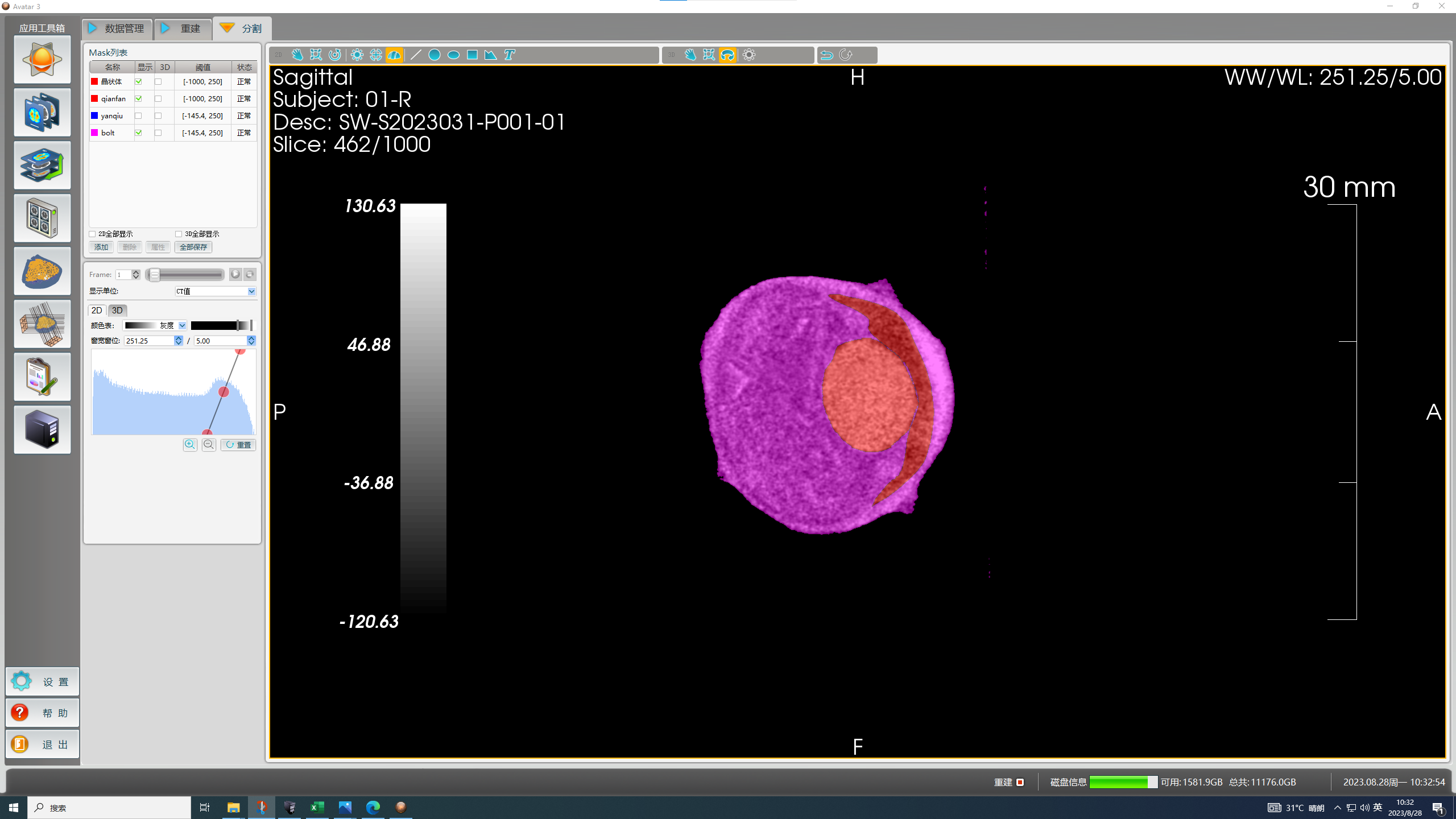

Supplement: S2 Data — (ZIP) [file pone.0310830.s002.zip › CT_pigs/01-R.png]

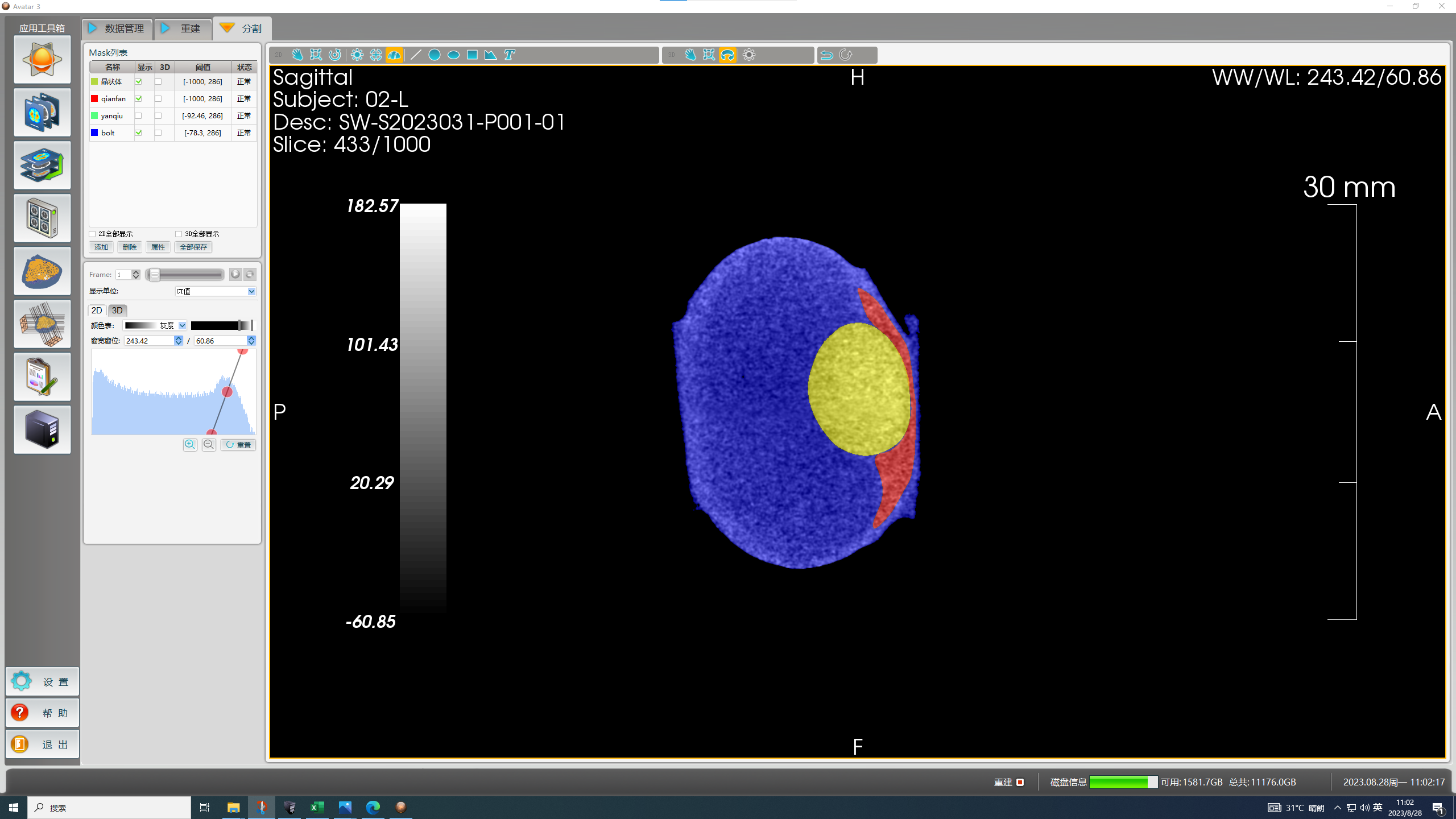

Supplement: S2 Data — (ZIP) [file pone.0310830.s002.zip › CT_pigs/02-L.png]

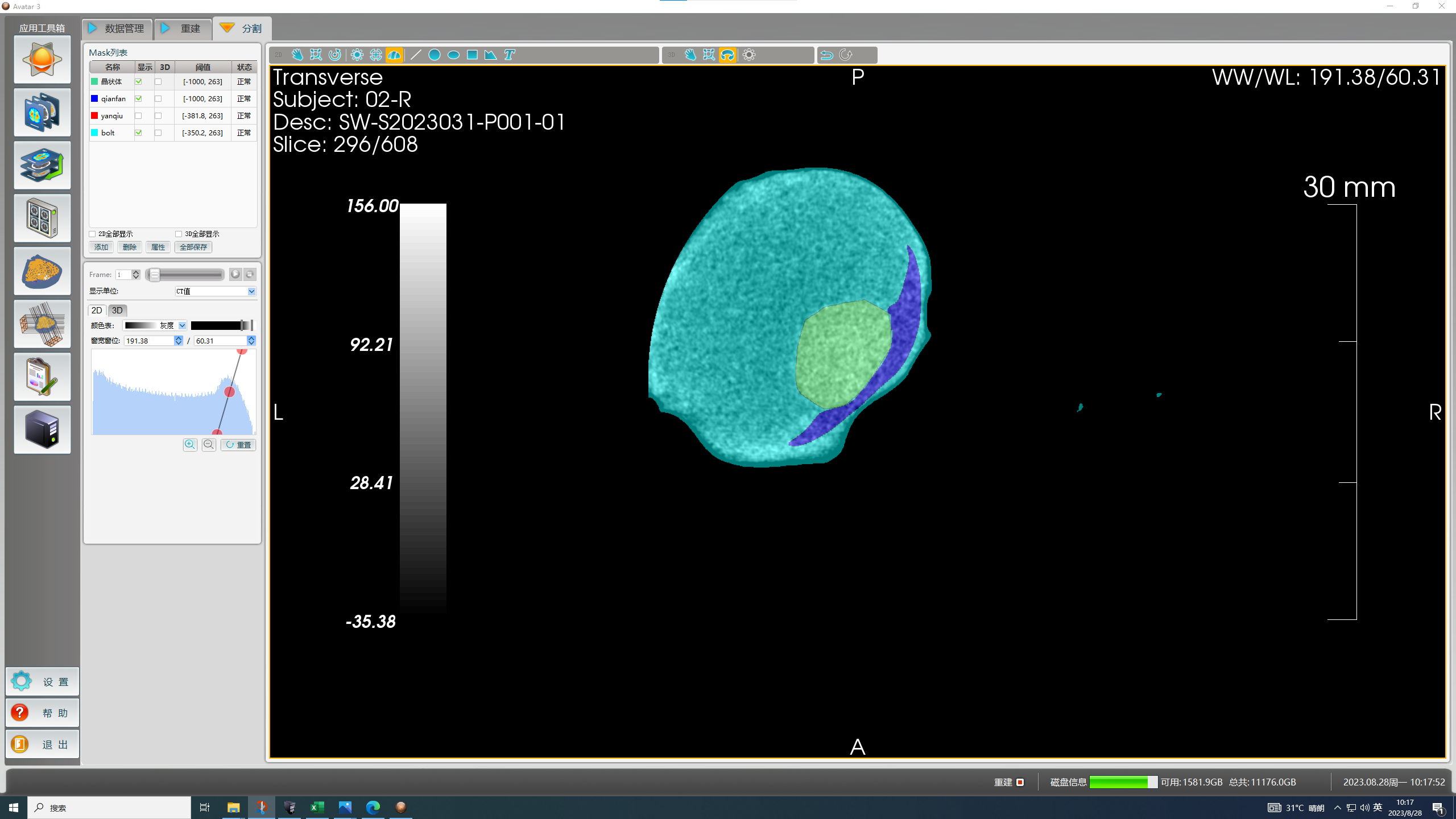

Supplement: S2 Data — (ZIP) [file pone.0310830.s002.zip › CT_pigs/02-R.png]

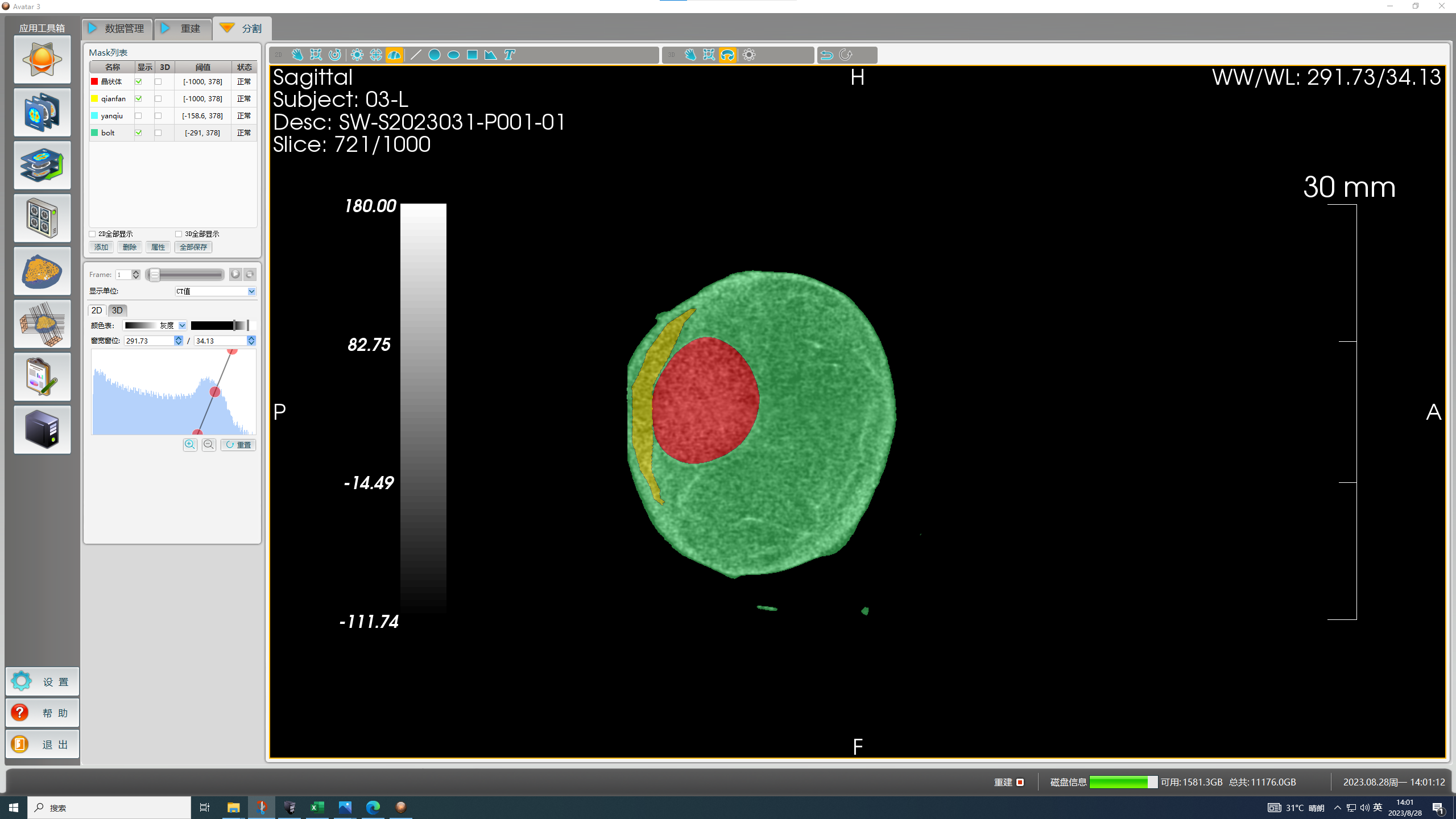

Supplement: S2 Data — (ZIP) [file pone.0310830.s002.zip › CT_pigs/03-L.png]

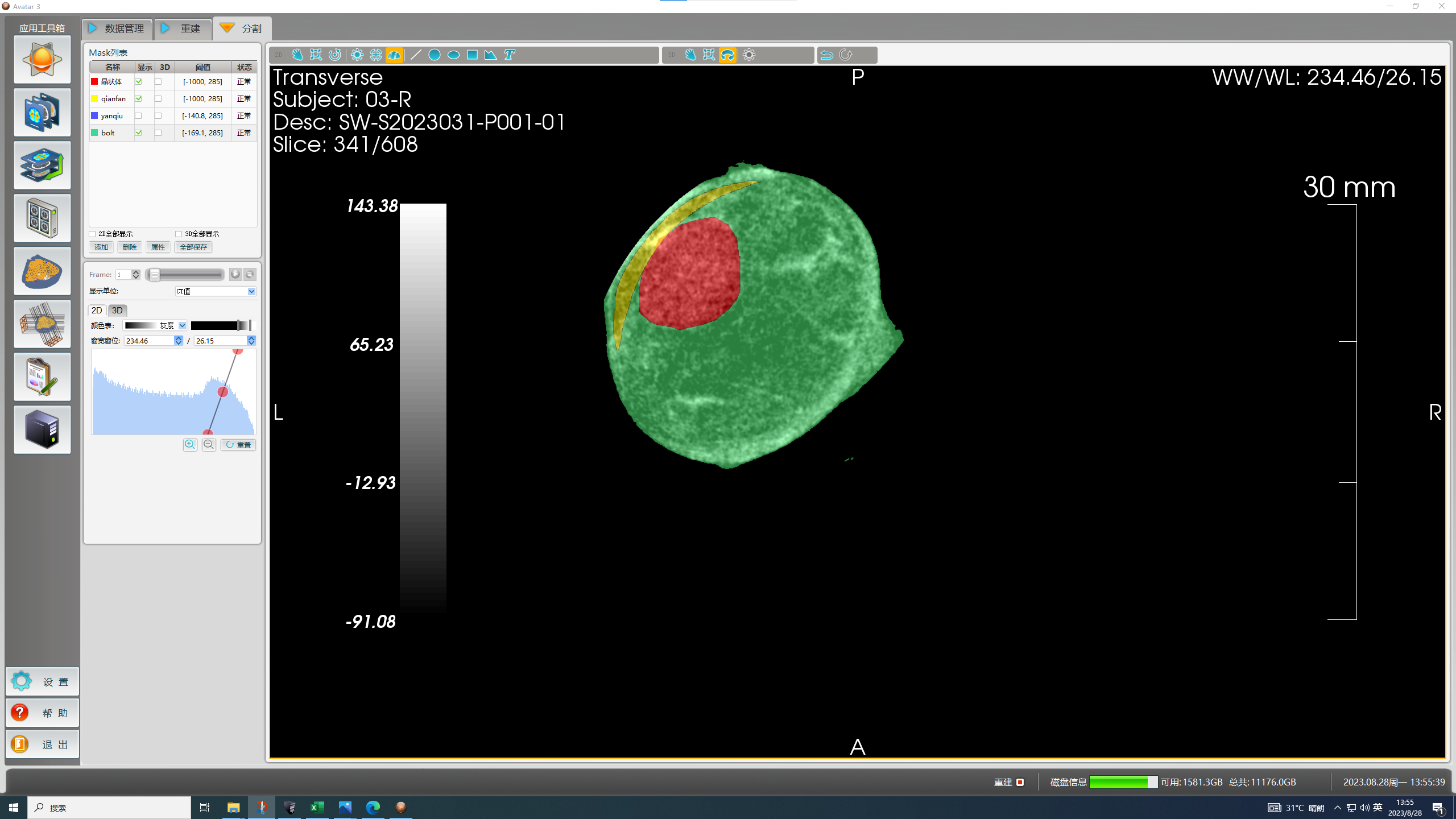

Supplement: S2 Data — (ZIP) [file pone.0310830.s002.zip › CT_pigs/03-R.png]

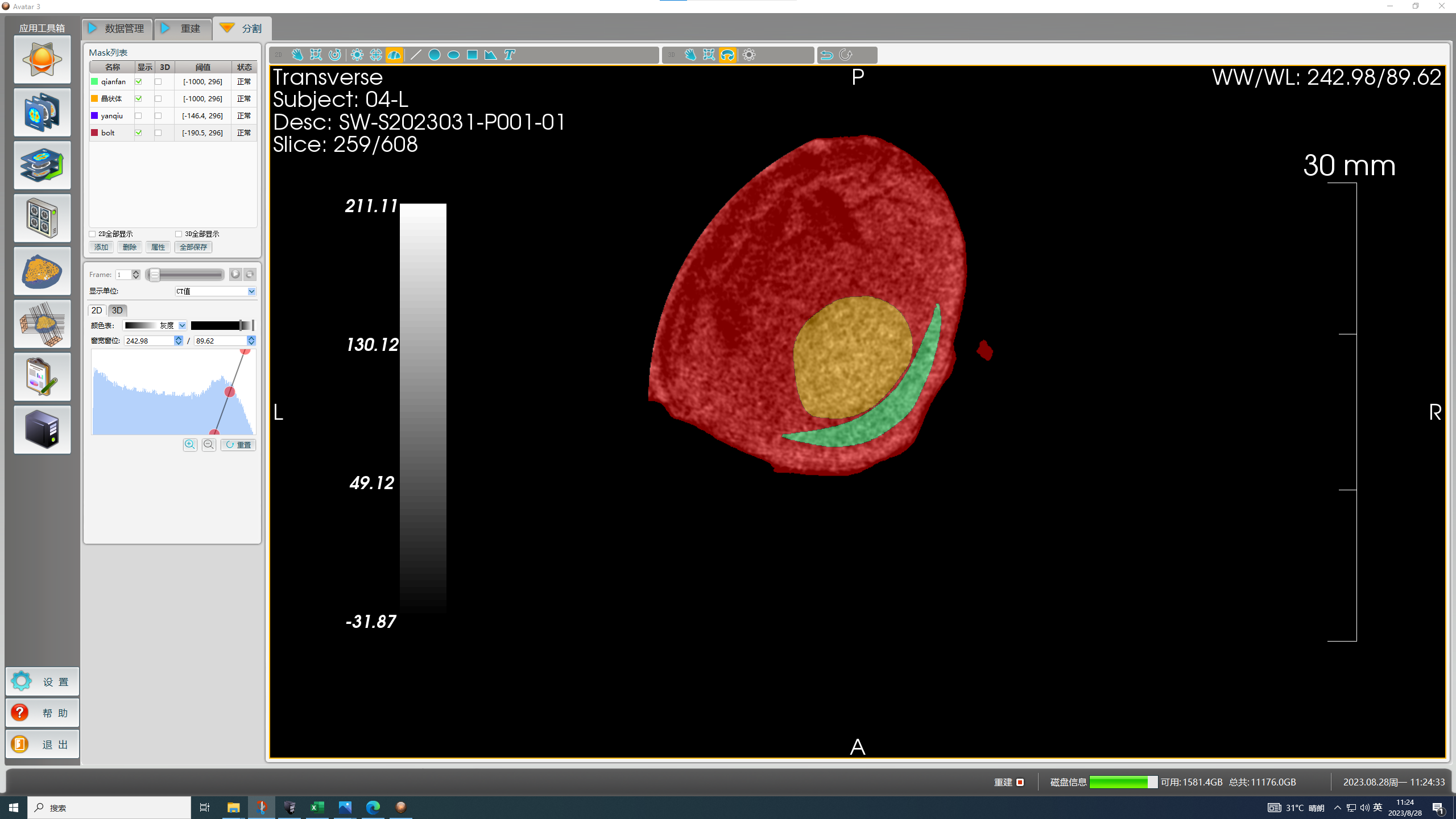

Supplement: S2 Data — (ZIP) [file pone.0310830.s002.zip › CT_pigs/04-L.png]

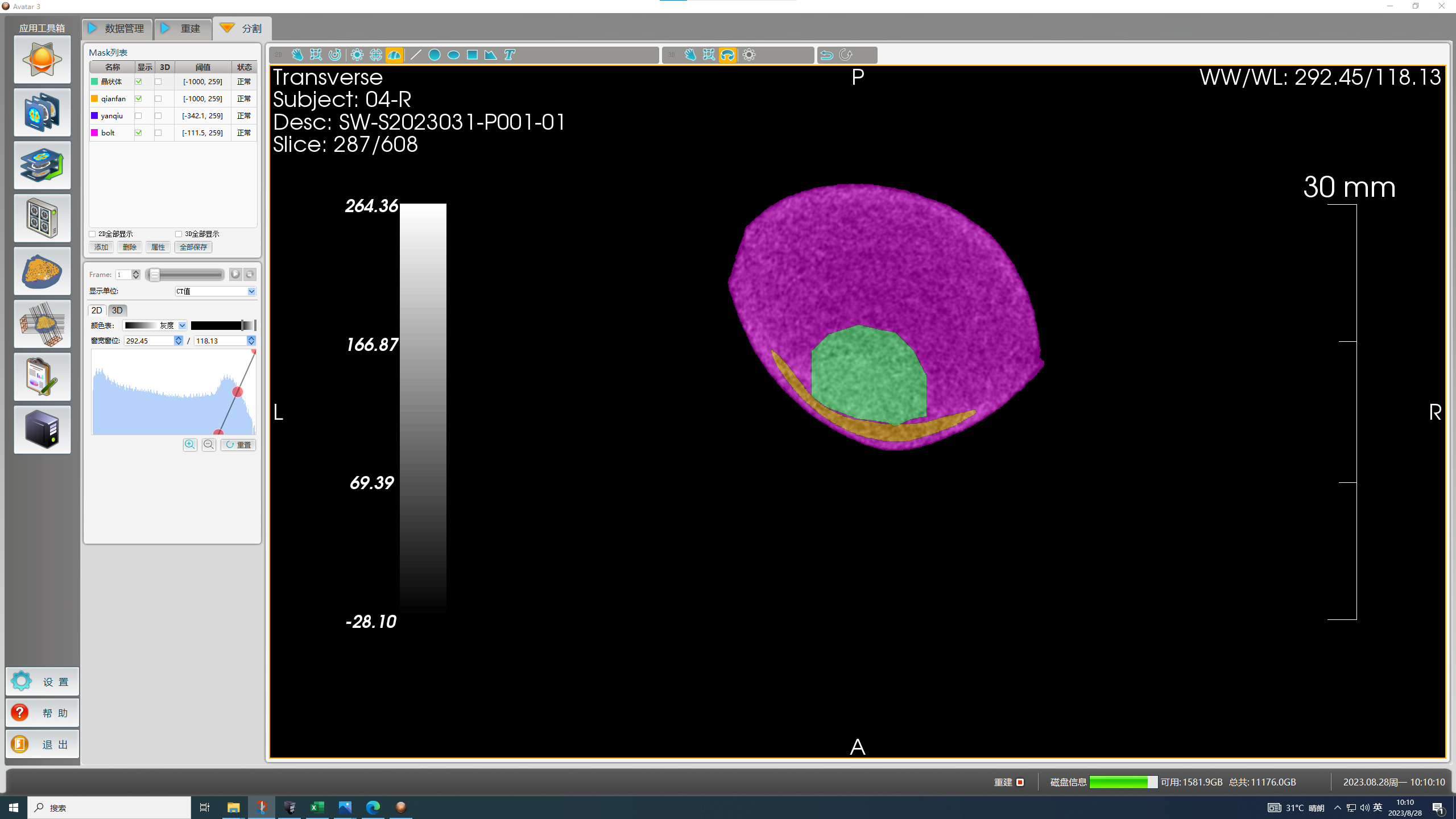

Supplement: S2 Data — (ZIP) [file pone.0310830.s002.zip › CT_pigs/04-R.png]

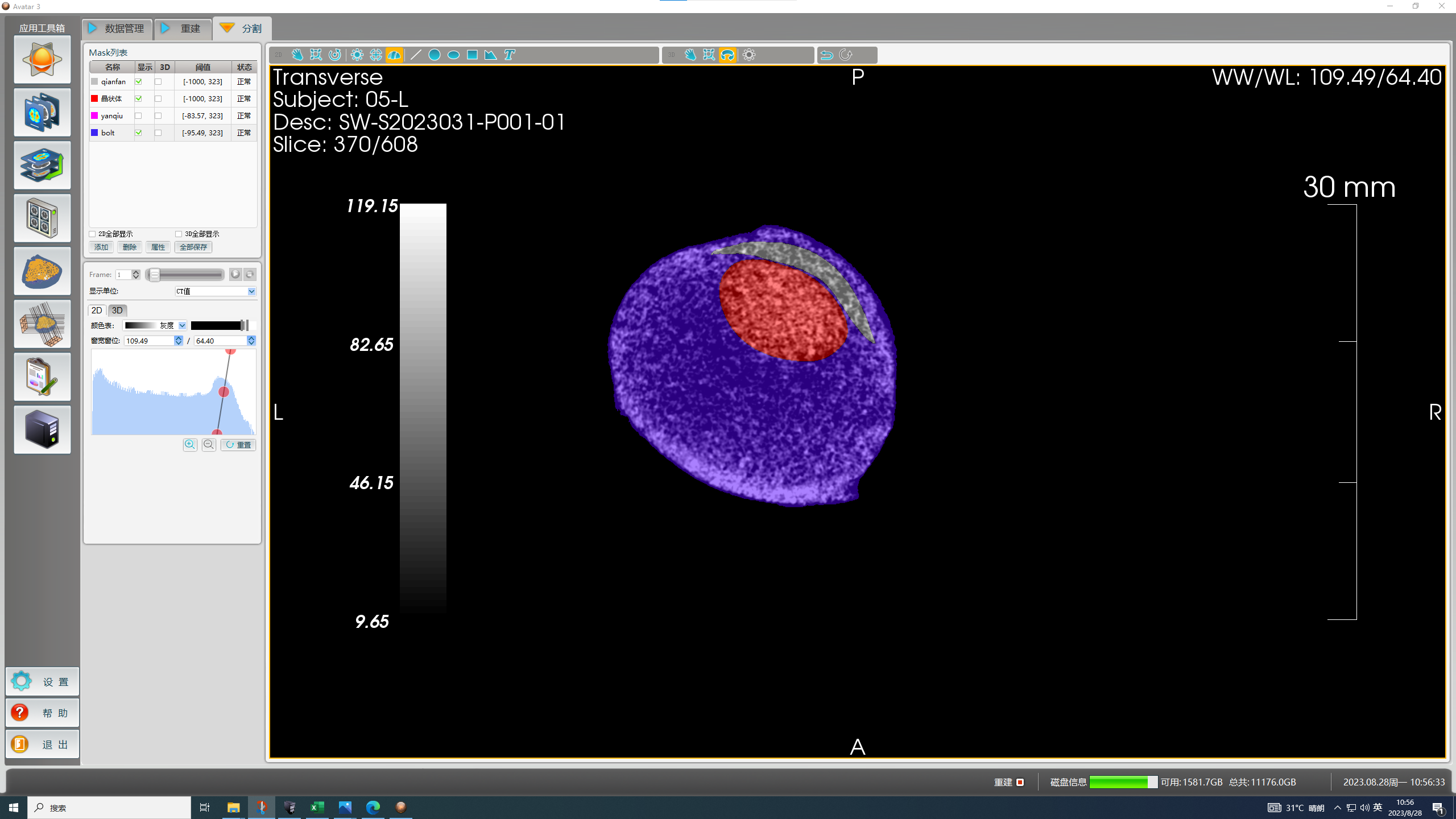

Supplement: S2 Data — (ZIP) [file pone.0310830.s002.zip › CT_pigs/05-L.png]

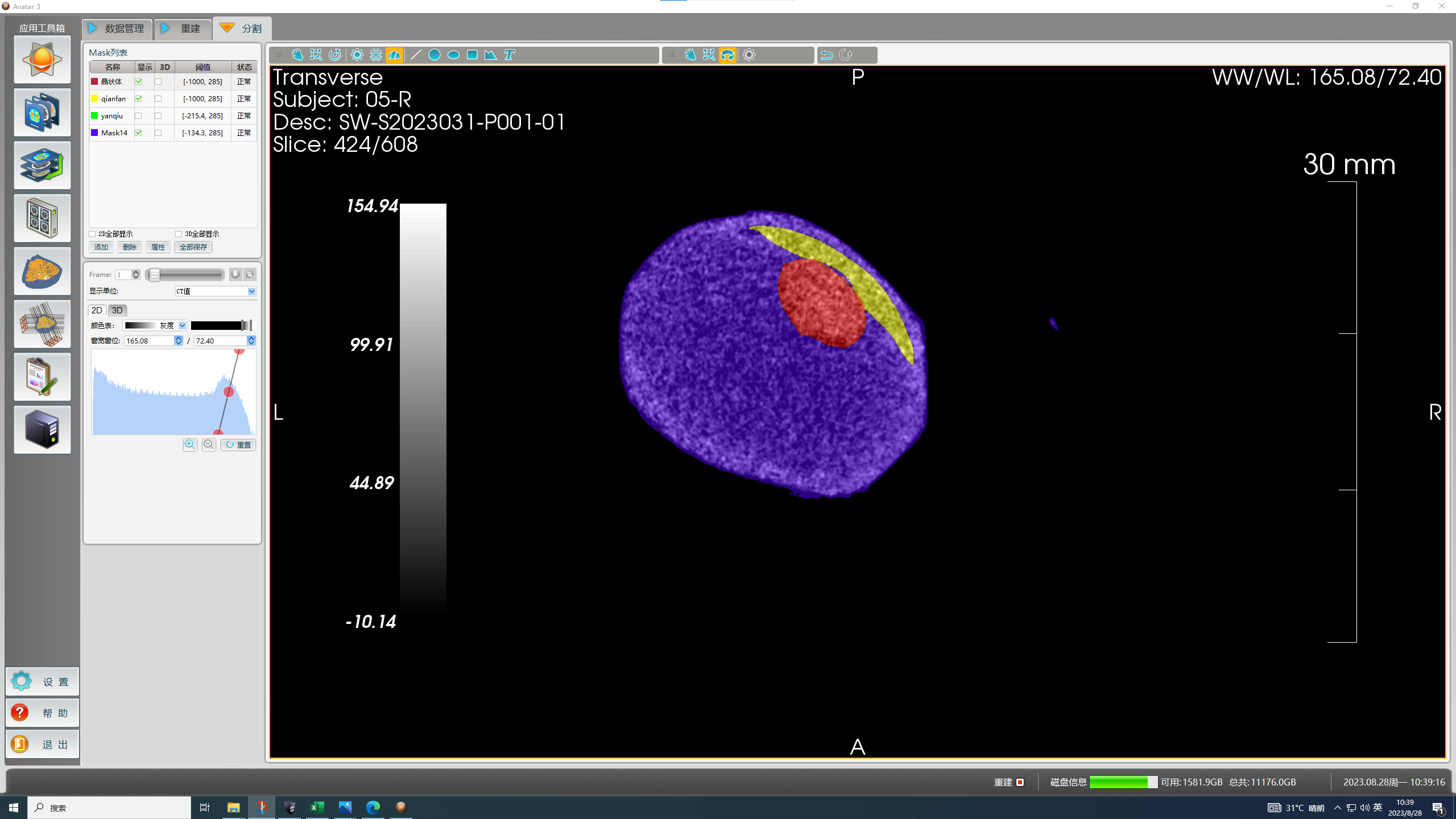

Supplement: S2 Data — (ZIP) [file pone.0310830.s002.zip › CT_pigs/05-R.png]

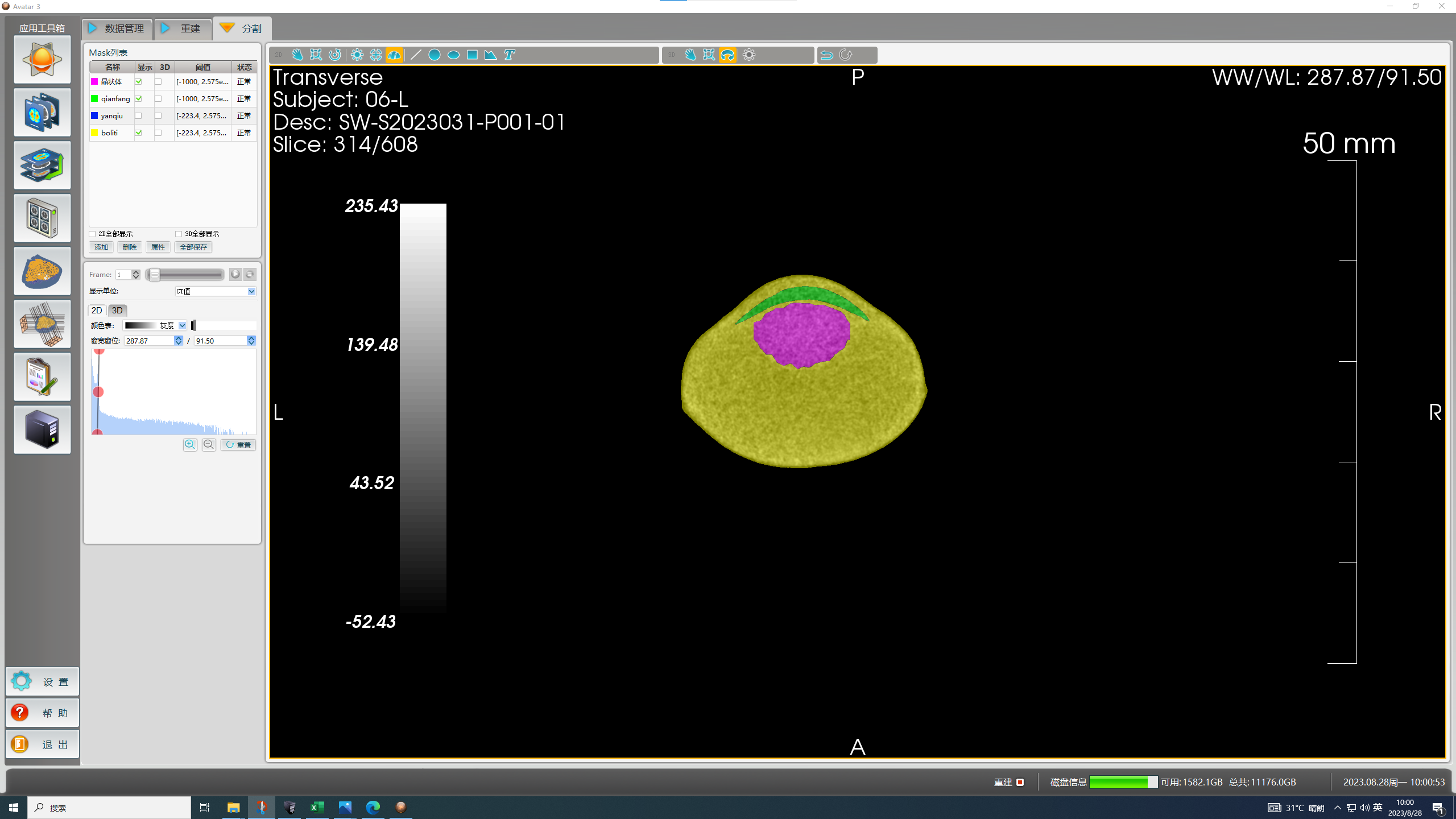

Supplement: S2 Data — (ZIP) [file pone.0310830.s002.zip › CT_pigs/06-L.png]

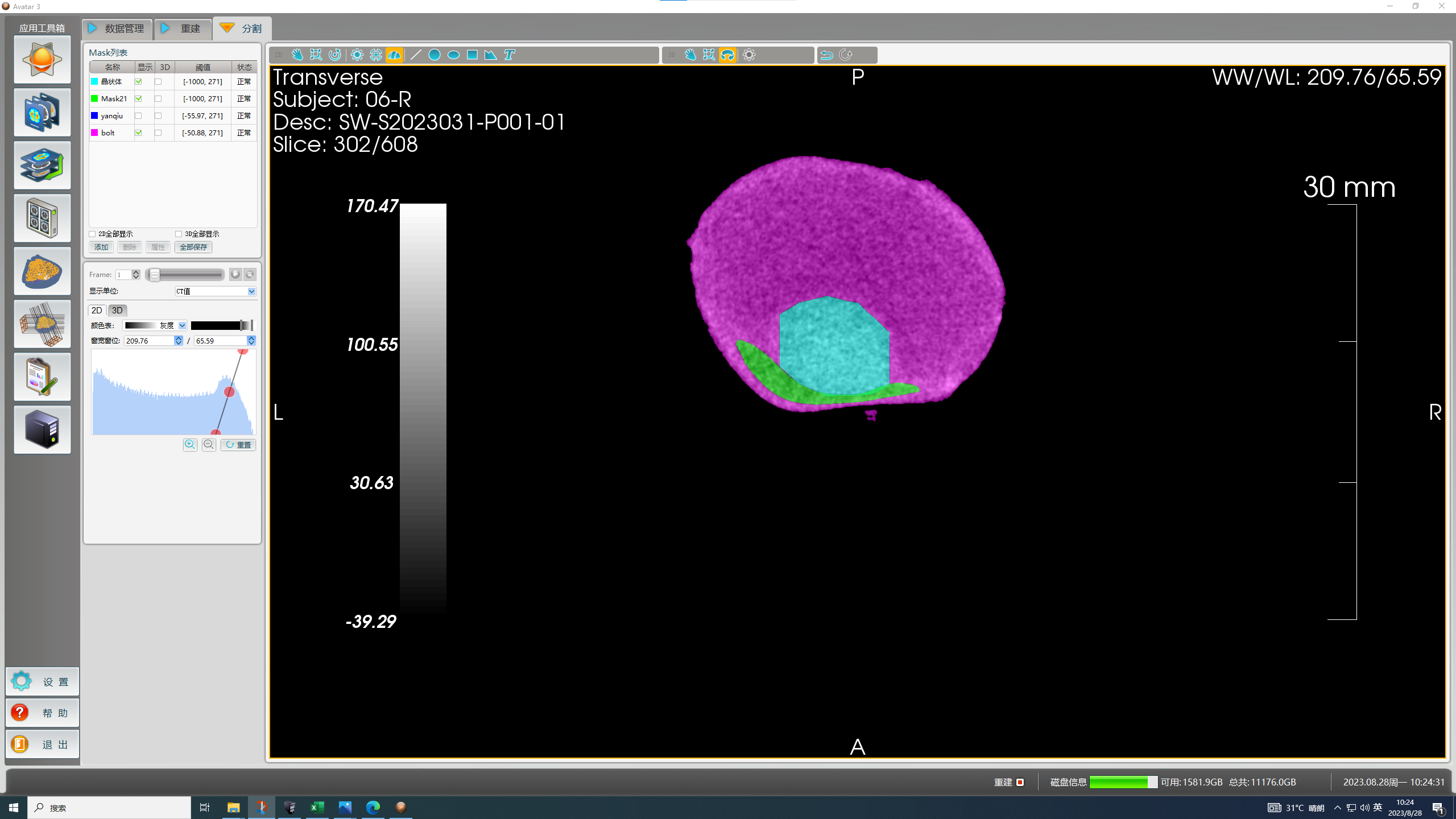

Supplement: S2 Data — (ZIP) [file pone.0310830.s002.zip › CT_pigs/06-R.png]

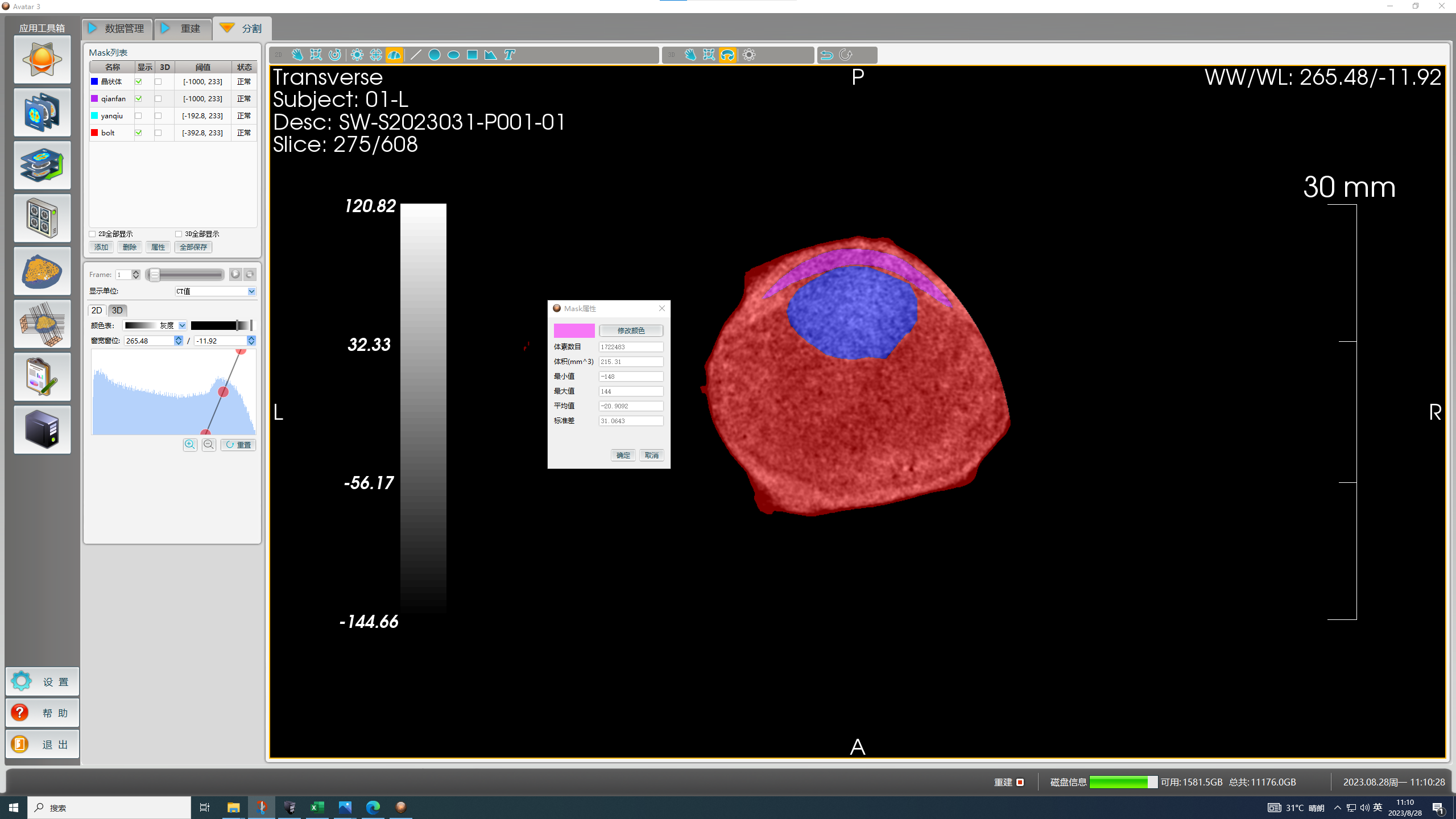

Supplement: S2 Data — (ZIP) [file pone.0310830.s002.zip › CT_pigs/Anterior chamber/01-L.png]

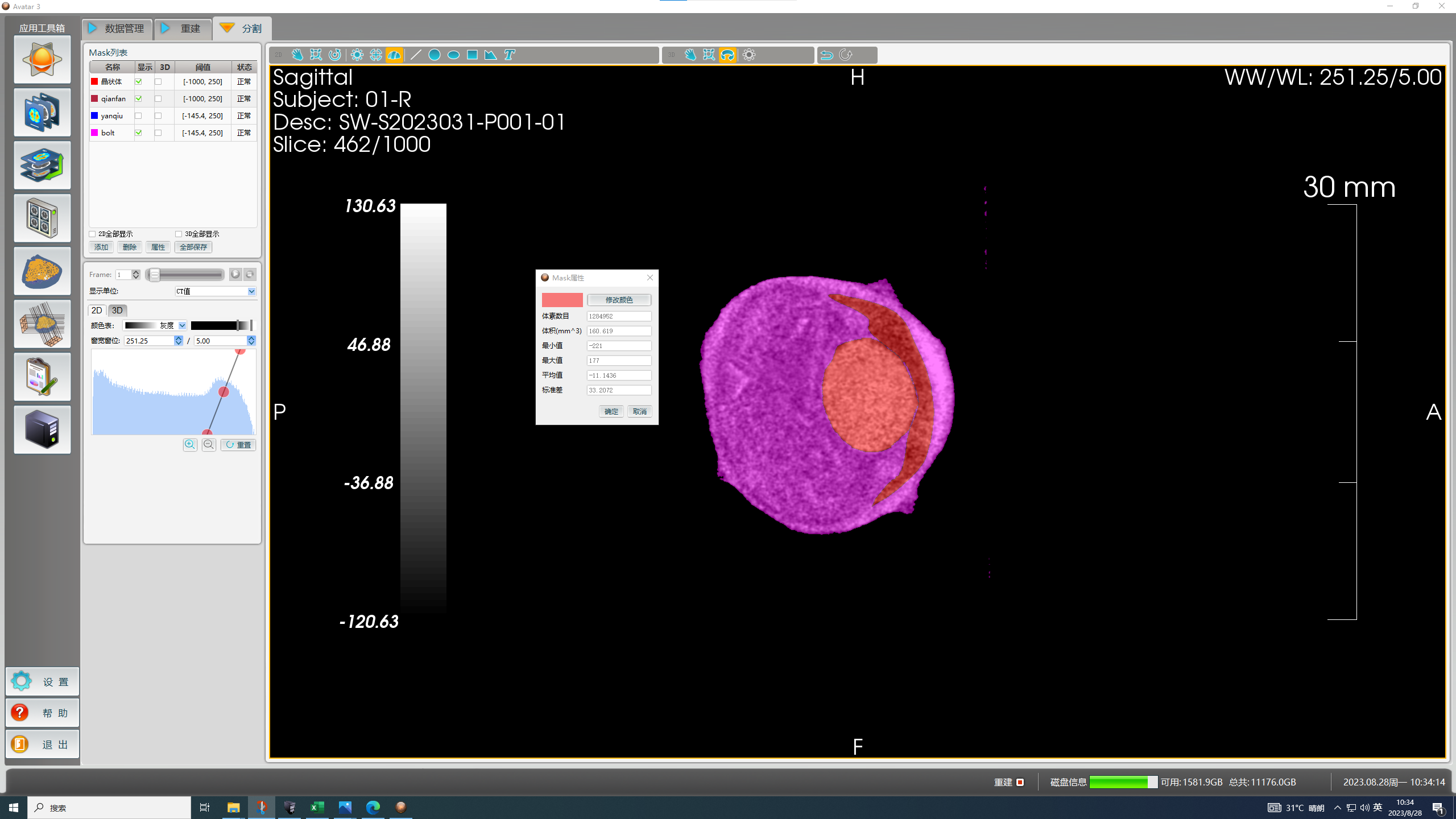

Supplement: S2 Data — (ZIP) [file pone.0310830.s002.zip › CT_pigs/Anterior chamber/01-R.png]

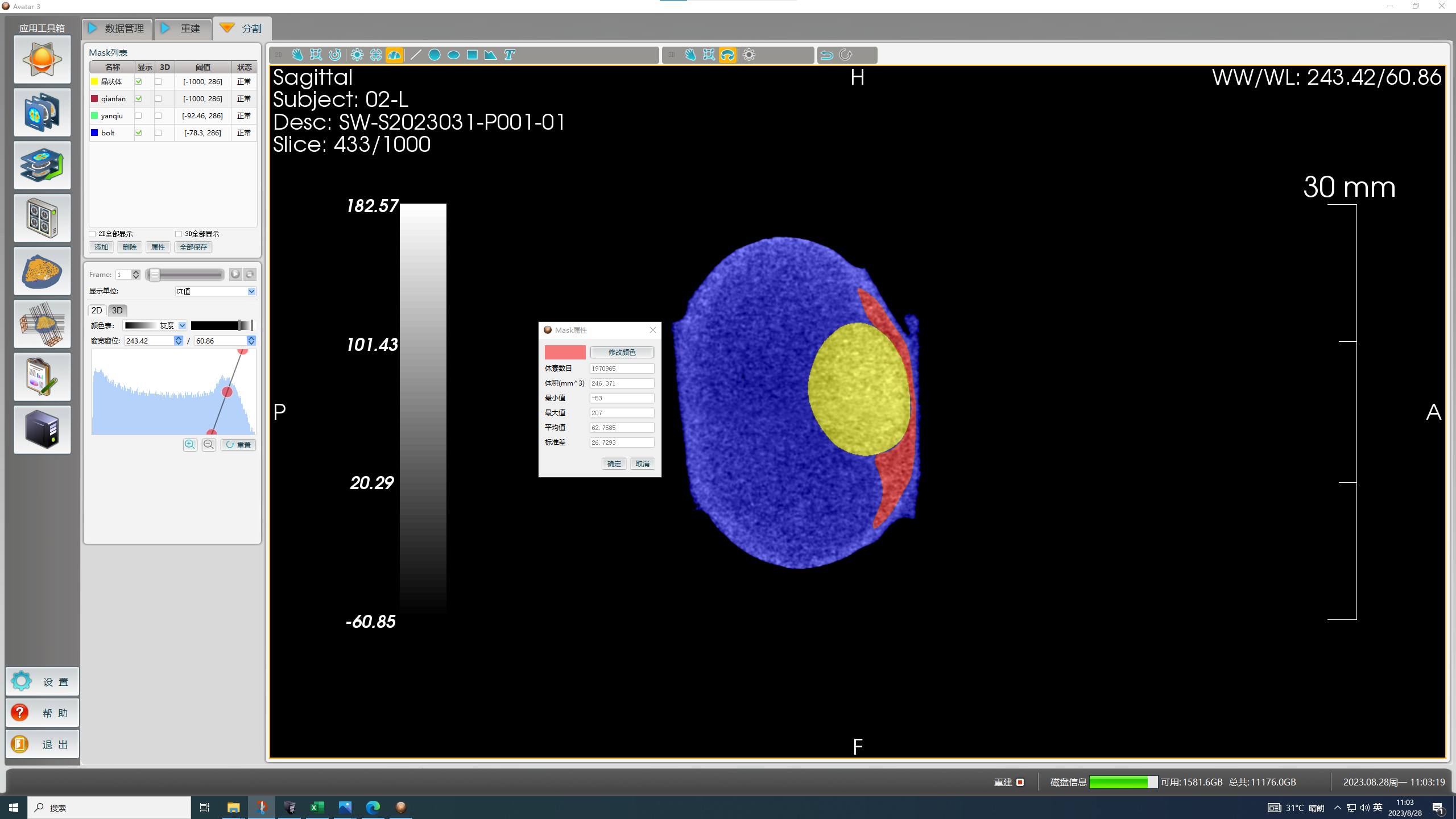

Supplement: S2 Data — (ZIP) [file pone.0310830.s002.zip › CT_pigs/Anterior chamber/02-L.png]

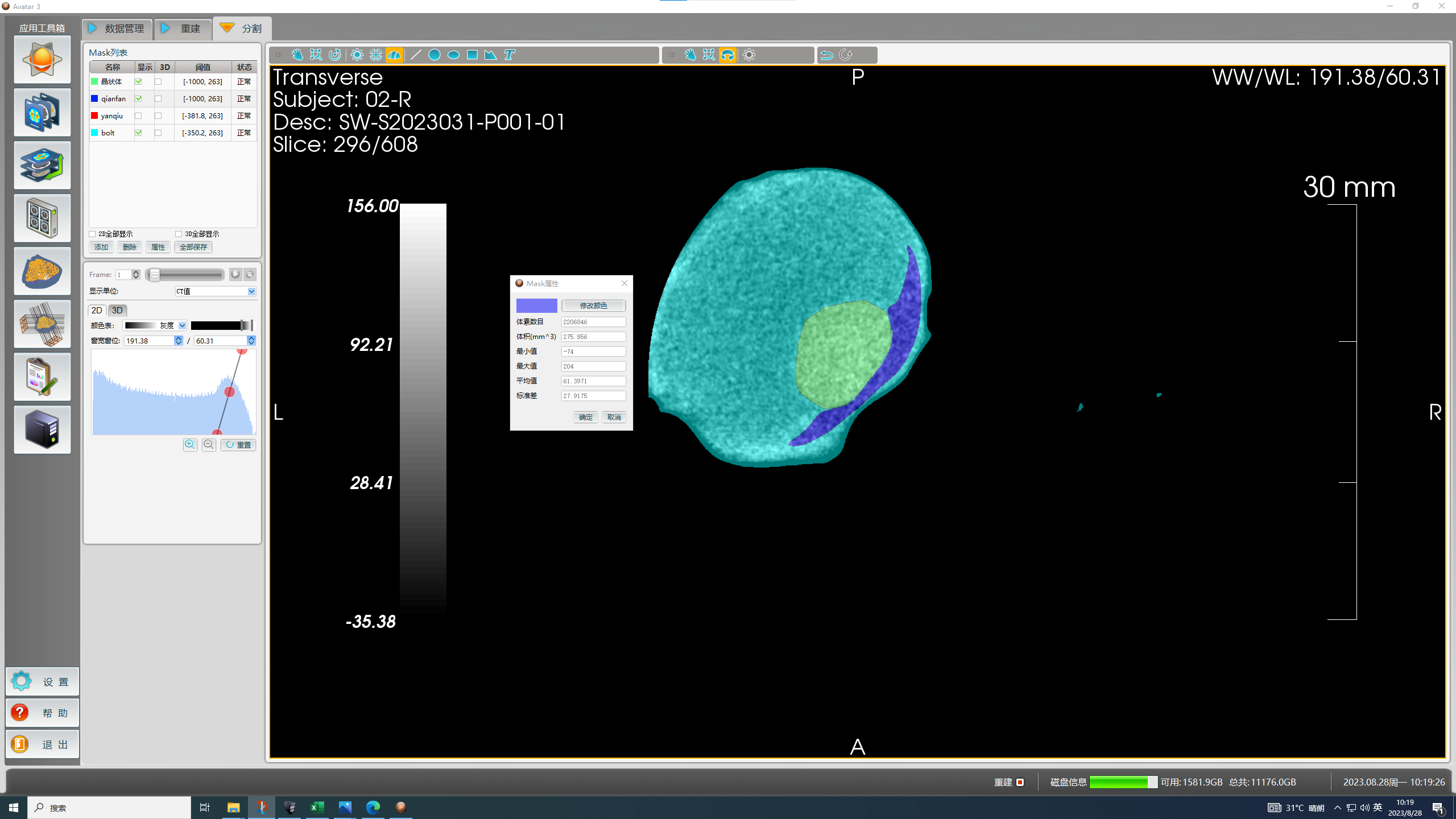

Supplement: S2 Data — (ZIP) [file pone.0310830.s002.zip › CT_pigs/Anterior chamber/02-R.png]

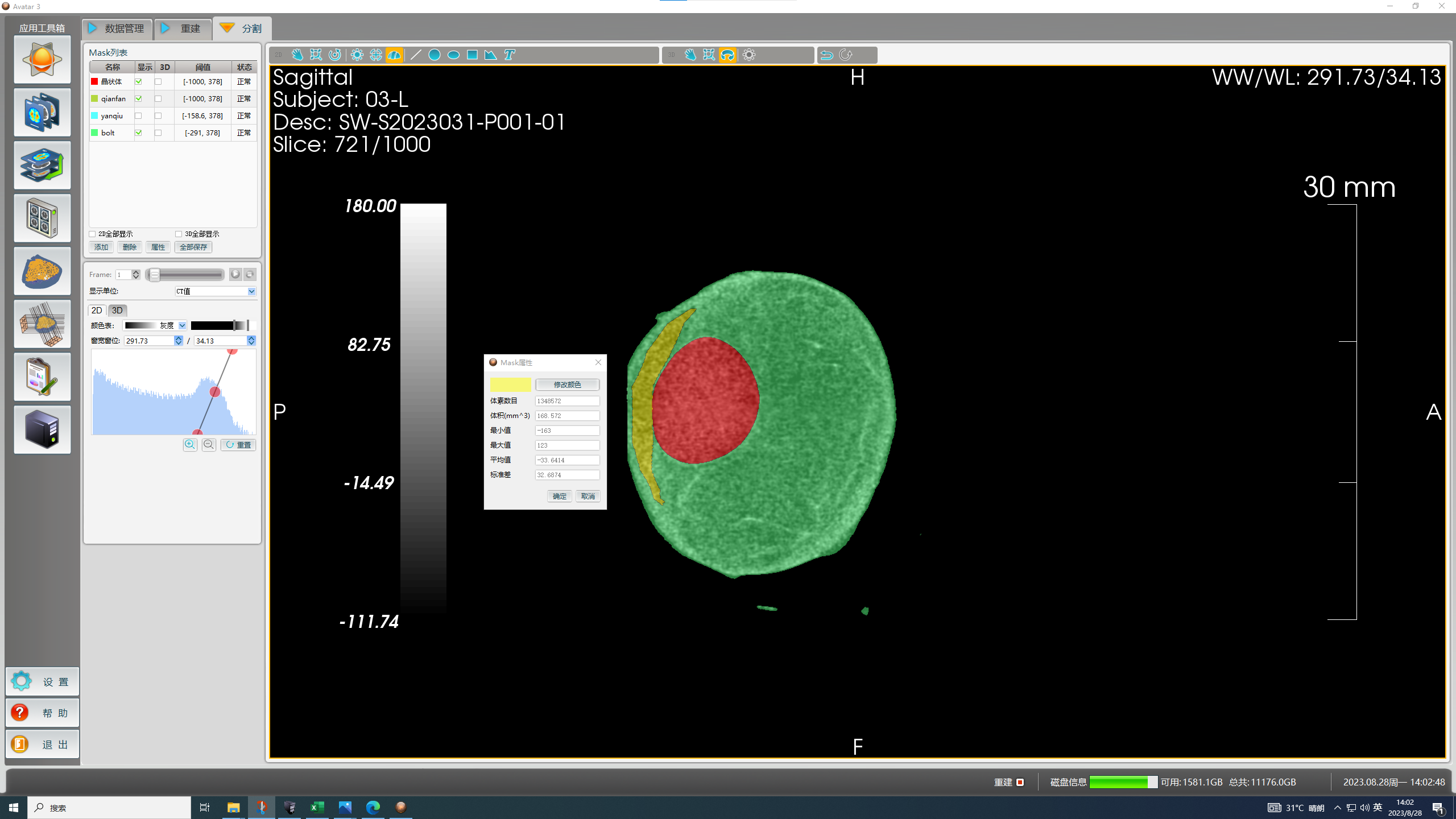

Supplement: S2 Data — (ZIP) [file pone.0310830.s002.zip › CT_pigs/Anterior chamber/03-L.png]

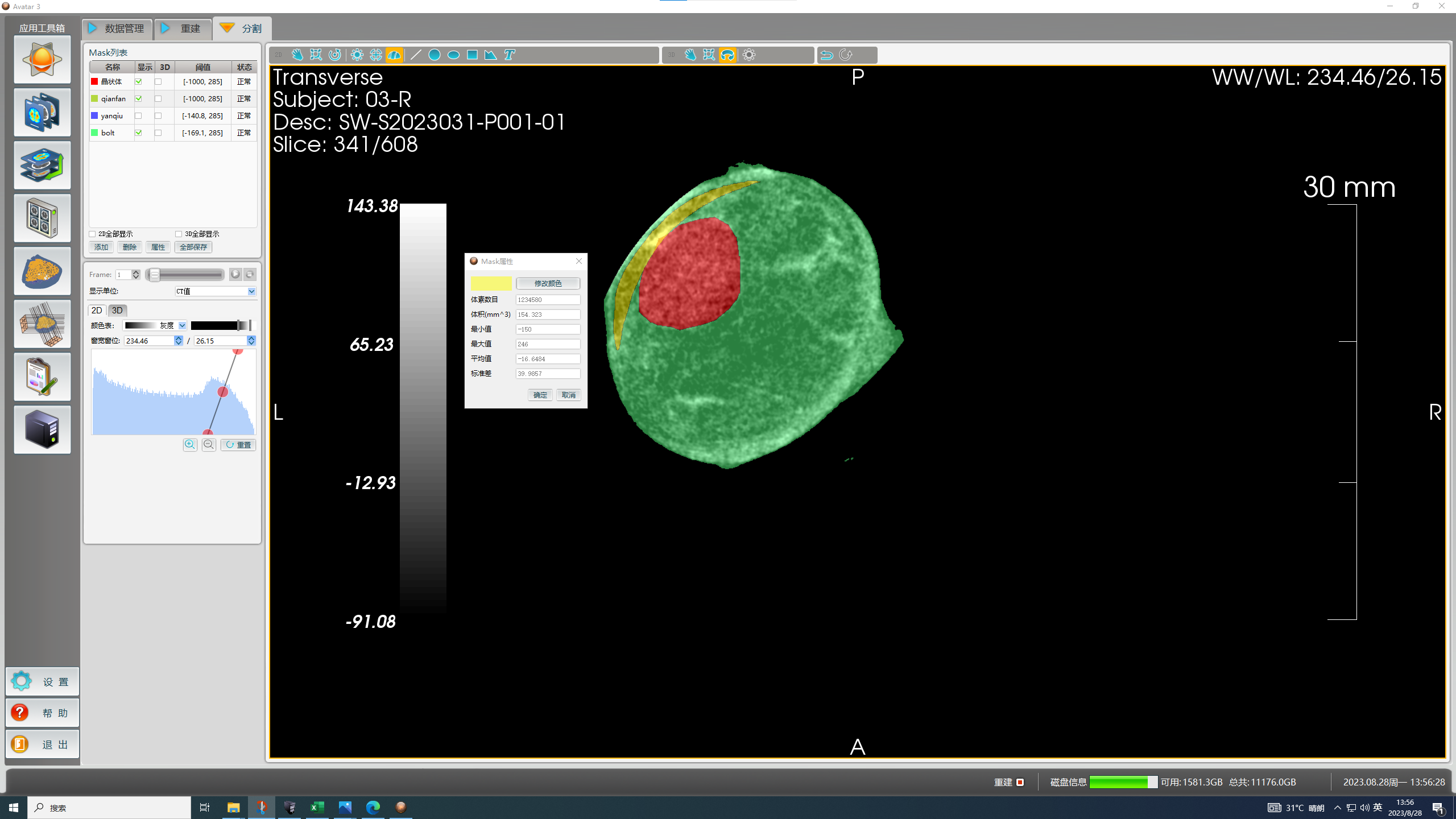

Supplement: S2 Data — (ZIP) [file pone.0310830.s002.zip › CT_pigs/Anterior chamber/03-R.png]

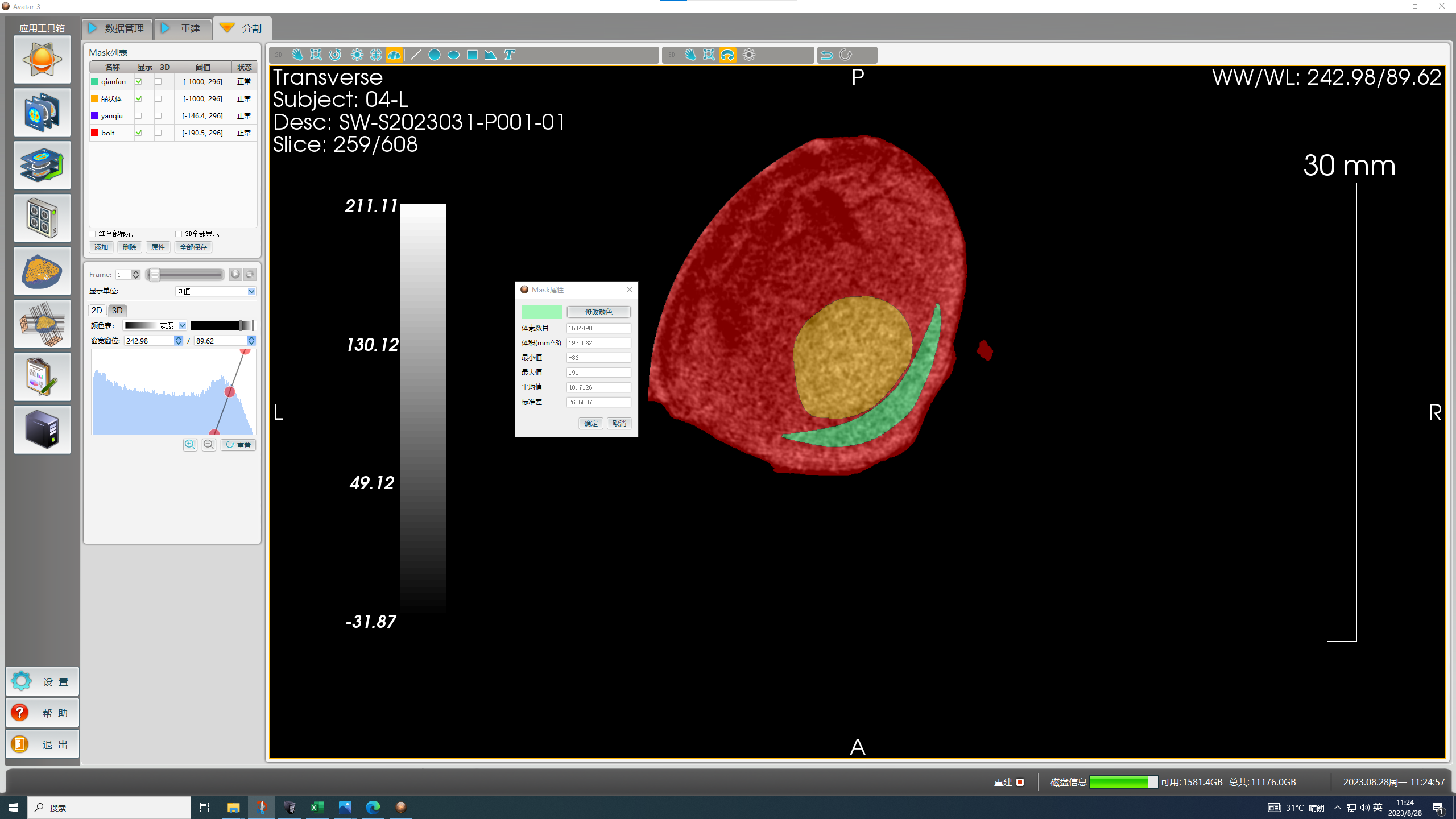

Supplement: S2 Data — (ZIP) [file pone.0310830.s002.zip › CT_pigs/Anterior chamber/04-L.png]

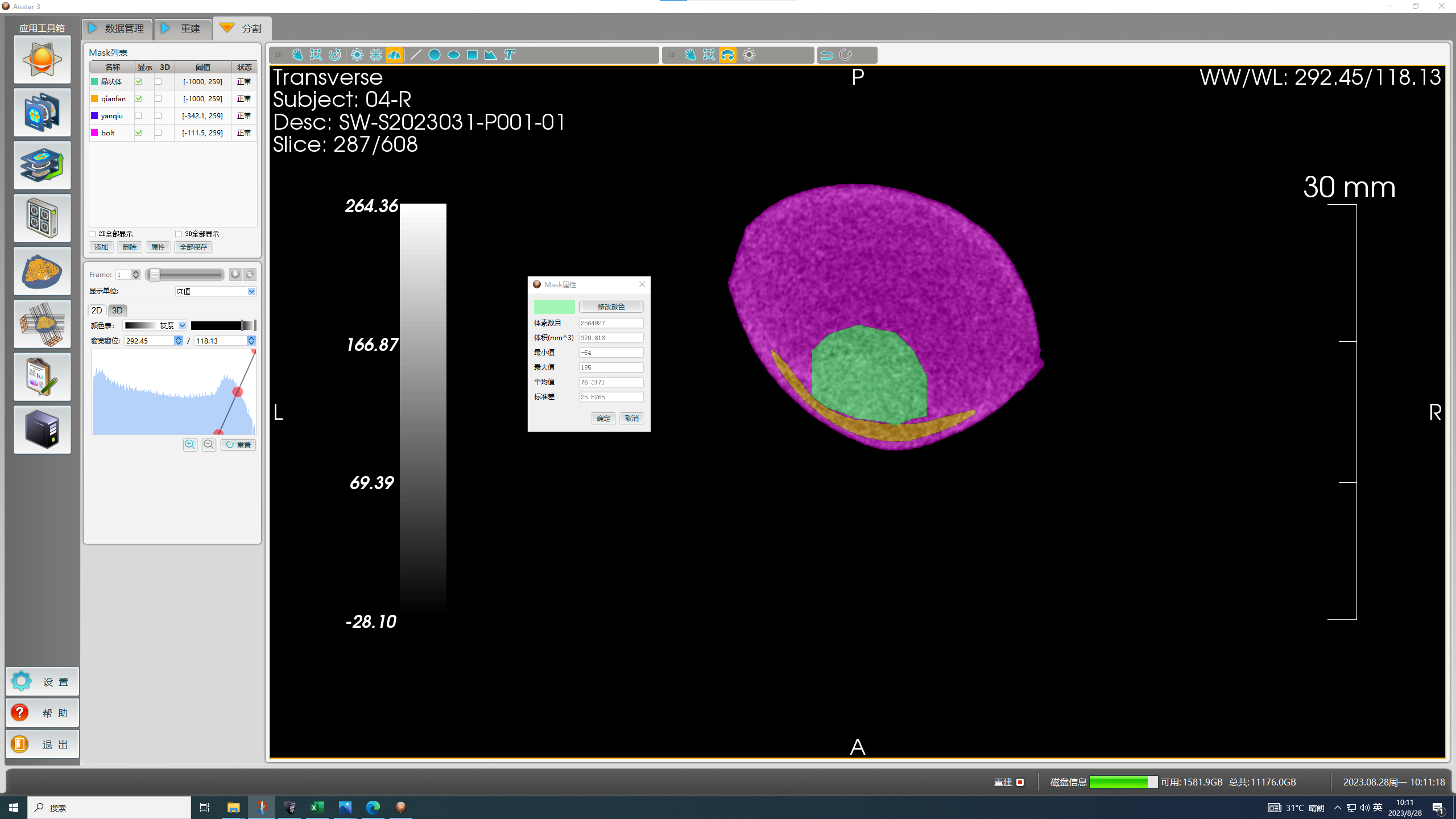

Supplement: S2 Data — (ZIP) [file pone.0310830.s002.zip › CT_pigs/Anterior chamber/04-R.png]

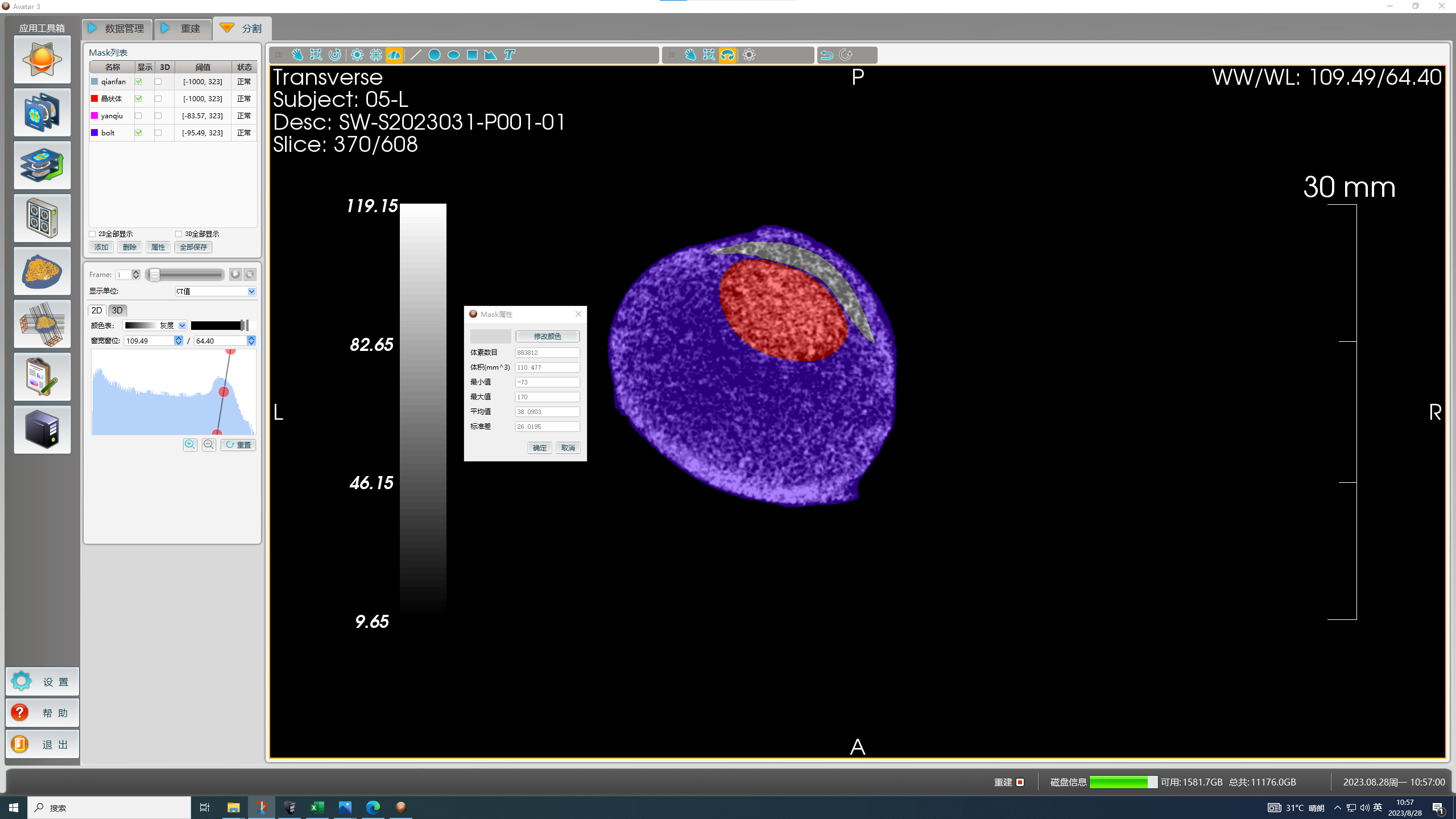

Supplement: S2 Data — (ZIP) [file pone.0310830.s002.zip › CT_pigs/Anterior chamber/05-L.png]

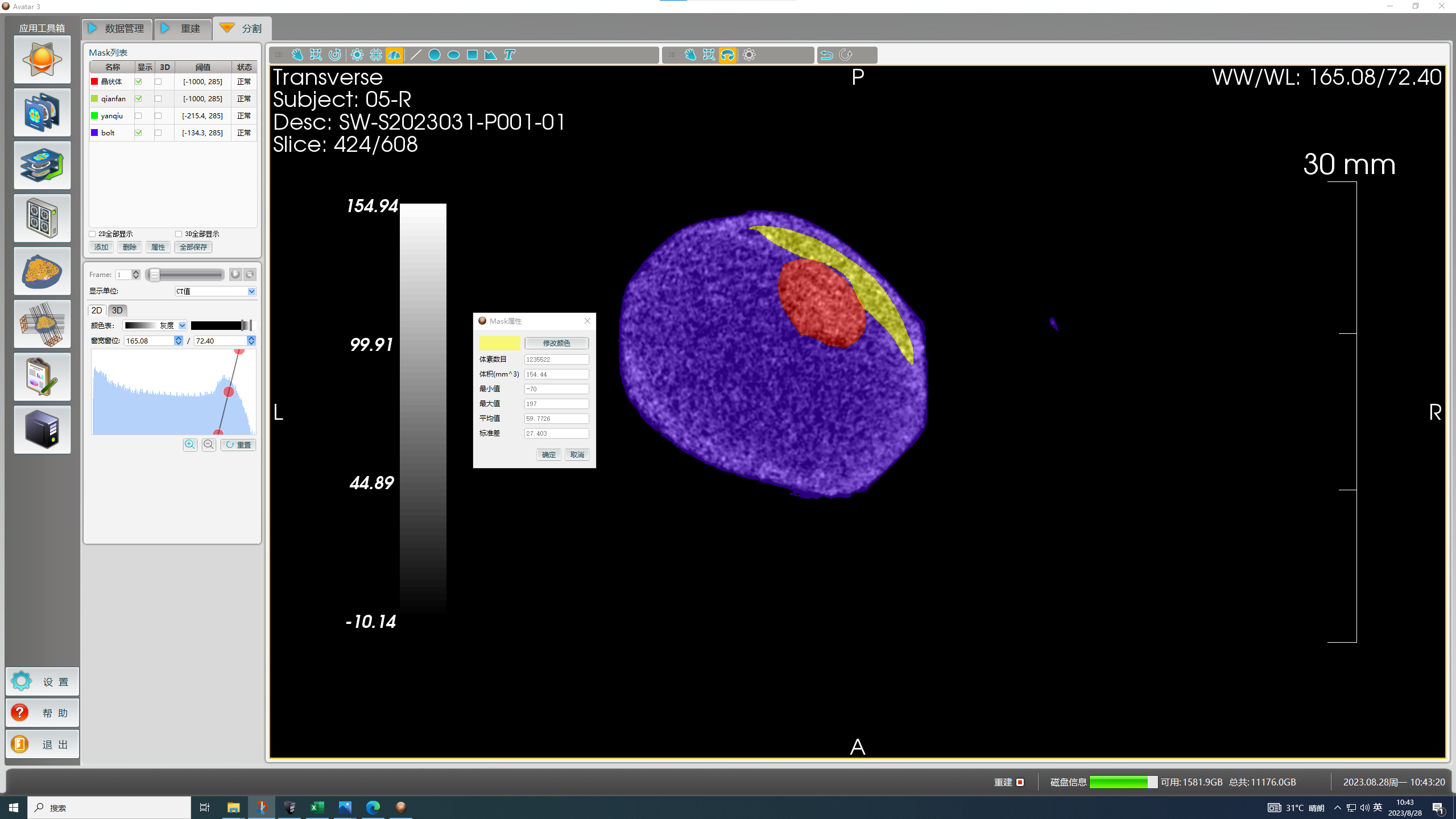

Supplement: S2 Data — (ZIP) [file pone.0310830.s002.zip › CT_pigs/Anterior chamber/05-R.png]

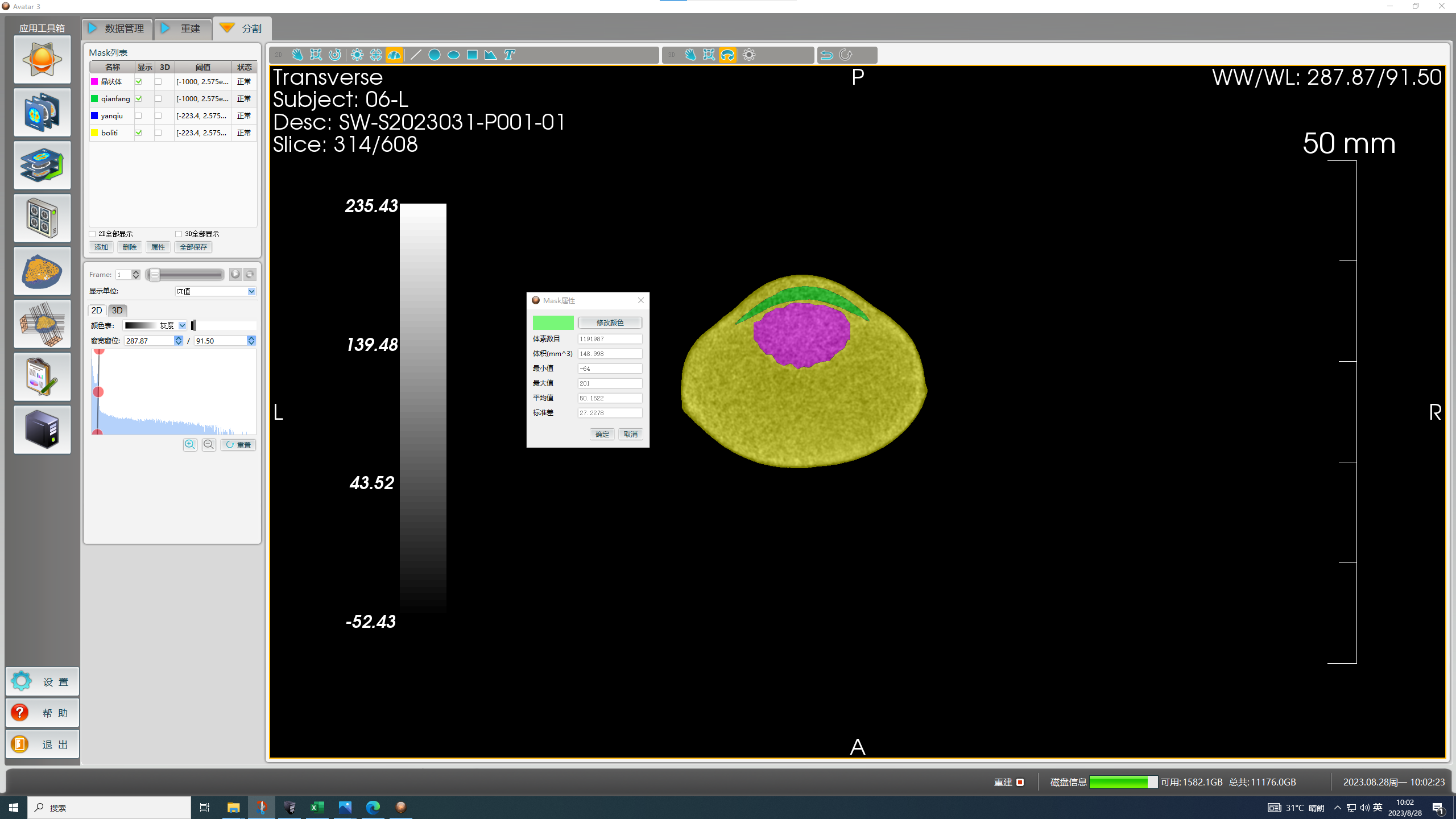

Supplement: S2 Data — (ZIP) [file pone.0310830.s002.zip › CT_pigs/Anterior chamber/06-L.png]

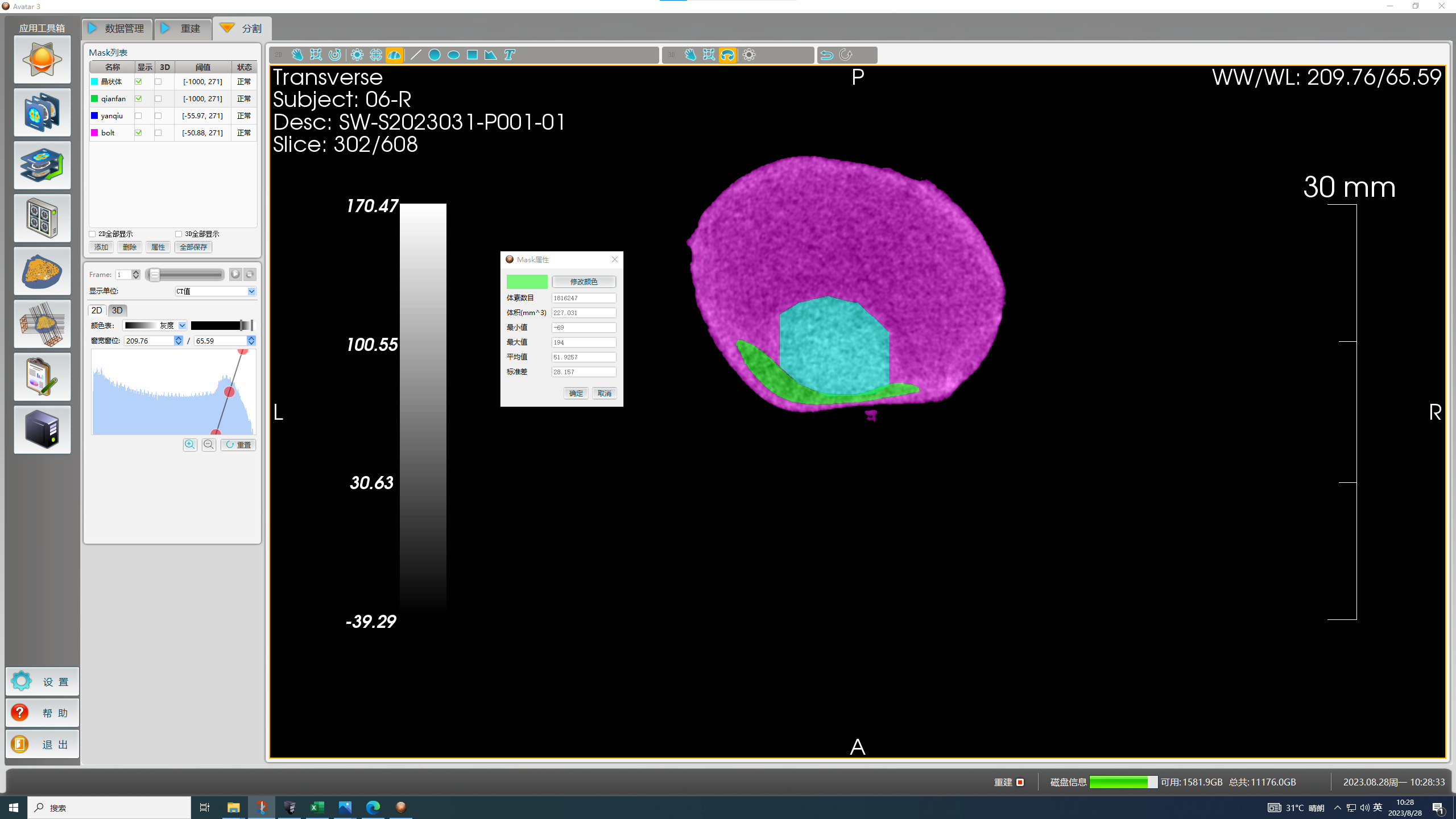

Supplement: S2 Data — (ZIP) [file pone.0310830.s002.zip › CT_pigs/Anterior chamber/06-R.png]

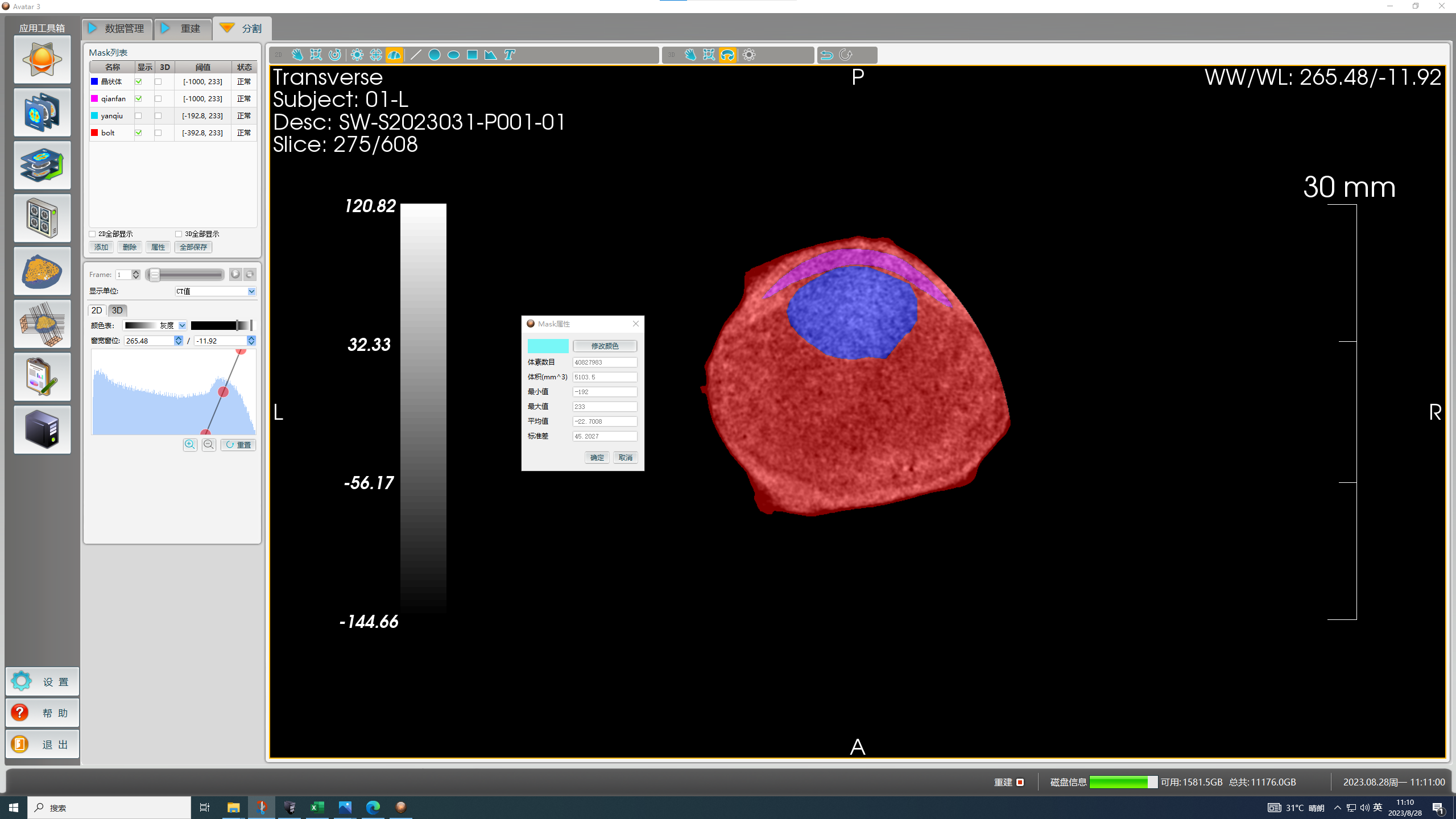

Supplement: S2 Data — (ZIP) [file pone.0310830.s002.zip › CT_pigs/Eyeball volume/01-L.png]

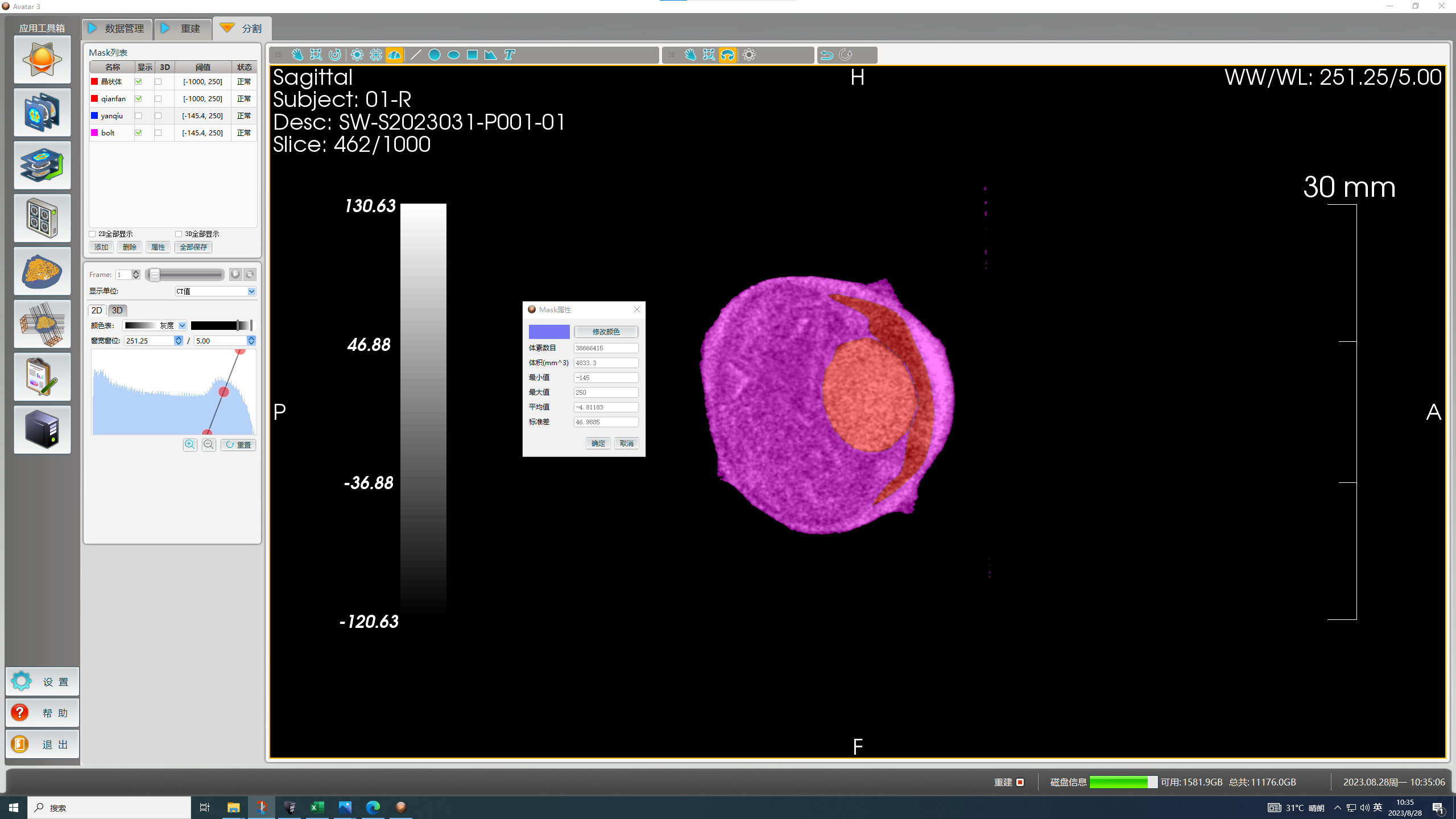

Supplement: S2 Data — (ZIP) [file pone.0310830.s002.zip › CT_pigs/Eyeball volume/01-R.png]

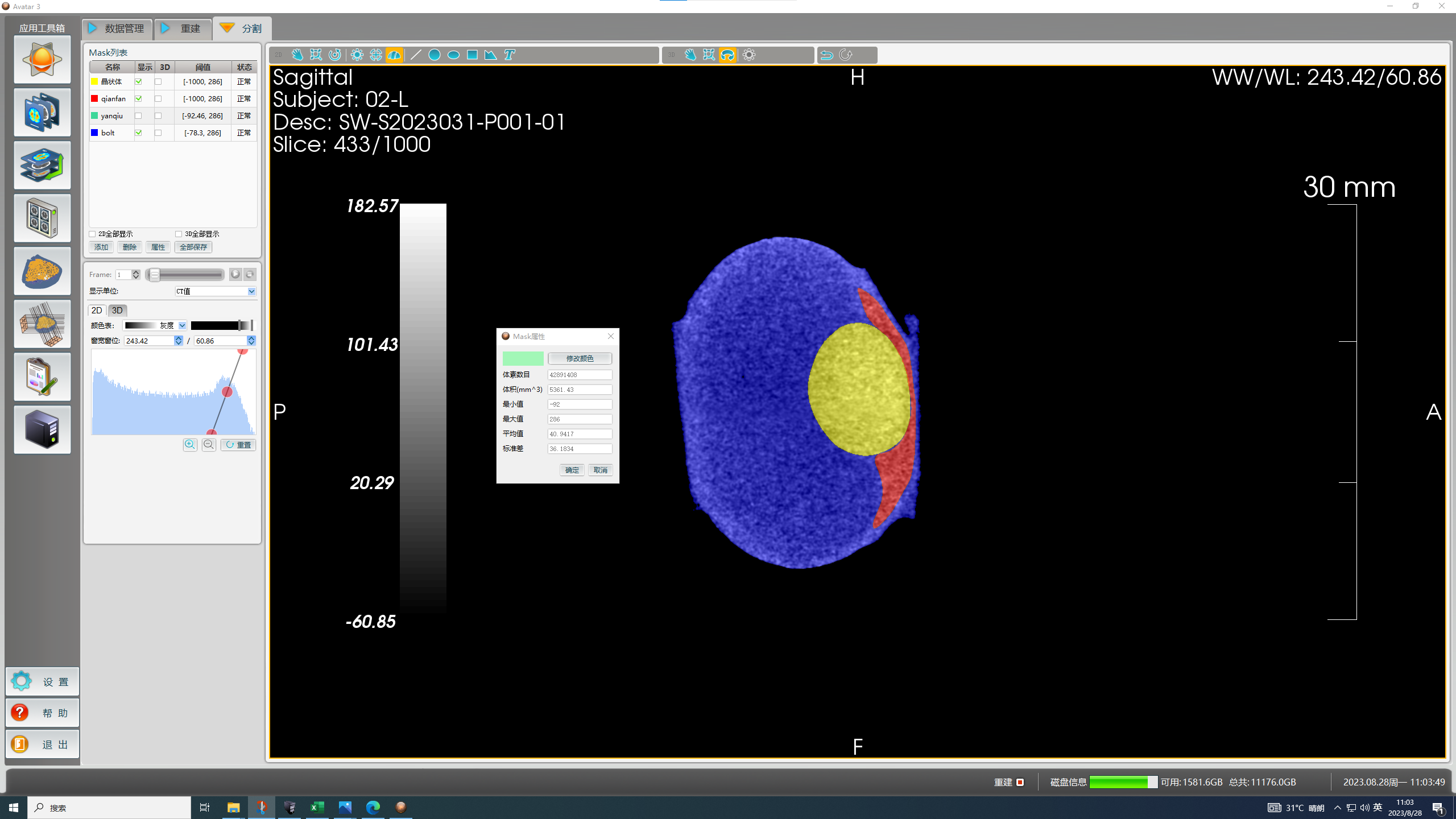

Supplement: S2 Data — (ZIP) [file pone.0310830.s002.zip › CT_pigs/Eyeball volume/02-L.png]

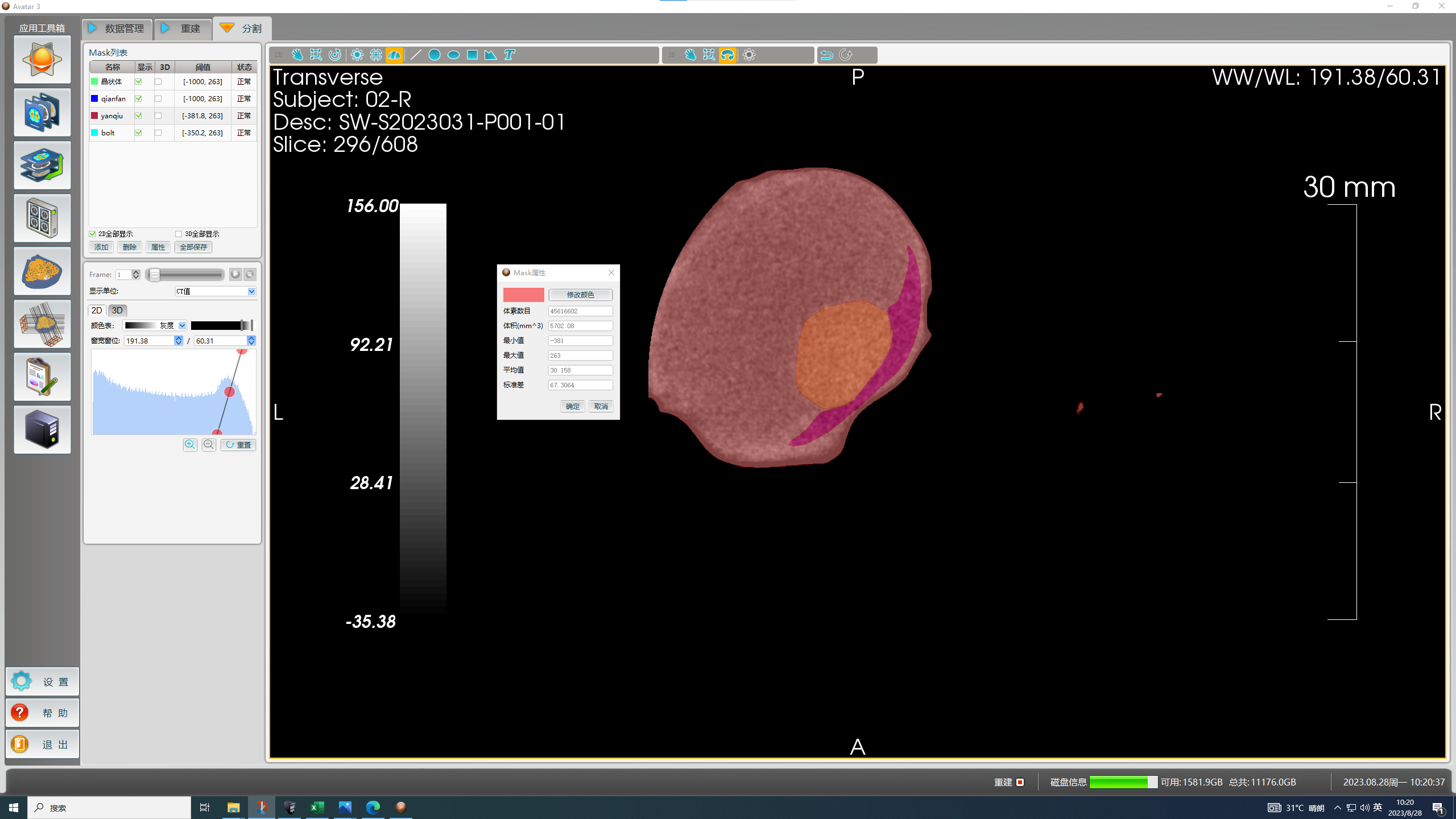

Supplement: S2 Data — (ZIP) [file pone.0310830.s002.zip › CT_pigs/Eyeball volume/02-R.png]

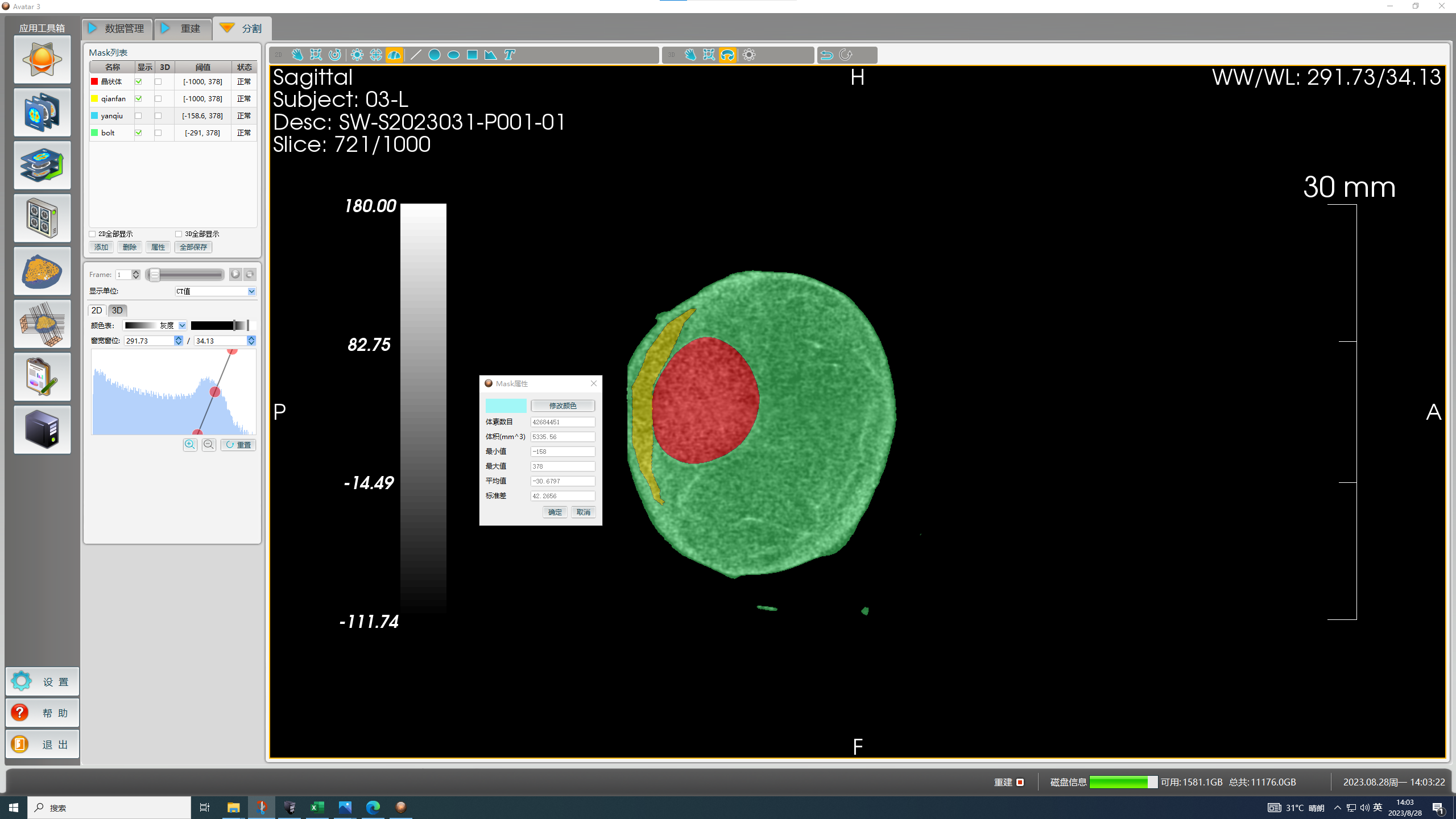

Supplement: S2 Data — (ZIP) [file pone.0310830.s002.zip › CT_pigs/Eyeball volume/03-L.png]

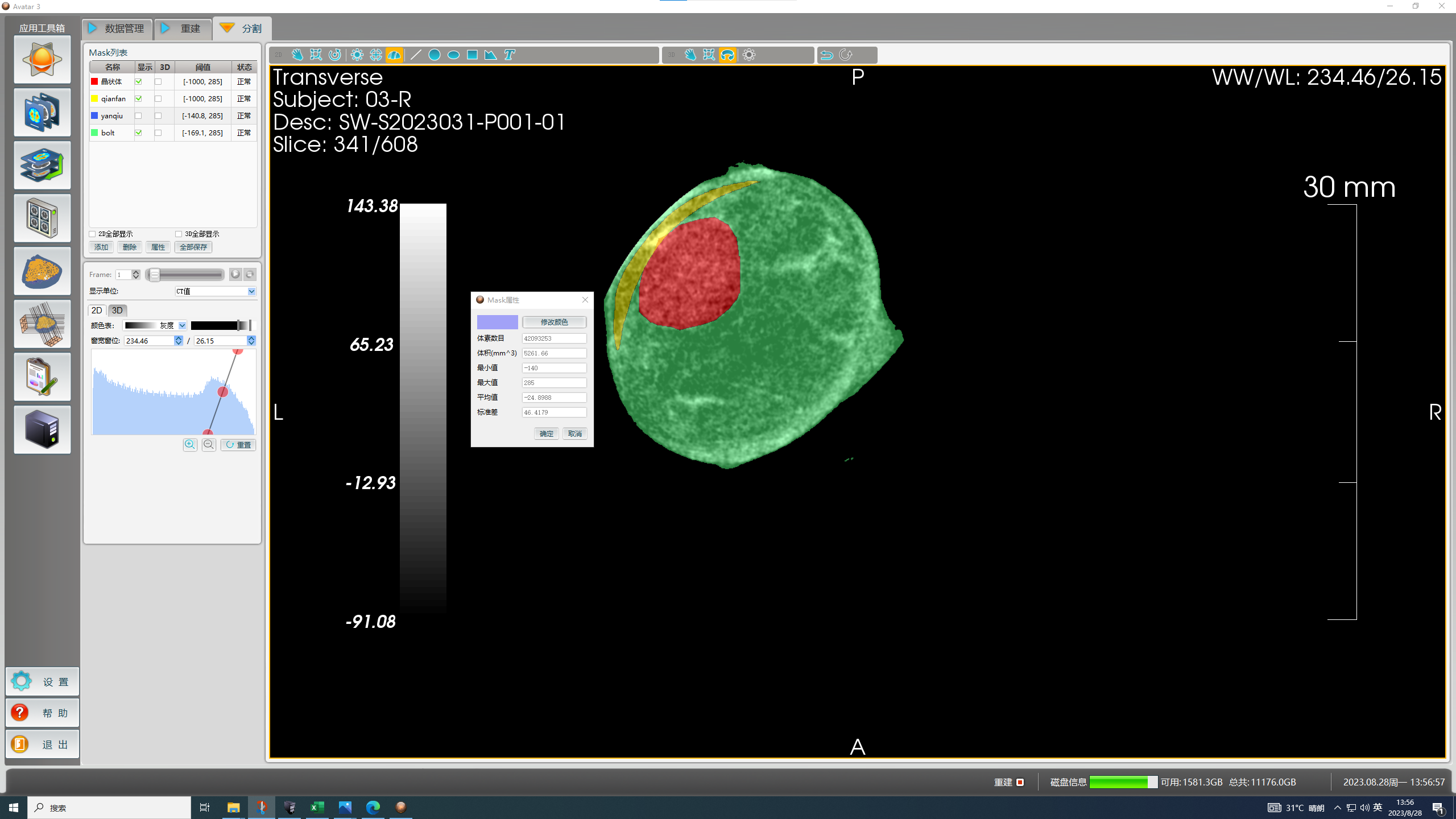

Supplement: S2 Data — (ZIP) [file pone.0310830.s002.zip › CT_pigs/Eyeball volume/03-R.png]

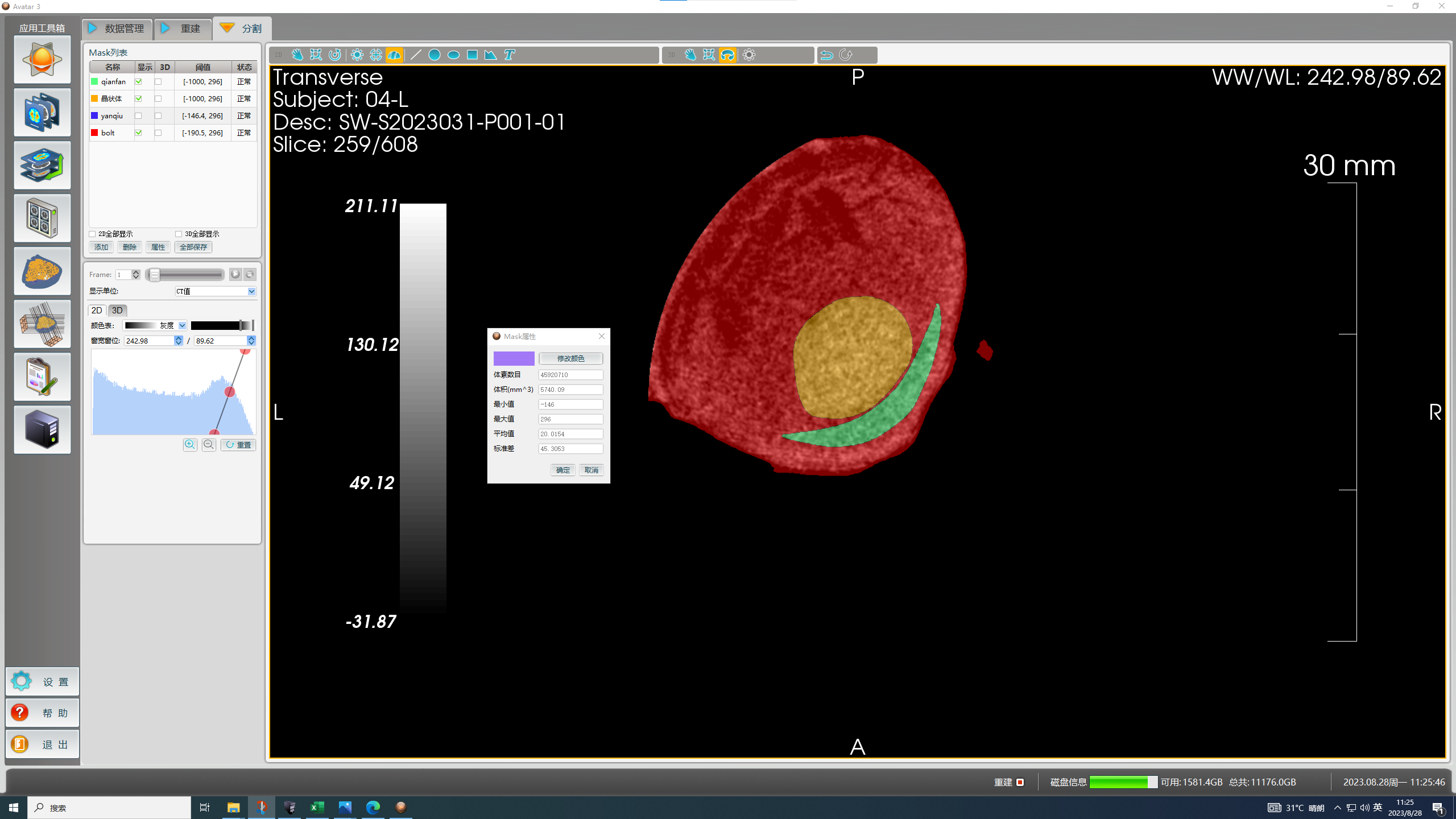

Supplement: S2 Data — (ZIP) [file pone.0310830.s002.zip › CT_pigs/Eyeball volume/04-L.png]

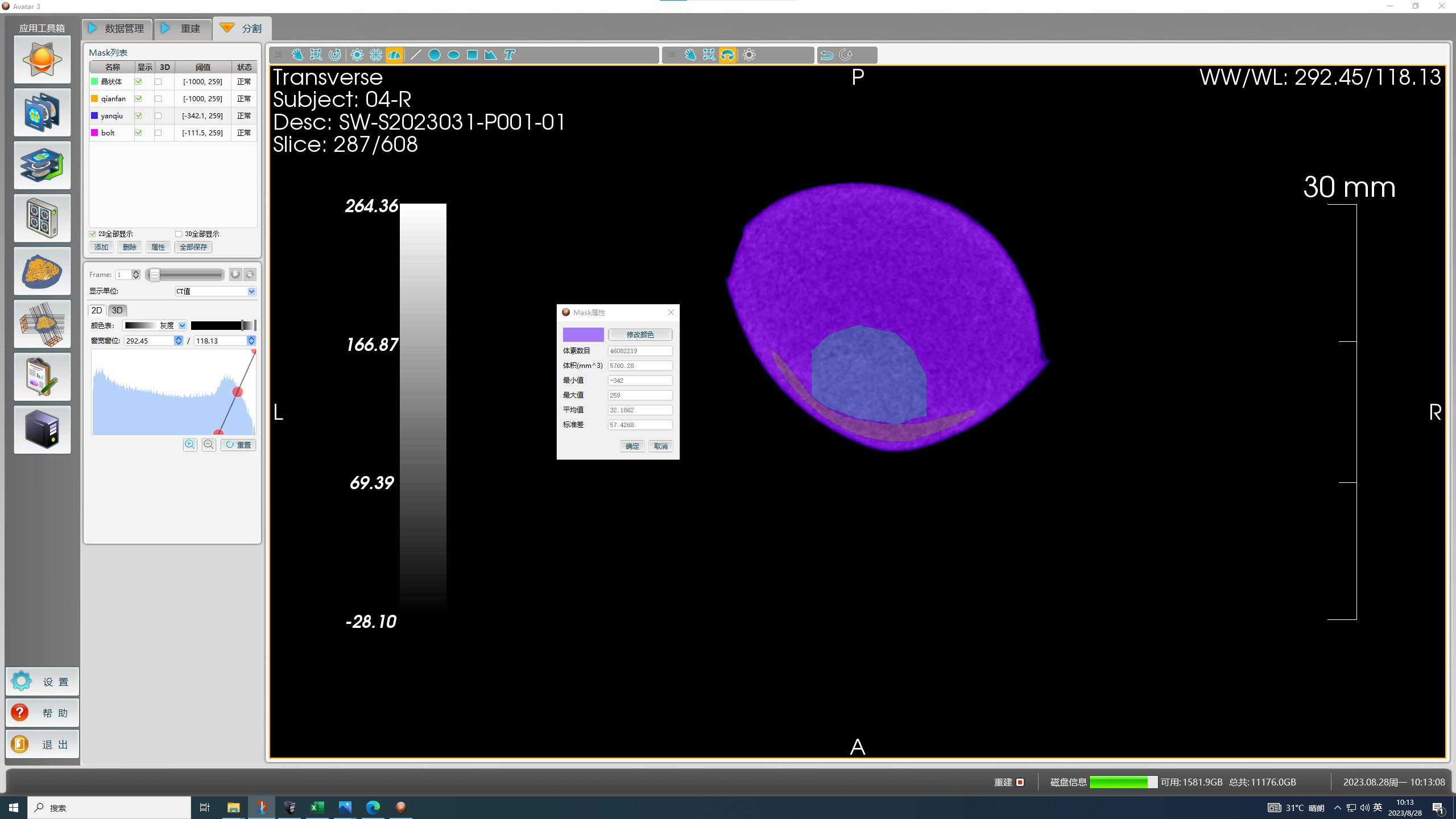

Supplement: S2 Data — (ZIP) [file pone.0310830.s002.zip › CT_pigs/Eyeball volume/04-R.png]

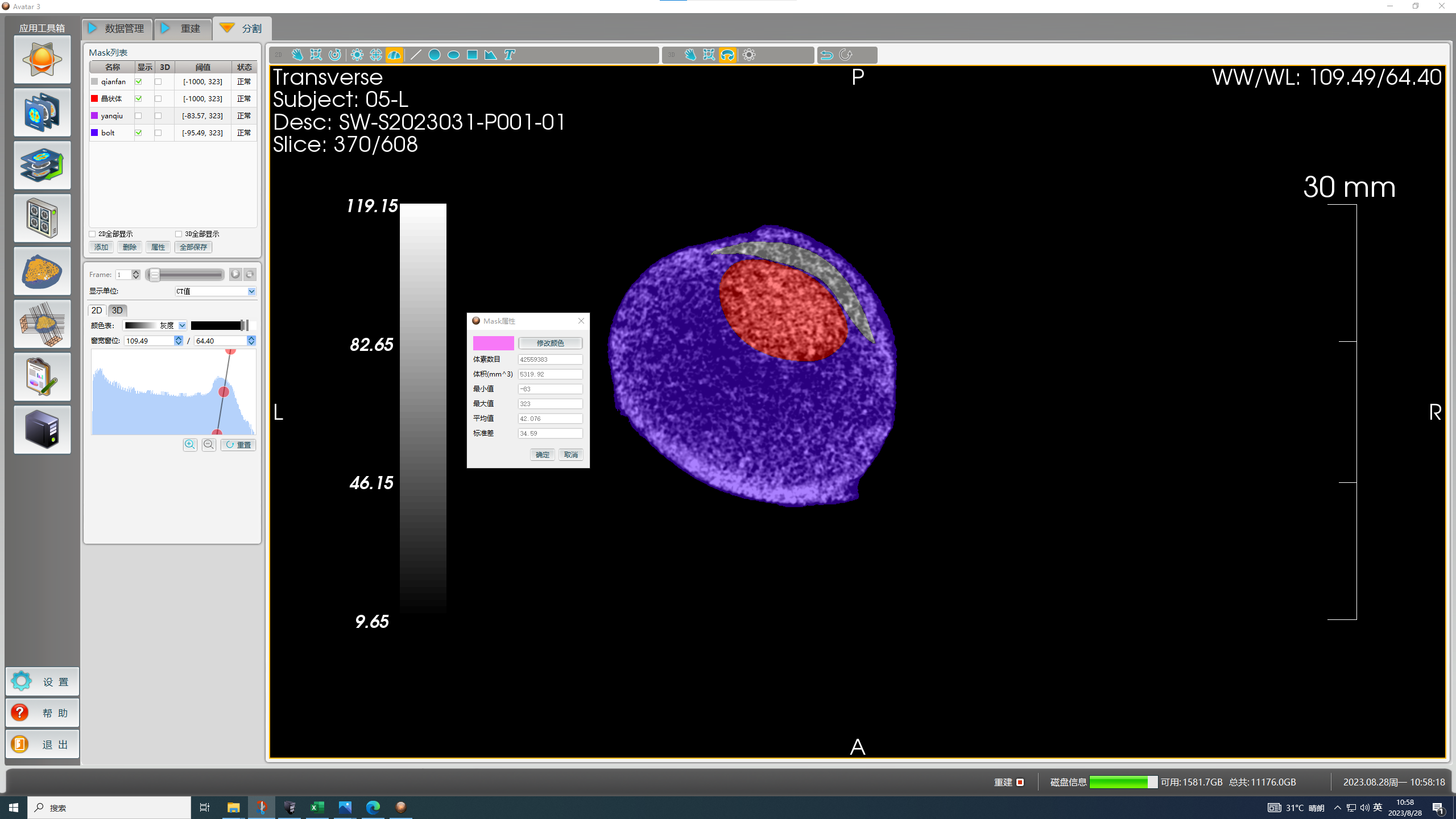

Supplement: S2 Data — (ZIP) [file pone.0310830.s002.zip › CT_pigs/Eyeball volume/05-L.png]

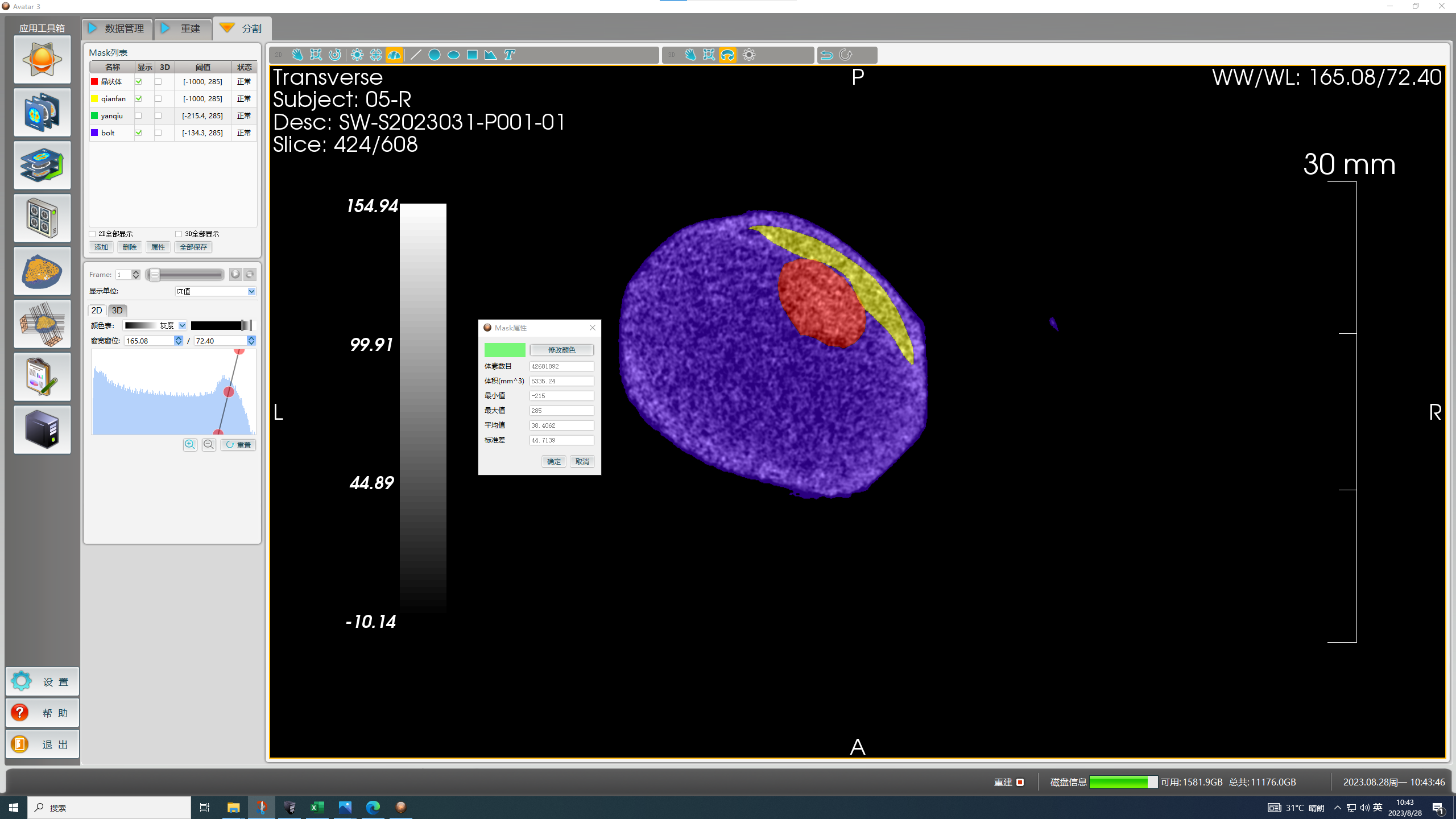

Supplement: S2 Data — (ZIP) [file pone.0310830.s002.zip › CT_pigs/Eyeball volume/05-R.png]

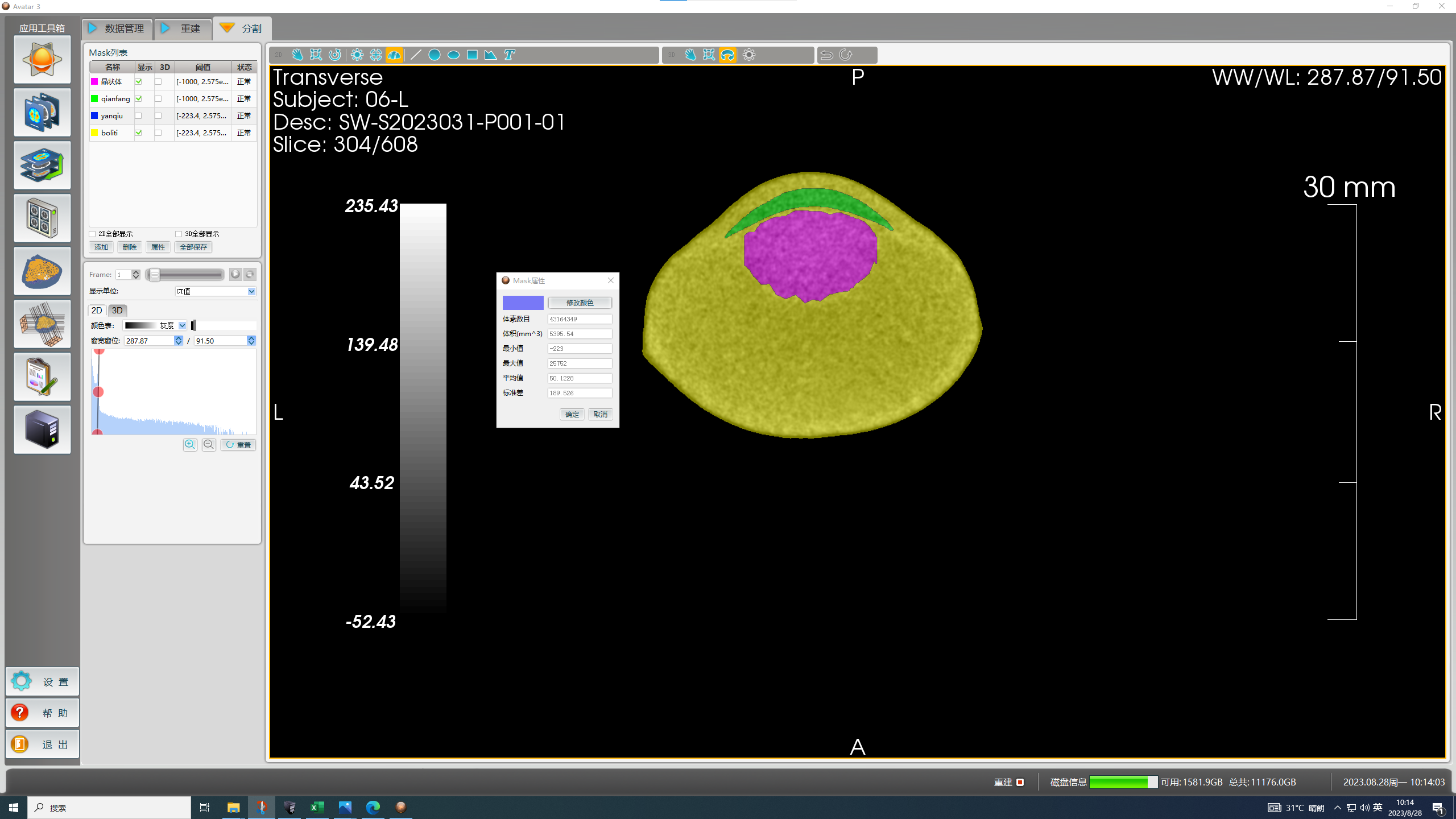

Supplement: S2 Data — (ZIP) [file pone.0310830.s002.zip › CT_pigs/Eyeball volume/06-L.png]

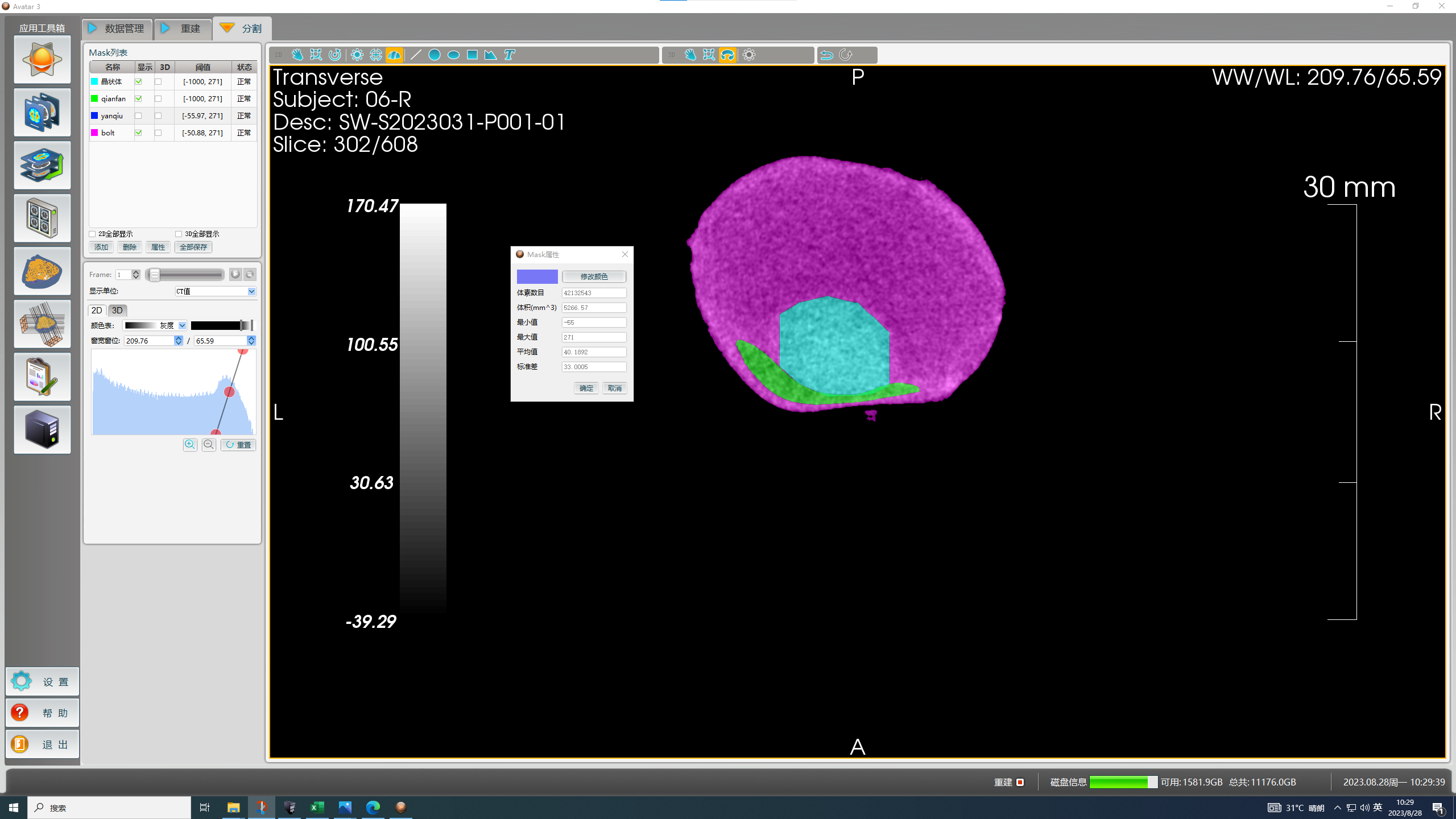

Supplement: S2 Data — (ZIP) [file pone.0310830.s002.zip › CT_pigs/Eyeball volume/06-R.png]

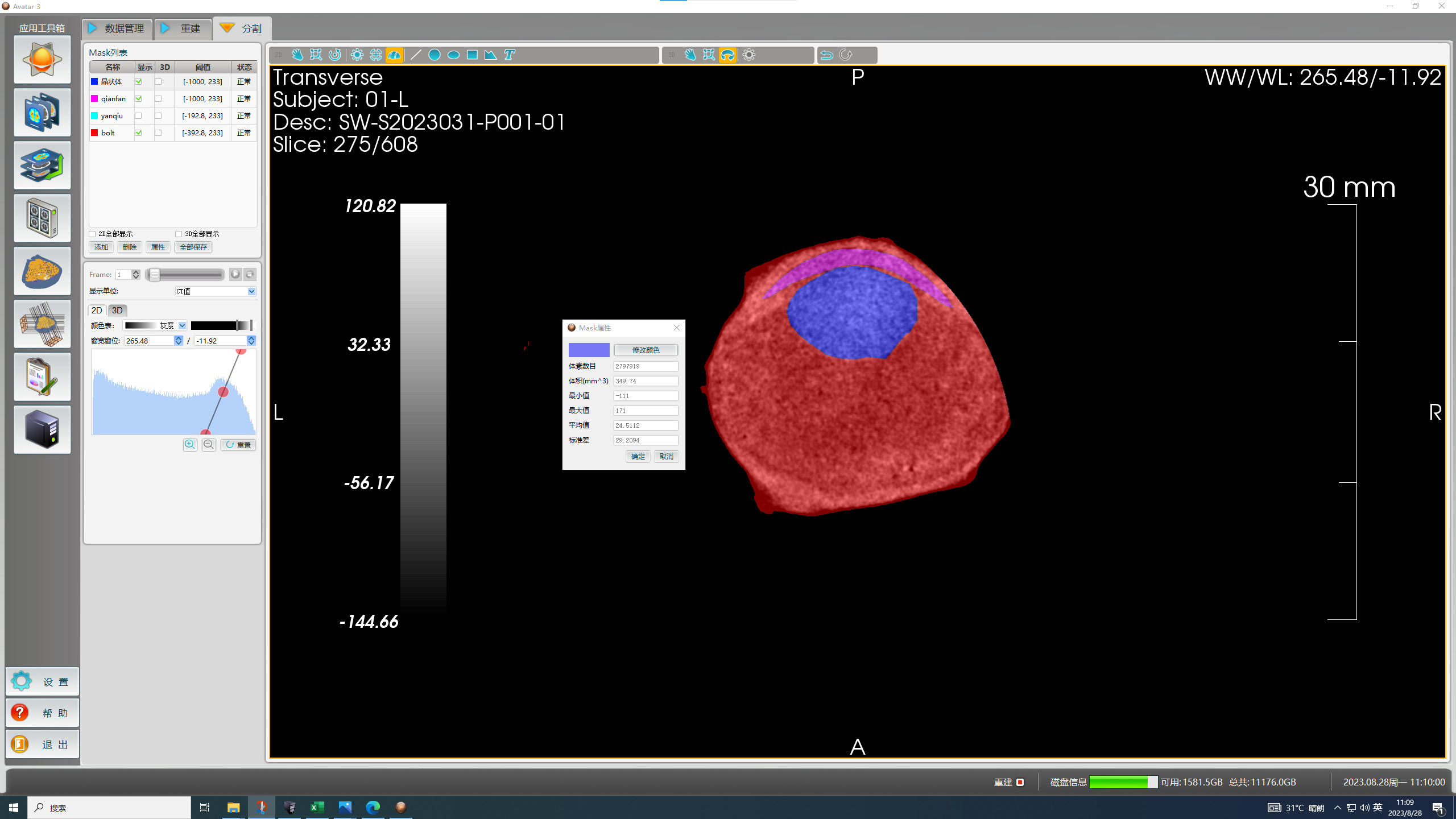

Supplement: S2 Data — (ZIP) [file pone.0310830.s002.zip › CT_pigs/lens/01-L.png]

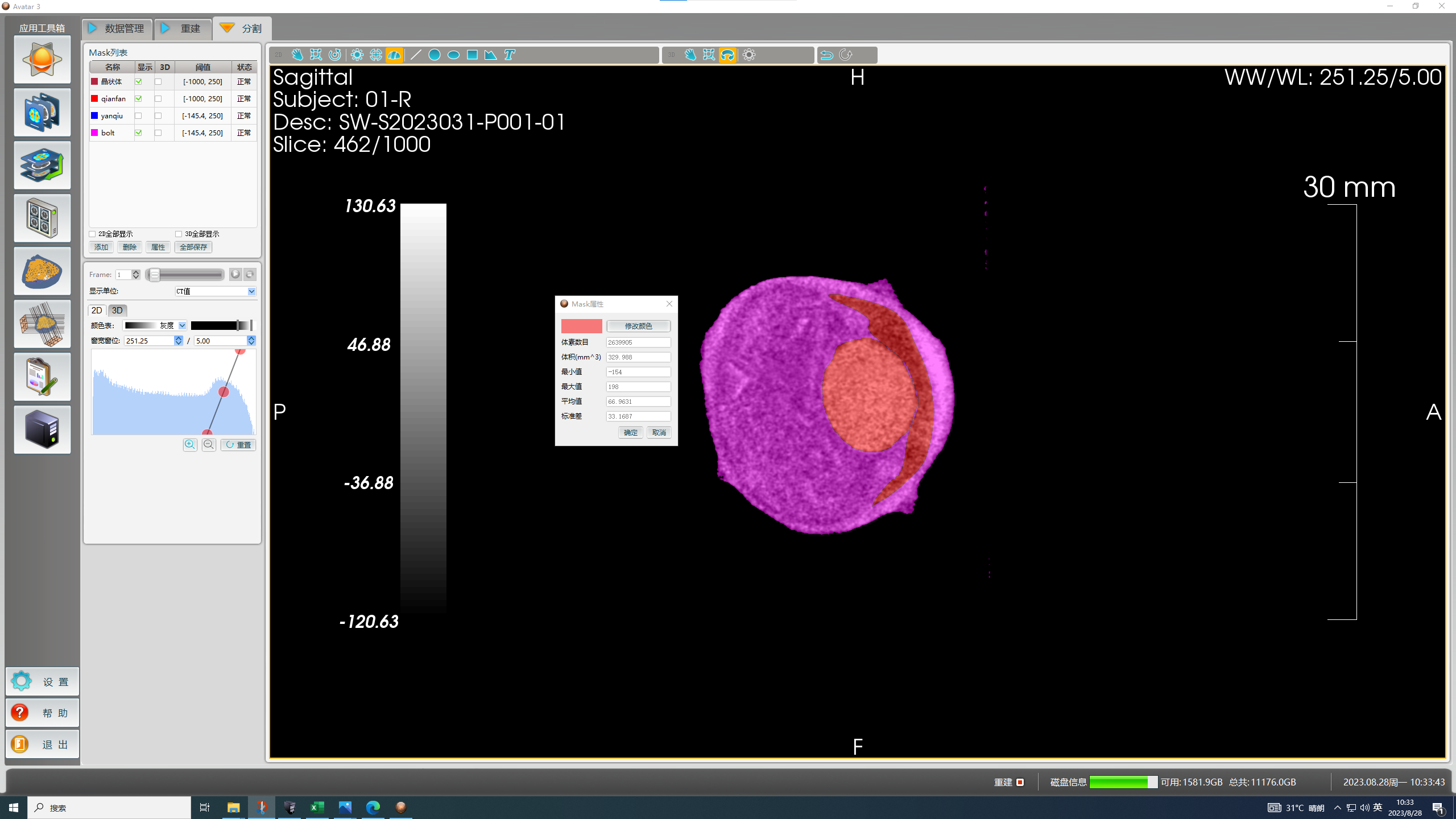

Supplement: S2 Data — (ZIP) [file pone.0310830.s002.zip › CT_pigs/lens/01-R.png]

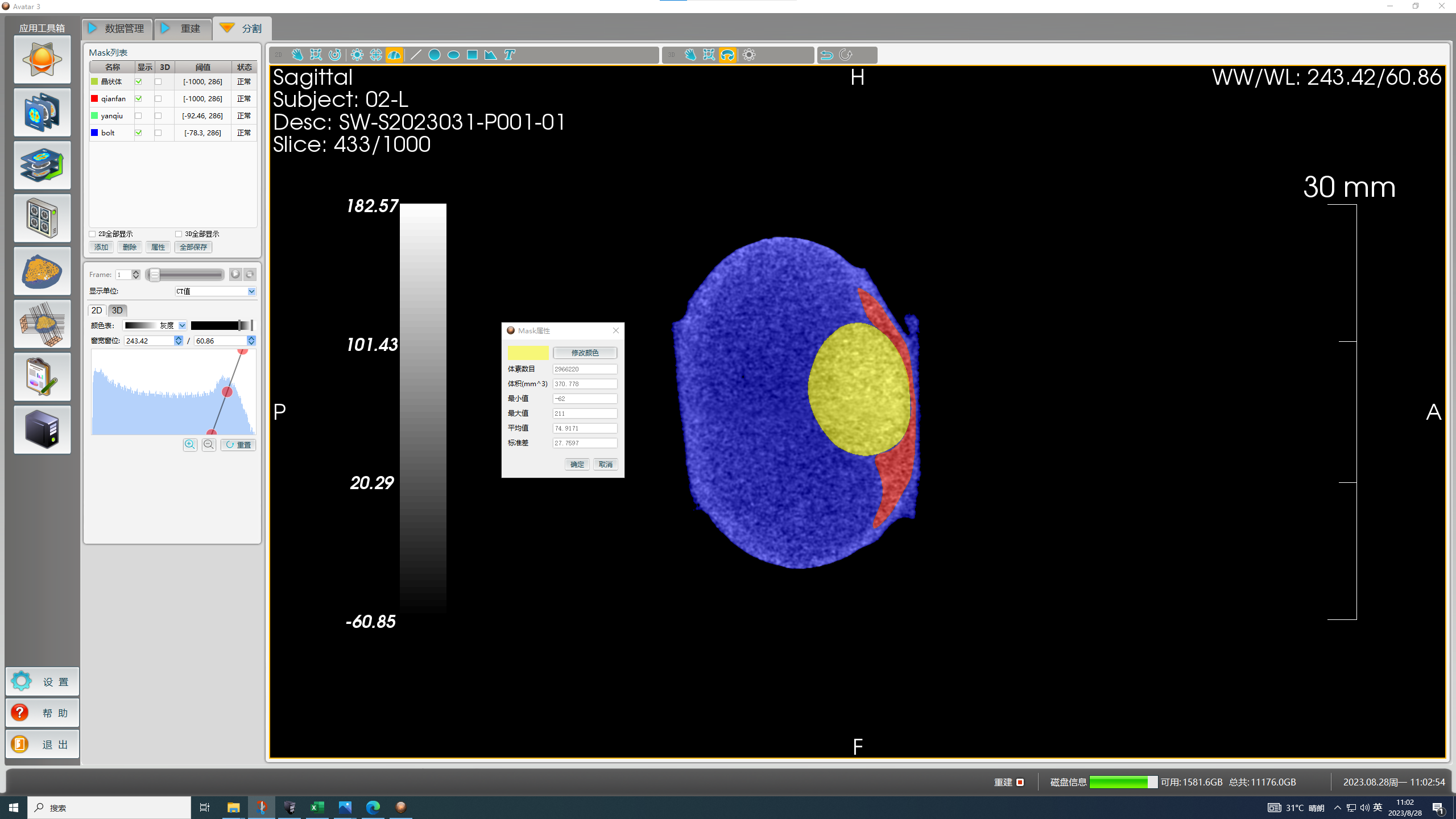

Supplement: S2 Data — (ZIP) [file pone.0310830.s002.zip › CT_pigs/lens/02-L.png]

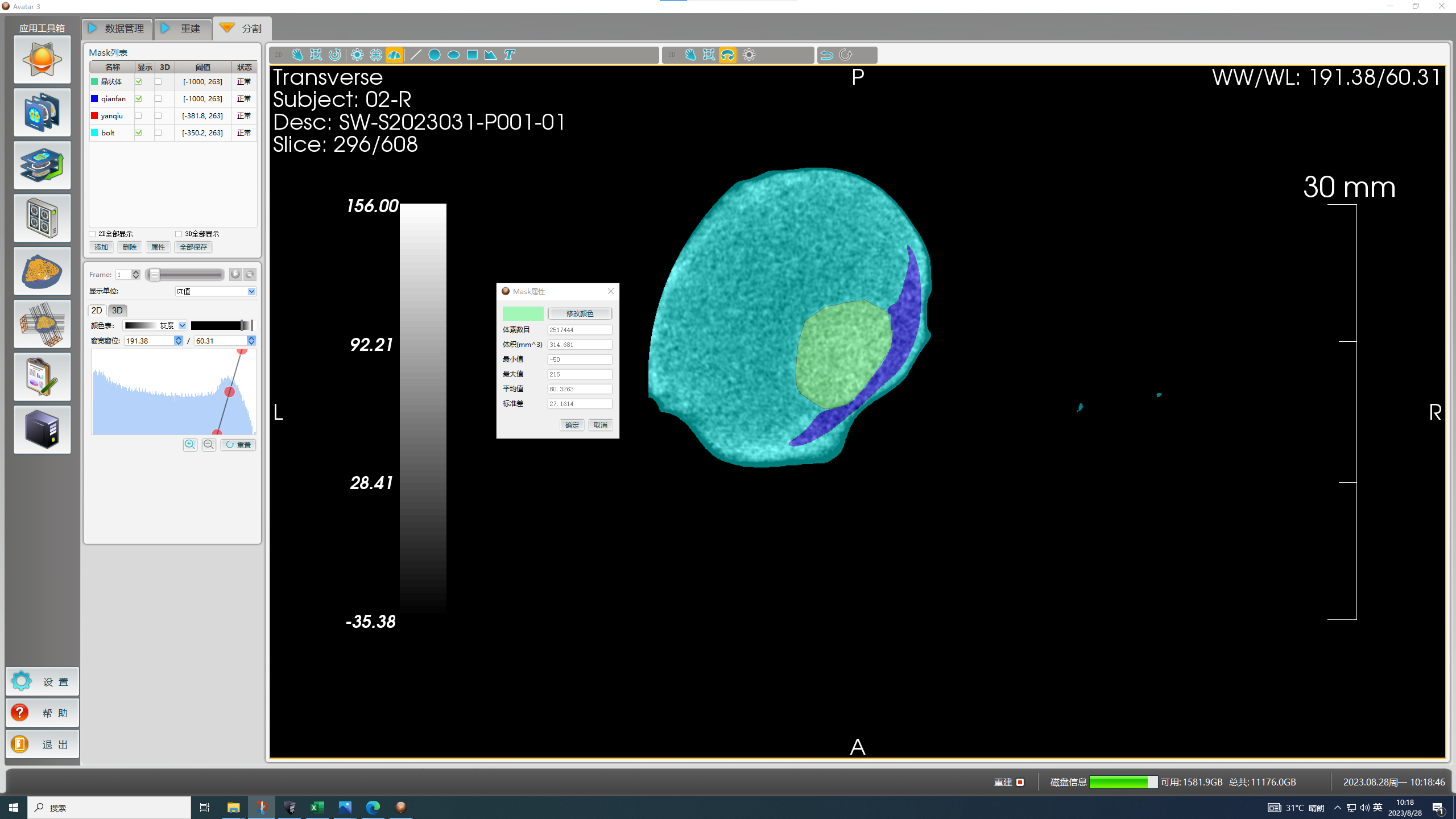

Supplement: S2 Data — (ZIP) [file pone.0310830.s002.zip › CT_pigs/lens/02-R.png]

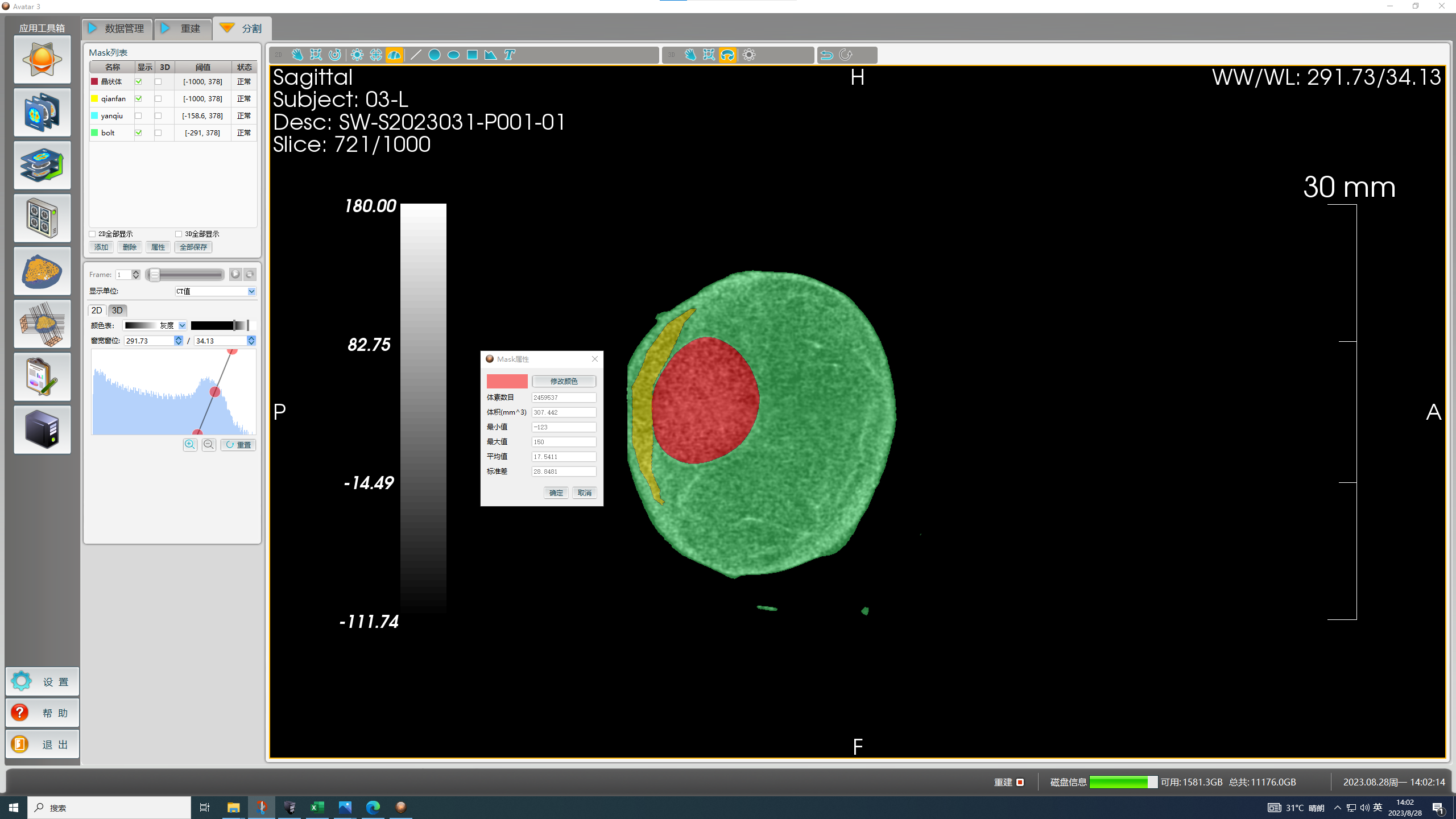

Supplement: S2 Data — (ZIP) [file pone.0310830.s002.zip › CT_pigs/lens/03-L.png]

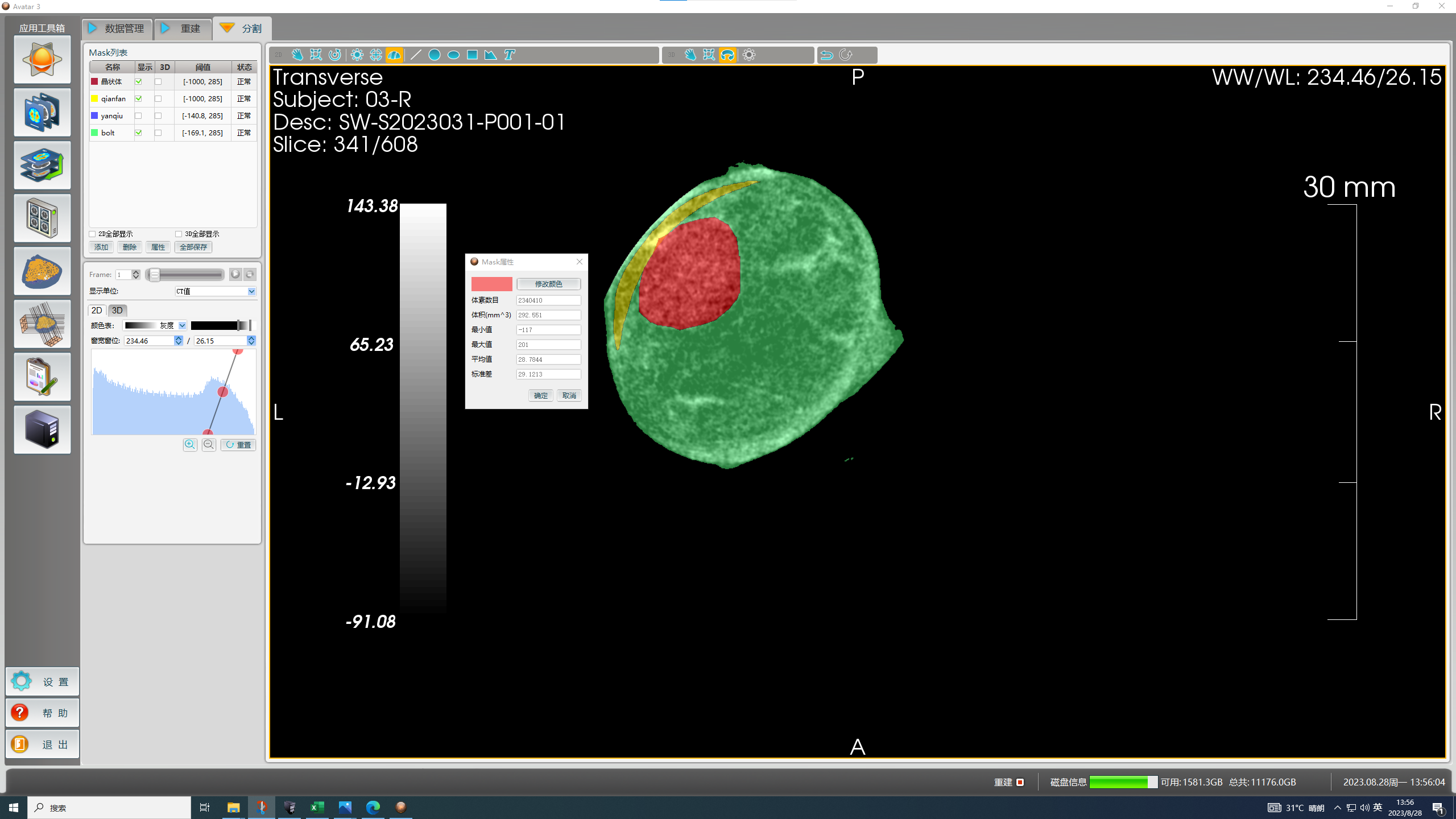

Supplement: S2 Data — (ZIP) [file pone.0310830.s002.zip › CT_pigs/lens/03-R.png]

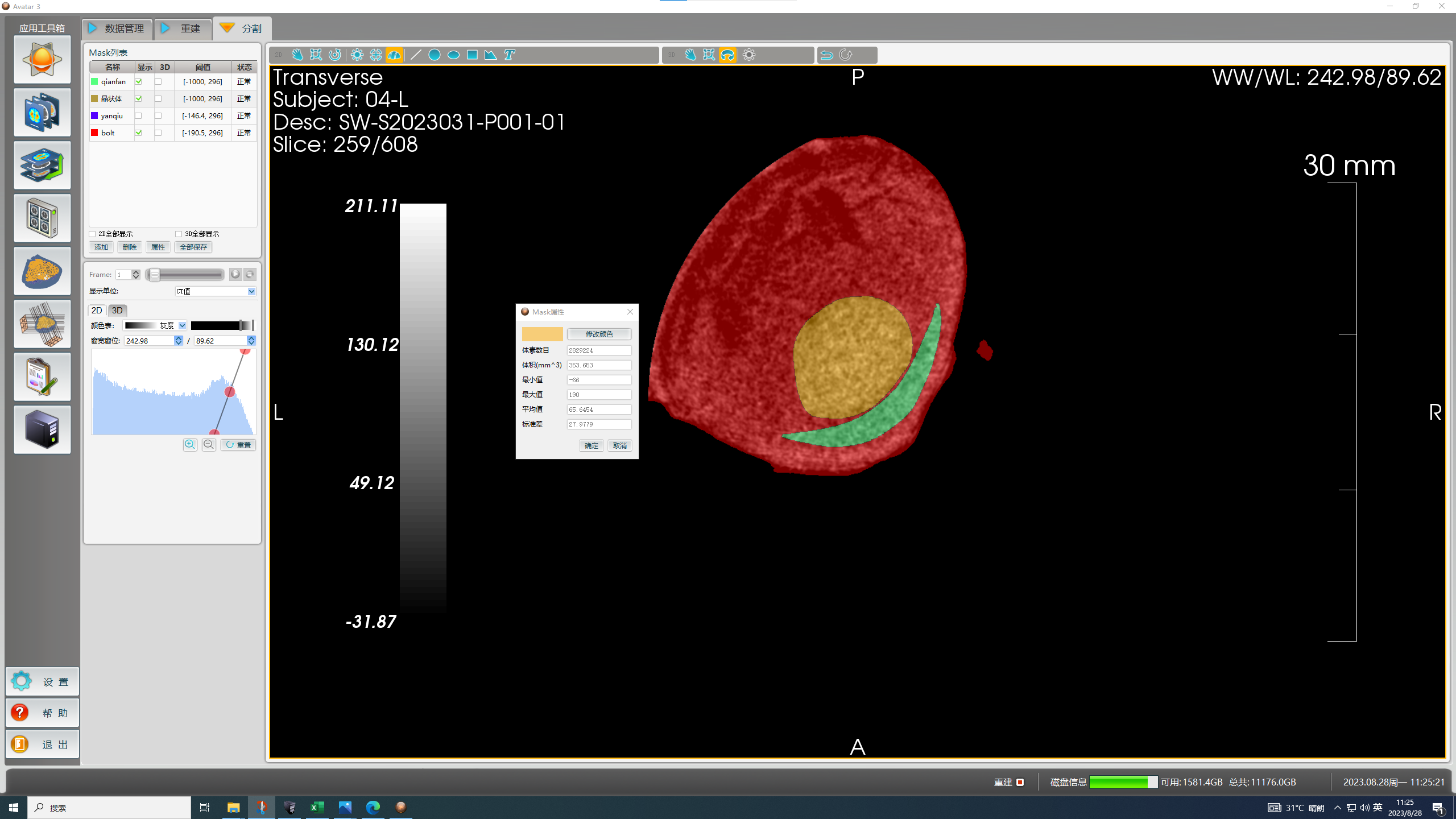

Supplement: S2 Data — (ZIP) [file pone.0310830.s002.zip › CT_pigs/lens/04-L.png]

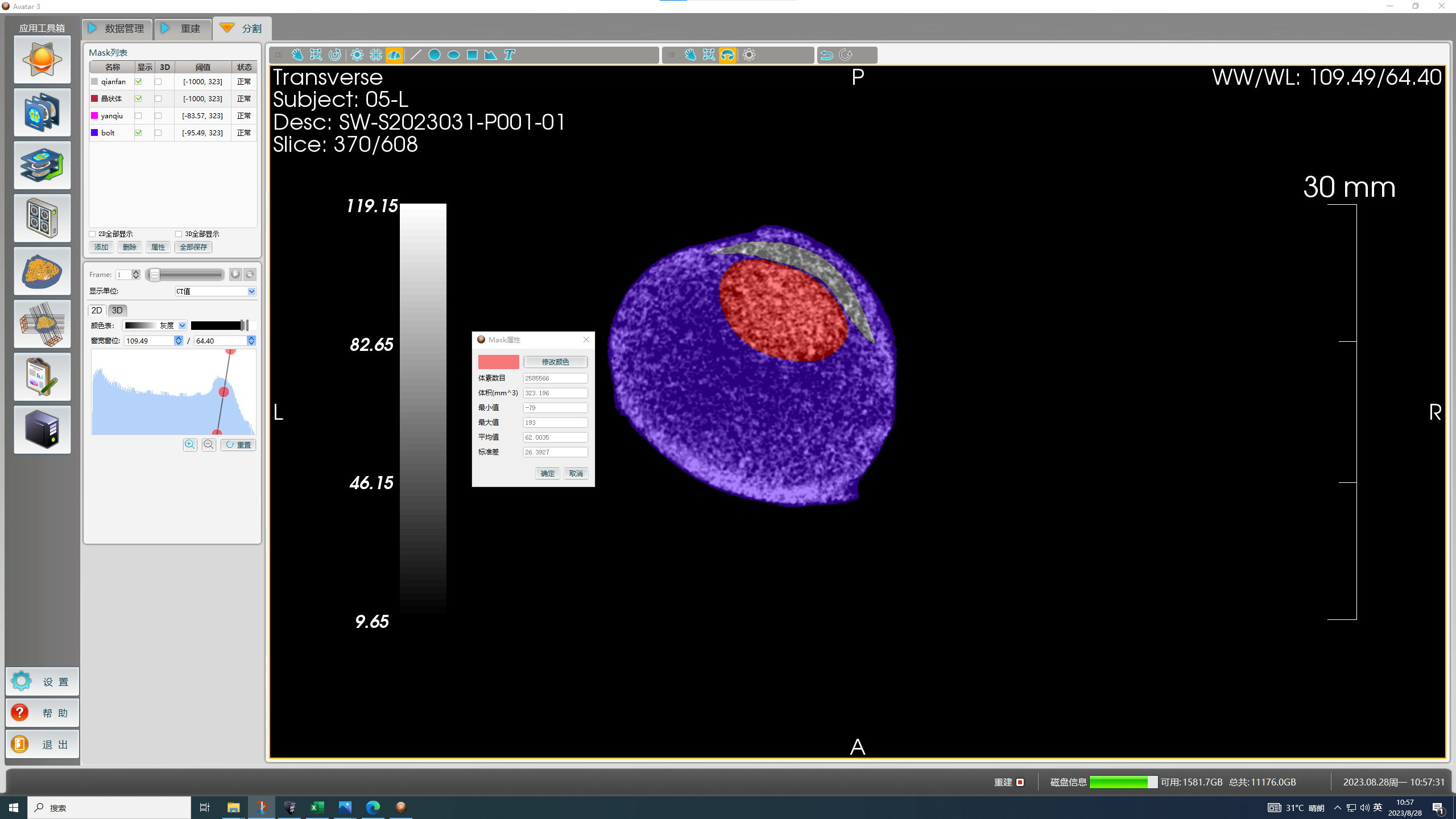

Supplement: S2 Data — (ZIP) [file pone.0310830.s002.zip › CT_pigs/lens/05-L.png]

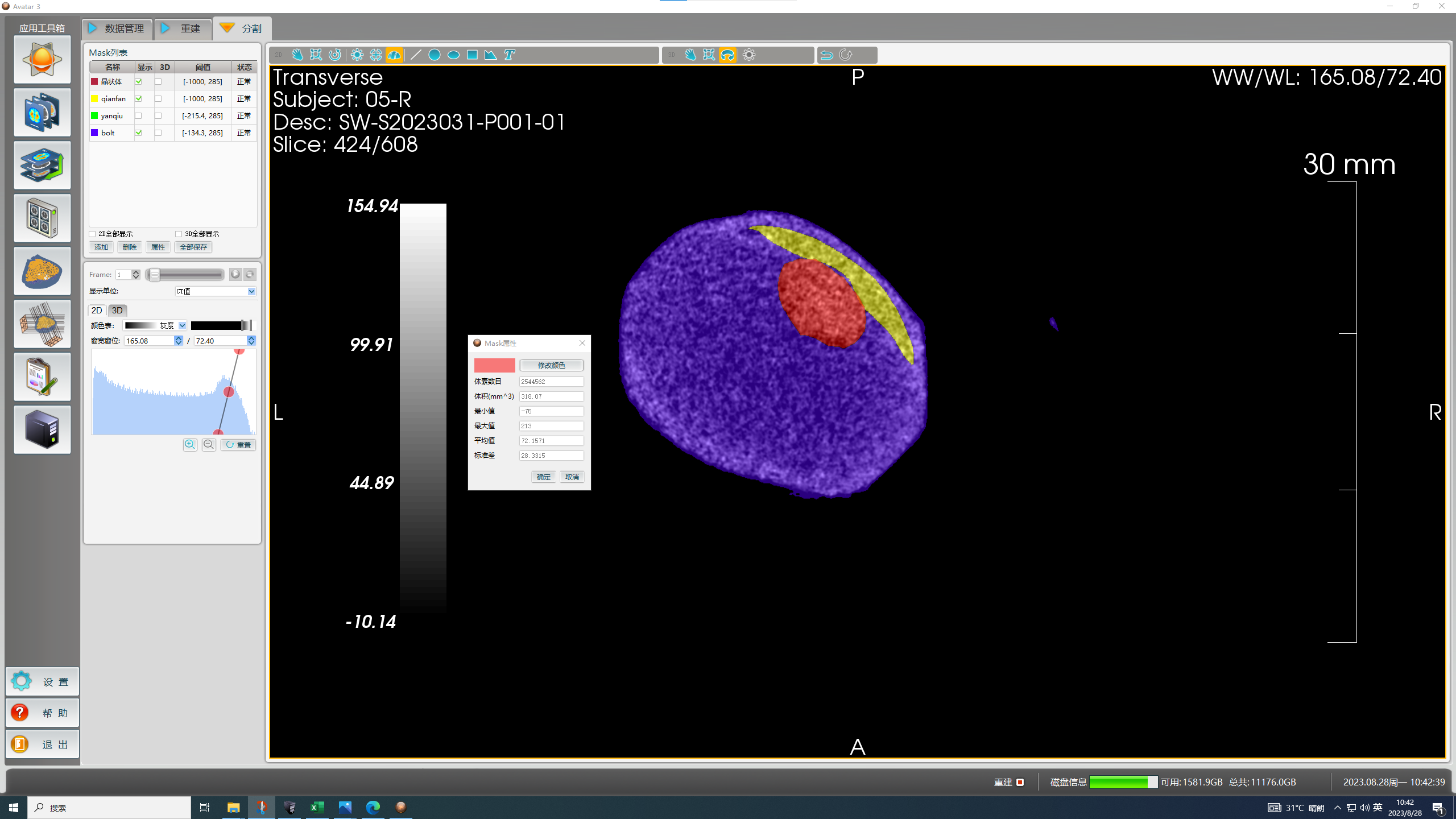

Supplement: S2 Data — (ZIP) [file pone.0310830.s002.zip › CT_pigs/lens/05-R.png]

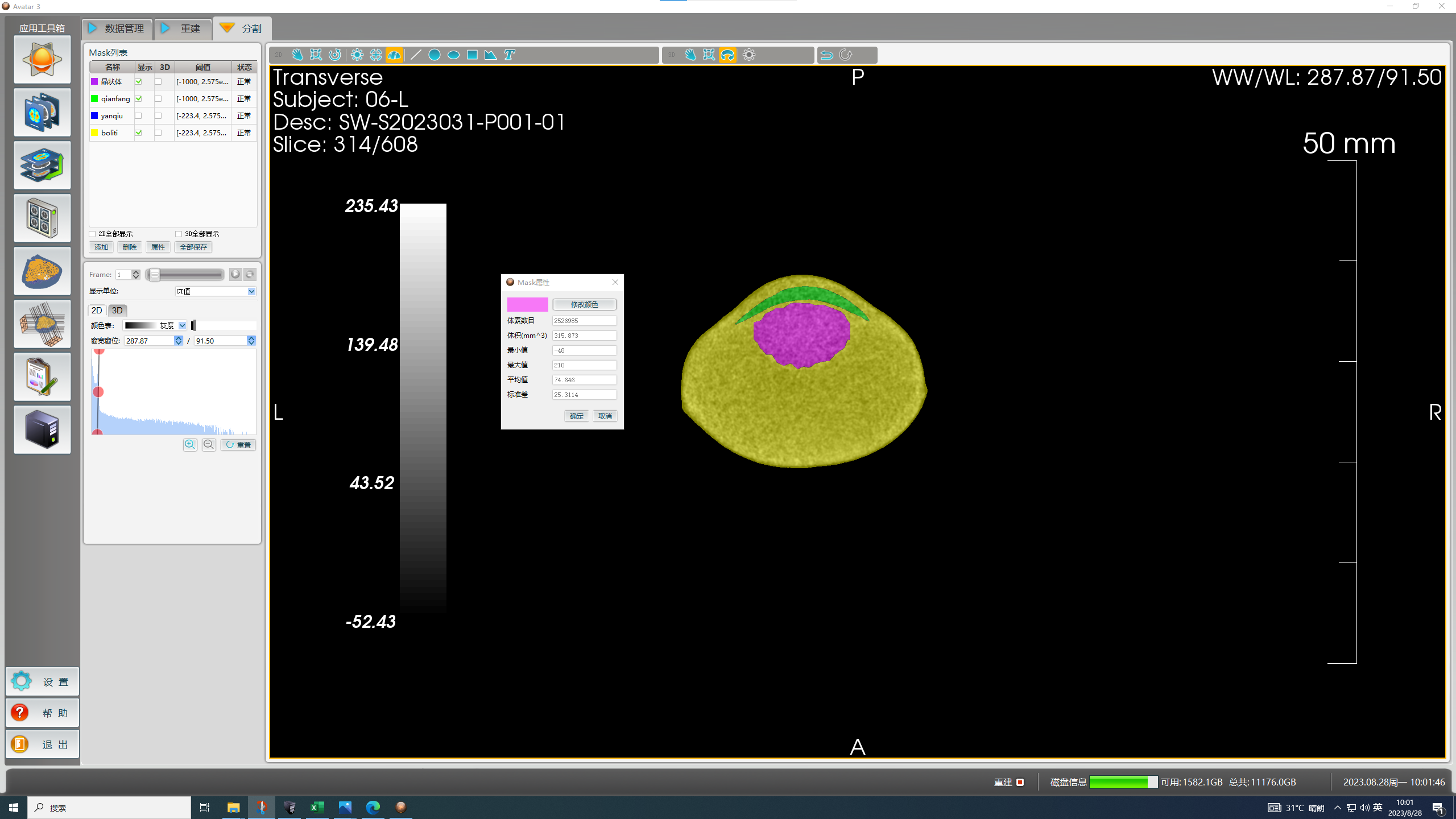

Supplement: S2 Data — (ZIP) [file pone.0310830.s002.zip › CT_pigs/lens/06-L.png]

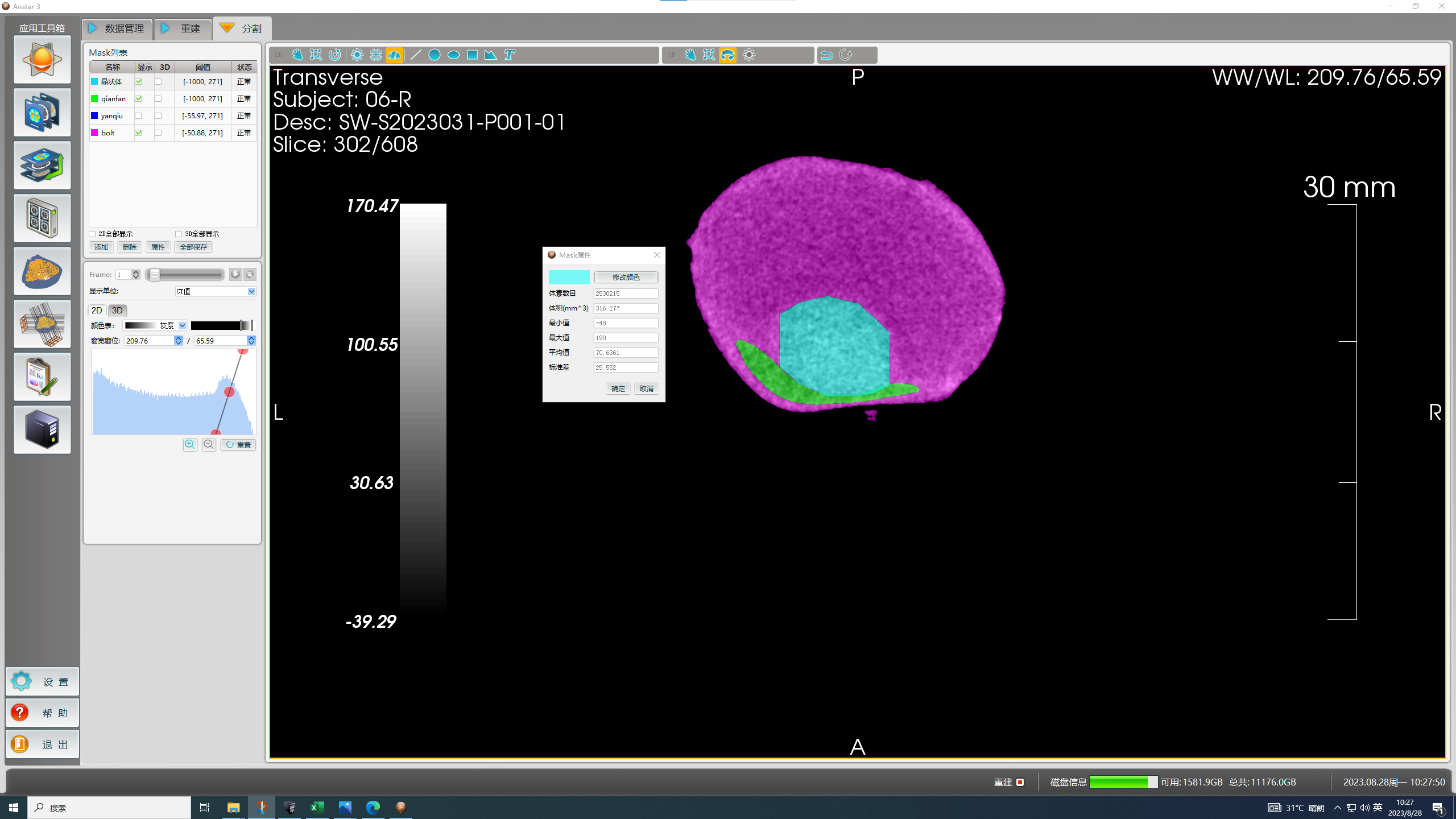

Supplement: S2 Data — (ZIP) [file pone.0310830.s002.zip › CT_pigs/lens/06-R.png]

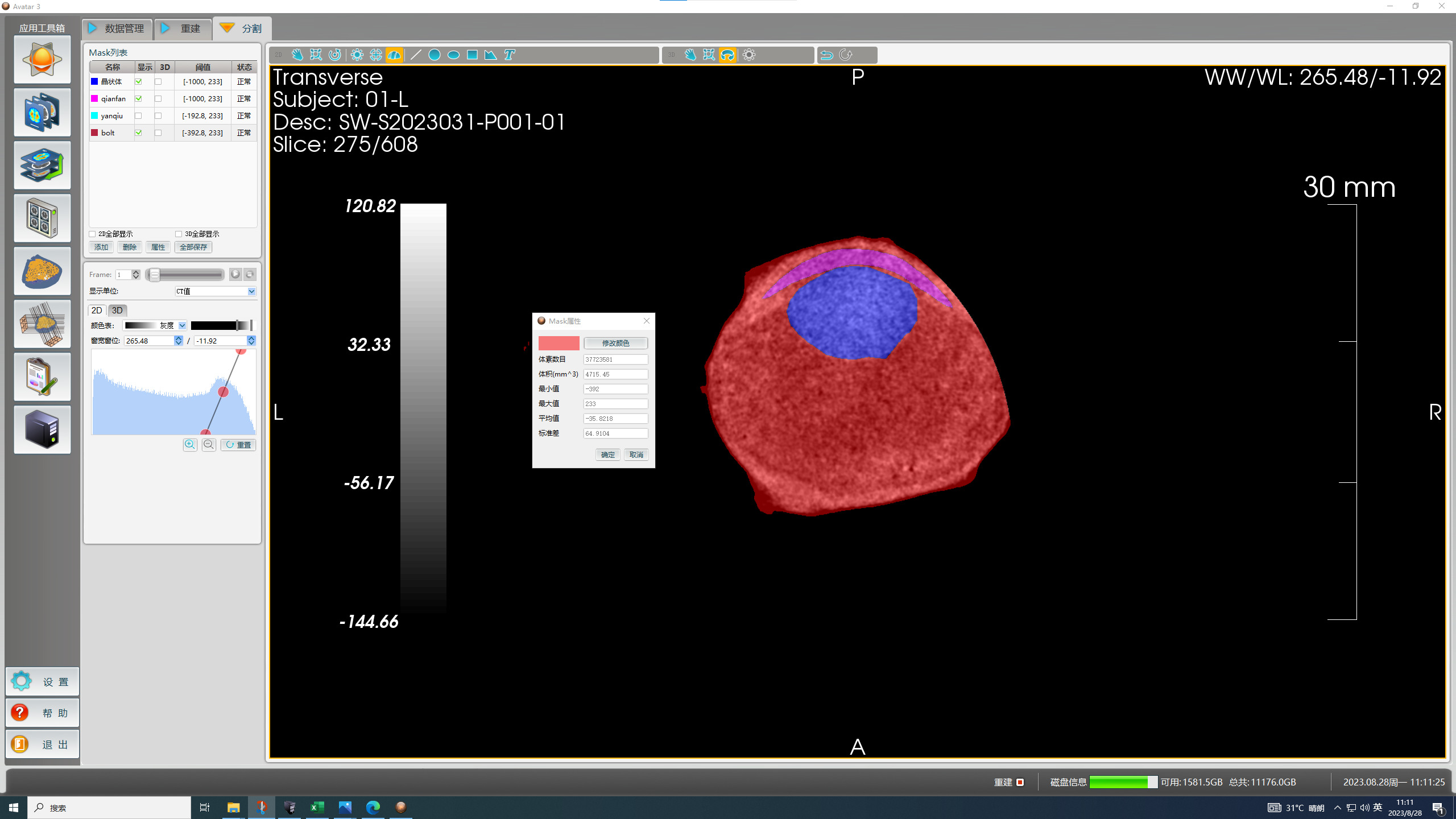

Supplement: S2 Data — (ZIP) [file pone.0310830.s002.zip › CT_pigs/Vitreous body/01-L.png]

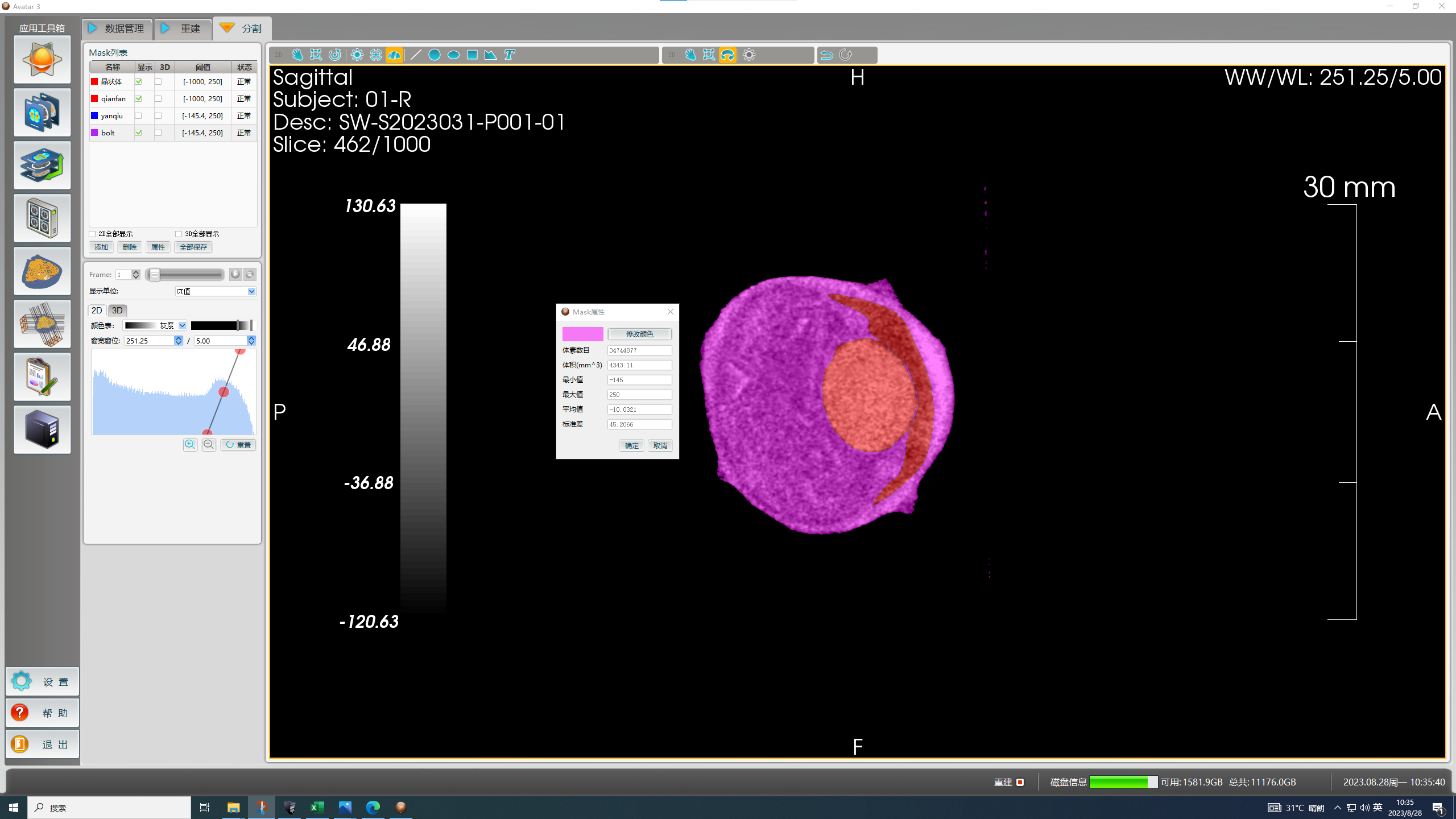

Supplement: S2 Data — (ZIP) [file pone.0310830.s002.zip › CT_pigs/Vitreous body/01-R.png]

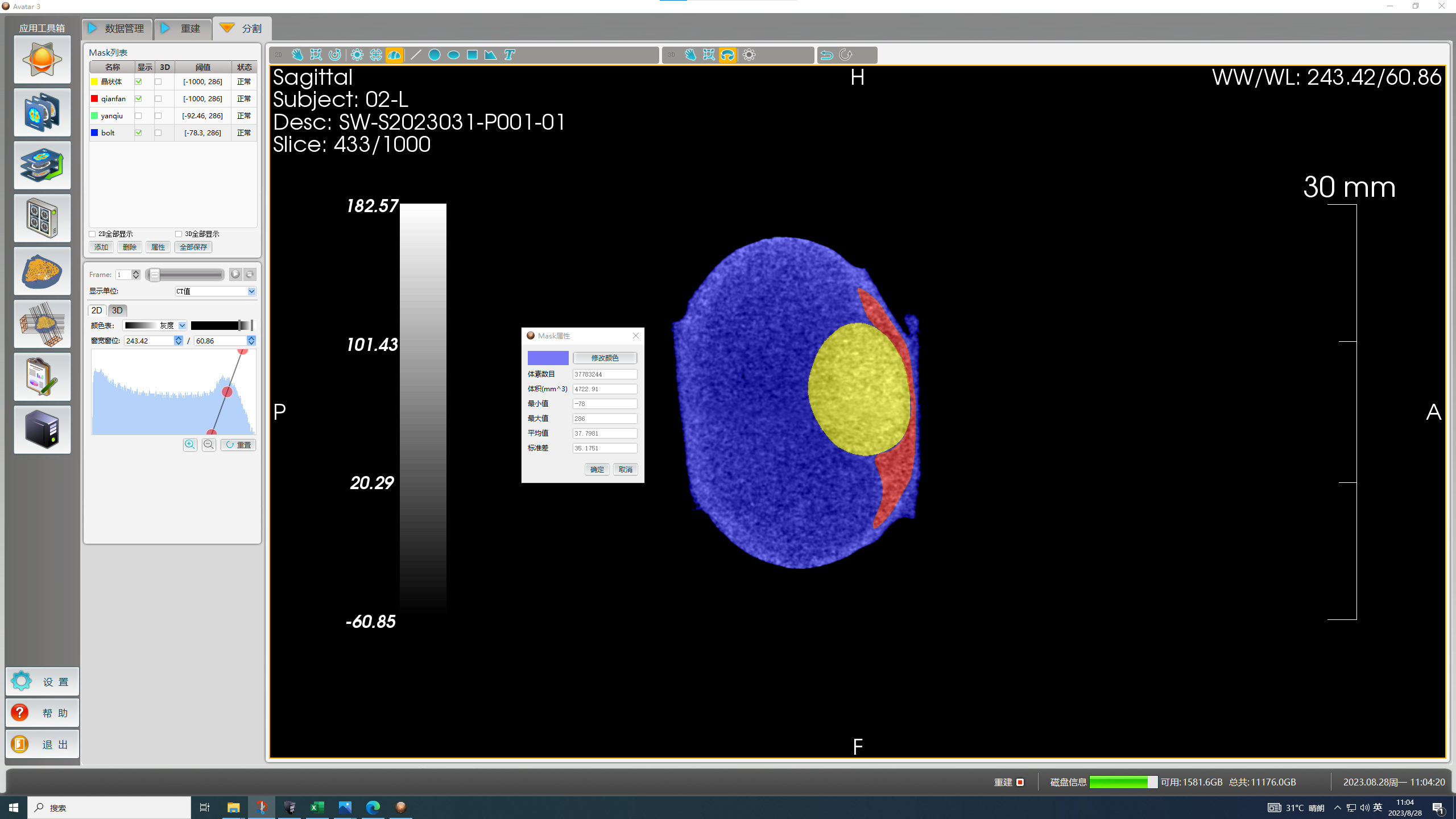

Supplement: S2 Data — (ZIP) [file pone.0310830.s002.zip › CT_pigs/Vitreous body/02-L.png]

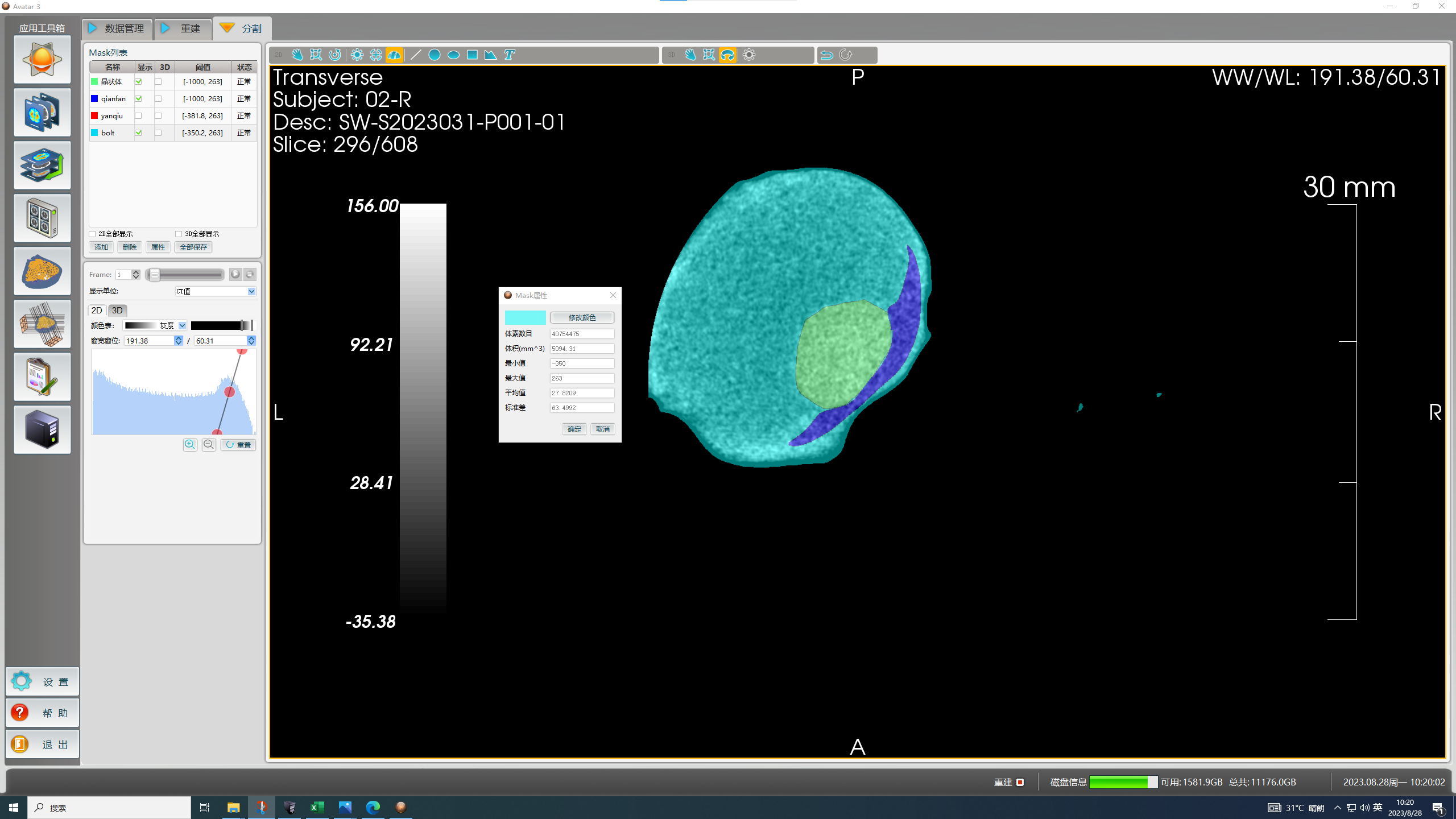

Supplement: S2 Data — (ZIP) [file pone.0310830.s002.zip › CT_pigs/Vitreous body/02-R.png]

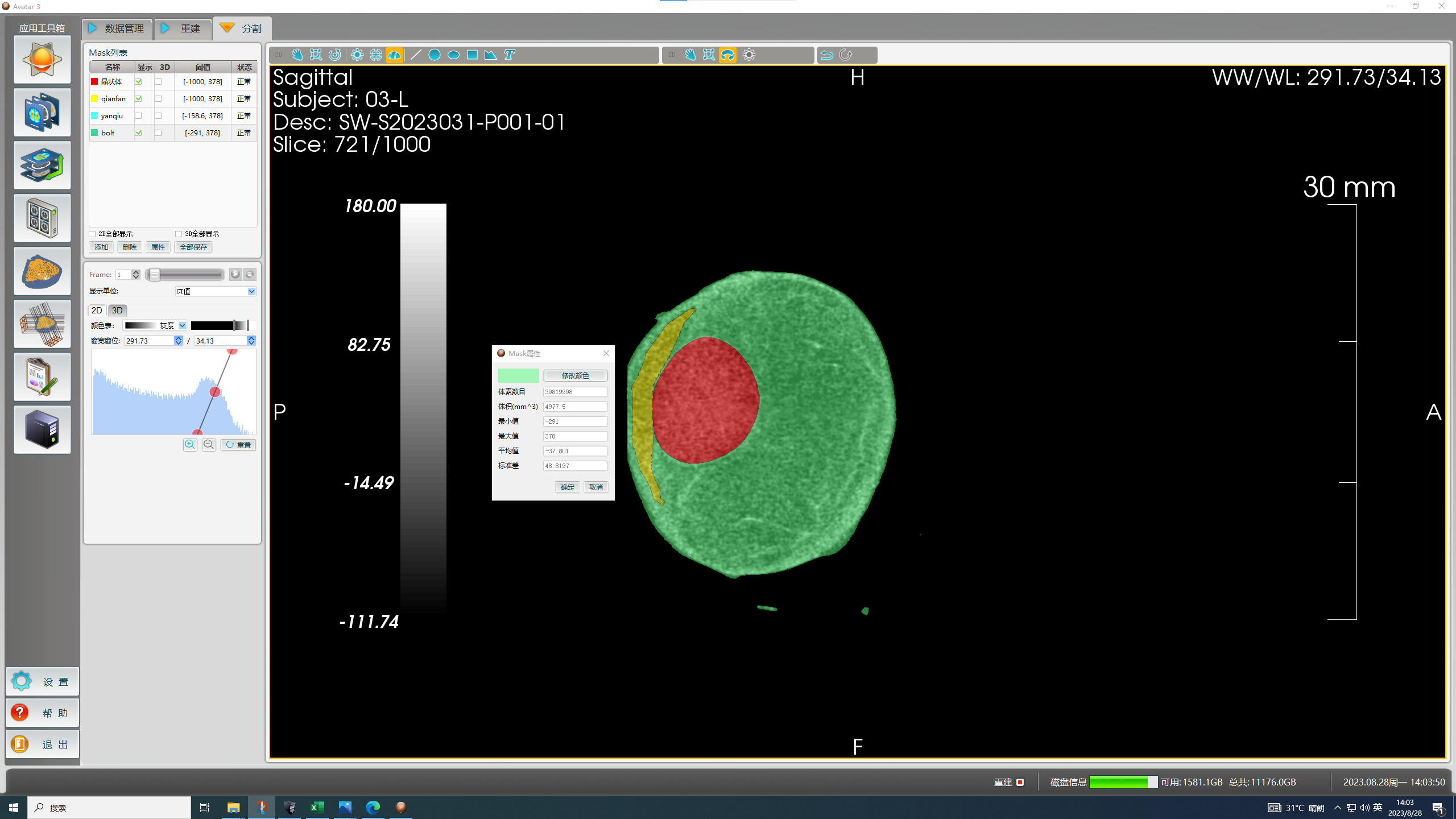

Supplement: S2 Data — (ZIP) [file pone.0310830.s002.zip › CT_pigs/Vitreous body/03-L.png]

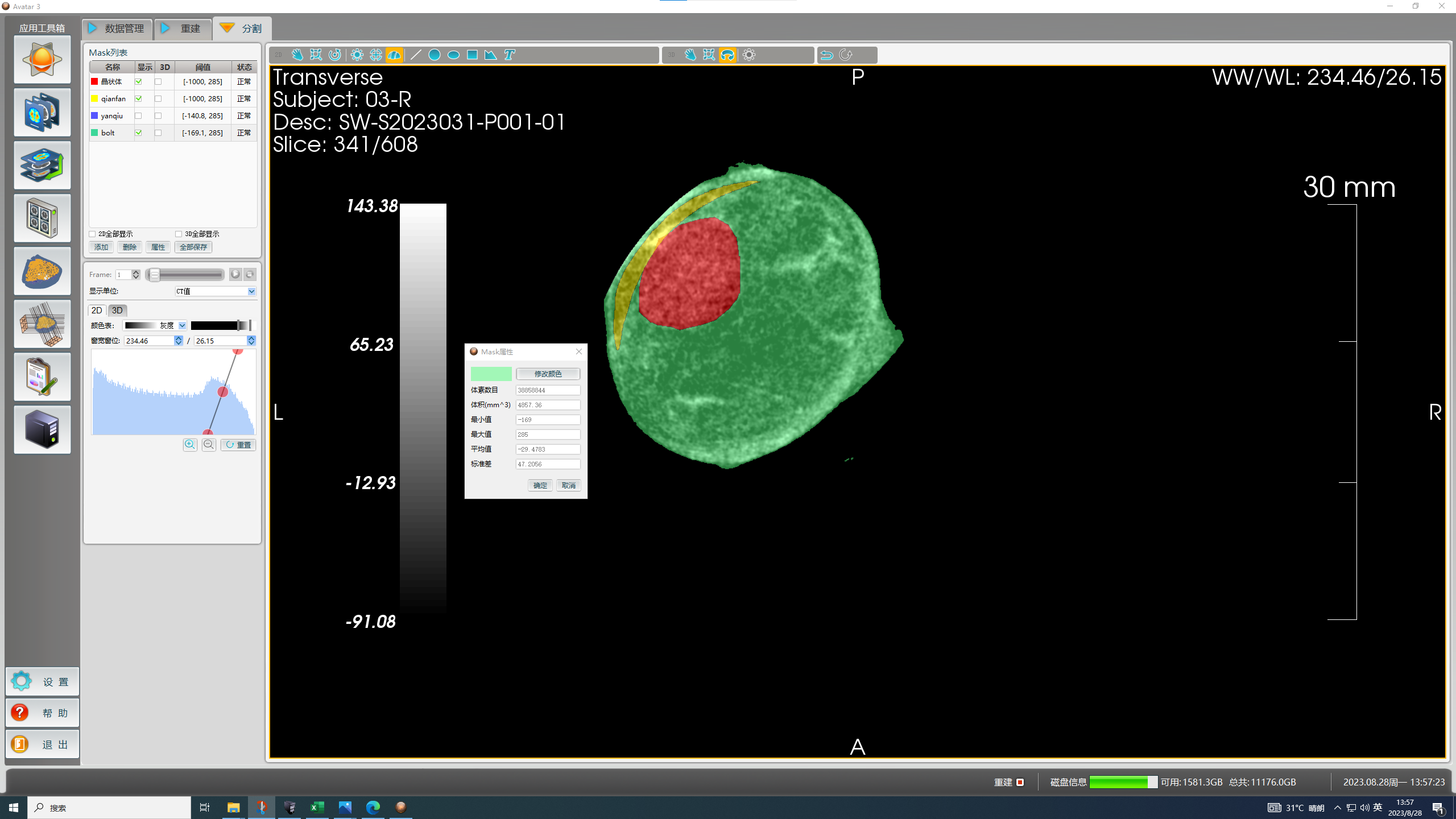

Supplement: S2 Data — (ZIP) [file pone.0310830.s002.zip › CT_pigs/Vitreous body/03-R.png]

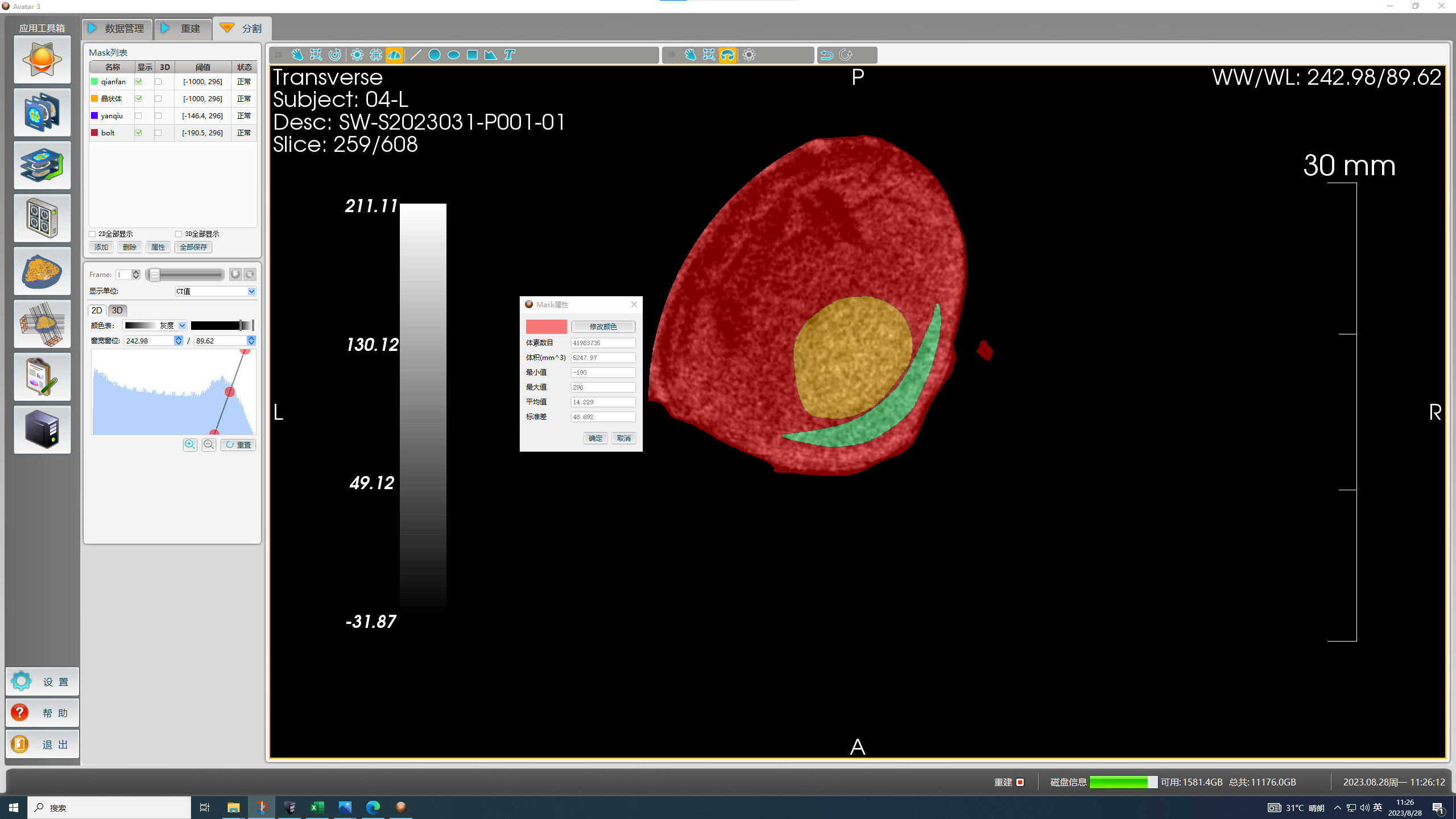

Supplement: S2 Data — (ZIP) [file pone.0310830.s002.zip › CT_pigs/Vitreous body/04-L.png]

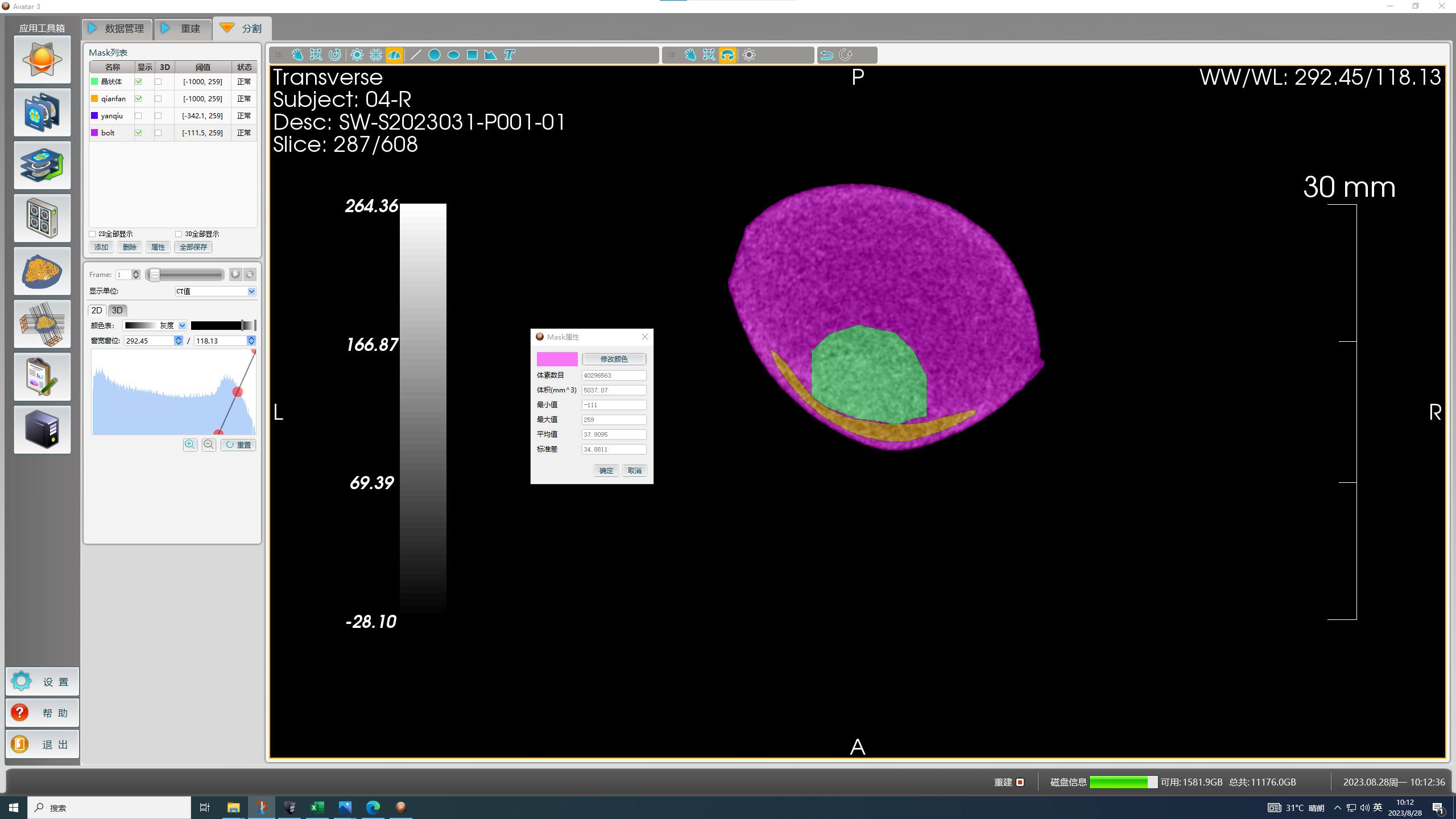

Supplement: S2 Data — (ZIP) [file pone.0310830.s002.zip › CT_pigs/Vitreous body/04-R.png]

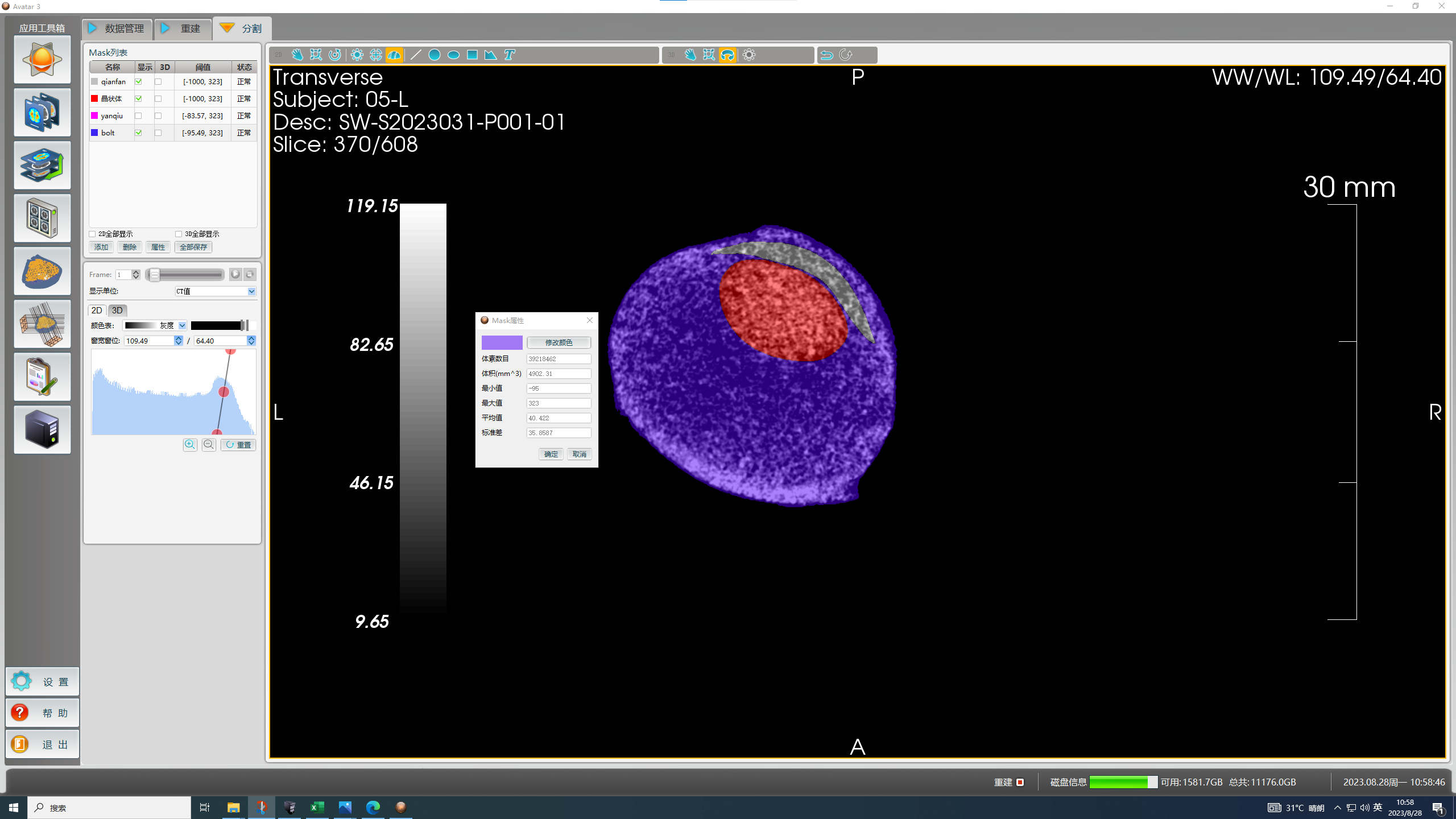

Supplement: S2 Data — (ZIP) [file pone.0310830.s002.zip › CT_pigs/Vitreous body/05-L.png]

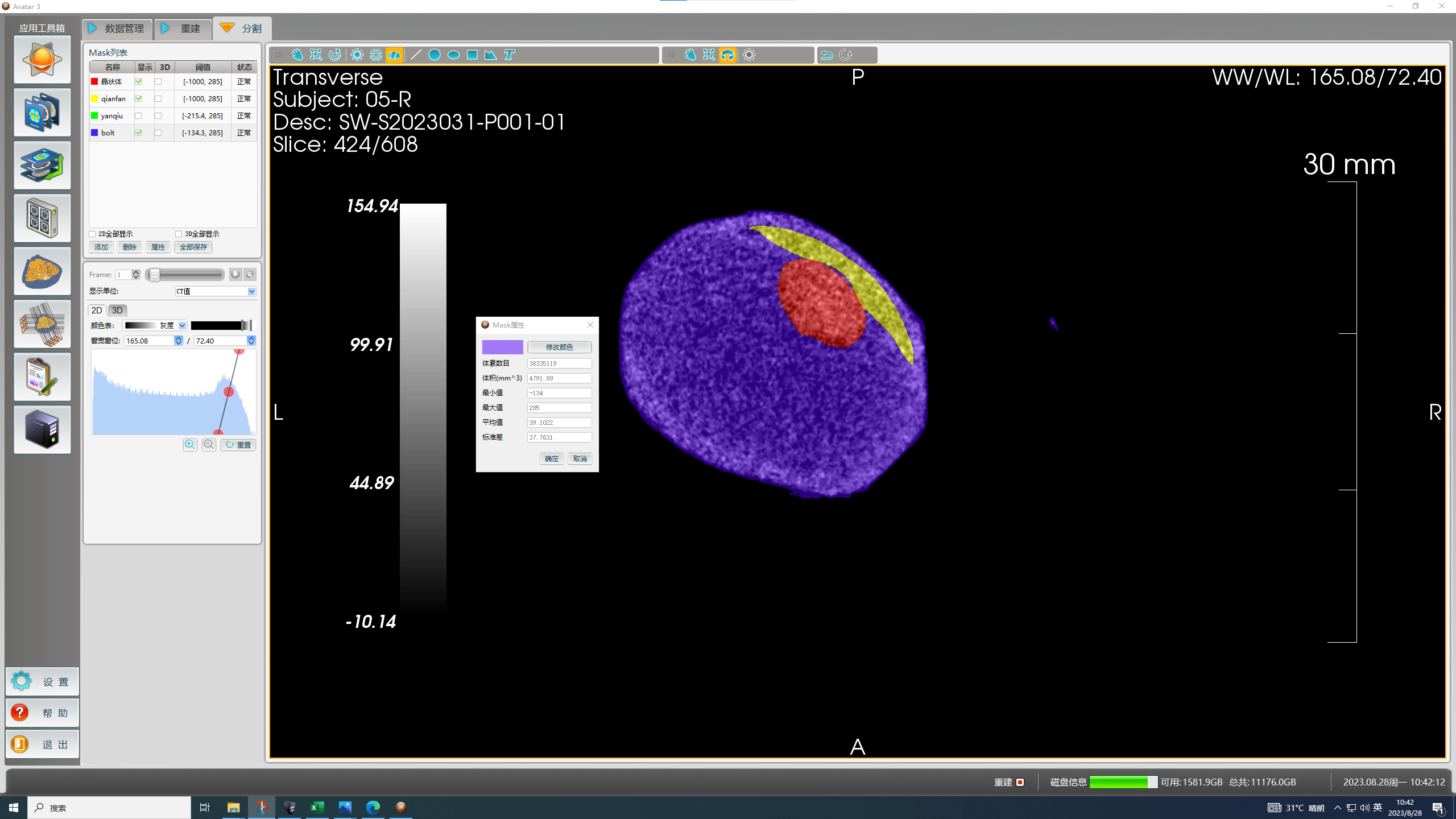

Supplement: S2 Data — (ZIP) [file pone.0310830.s002.zip › CT_pigs/Vitreous body/05-R.png]

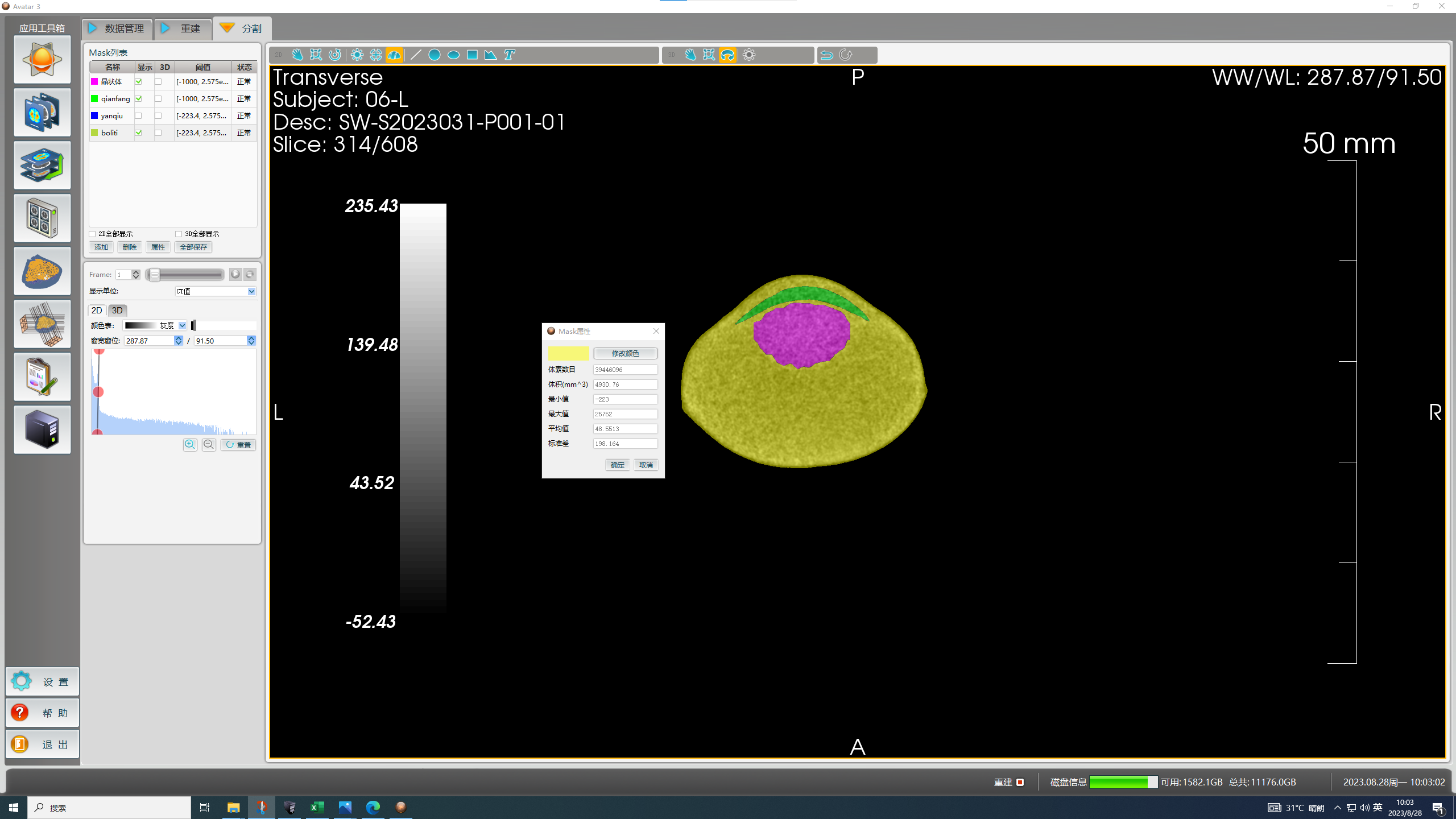

Supplement: S2 Data — (ZIP) [file pone.0310830.s002.zip › CT_pigs/Vitreous body/06-L.png]

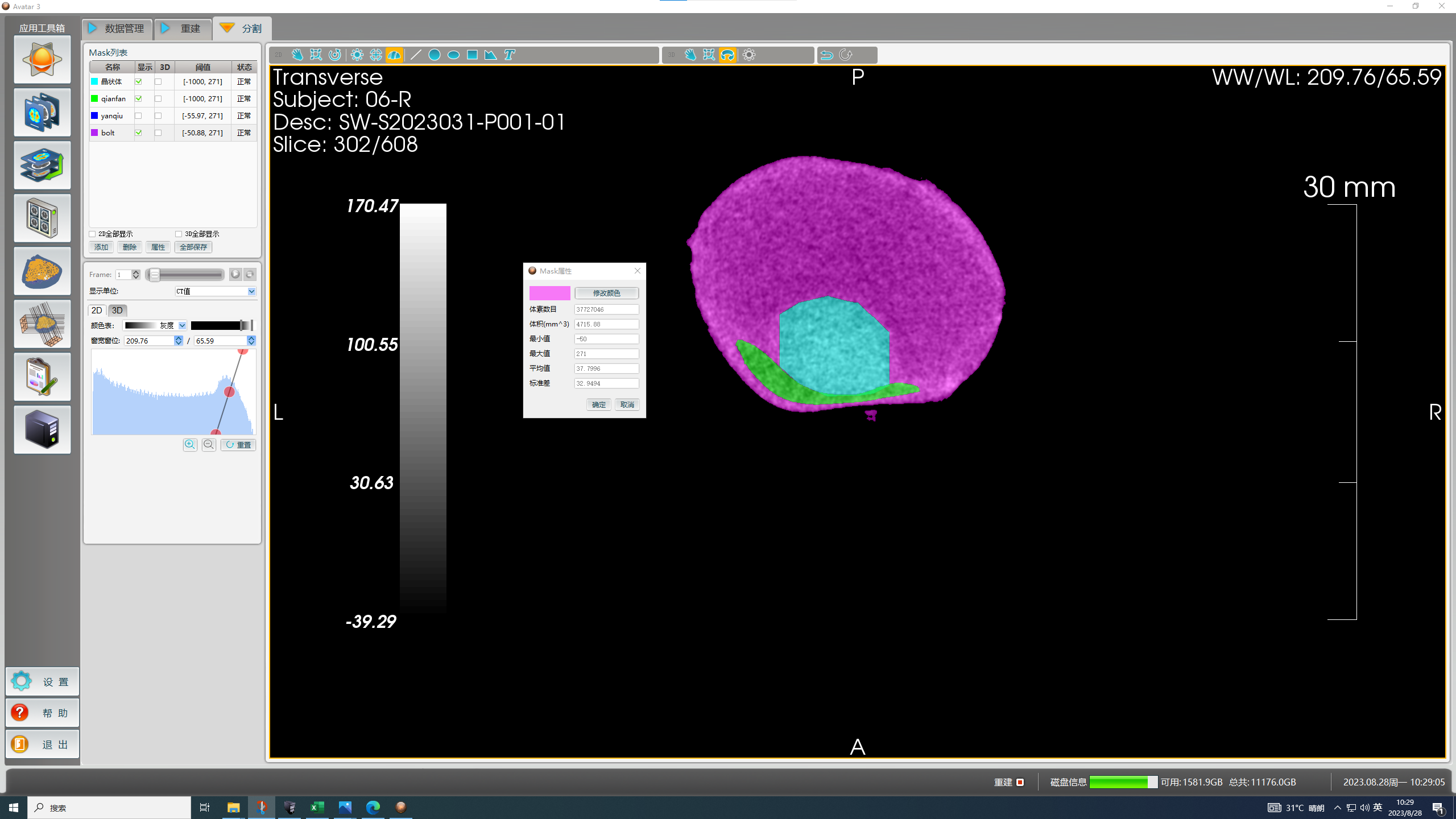

Supplement: S2 Data — (ZIP) [file pone.0310830.s002.zip › CT_pigs/Vitreous body/06-R.png]

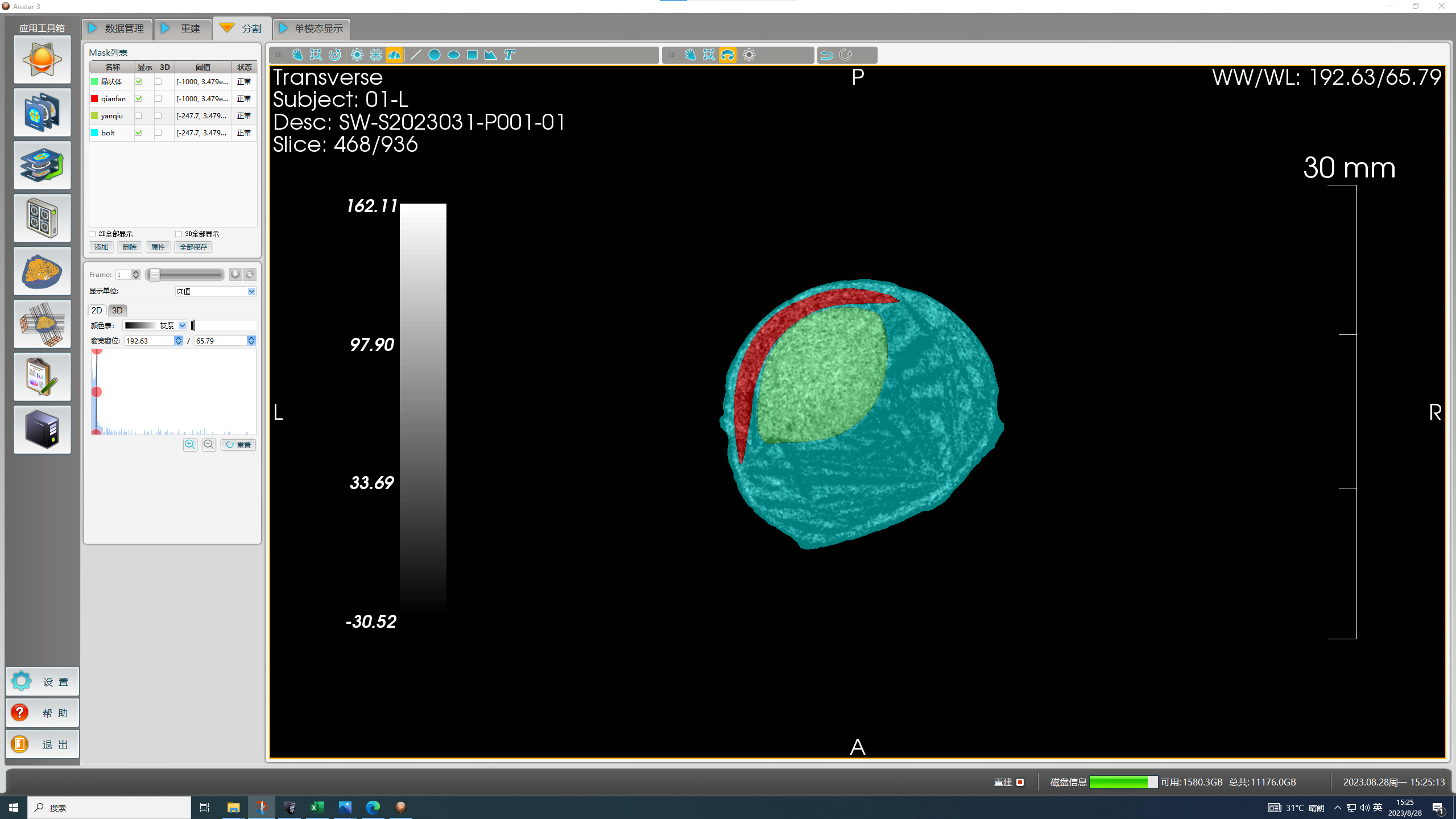

Supplement: S3 Data — (ZIP) [file pone.0310830.s003.zip › CT_rabbits/01-L.png]

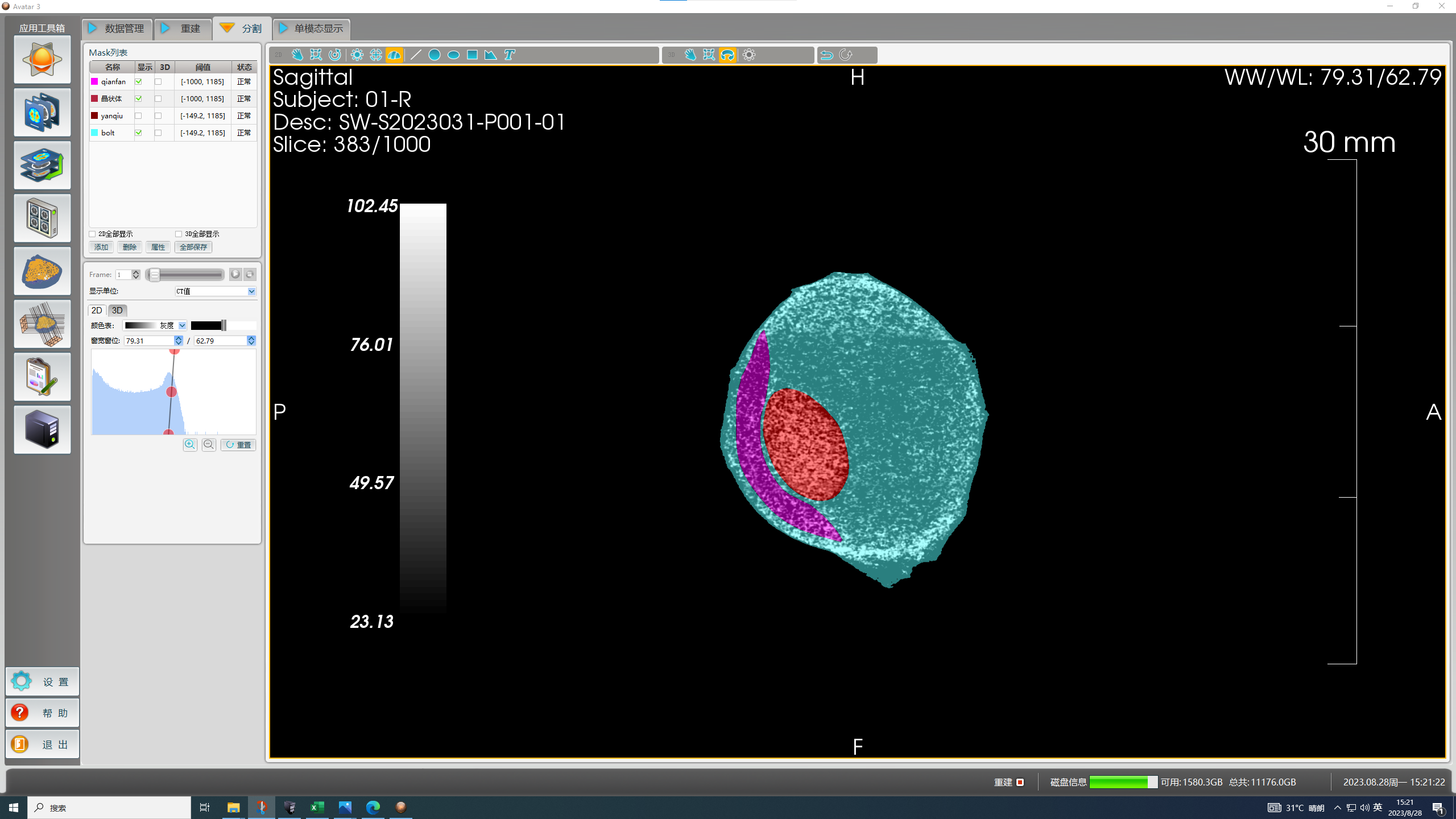

Supplement: S3 Data — (ZIP) [file pone.0310830.s003.zip › CT_rabbits/01-R.png]

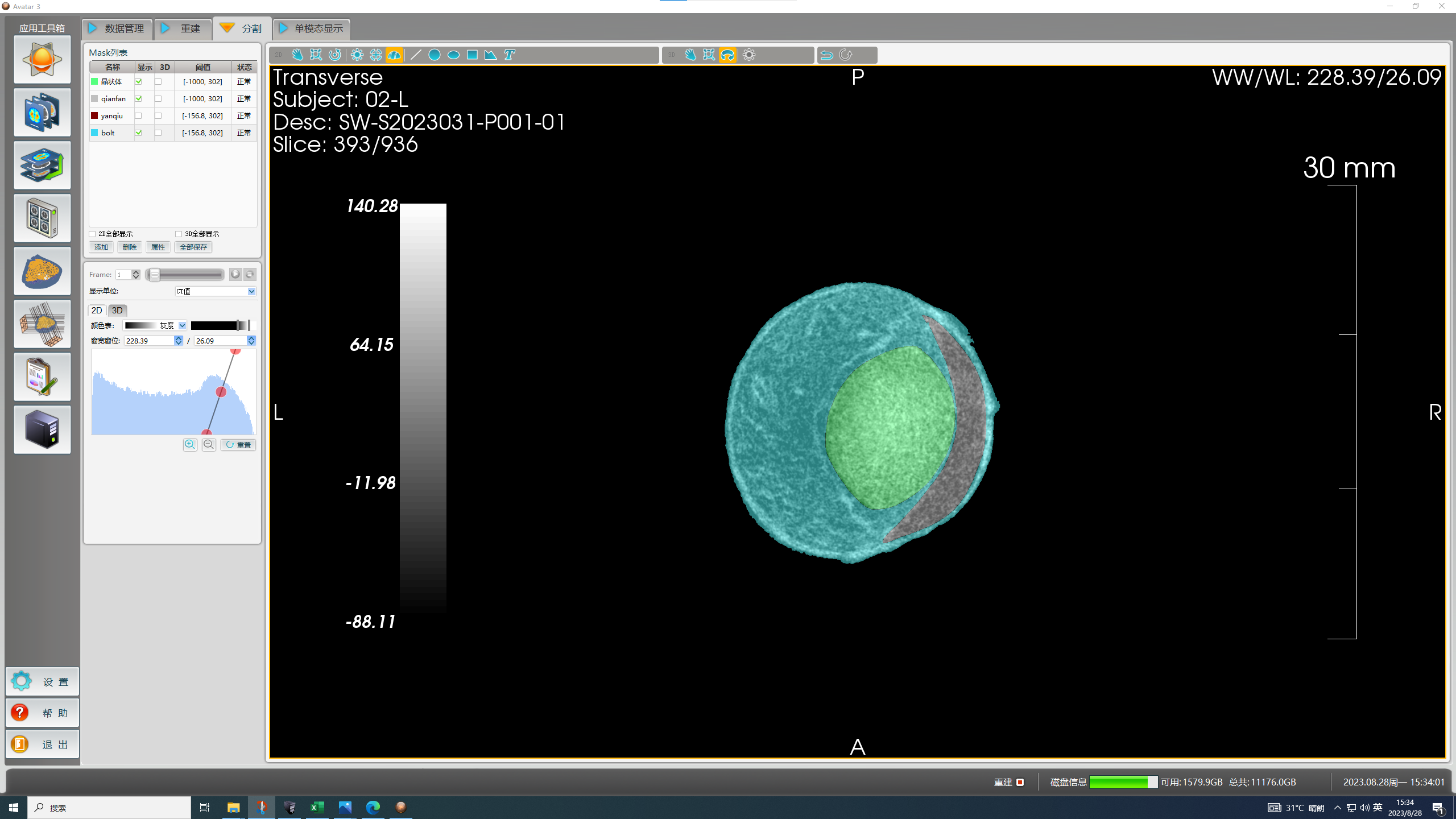

Supplement: S3 Data — (ZIP) [file pone.0310830.s003.zip › CT_rabbits/02-L.png]

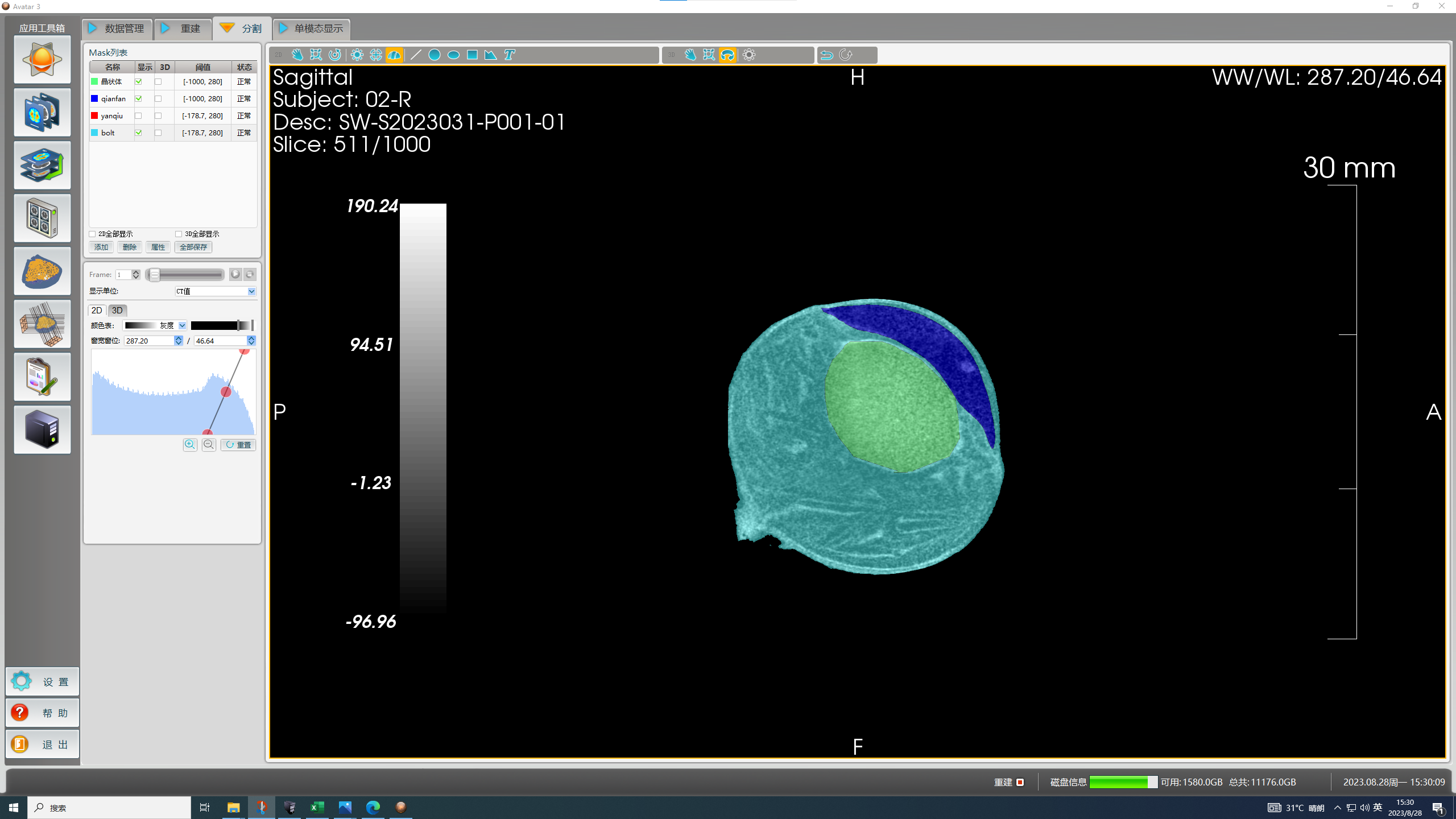

Supplement: S3 Data — (ZIP) [file pone.0310830.s003.zip › CT_rabbits/02-R.png]

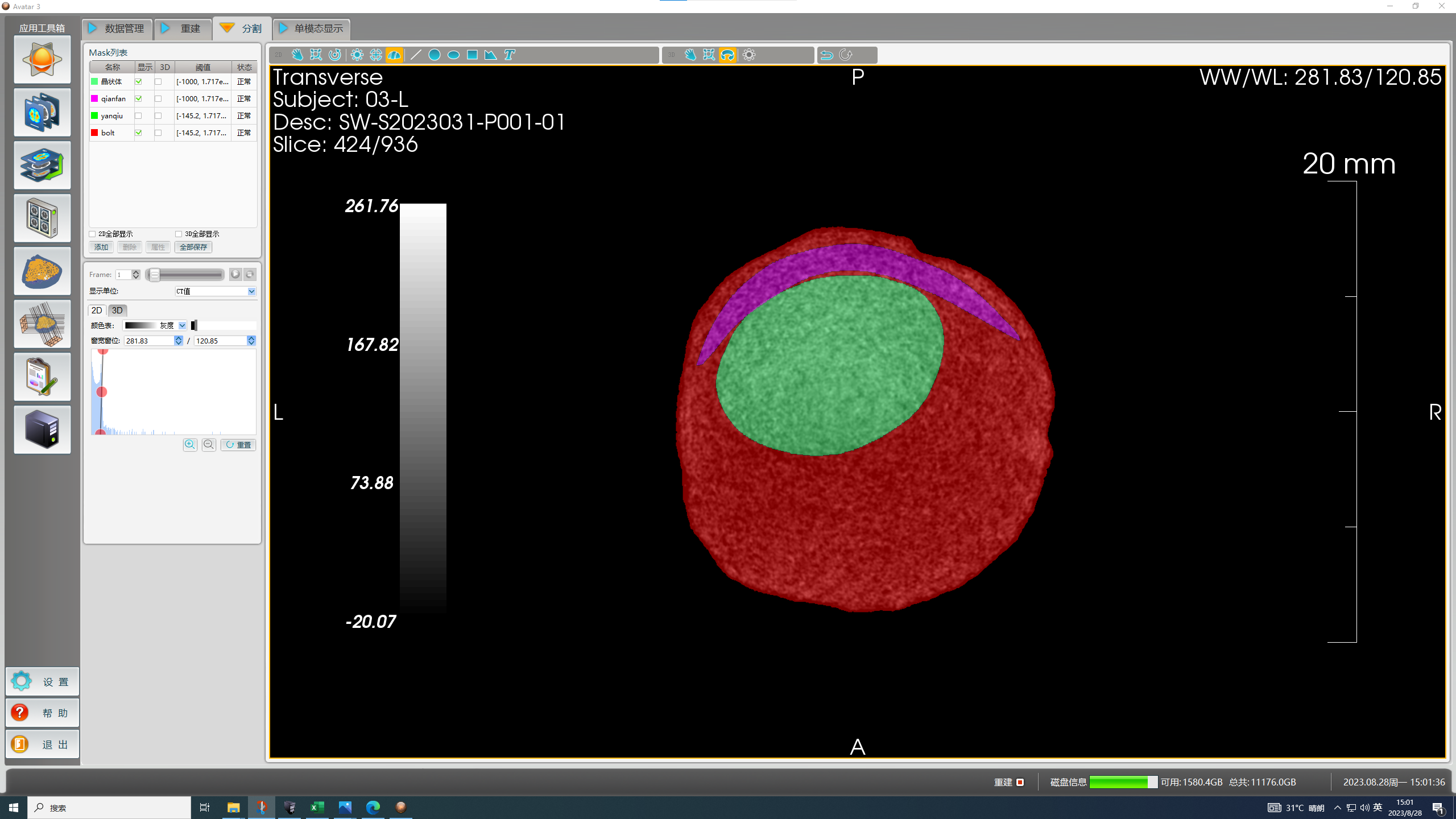

Supplement: S3 Data — (ZIP) [file pone.0310830.s003.zip › CT_rabbits/03-L.png]

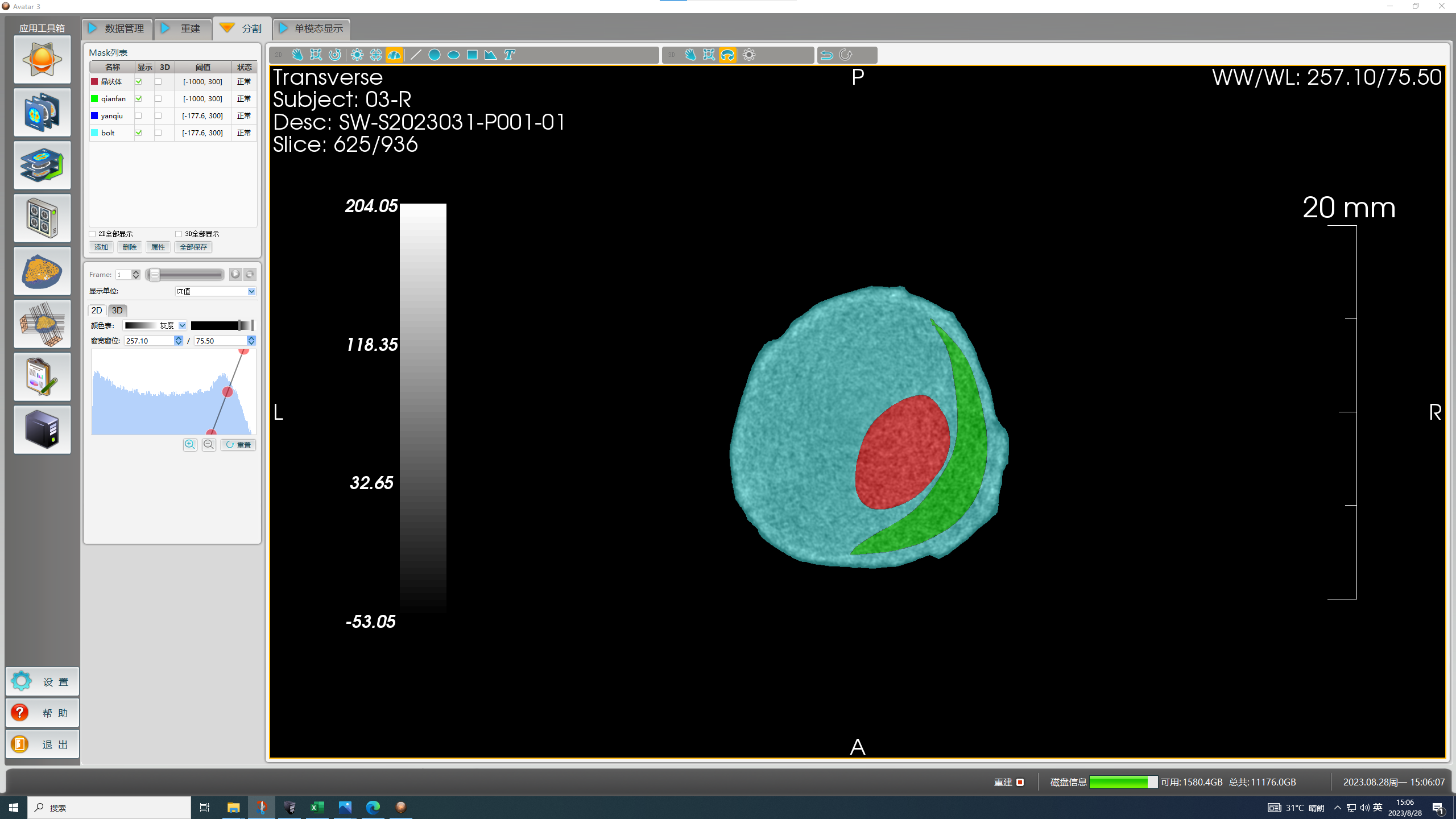

Supplement: S3 Data — (ZIP) [file pone.0310830.s003.zip › CT_rabbits/03-R.png]

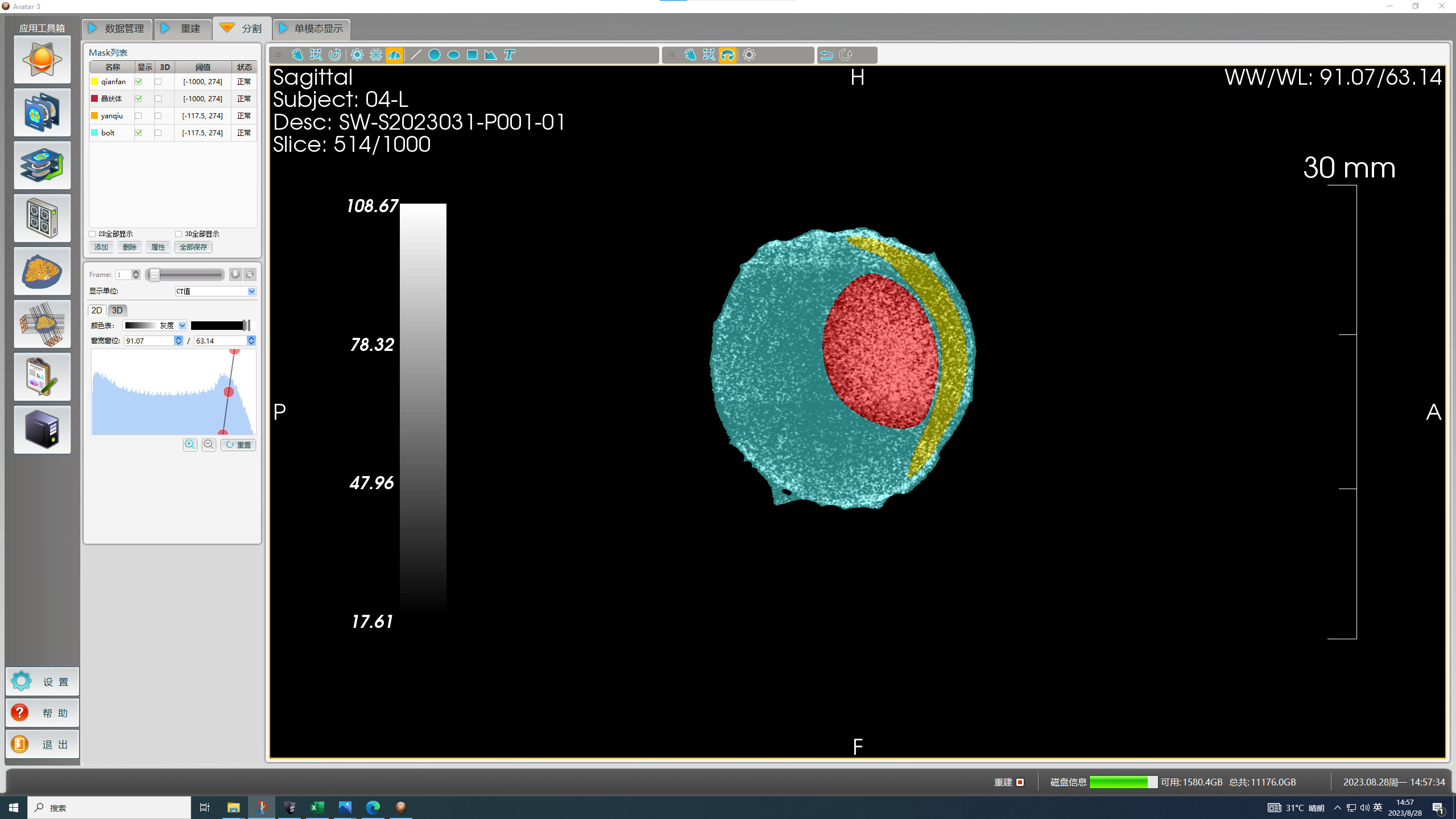

Supplement: S3 Data — (ZIP) [file pone.0310830.s003.zip › CT_rabbits/04-L.png]

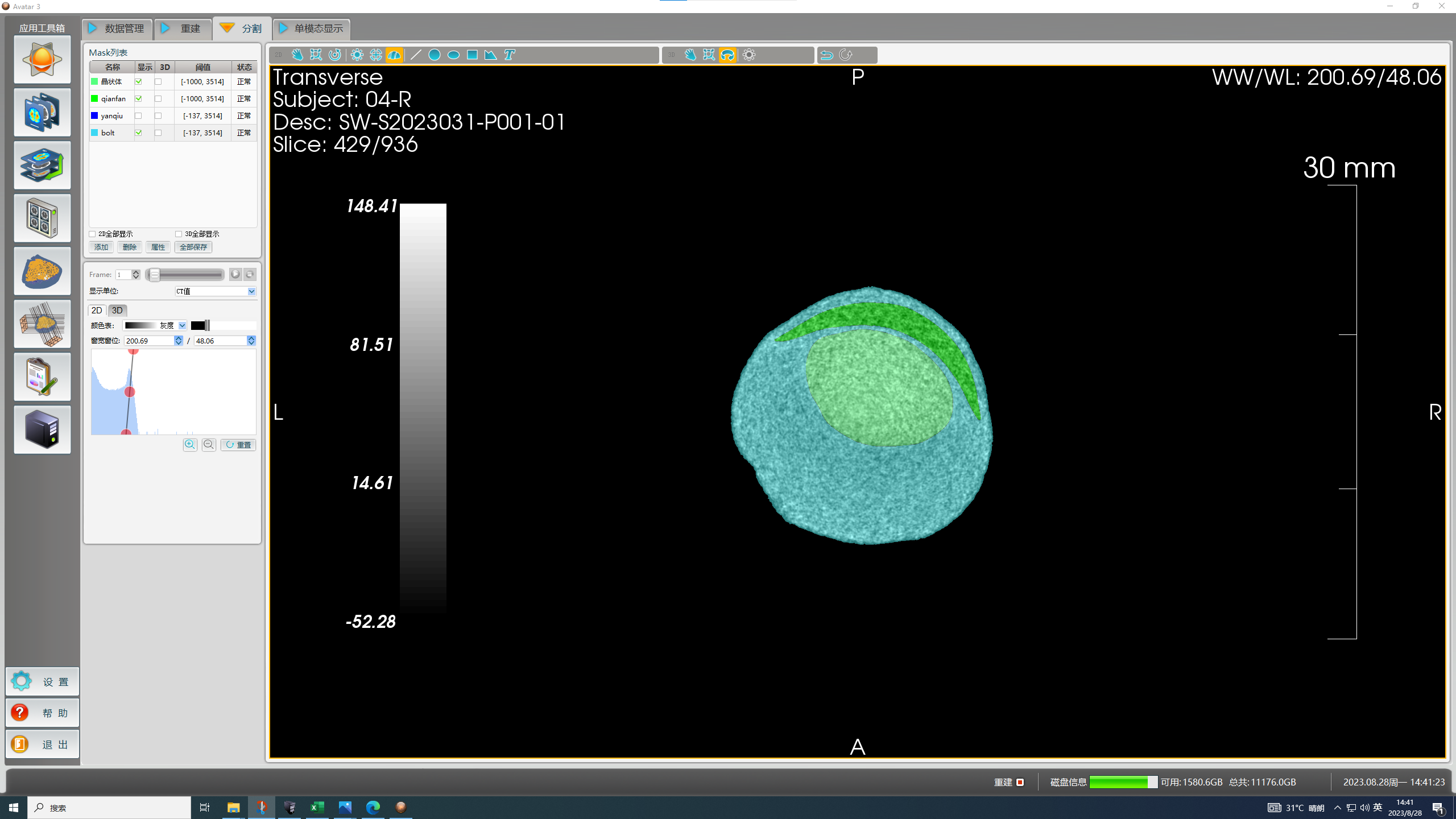

Supplement: S3 Data — (ZIP) [file pone.0310830.s003.zip › CT_rabbits/04-R.png]

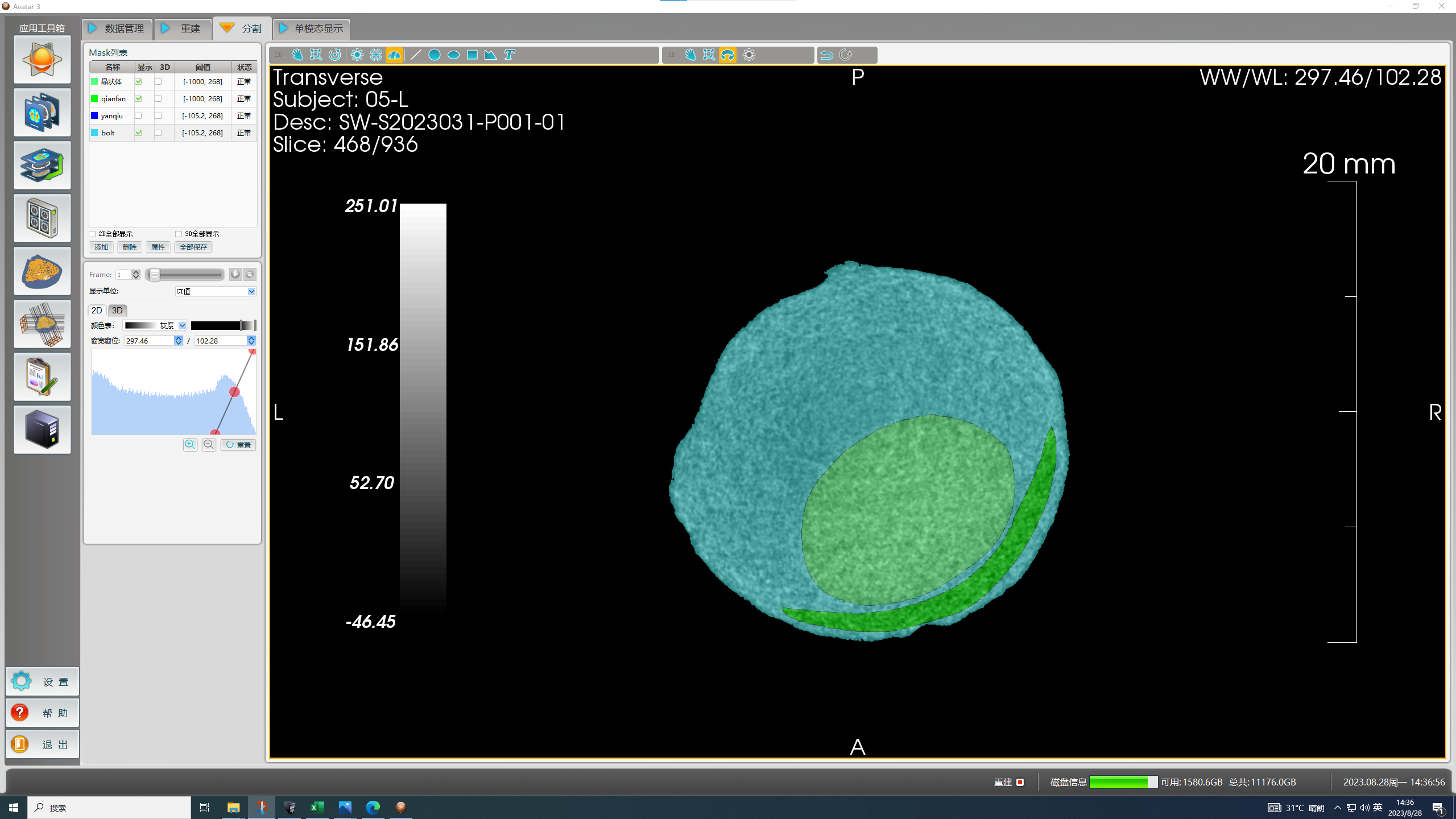

Supplement: S3 Data — (ZIP) [file pone.0310830.s003.zip › CT_rabbits/05-L.png]

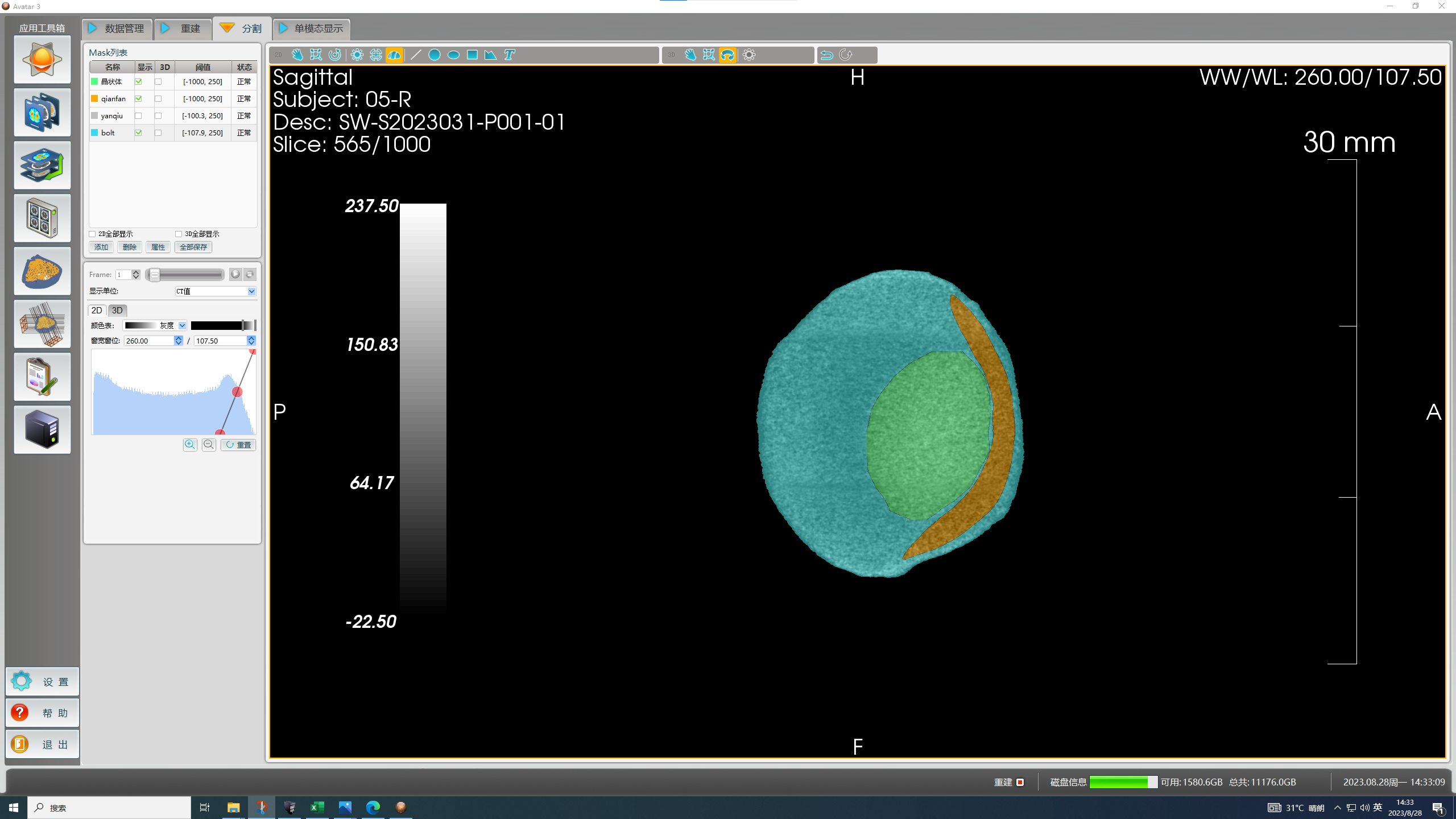

Supplement: S3 Data — (ZIP) [file pone.0310830.s003.zip › CT_rabbits/05-R.png]

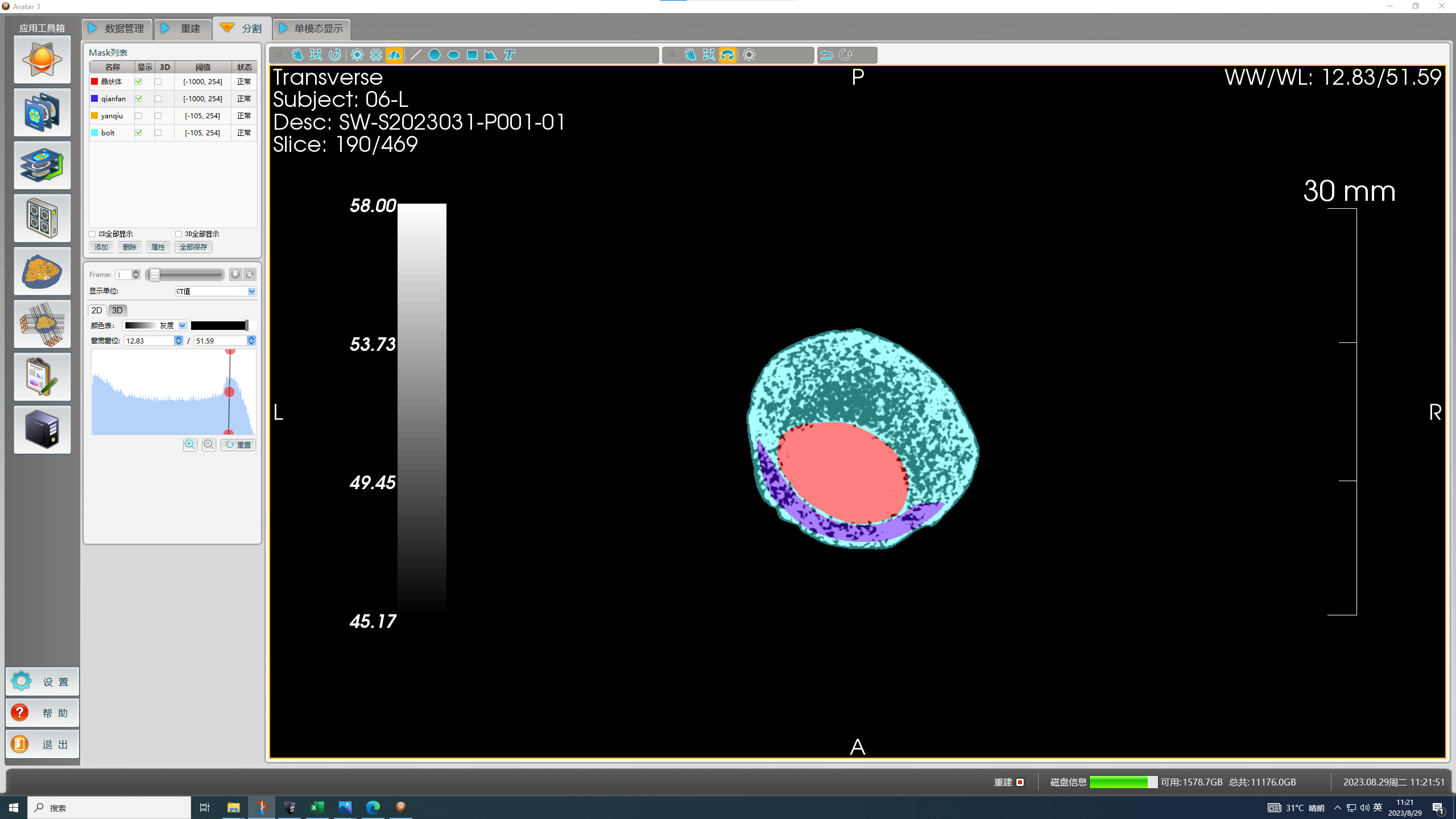

Supplement: S3 Data — (ZIP) [file pone.0310830.s003.zip › CT_rabbits/06-L.png]

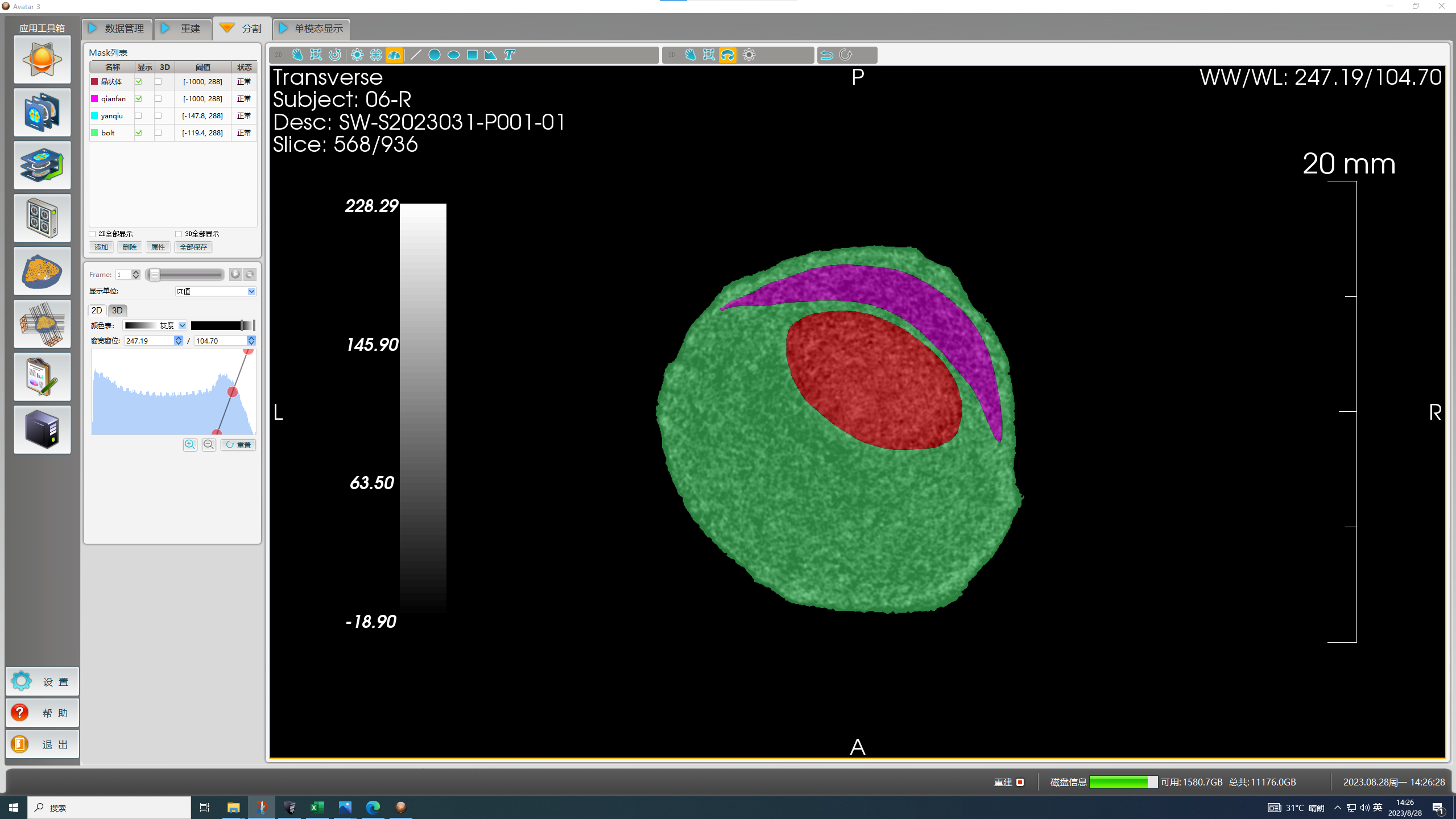

Supplement: S3 Data — (ZIP) [file pone.0310830.s003.zip › CT_rabbits/06-R.png]

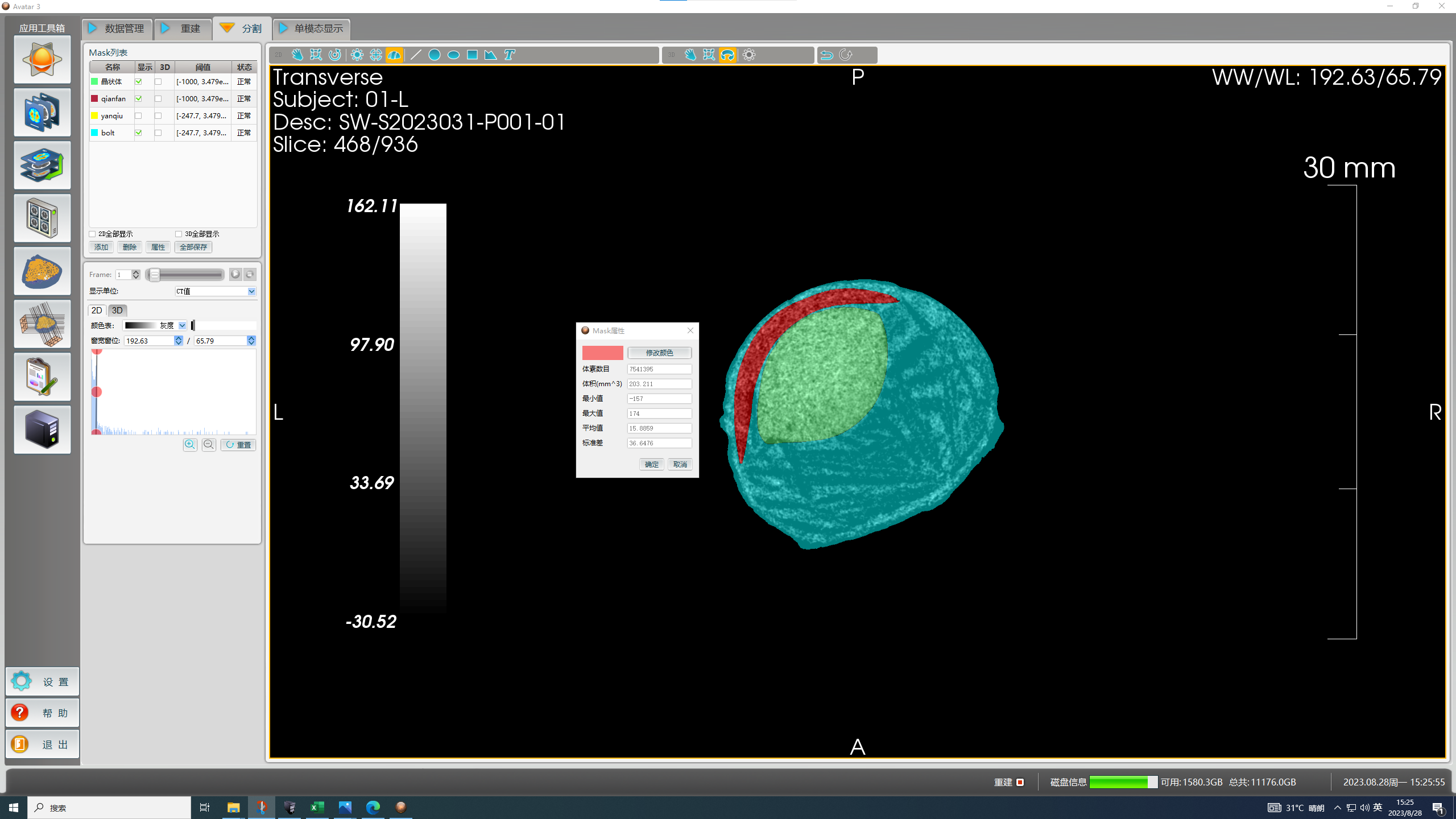

Supplement: S3 Data — (ZIP) [file pone.0310830.s003.zip › CT_rabbits/Anterior chamber/01-L.png]

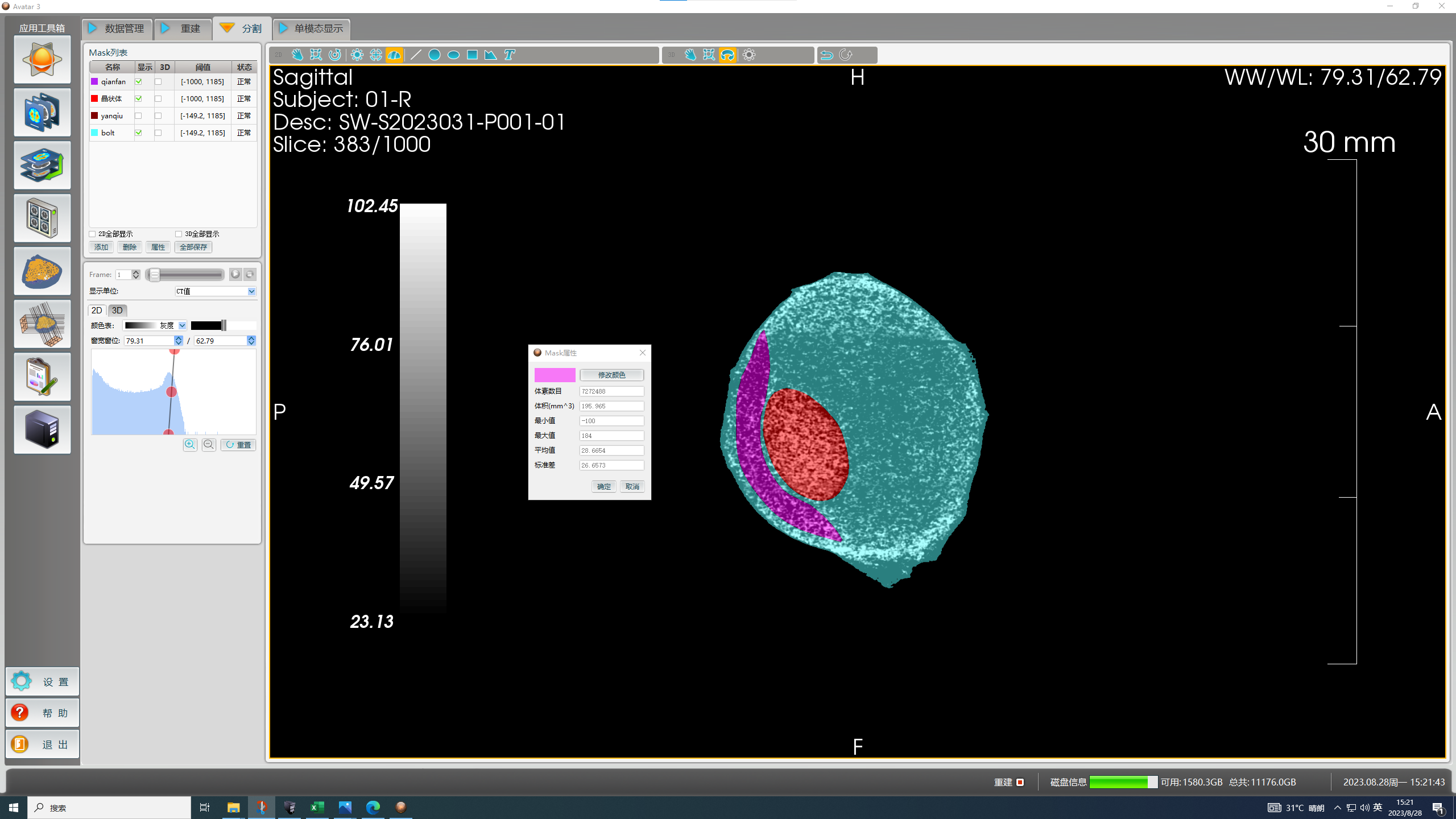

Supplement: S3 Data — (ZIP) [file pone.0310830.s003.zip › CT_rabbits/Anterior chamber/01-R.png]

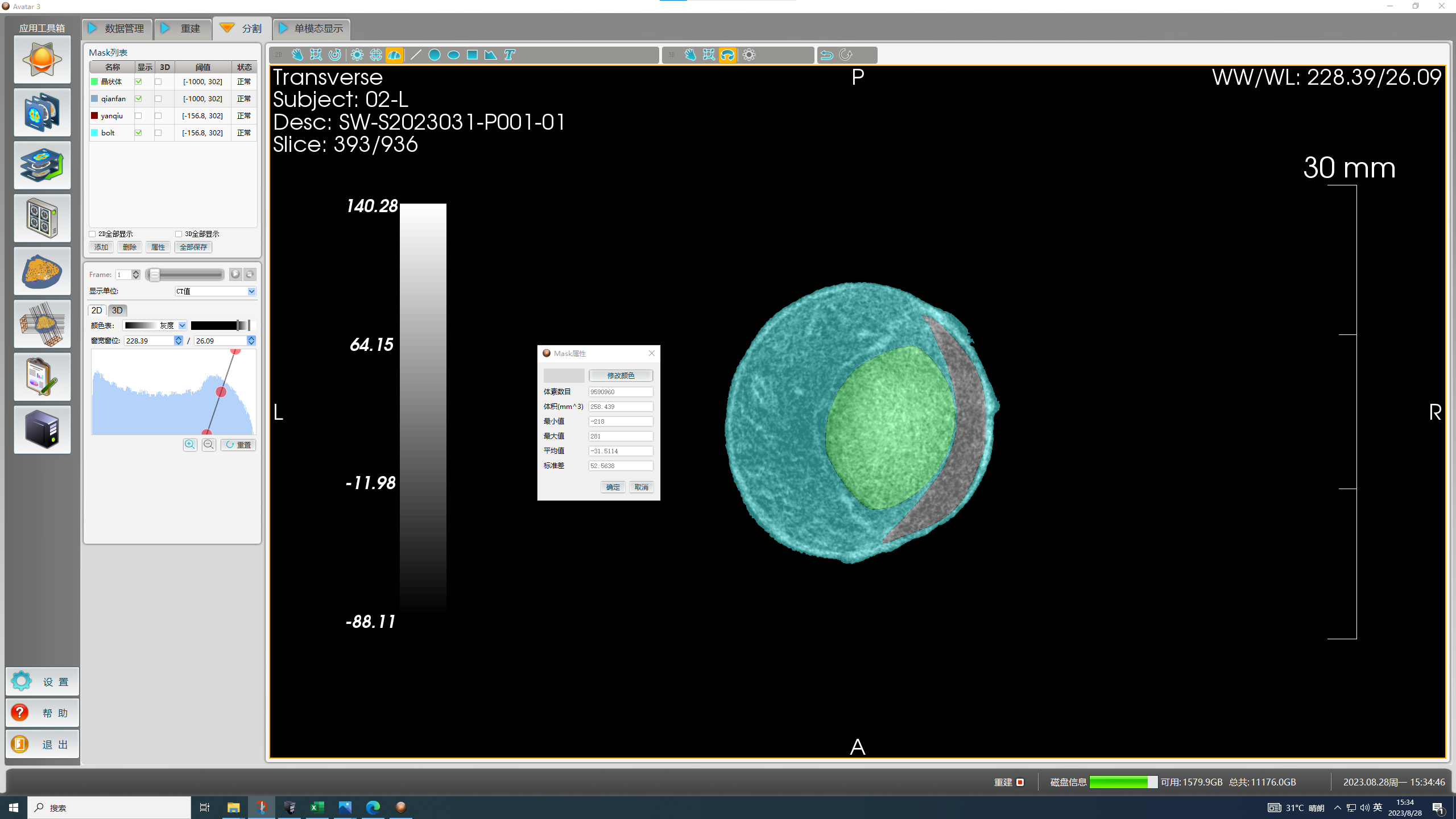

Supplement: S3 Data — (ZIP) [file pone.0310830.s003.zip › CT_rabbits/Anterior chamber/02-L.png]

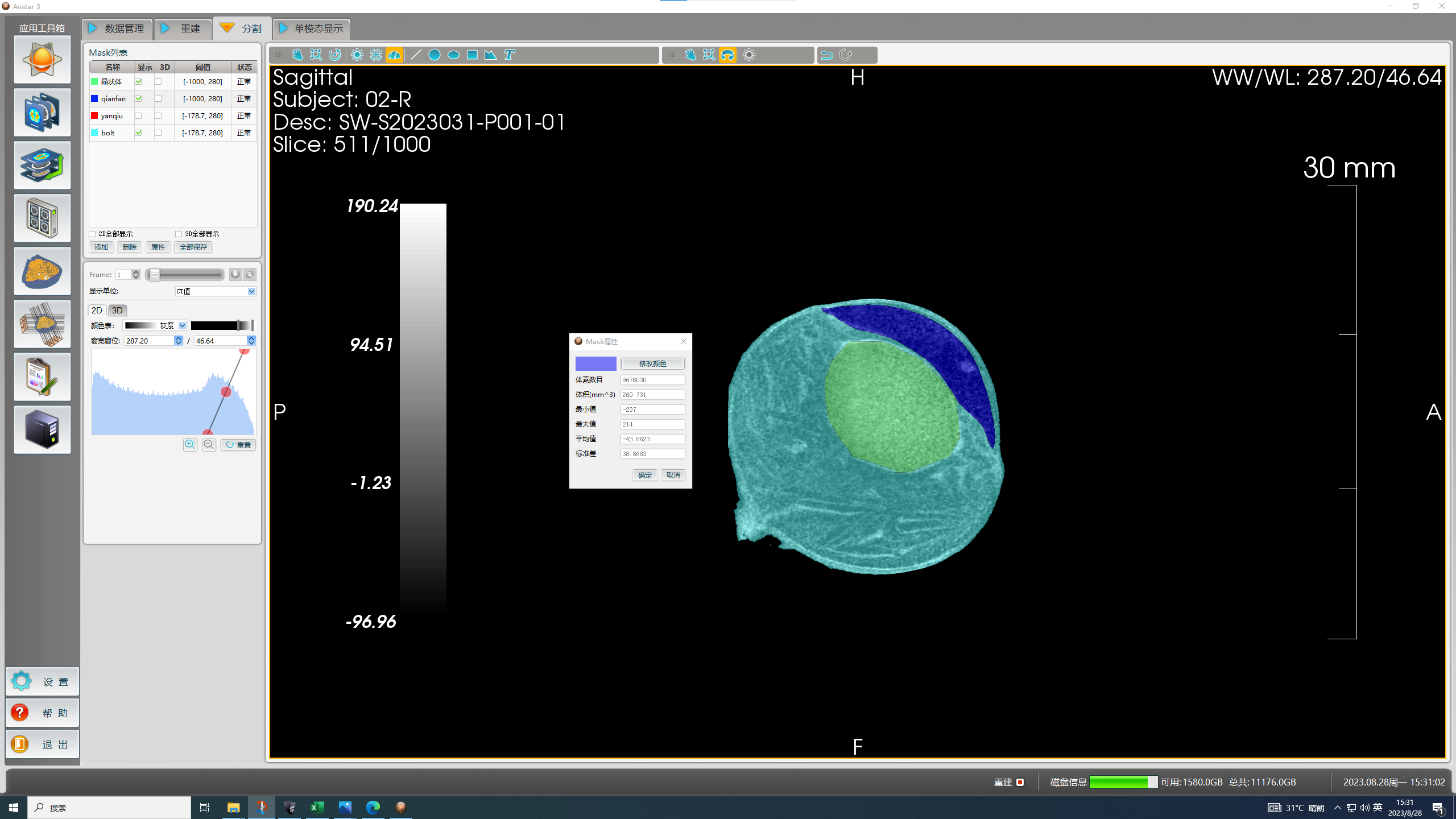

Supplement: S3 Data — (ZIP) [file pone.0310830.s003.zip › CT_rabbits/Anterior chamber/02-R.png]

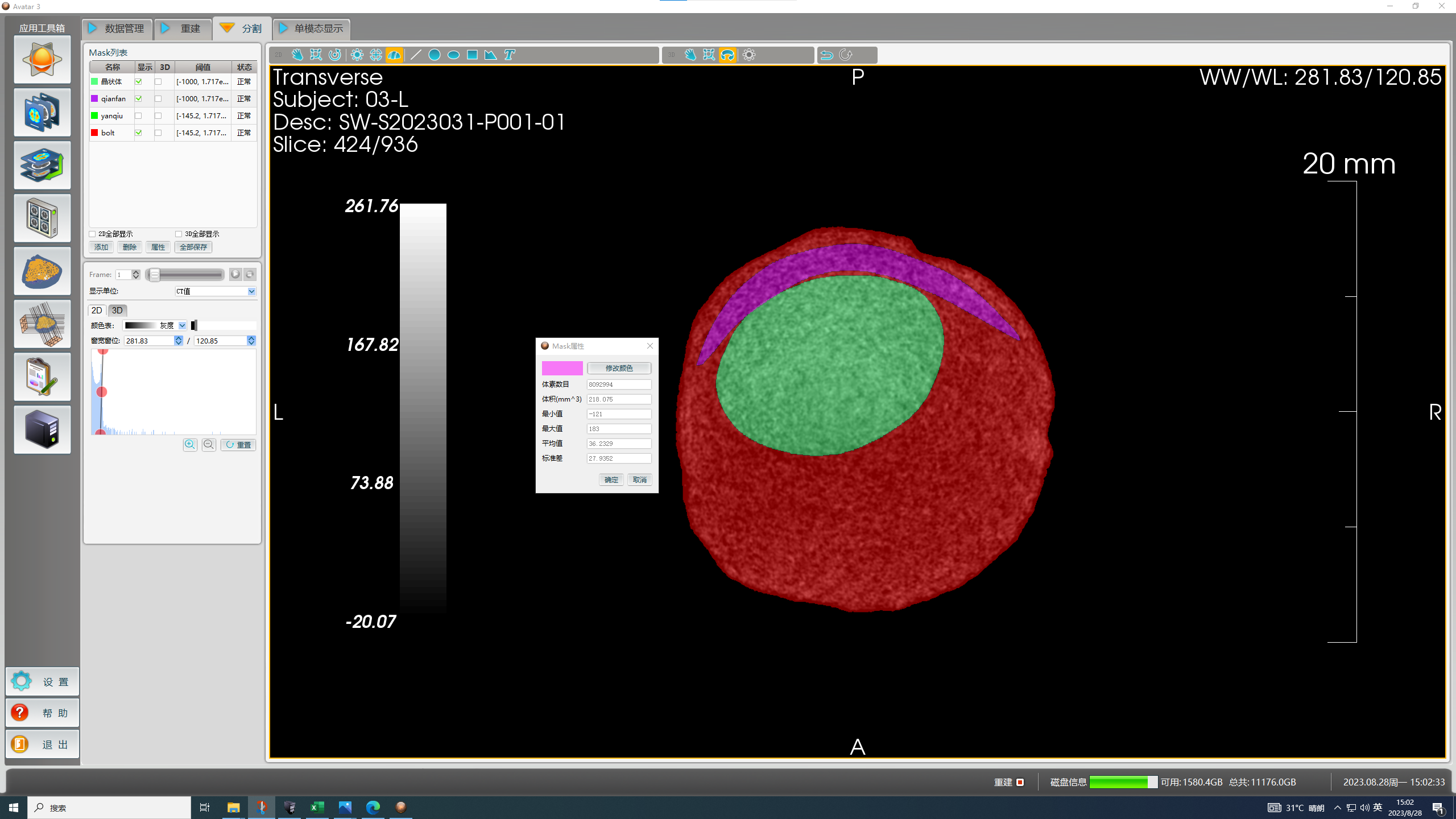

Supplement: S3 Data — (ZIP) [file pone.0310830.s003.zip › CT_rabbits/Anterior chamber/03-L.png]

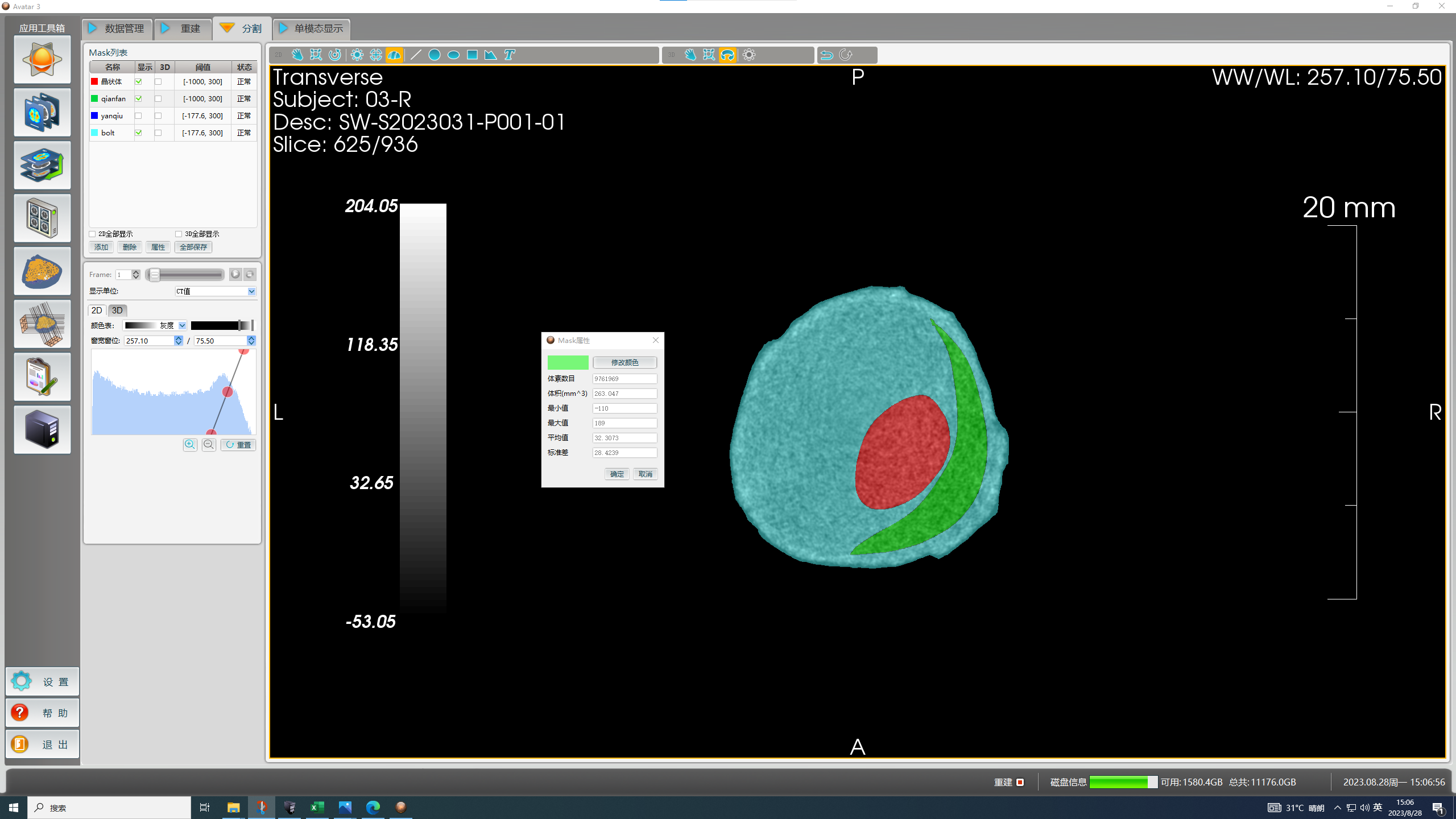

Supplement: S3 Data — (ZIP) [file pone.0310830.s003.zip › CT_rabbits/Anterior chamber/03-R.png]

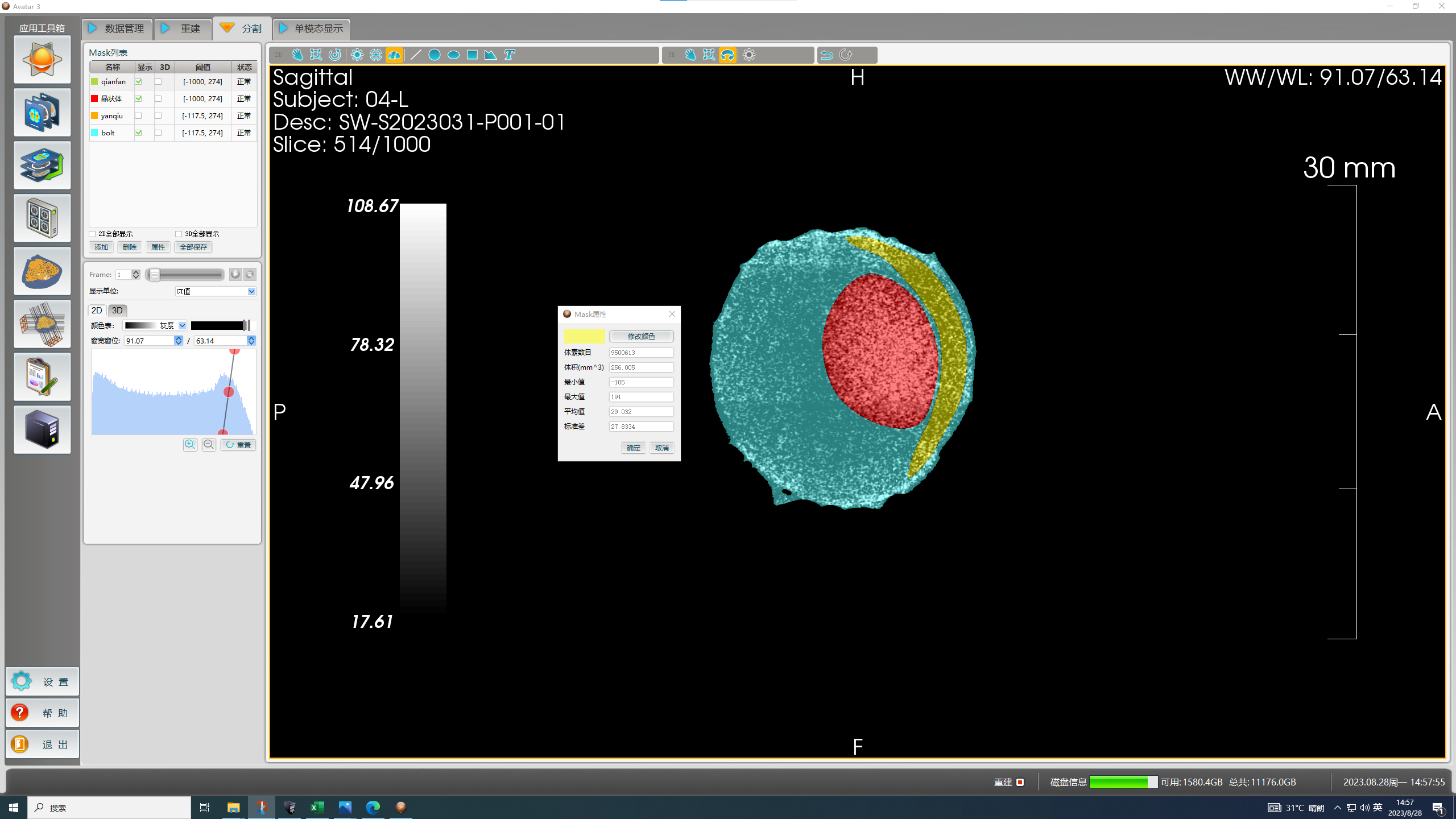

Supplement: S3 Data — (ZIP) [file pone.0310830.s003.zip › CT_rabbits/Anterior chamber/04-L.png]

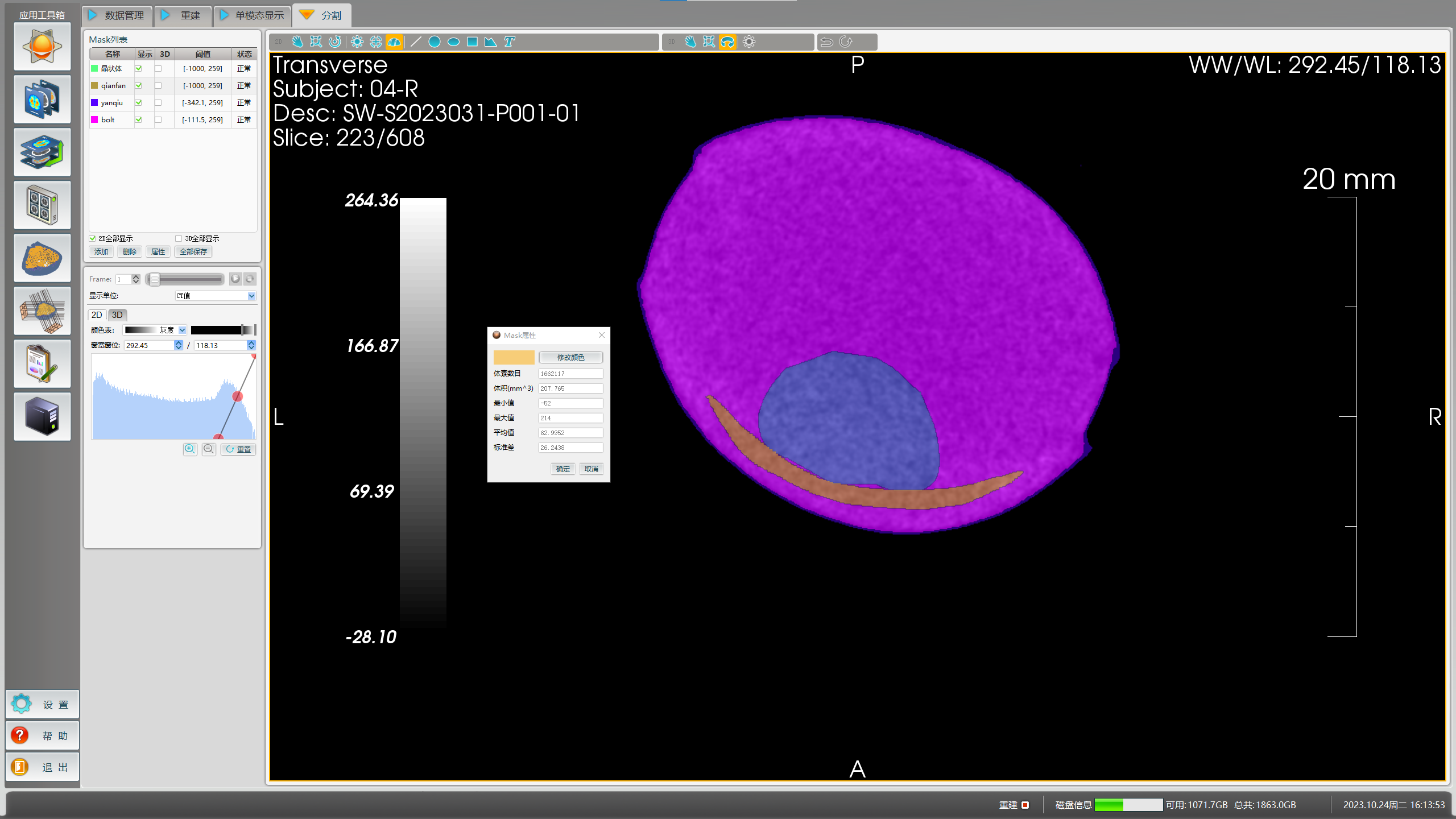

Supplement: S3 Data — (ZIP) [file pone.0310830.s003.zip › CT_rabbits/Anterior chamber/04-R2.png]

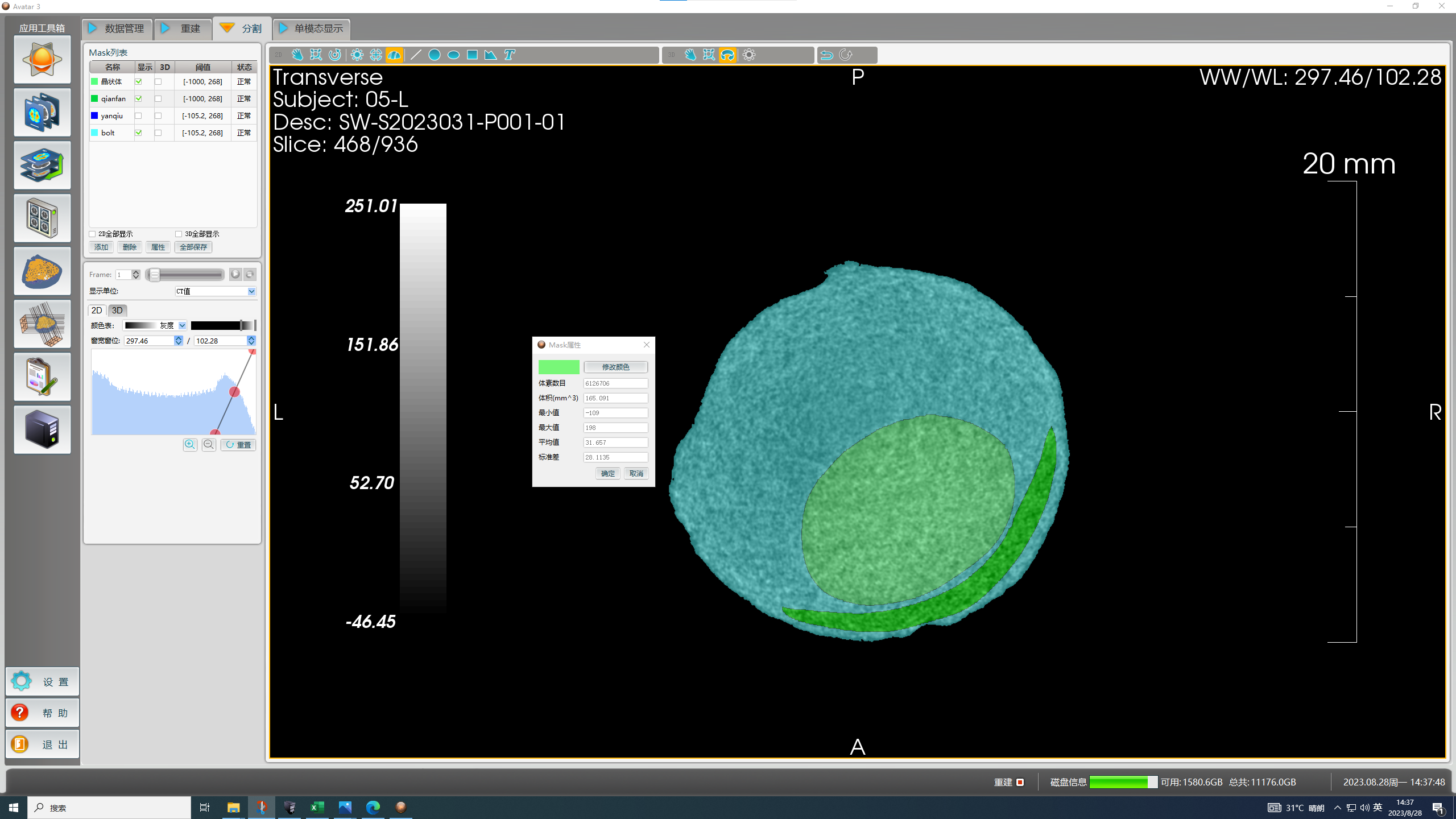

Supplement: S3 Data — (ZIP) [file pone.0310830.s003.zip › CT_rabbits/Anterior chamber/05-L.png]

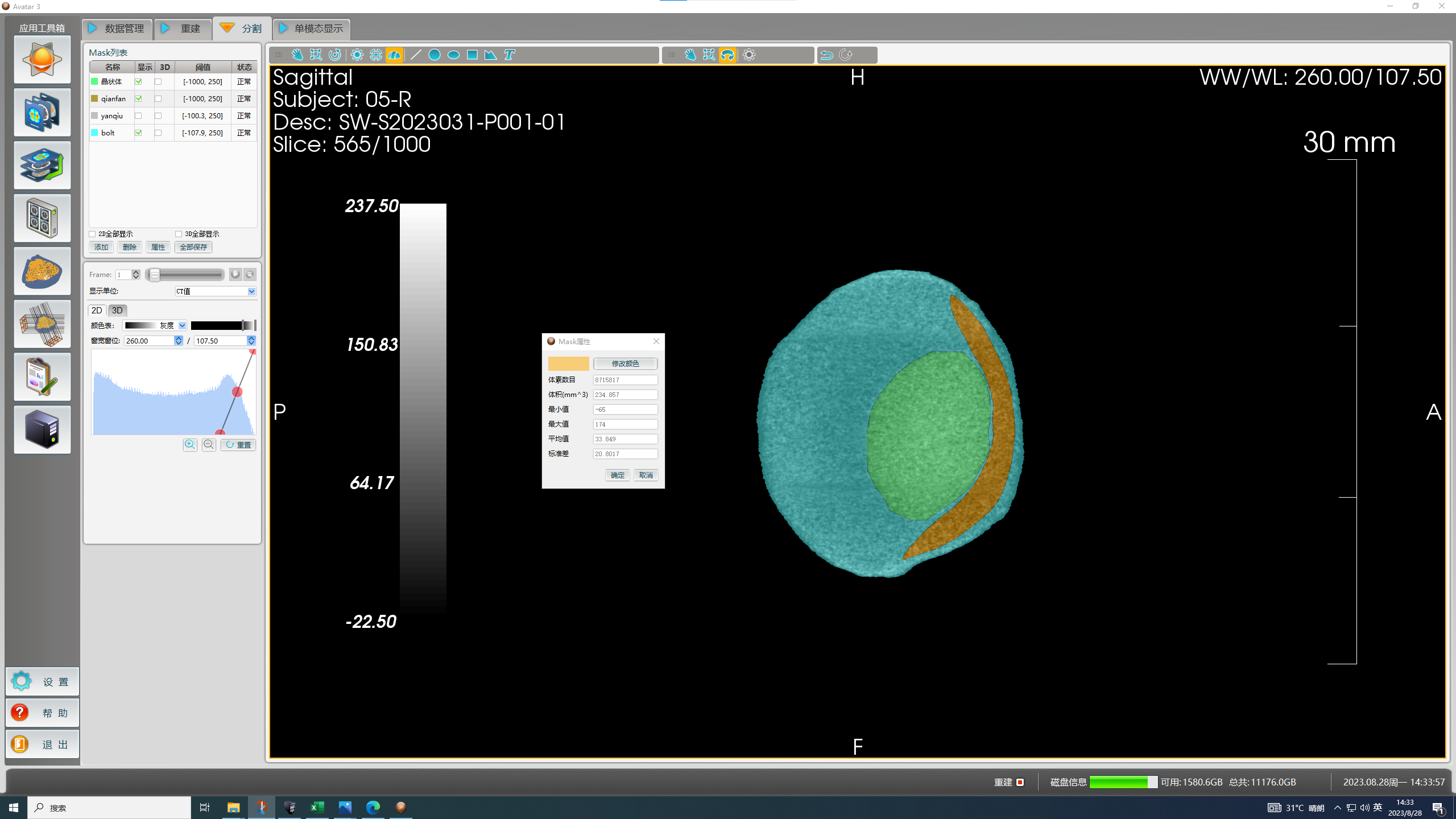

Supplement: S3 Data — (ZIP) [file pone.0310830.s003.zip › CT_rabbits/Anterior chamber/05-R.png]

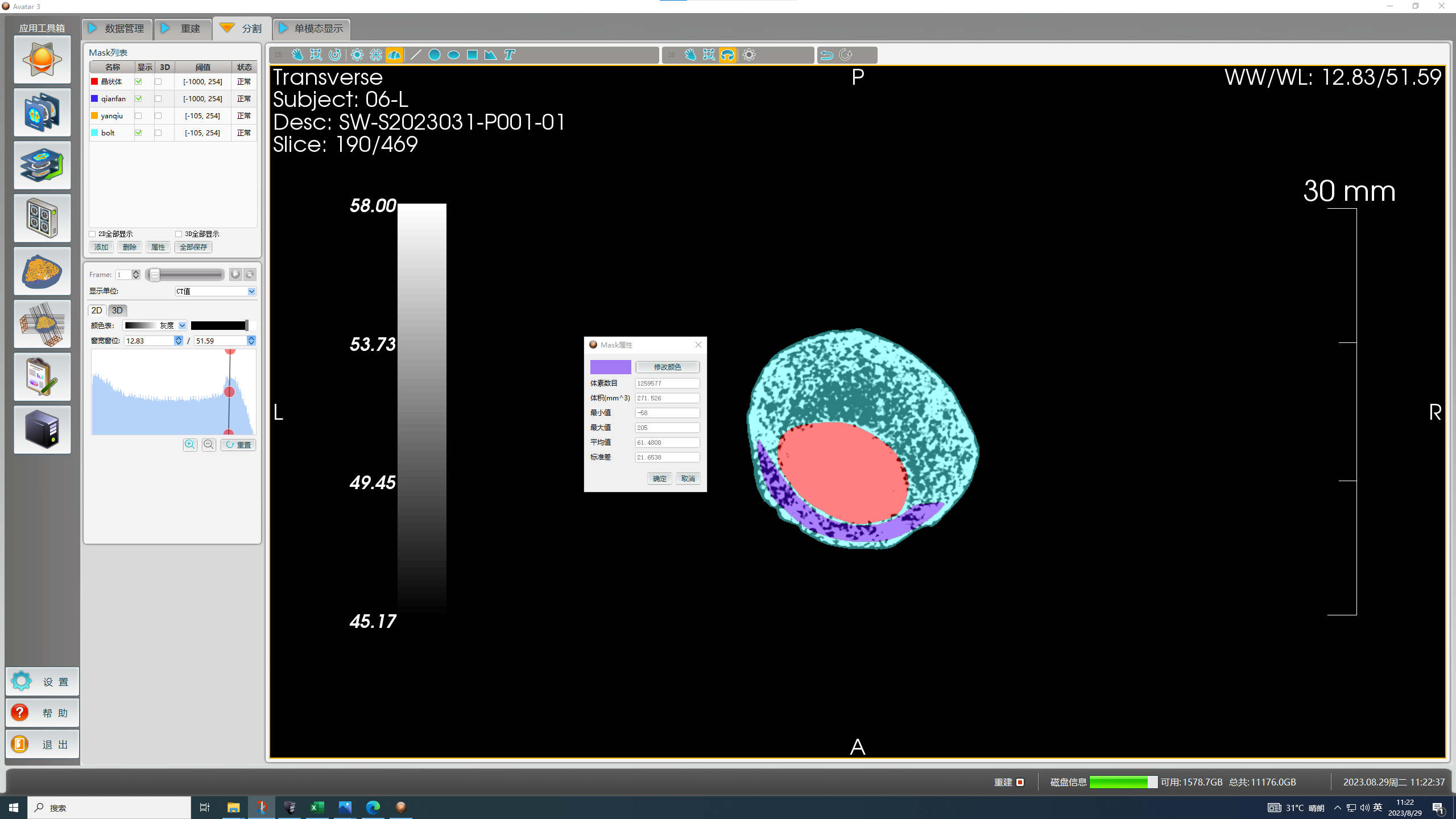

Supplement: S3 Data — (ZIP) [file pone.0310830.s003.zip › CT_rabbits/Anterior chamber/06-L.png]

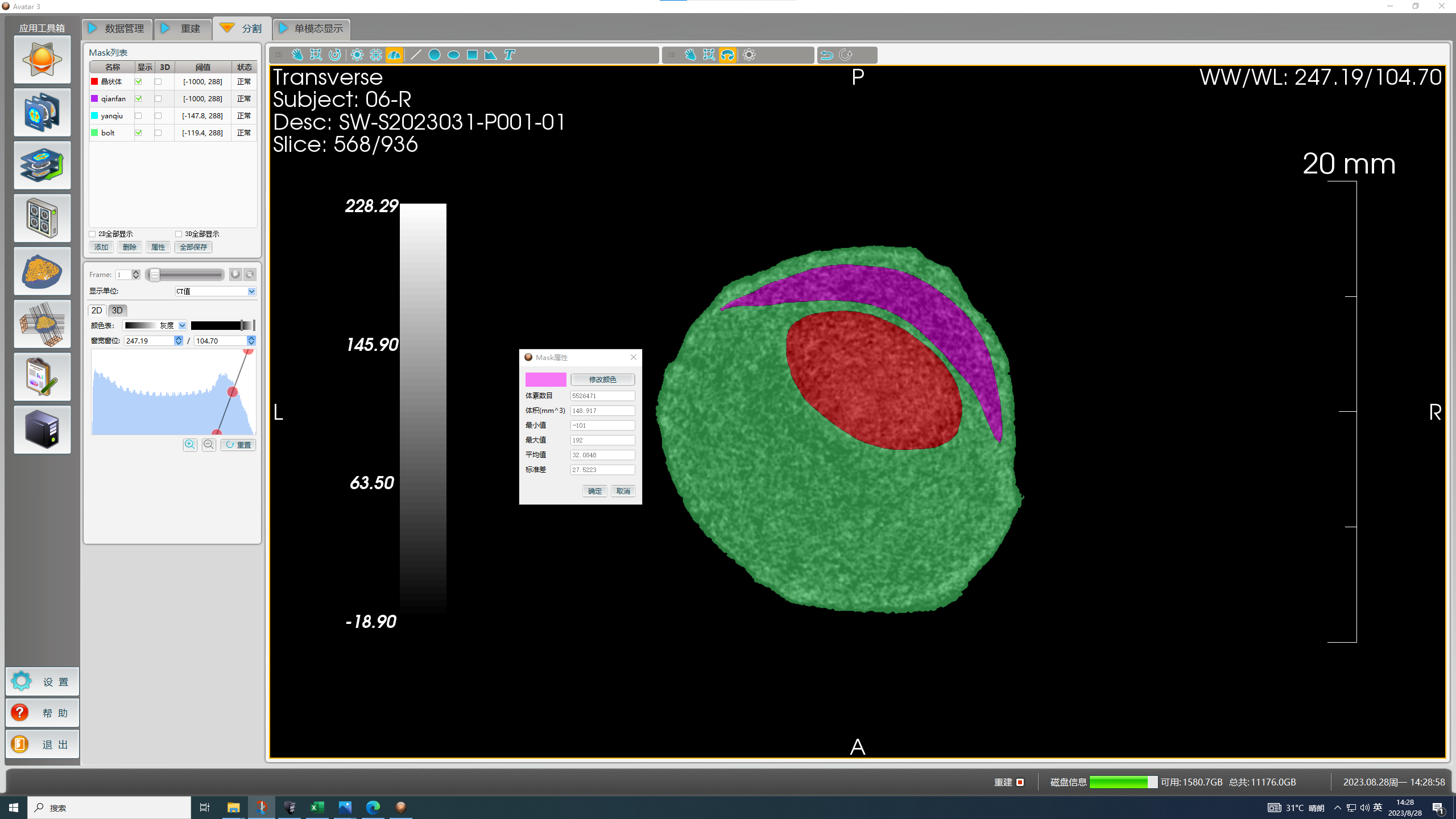

Supplement: S3 Data — (ZIP) [file pone.0310830.s003.zip › CT_rabbits/Anterior chamber/06-R.png]

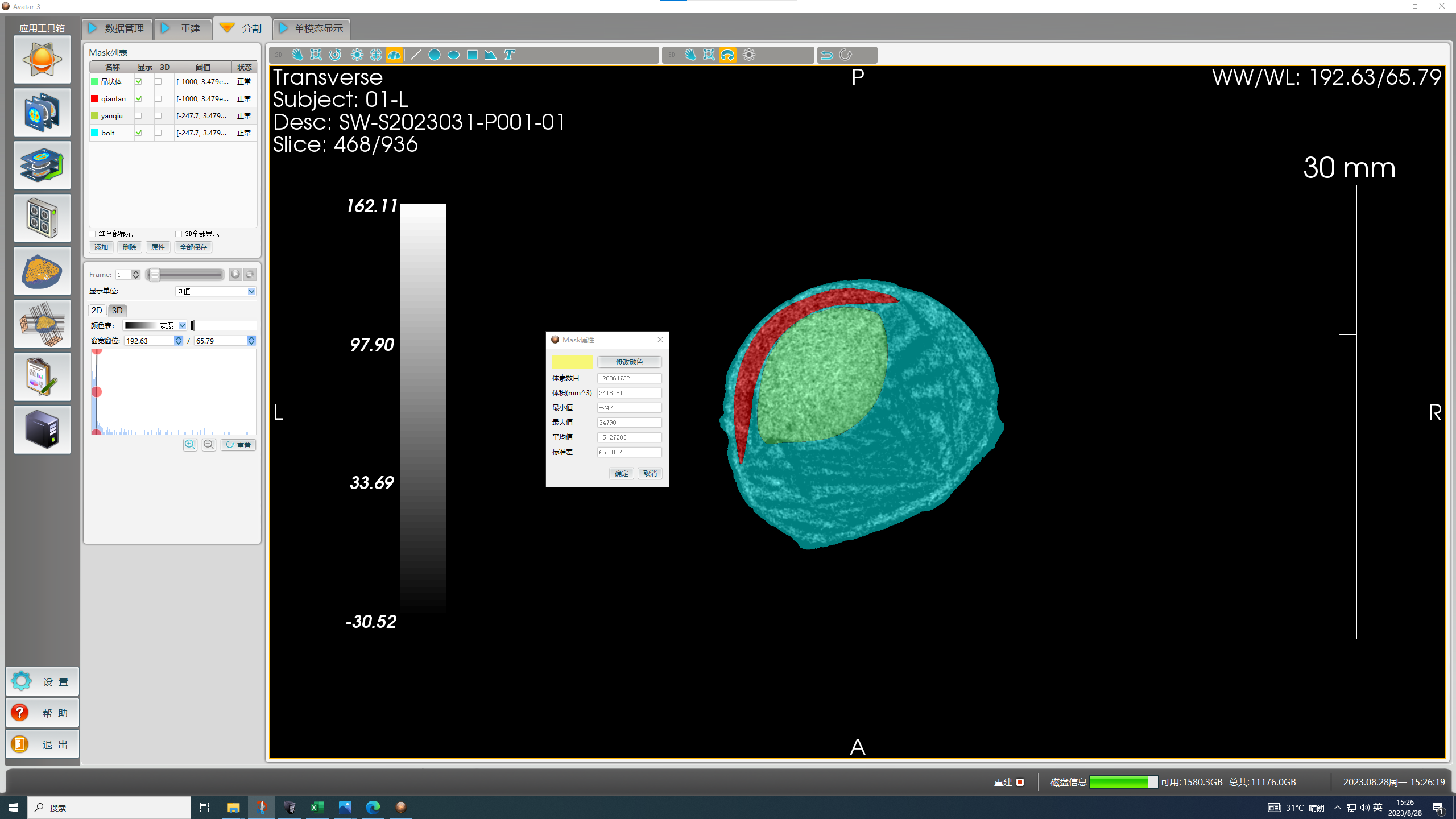

Supplement: S3 Data — (ZIP) [file pone.0310830.s003.zip › CT_rabbits/Eyeball volume/01-L.png]

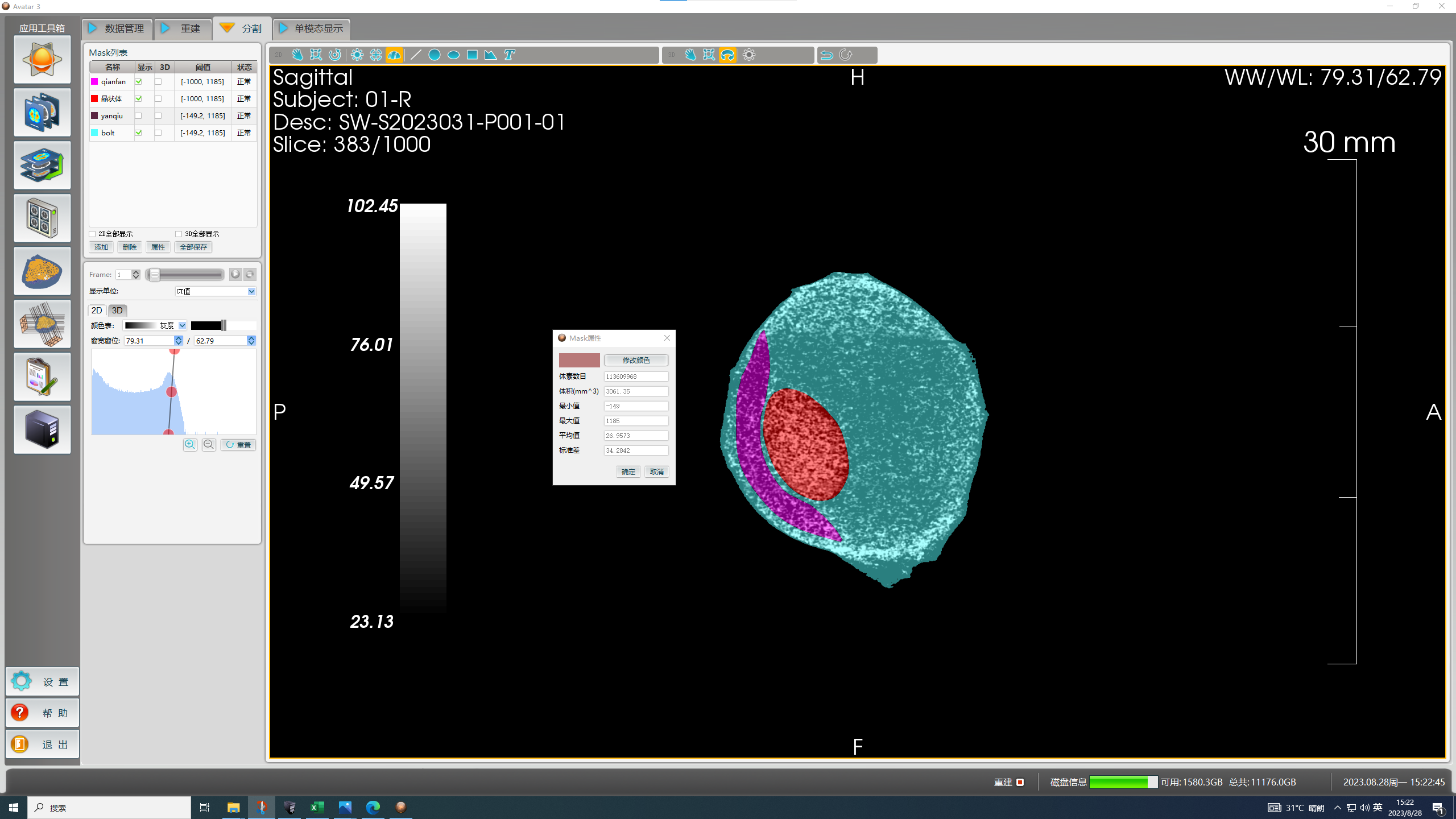

Supplement: S3 Data — (ZIP) [file pone.0310830.s003.zip › CT_rabbits/Eyeball volume/01-R.png]

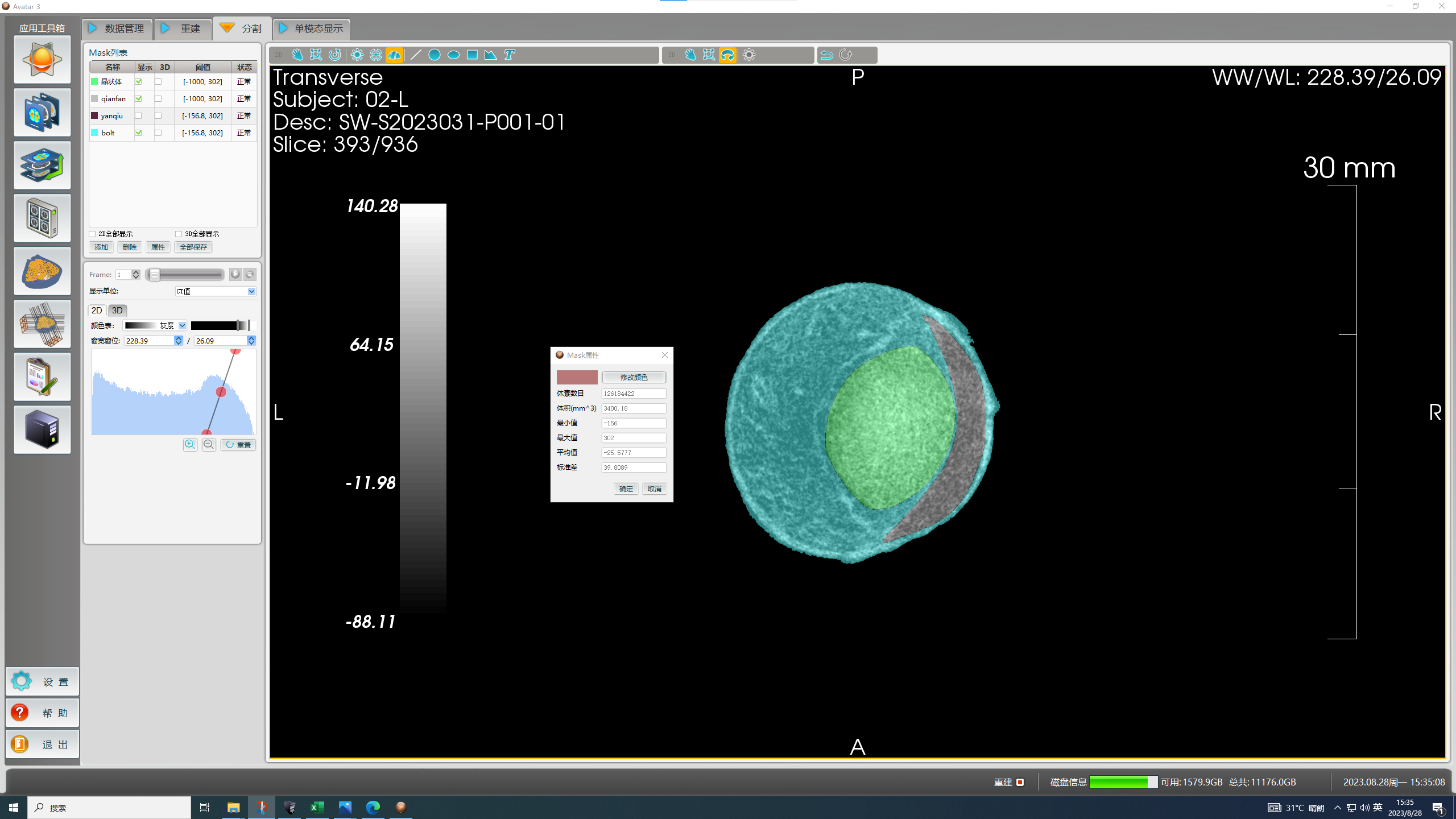

Supplement: S3 Data — (ZIP) [file pone.0310830.s003.zip › CT_rabbits/Eyeball volume/02-L.png]

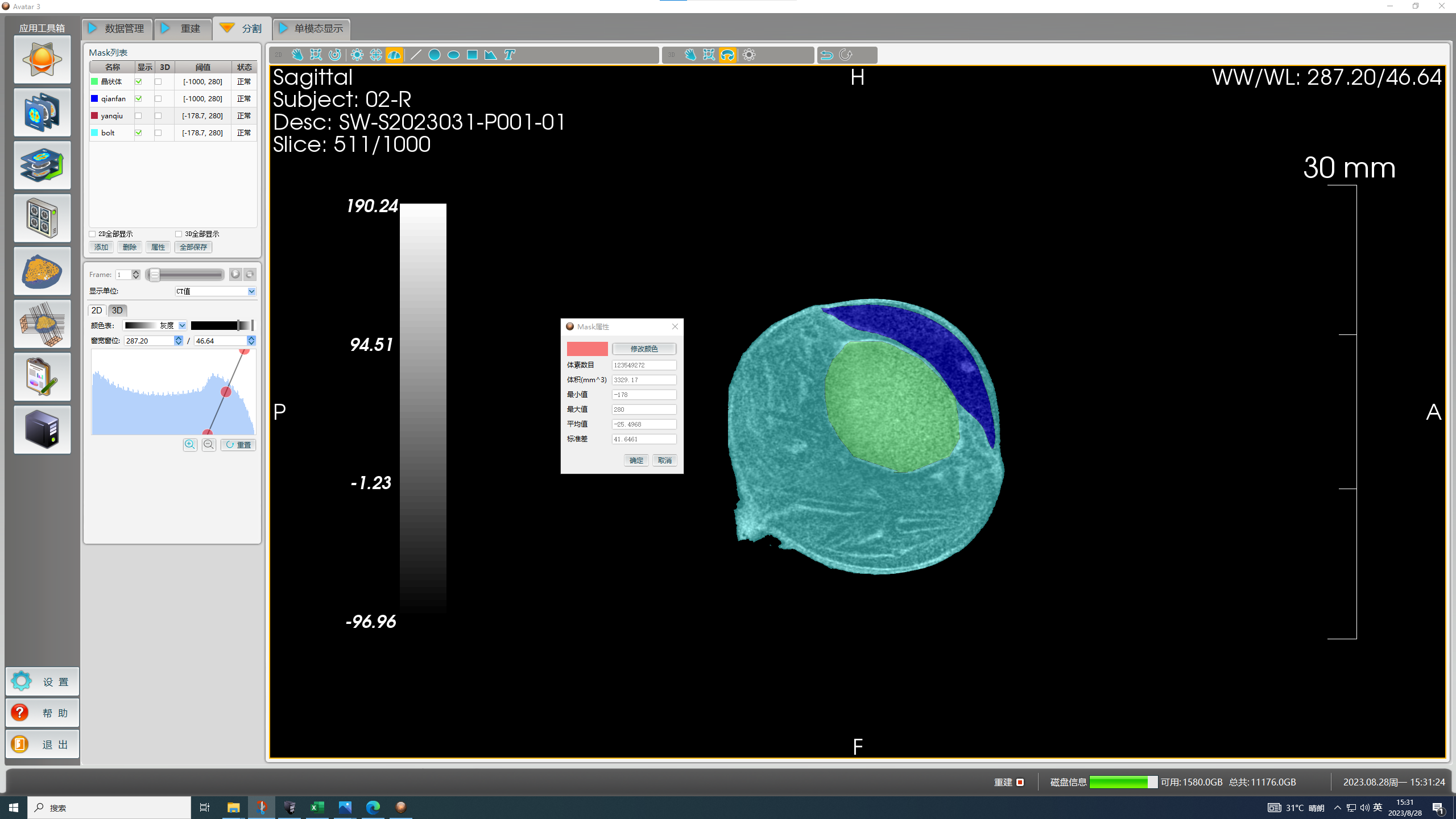

Supplement: S3 Data — (ZIP) [file pone.0310830.s003.zip › CT_rabbits/Eyeball volume/02-R.png]

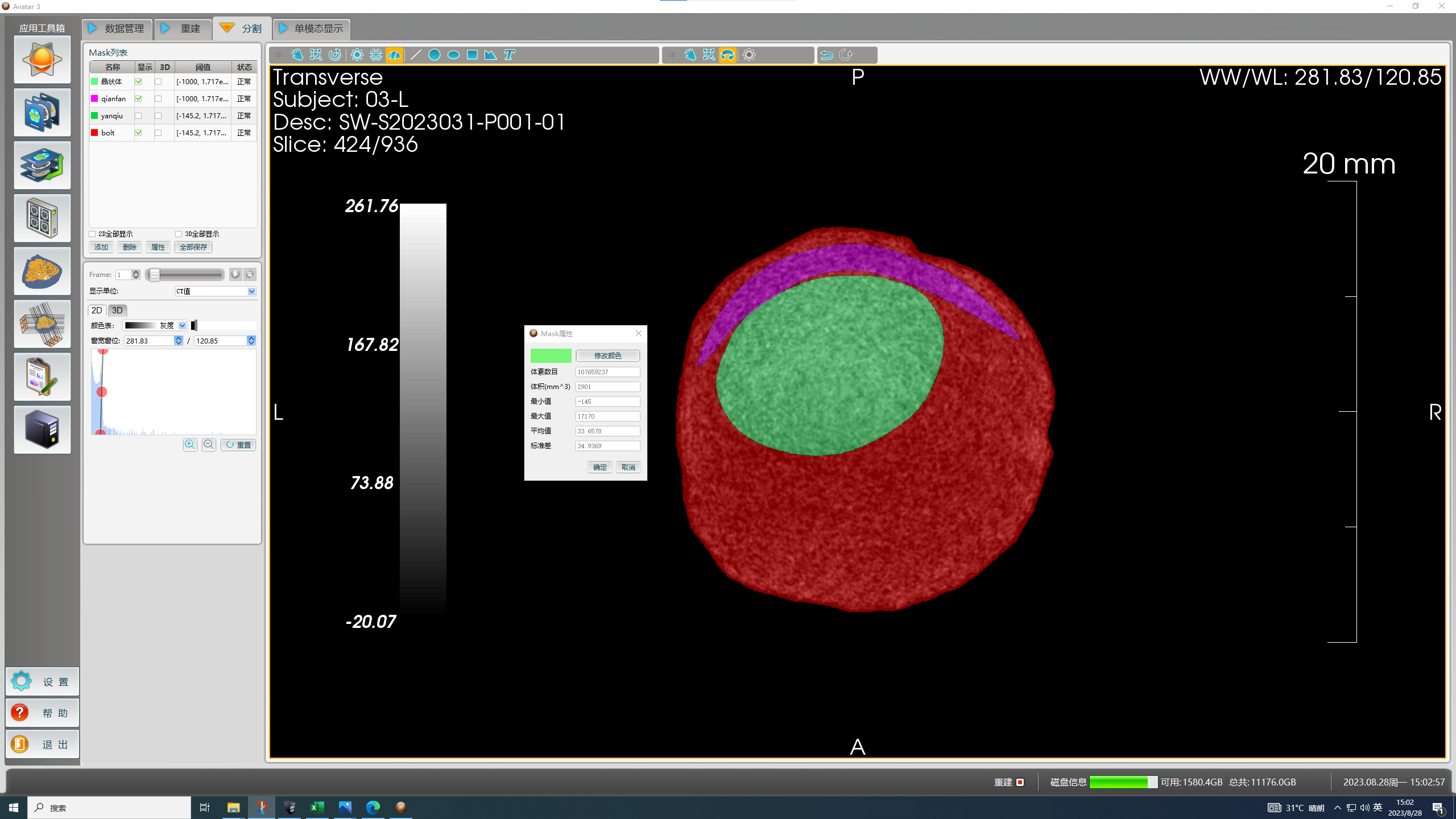

Supplement: S3 Data — (ZIP) [file pone.0310830.s003.zip › CT_rabbits/Eyeball volume/03-L.png]

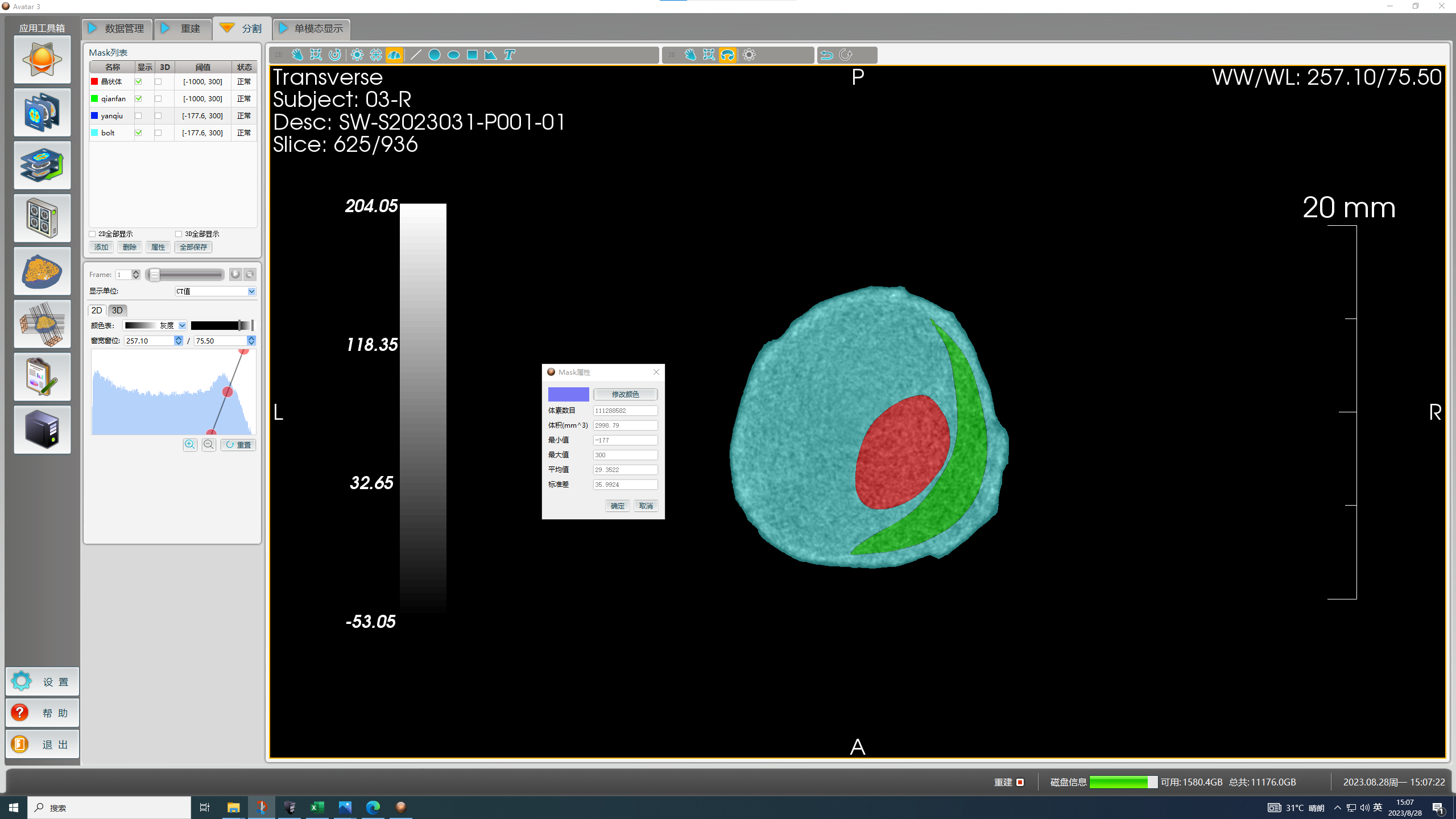

Supplement: S3 Data — (ZIP) [file pone.0310830.s003.zip › CT_rabbits/Eyeball volume/03-R.png]

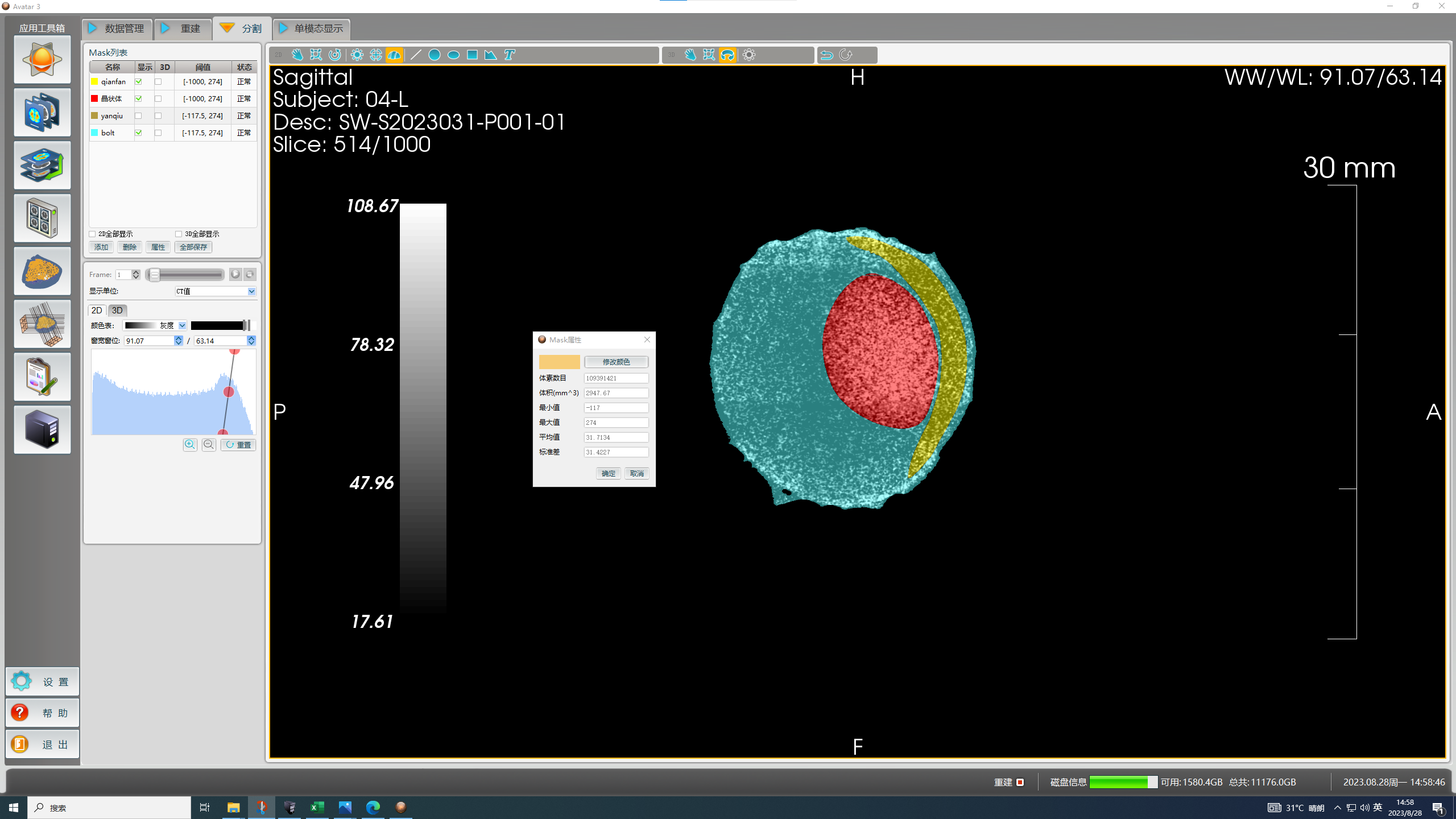

Supplement: S3 Data — (ZIP) [file pone.0310830.s003.zip › CT_rabbits/Eyeball volume/04-L.png]

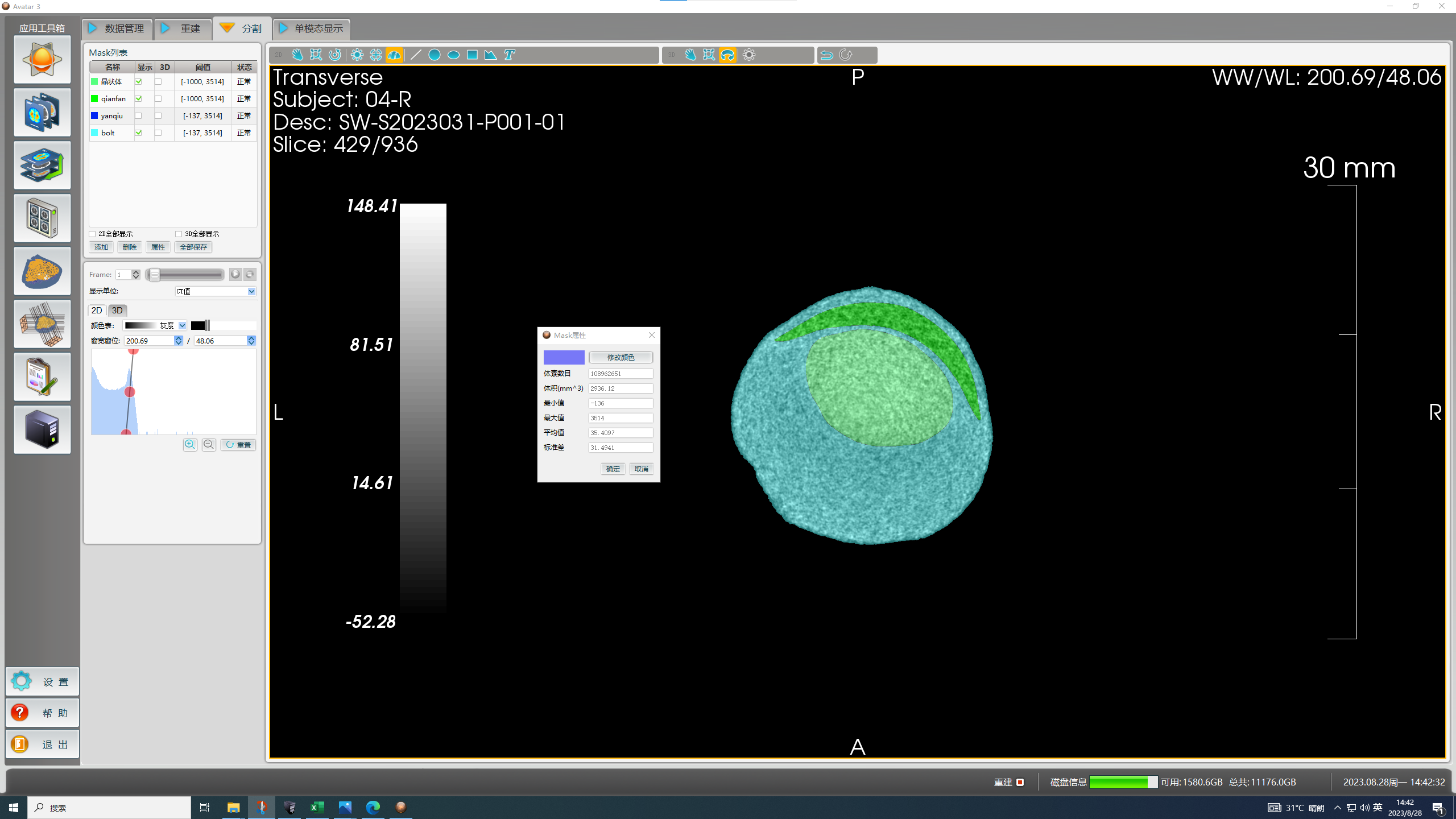

Supplement: S3 Data — (ZIP) [file pone.0310830.s003.zip › CT_rabbits/Eyeball volume/04-R.png]

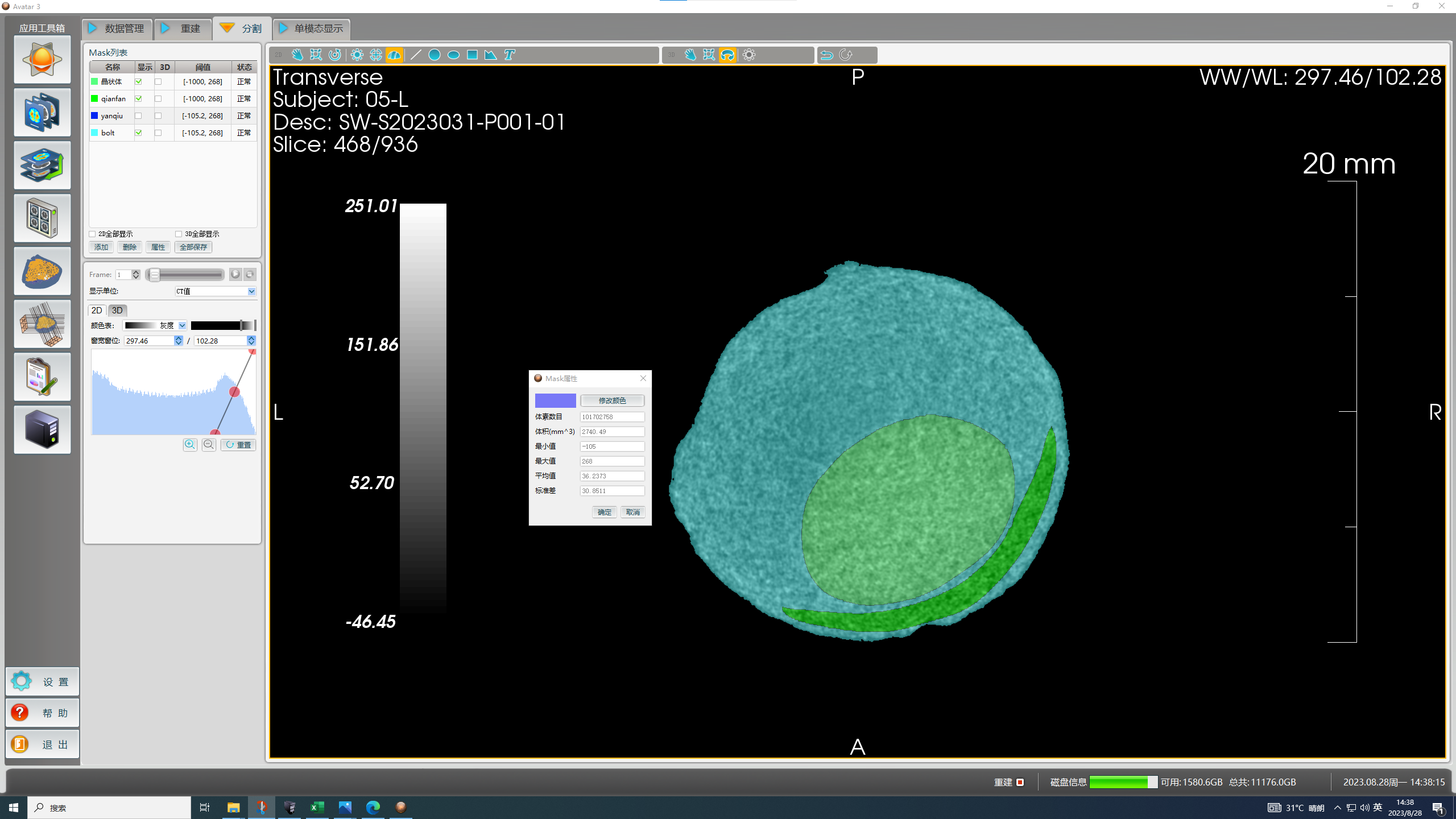

Supplement: S3 Data — (ZIP) [file pone.0310830.s003.zip › CT_rabbits/Eyeball volume/05-L.png]

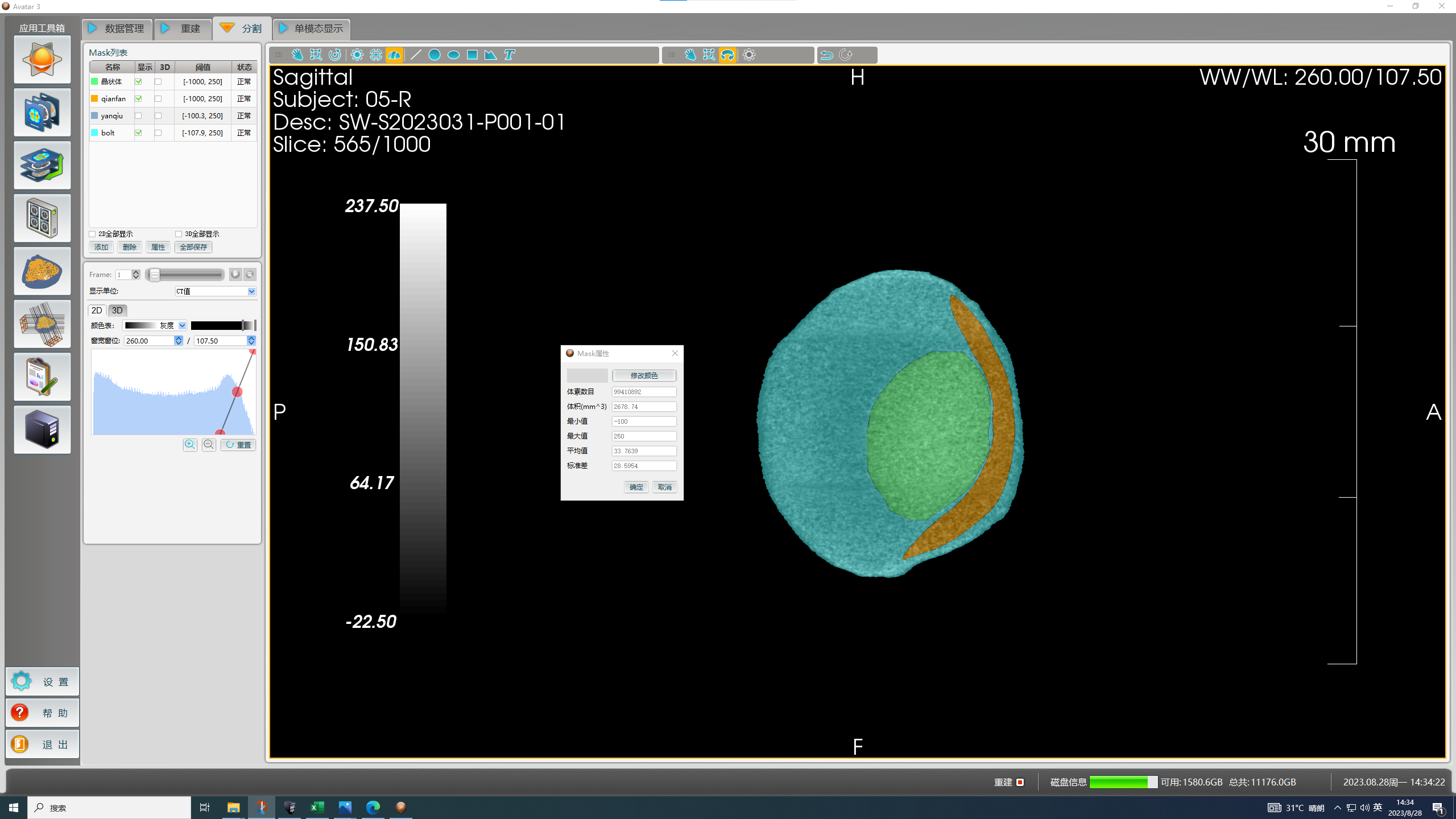

Supplement: S3 Data — (ZIP) [file pone.0310830.s003.zip › CT_rabbits/Eyeball volume/05-R.png]

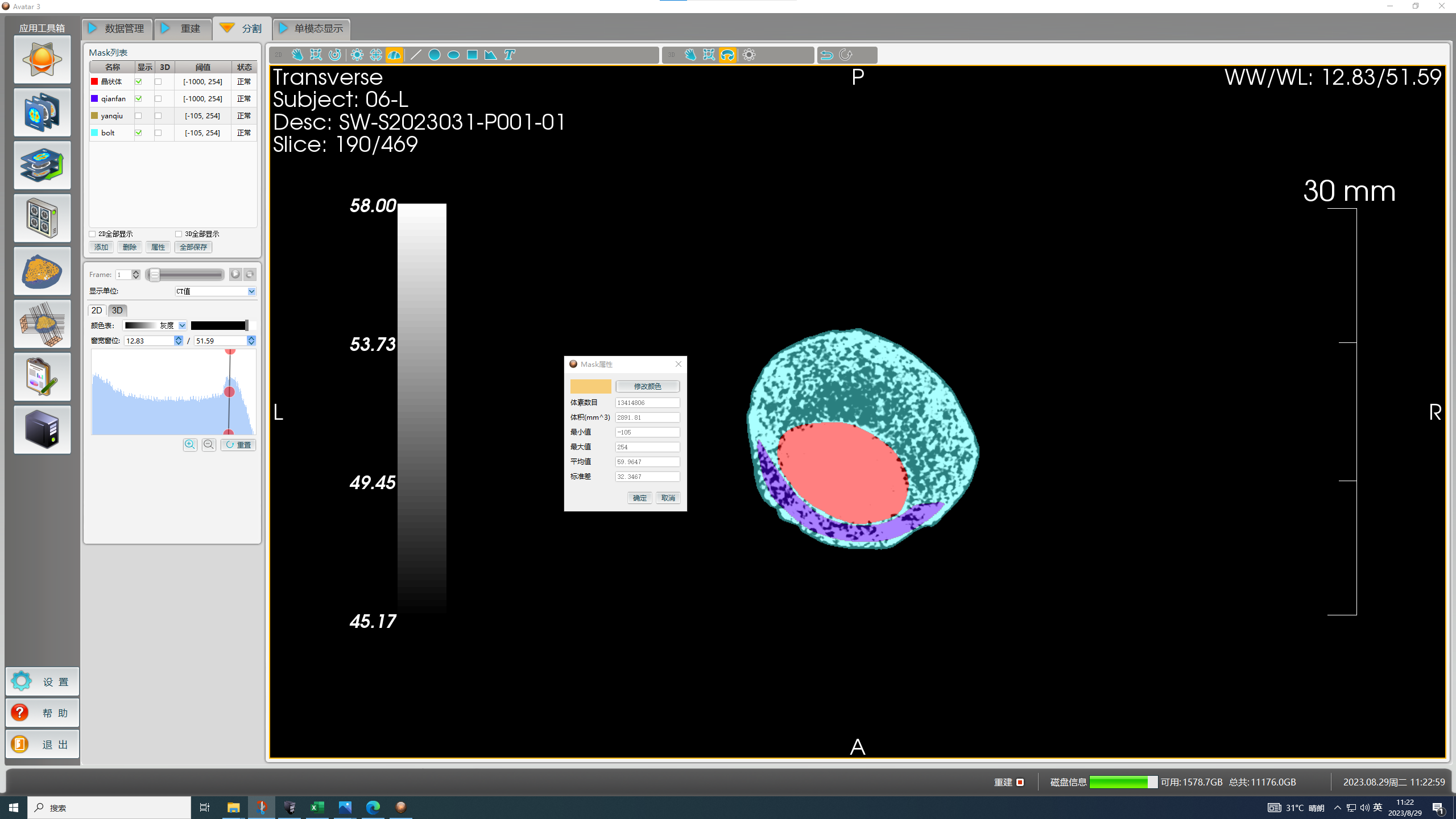

Supplement: S3 Data — (ZIP) [file pone.0310830.s003.zip › CT_rabbits/Eyeball volume/06-L.png]

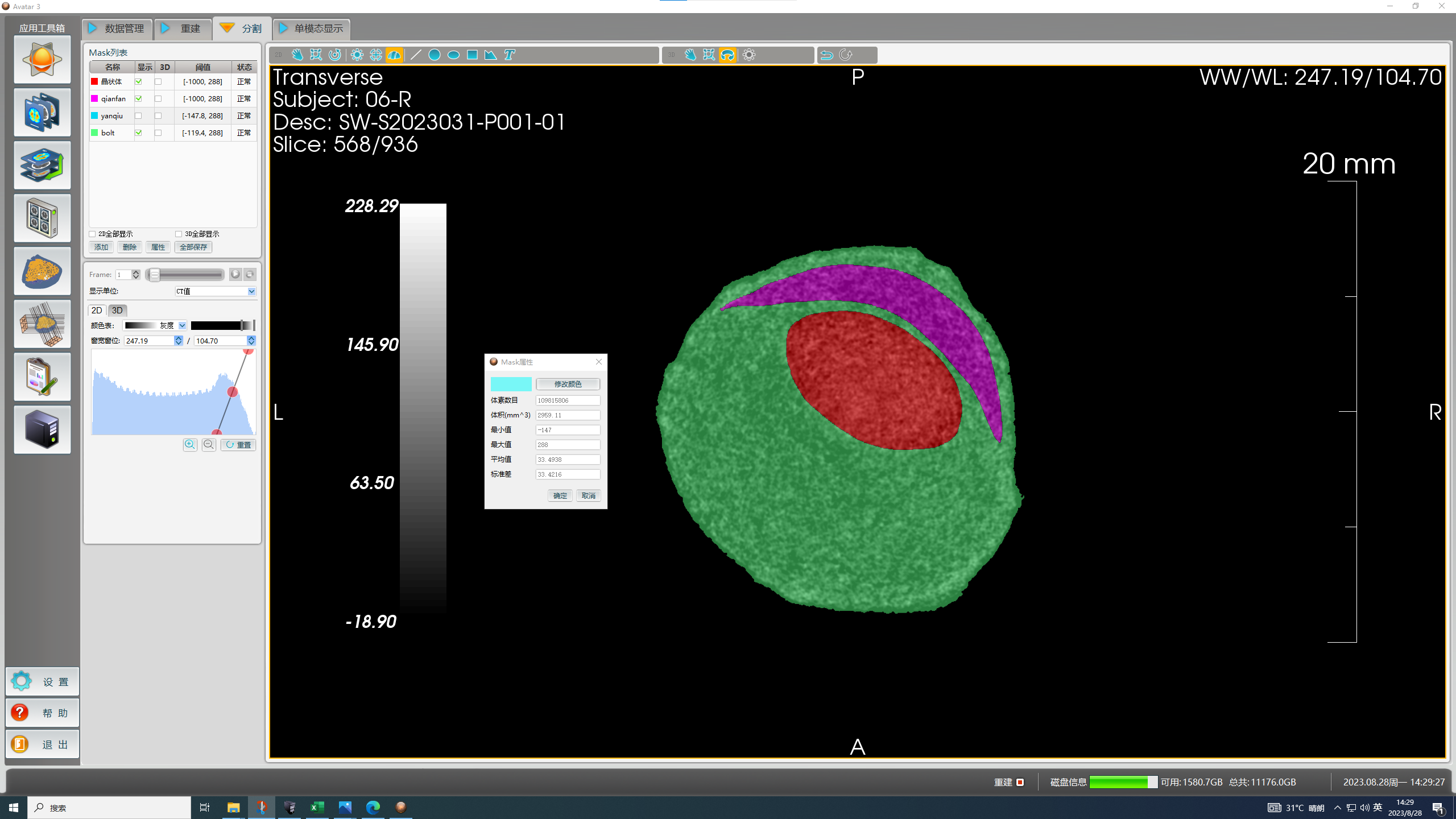

Supplement: S3 Data — (ZIP) [file pone.0310830.s003.zip › CT_rabbits/Eyeball volume/06-R.png]

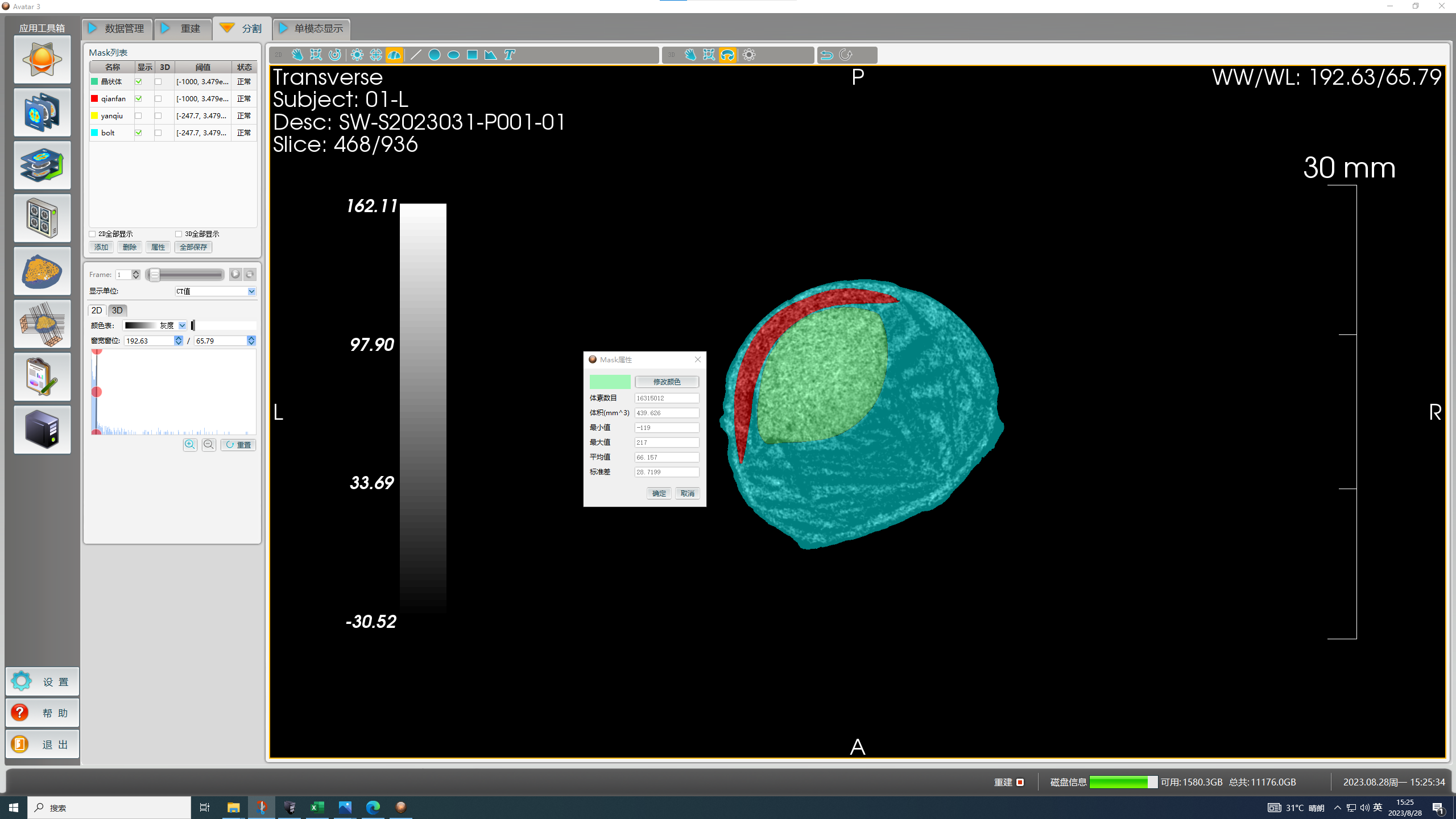

Supplement: S3 Data — (ZIP) [file pone.0310830.s003.zip › CT_rabbits/lens/01-L.png]

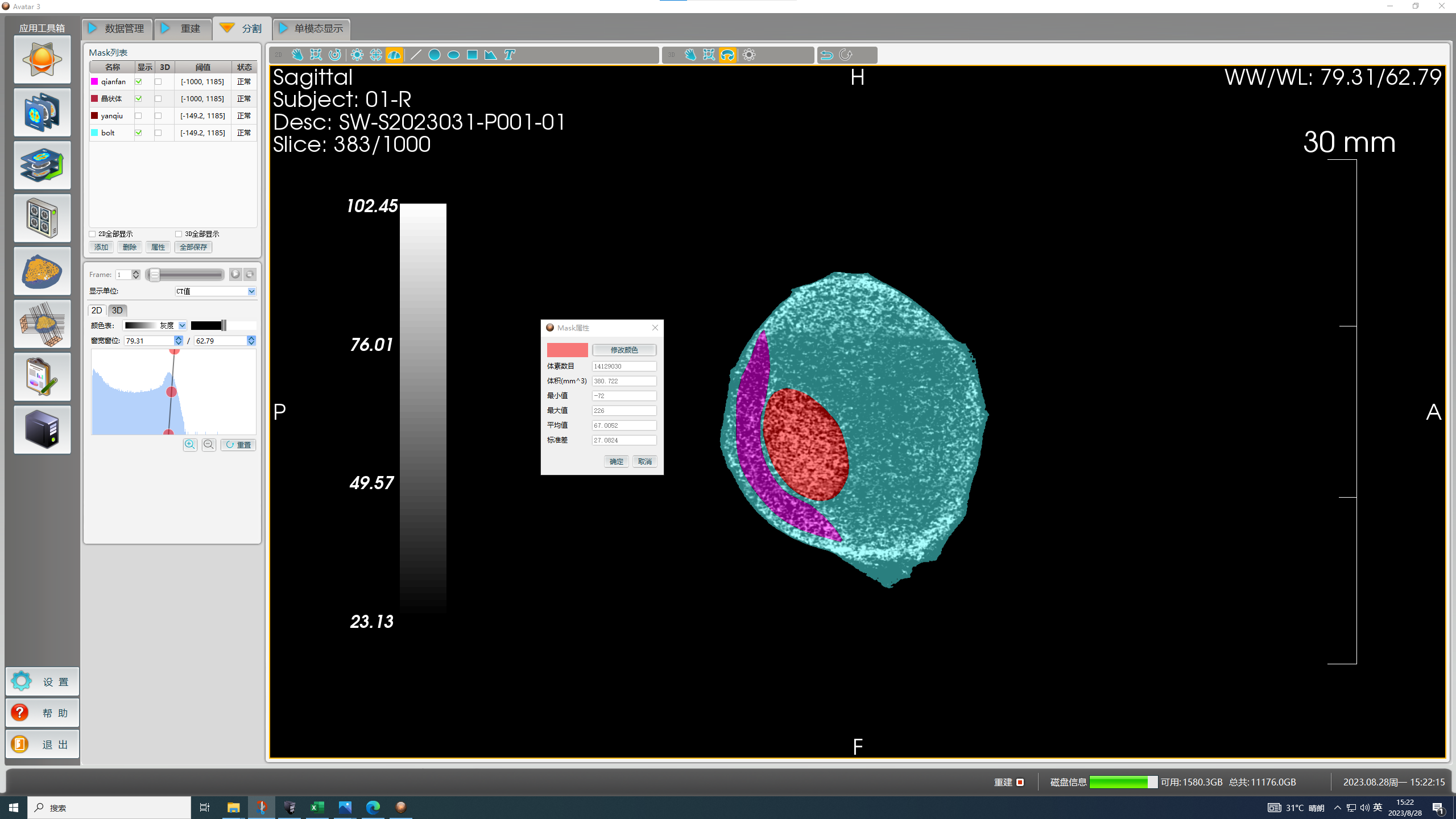

Supplement: S3 Data — (ZIP) [file pone.0310830.s003.zip › CT_rabbits/lens/01-R.png]

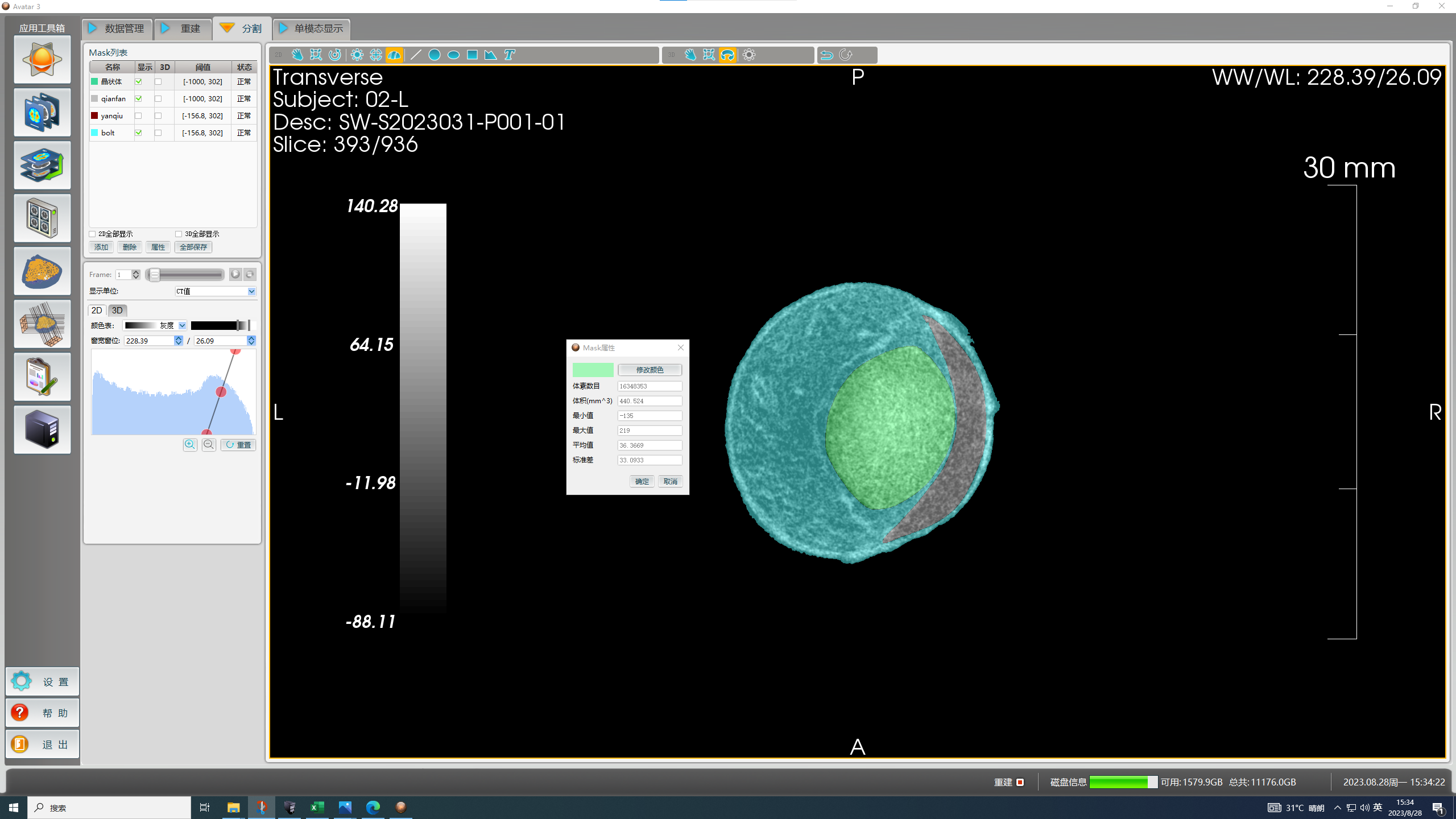

Supplement: S3 Data — (ZIP) [file pone.0310830.s003.zip › CT_rabbits/lens/02-L.png]

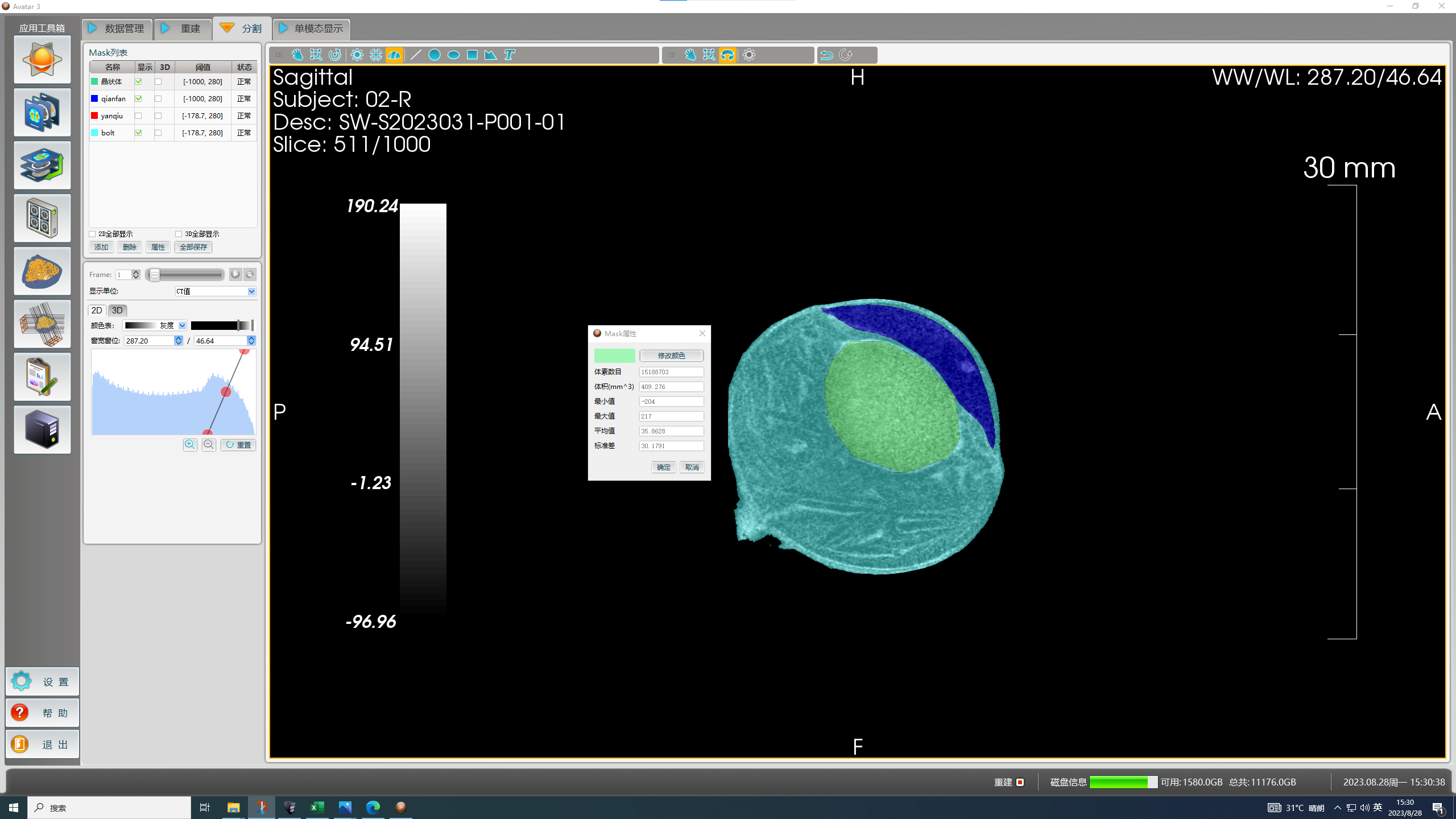

Supplement: S3 Data — (ZIP) [file pone.0310830.s003.zip › CT_rabbits/lens/02-R.png]

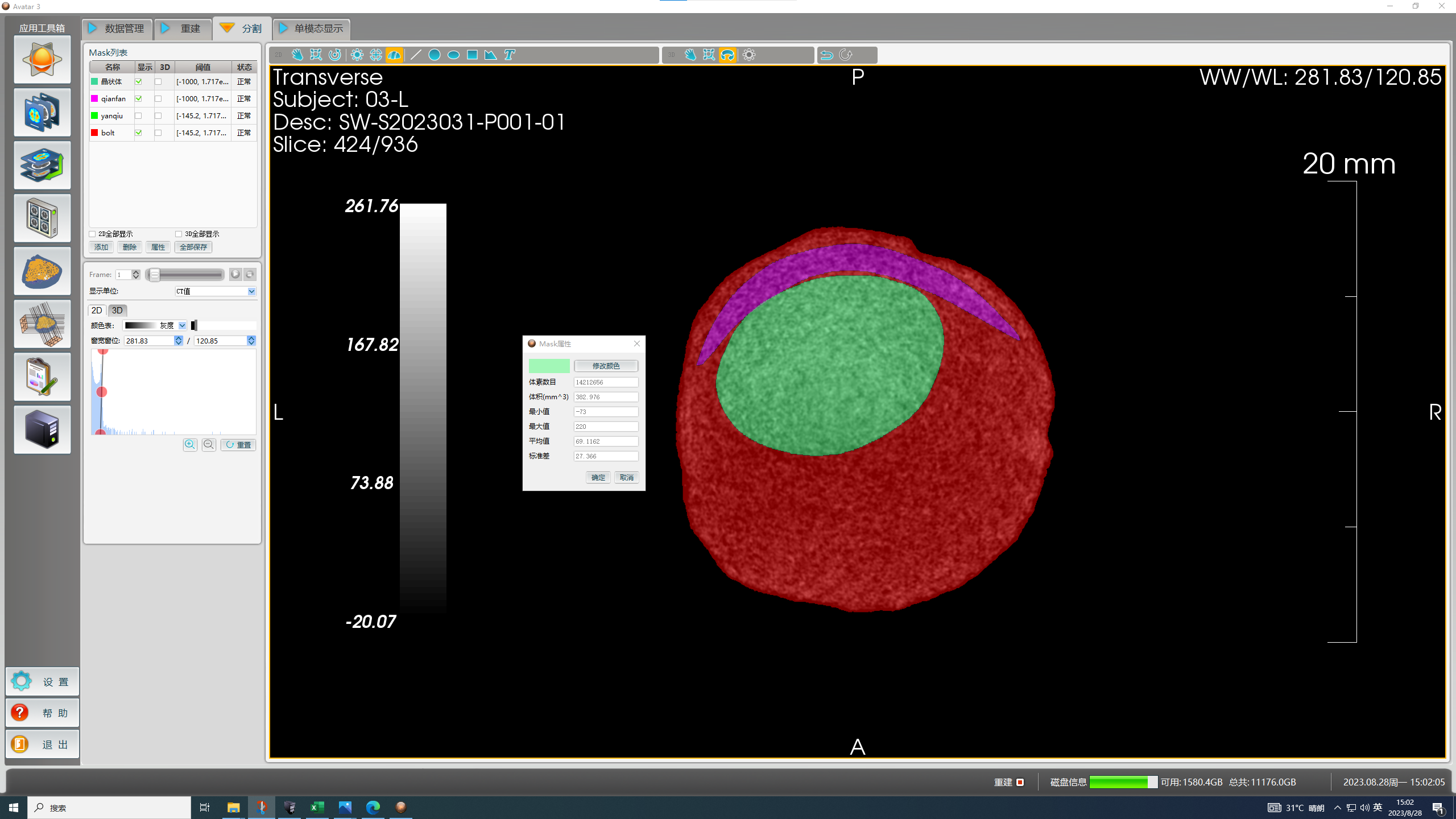

Supplement: S3 Data — (ZIP) [file pone.0310830.s003.zip › CT_rabbits/lens/03-L.png]
