# Supplementary figures and images for: Anatomical and Micro-CT measurement analysis of ocular volume and intraocular volume in adult Bama Miniature pigs, New Zealand rabbits, and Sprague-Dawley rats (part 2 of 2)
Source: PLoS One. 2024 Sep 20;19(9):e0310830. doi: 10.1371/journal.pone.0310830 (PMC11414937; doi:10.1371/journal.pone.0310830)

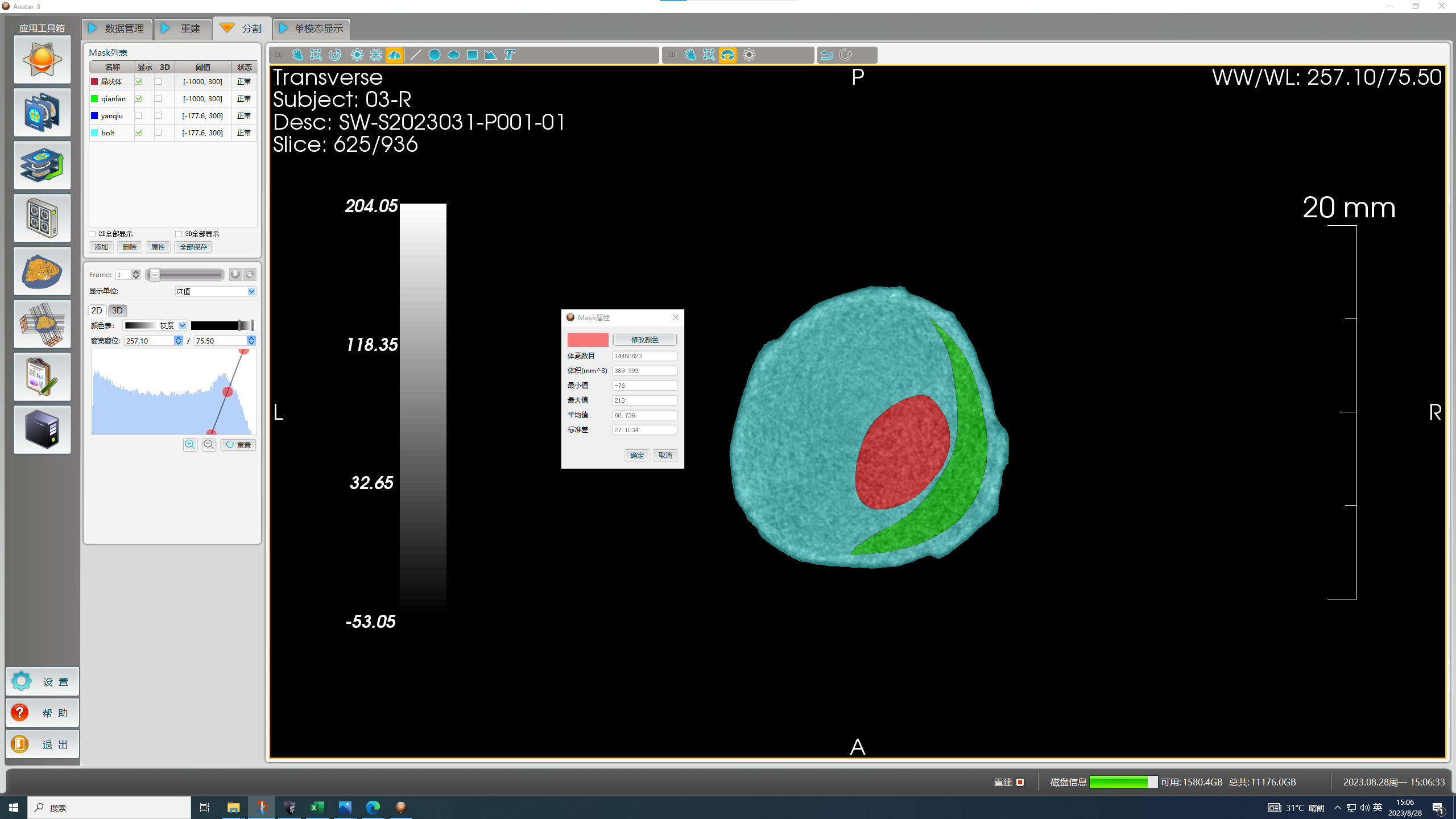

Supplement: S3 Data — (ZIP) [file pone.0310830.s003.zip › CT_rabbits/lens/03-R.png]

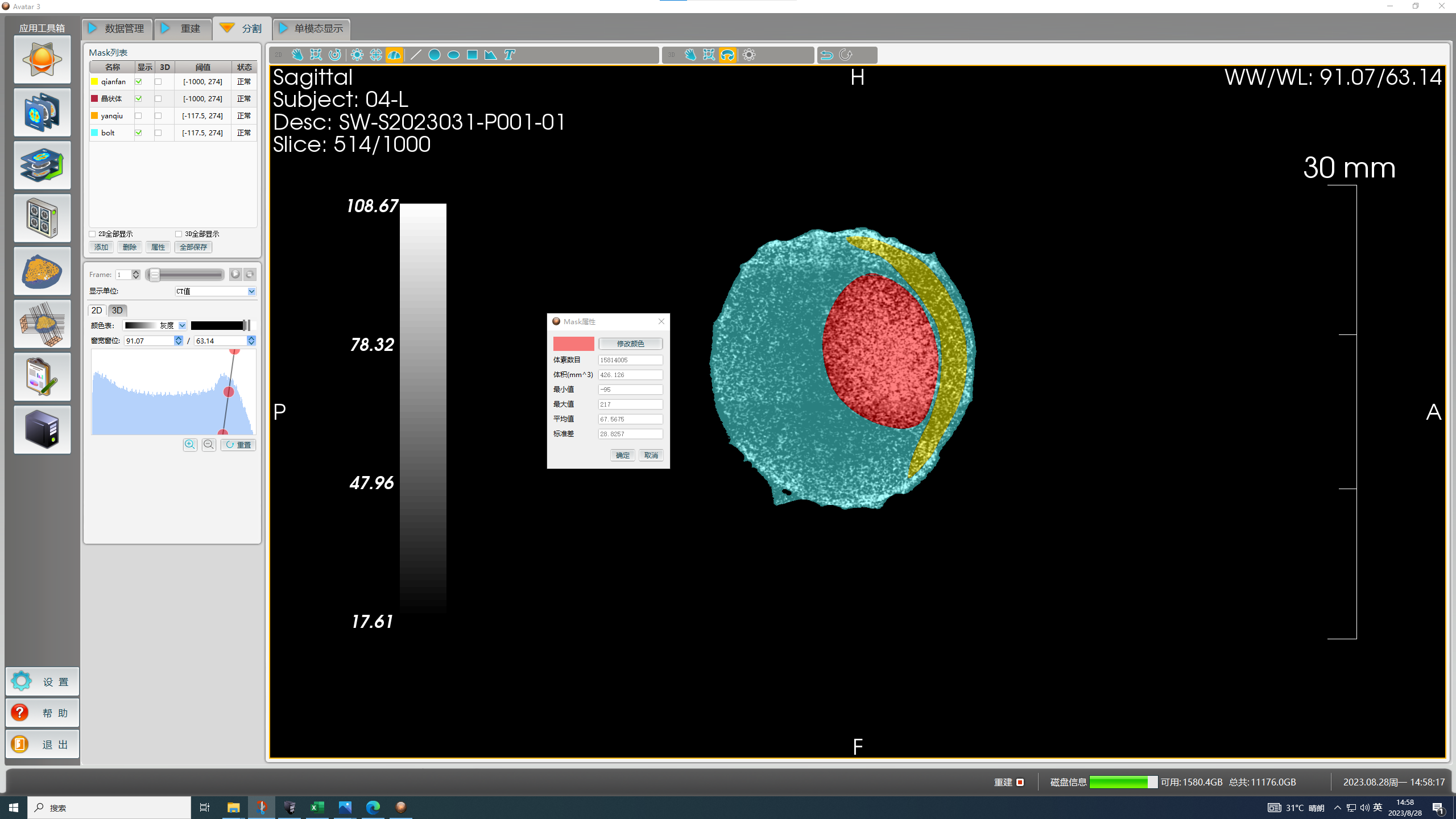

Supplement: S3 Data — (ZIP) [file pone.0310830.s003.zip › CT_rabbits/lens/04-L.png]

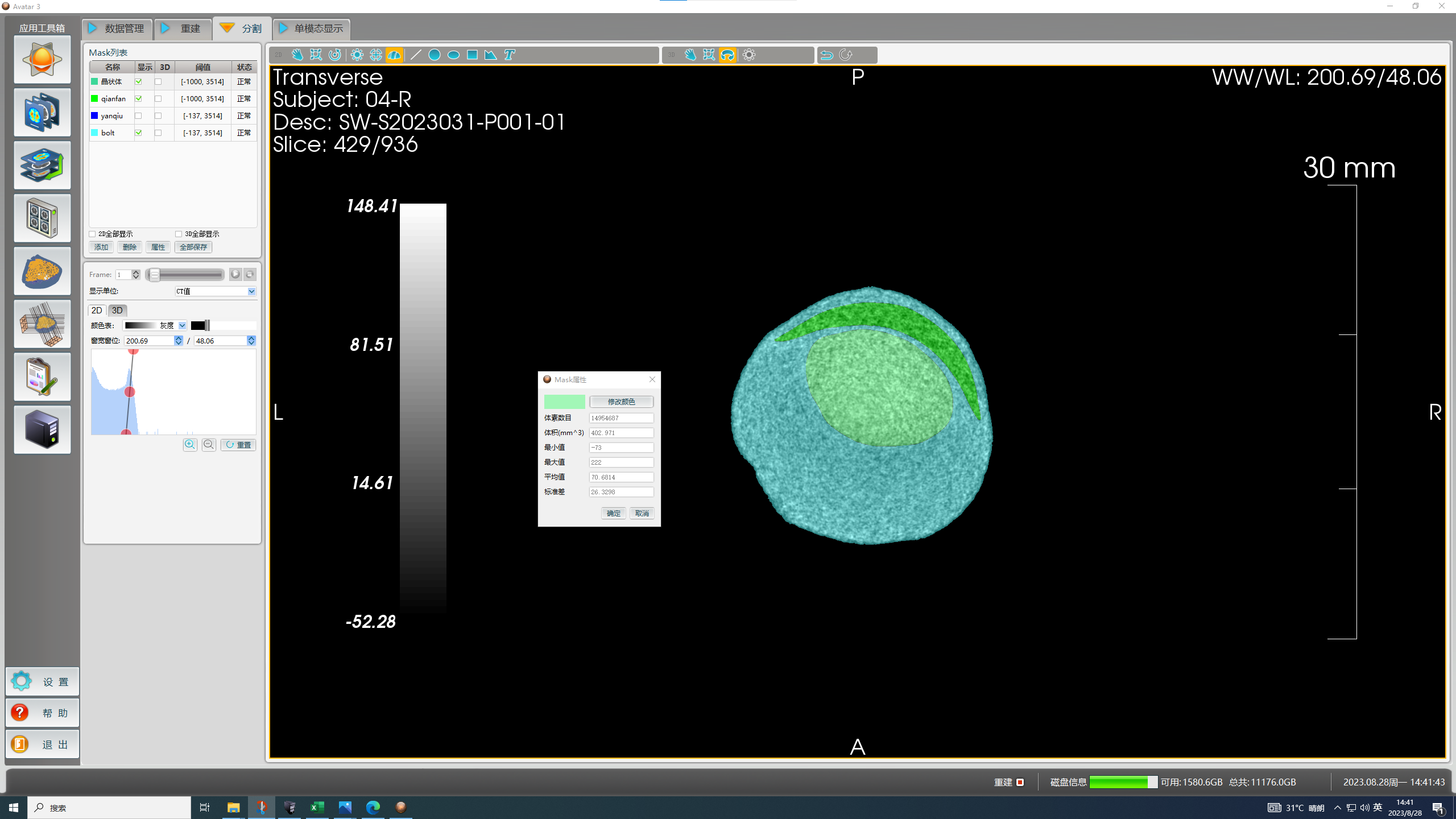

Supplement: S3 Data — (ZIP) [file pone.0310830.s003.zip › CT_rabbits/lens/04-R.png]

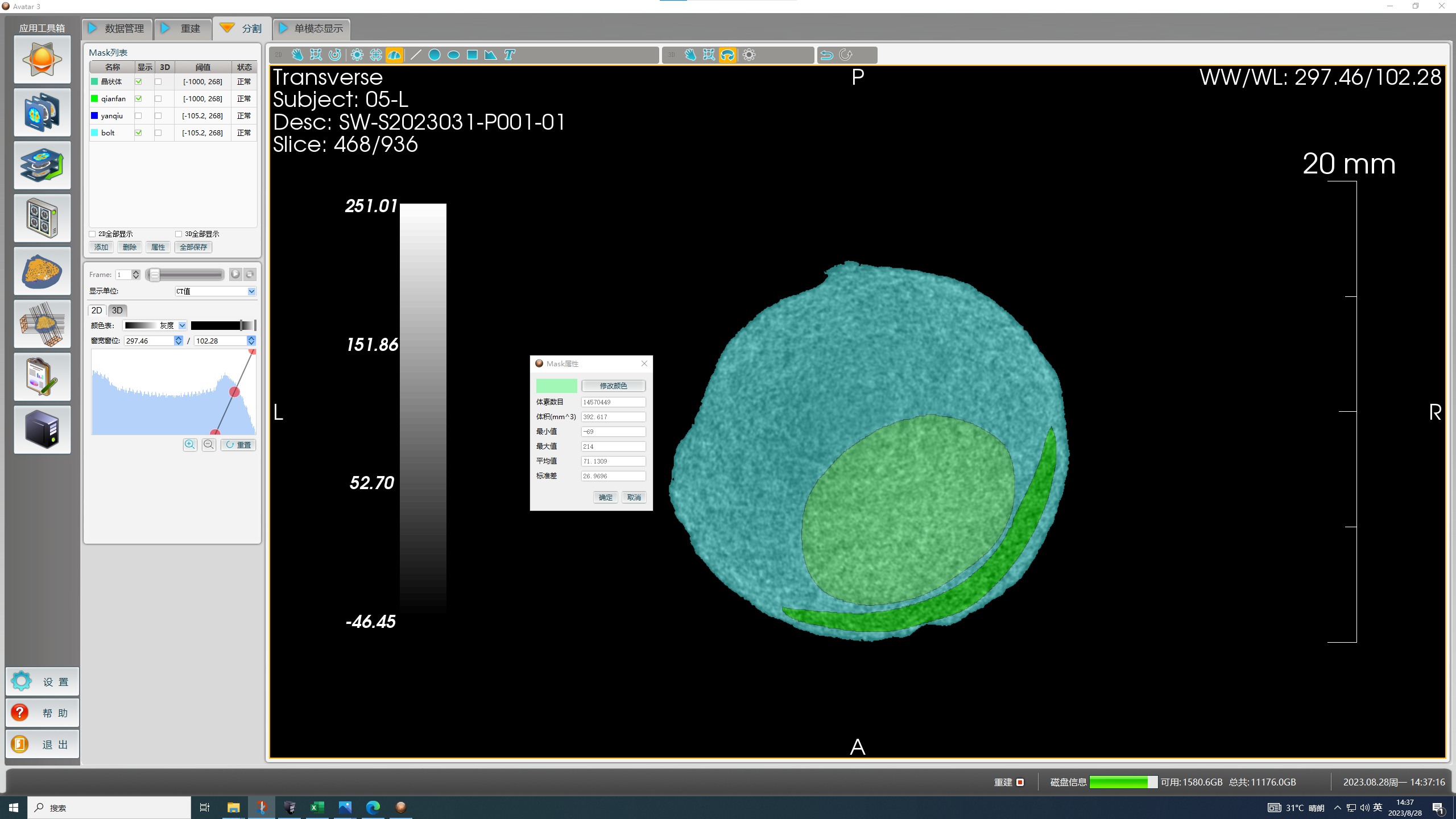

Supplement: S3 Data — (ZIP) [file pone.0310830.s003.zip › CT_rabbits/lens/05-L.png]

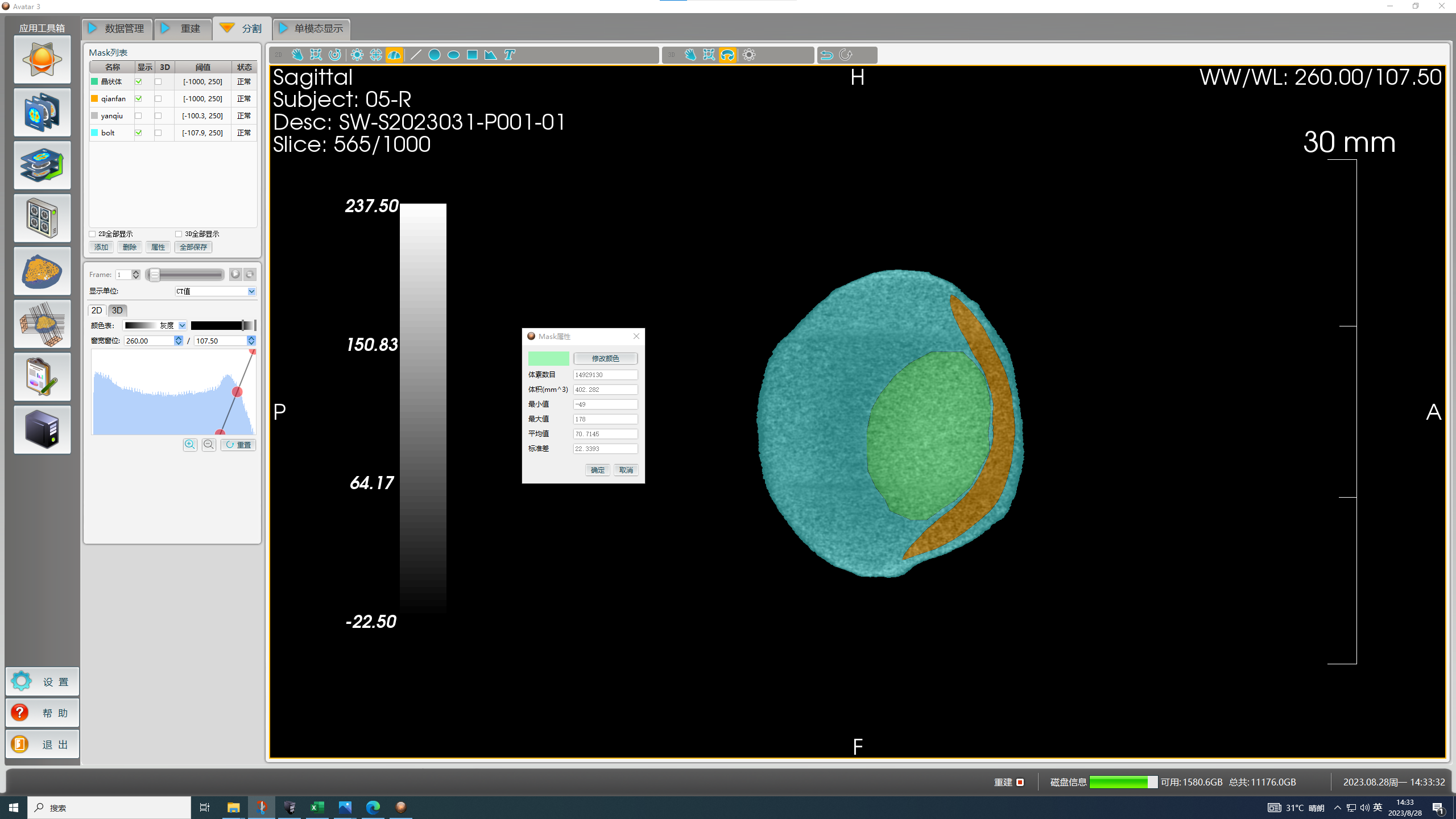

Supplement: S3 Data — (ZIP) [file pone.0310830.s003.zip › CT_rabbits/lens/05-R.png]

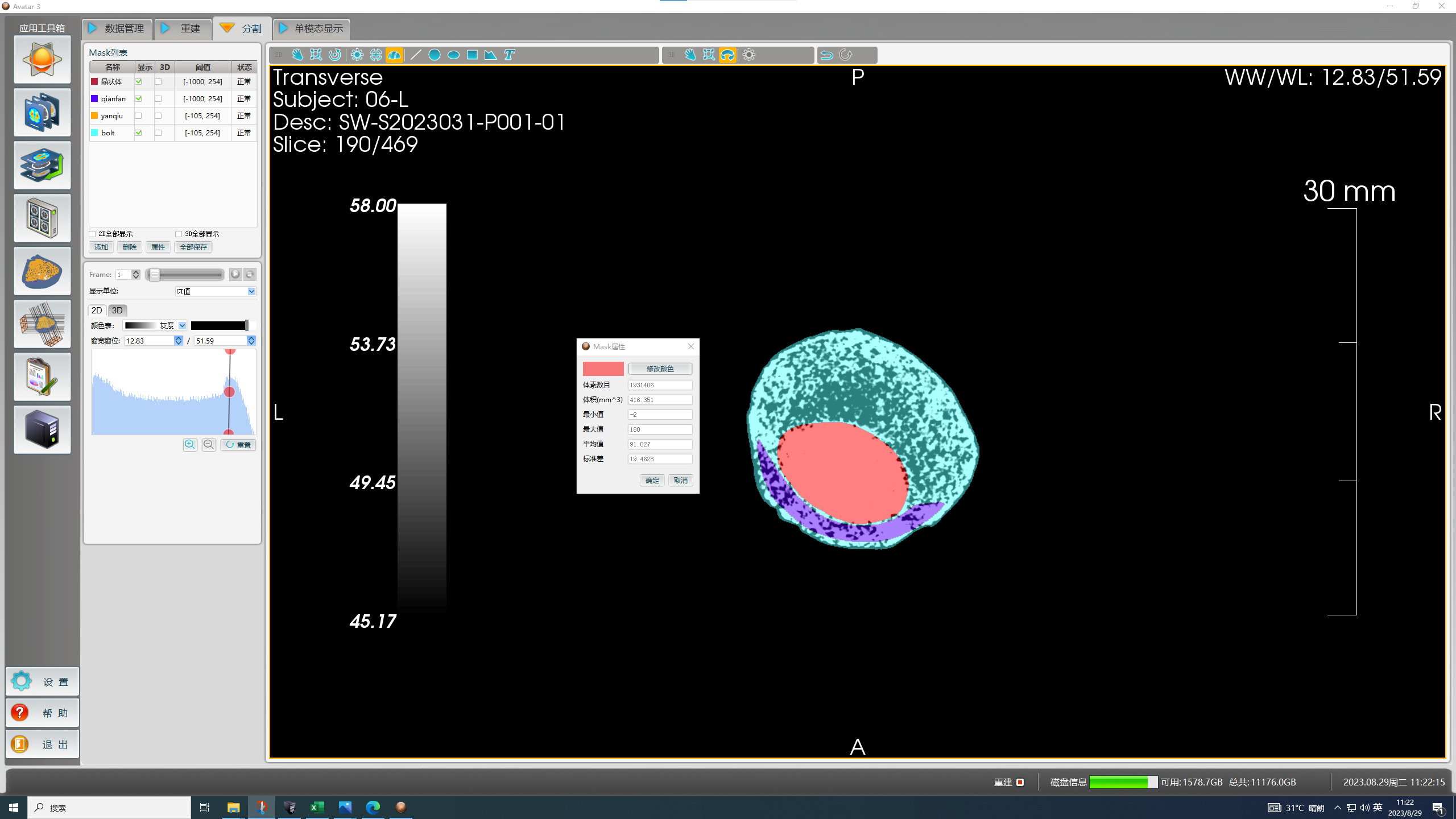

Supplement: S3 Data — (ZIP) [file pone.0310830.s003.zip › CT_rabbits/lens/06-L.png]

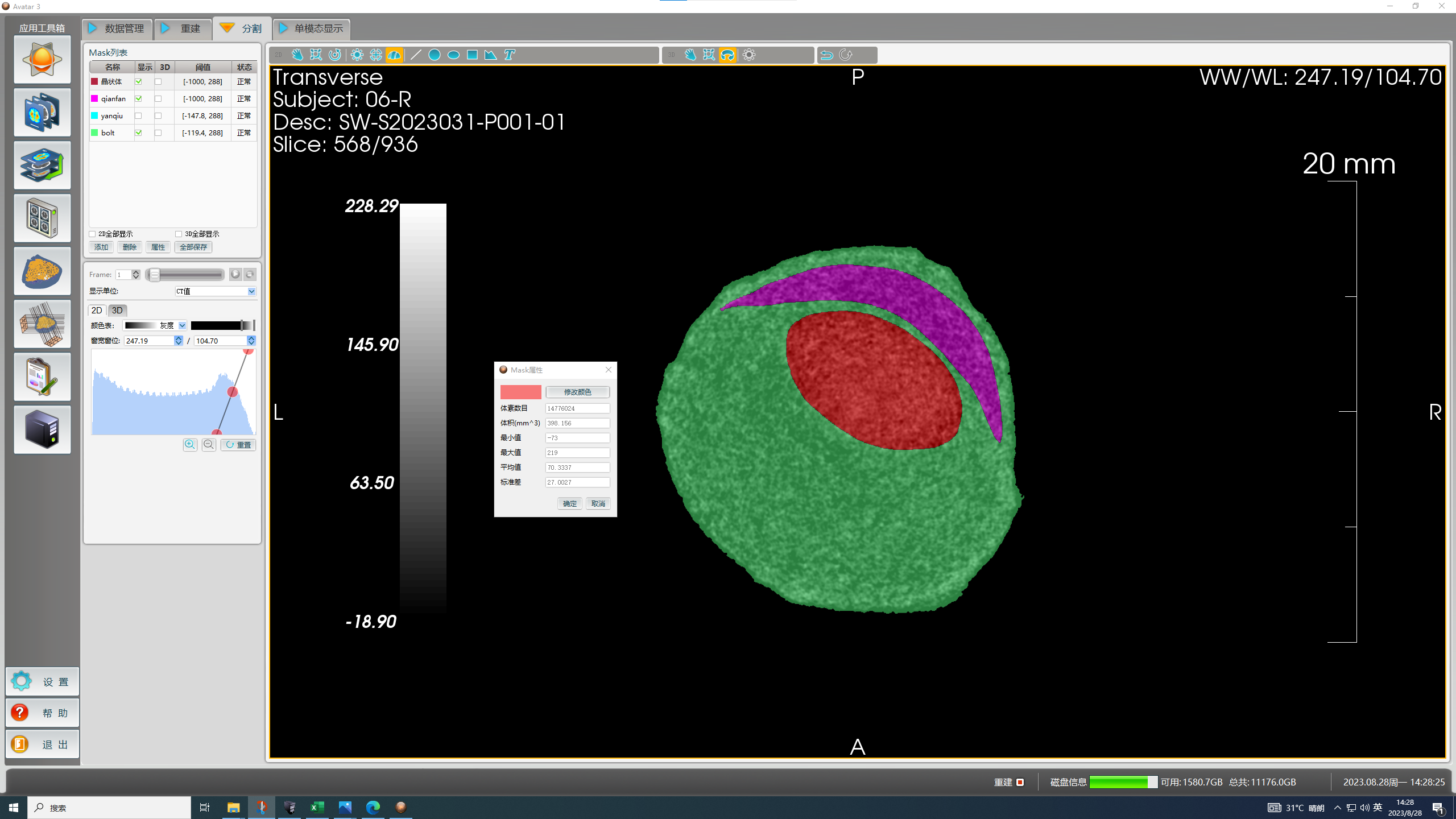

Supplement: S3 Data — (ZIP) [file pone.0310830.s003.zip › CT_rabbits/lens/06-R.png]

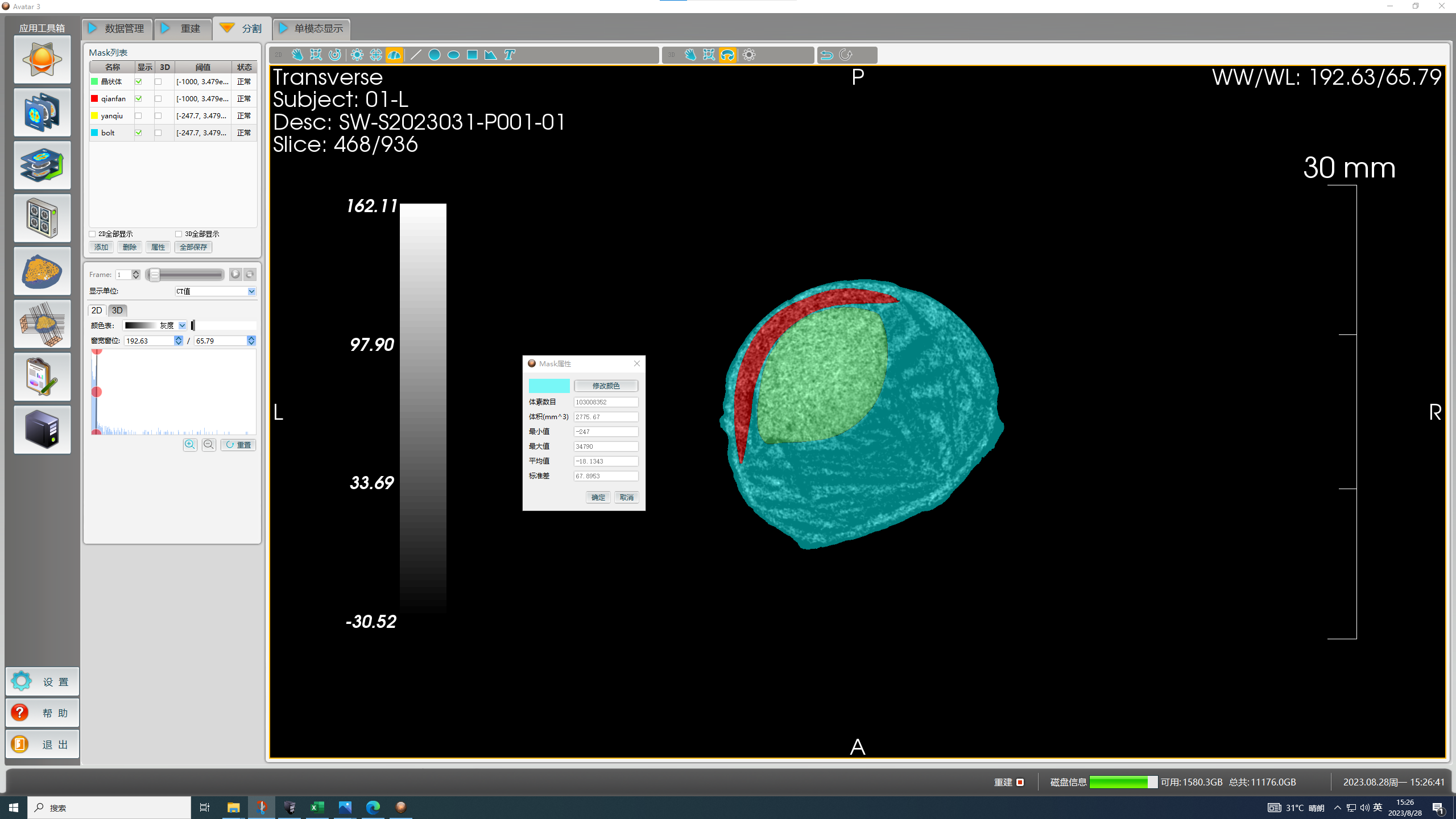

Supplement: S3 Data — (ZIP) [file pone.0310830.s003.zip › CT_rabbits/Vitreous body/01-L.png]

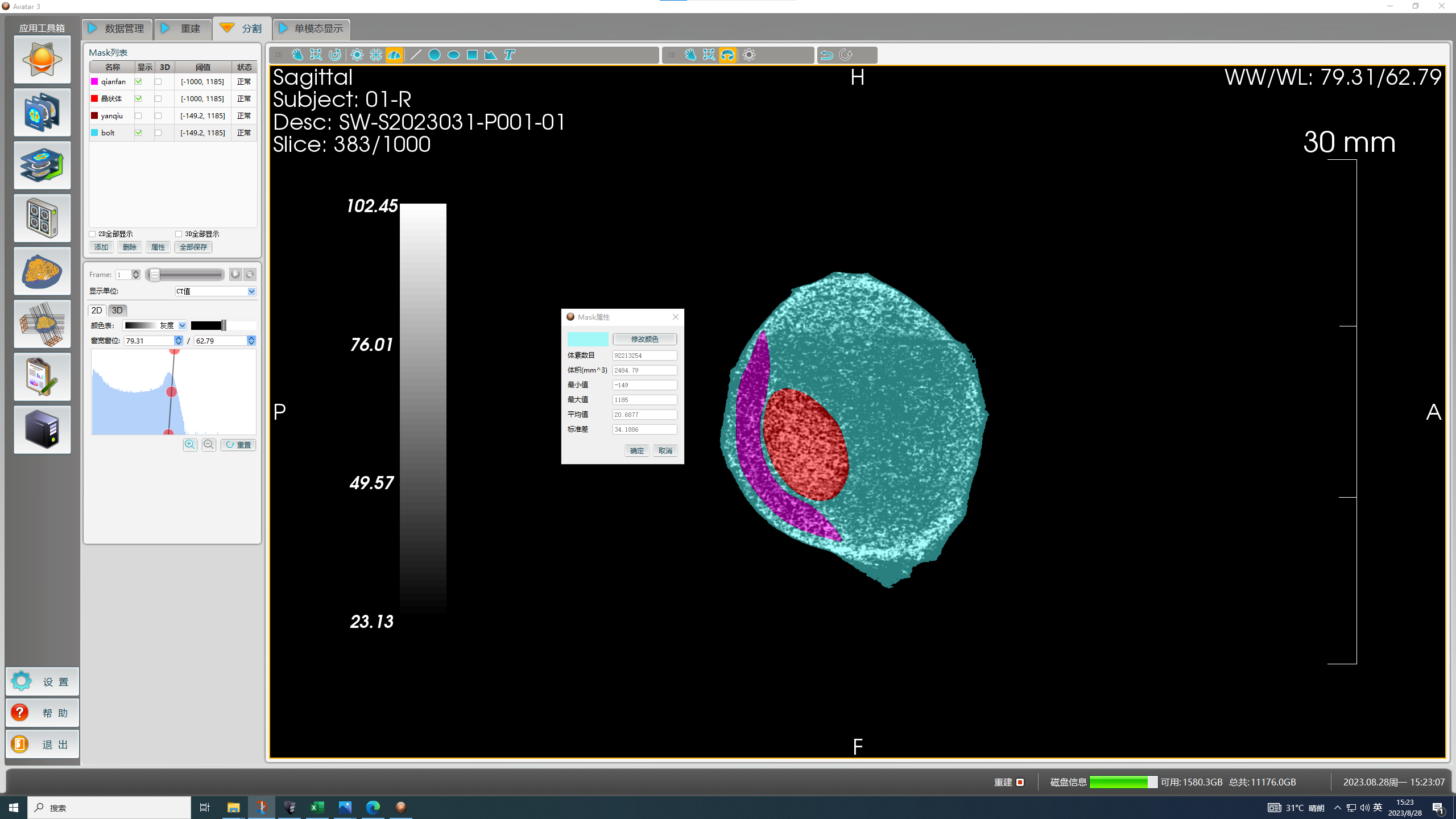

Supplement: S3 Data — (ZIP) [file pone.0310830.s003.zip › CT_rabbits/Vitreous body/01-R.png]

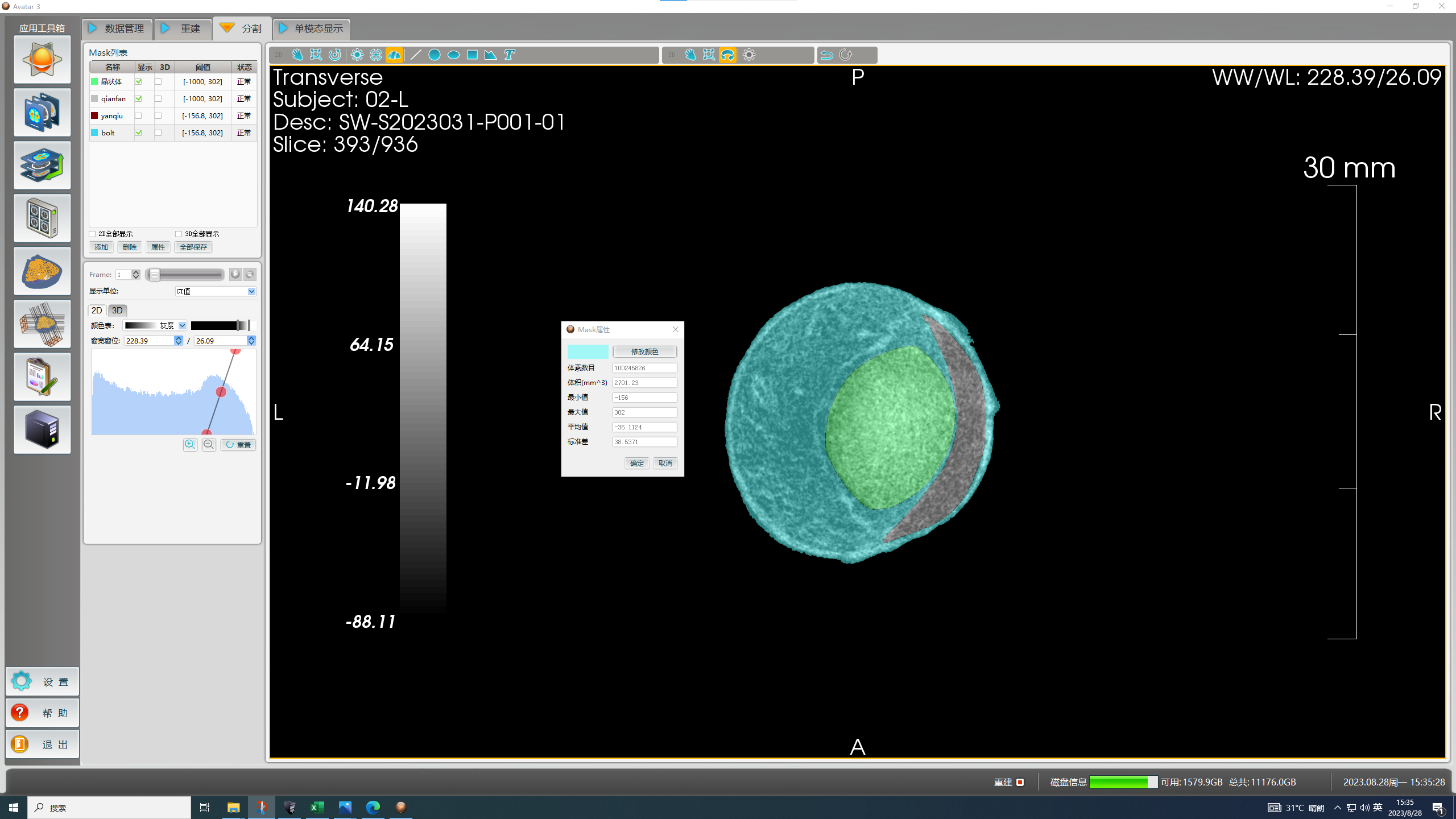

Supplement: S3 Data — (ZIP) [file pone.0310830.s003.zip › CT_rabbits/Vitreous body/02-L.png]

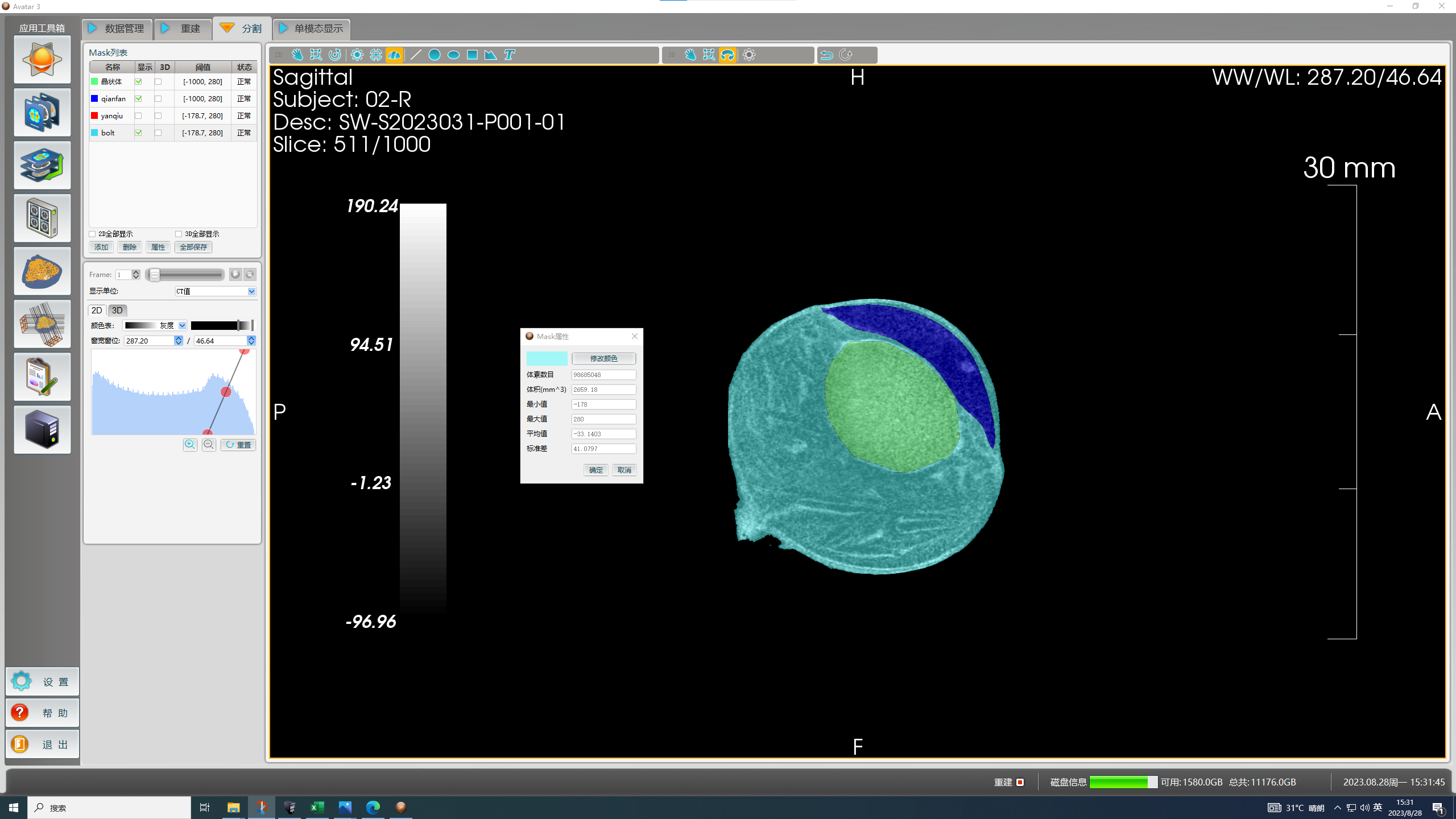

Supplement: S3 Data — (ZIP) [file pone.0310830.s003.zip › CT_rabbits/Vitreous body/02-R.png]

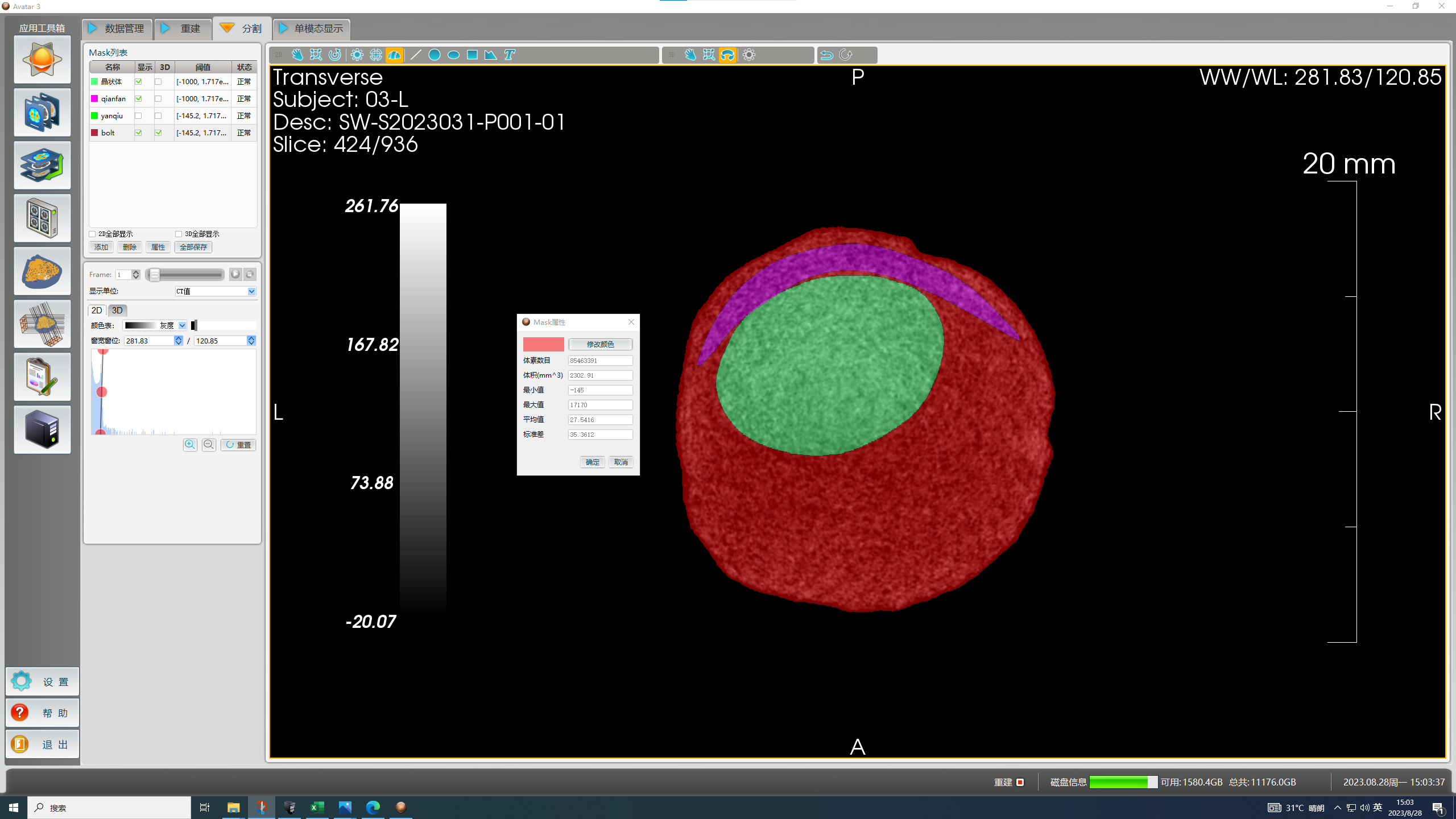

Supplement: S3 Data — (ZIP) [file pone.0310830.s003.zip › CT_rabbits/Vitreous body/03-L.png]

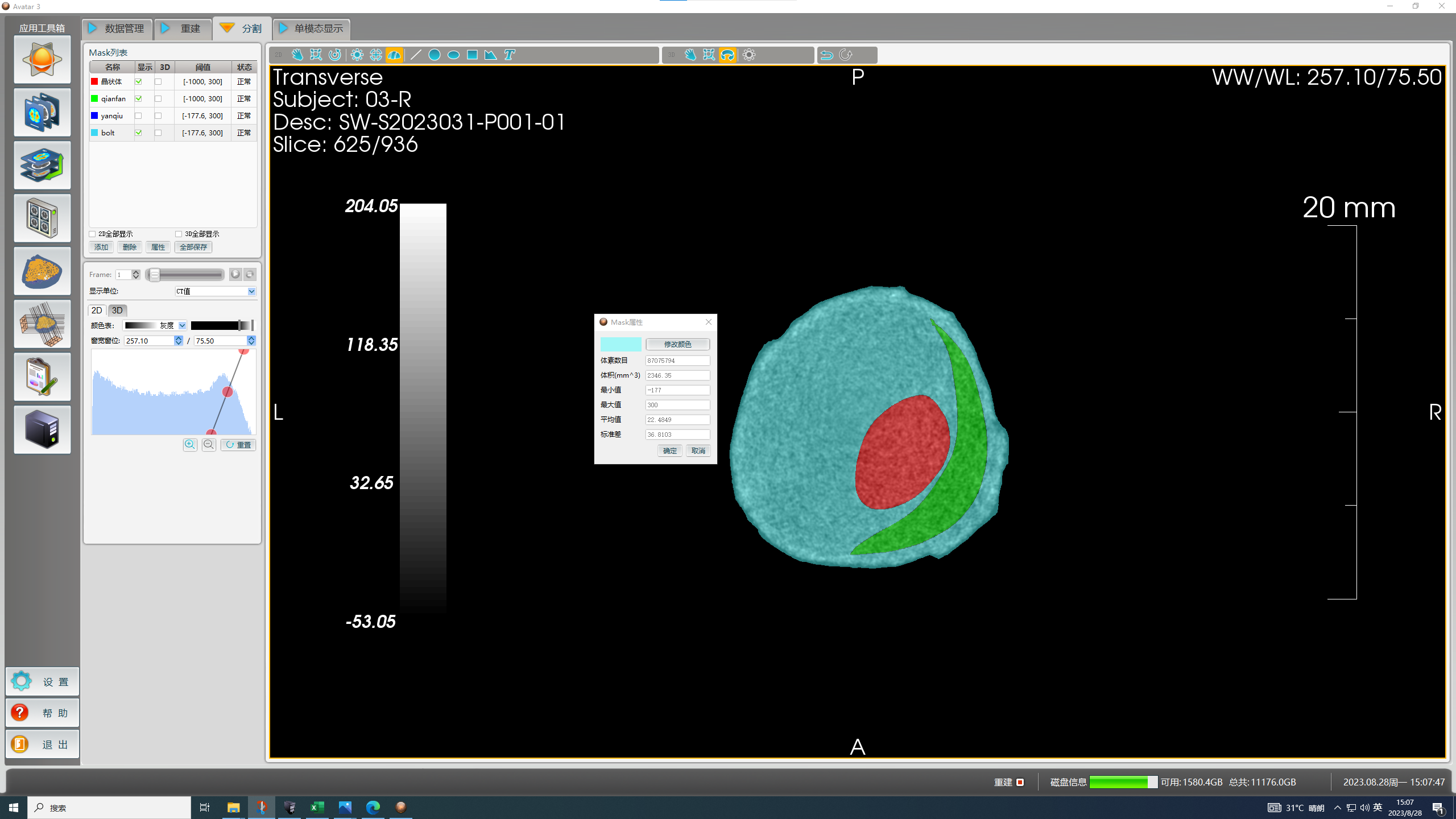

Supplement: S3 Data — (ZIP) [file pone.0310830.s003.zip › CT_rabbits/Vitreous body/03-R.png]

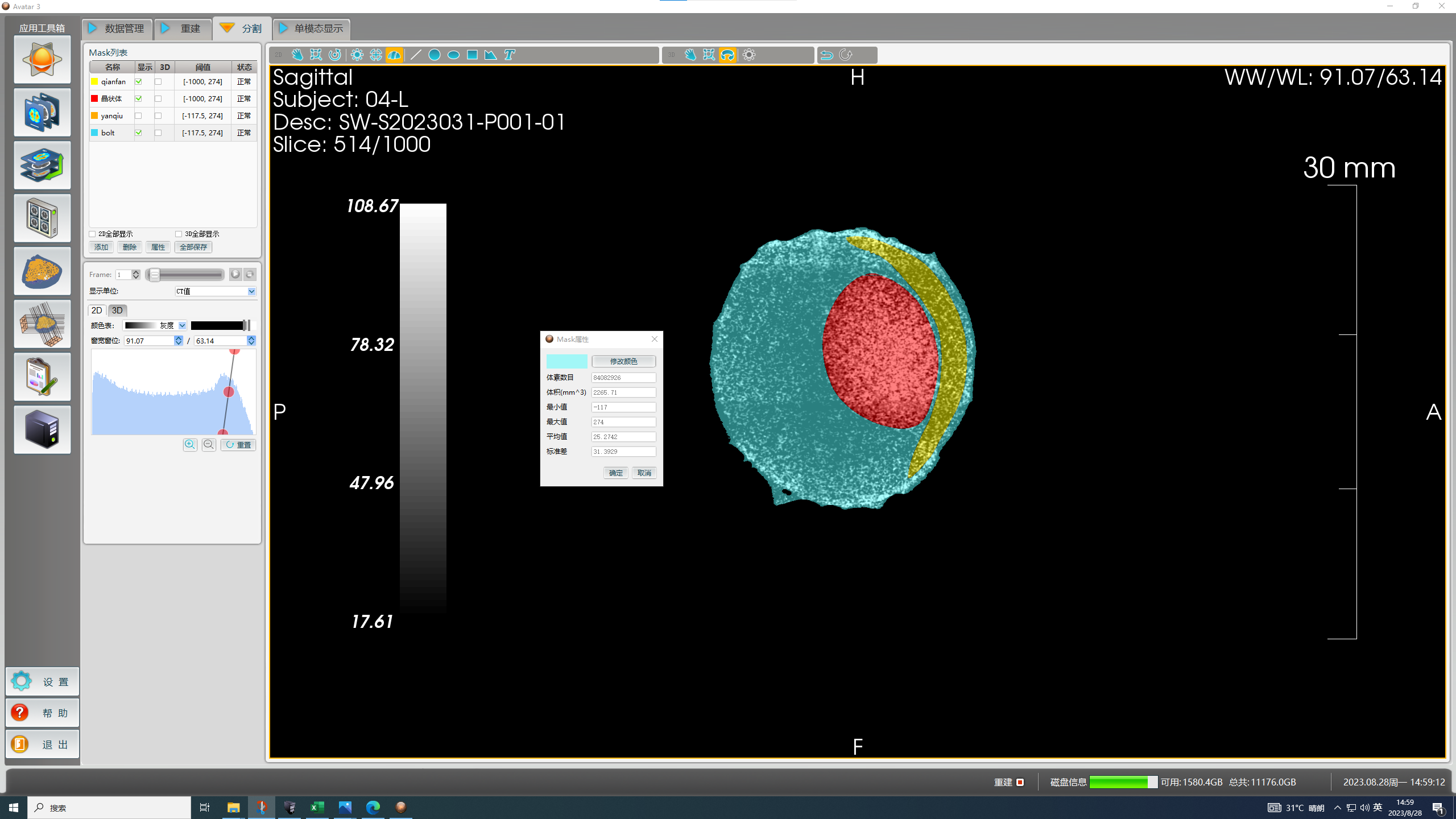

Supplement: S3 Data — (ZIP) [file pone.0310830.s003.zip › CT_rabbits/Vitreous body/04-L.png]

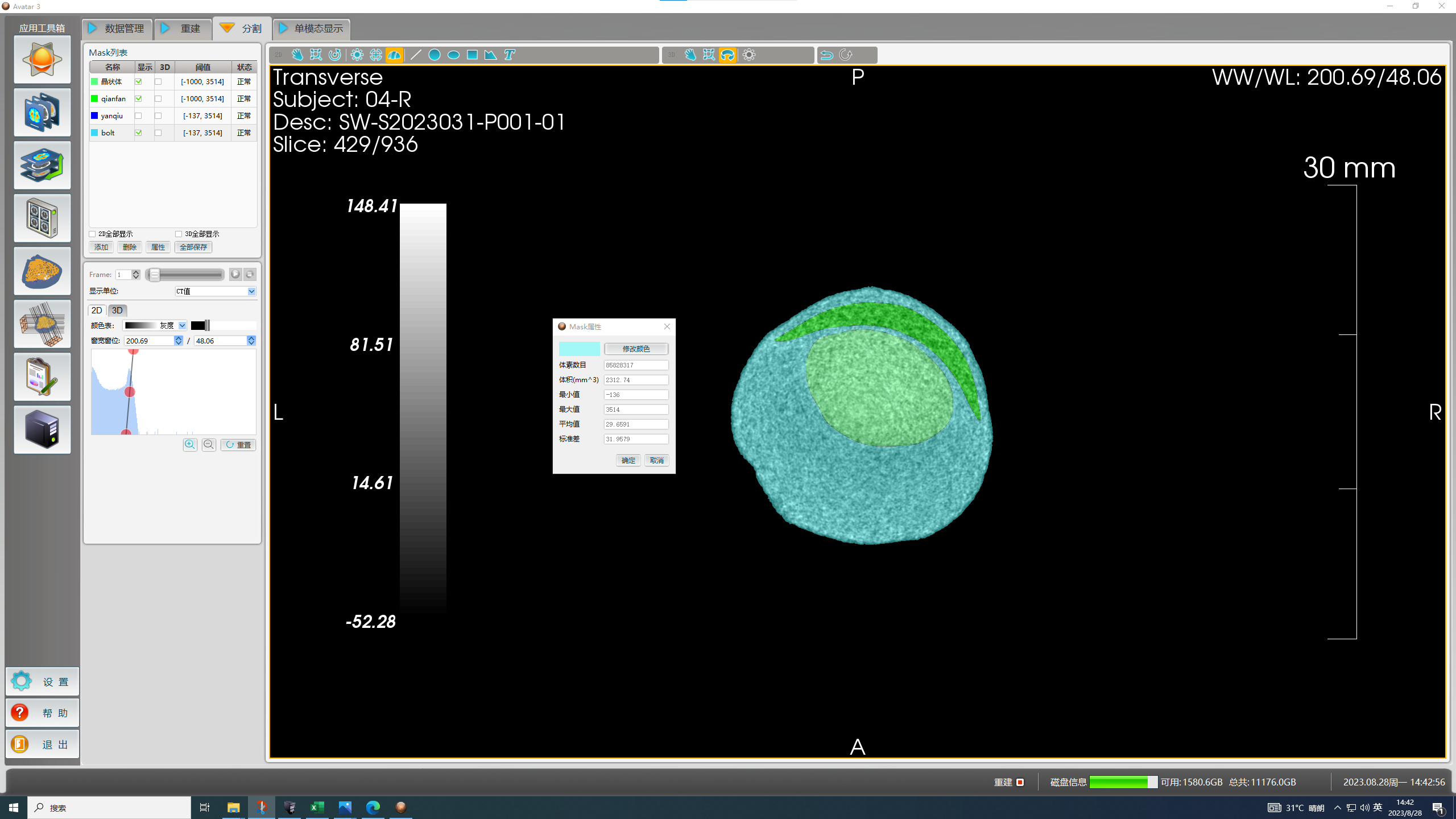

Supplement: S3 Data — (ZIP) [file pone.0310830.s003.zip › CT_rabbits/Vitreous body/04-R.png]

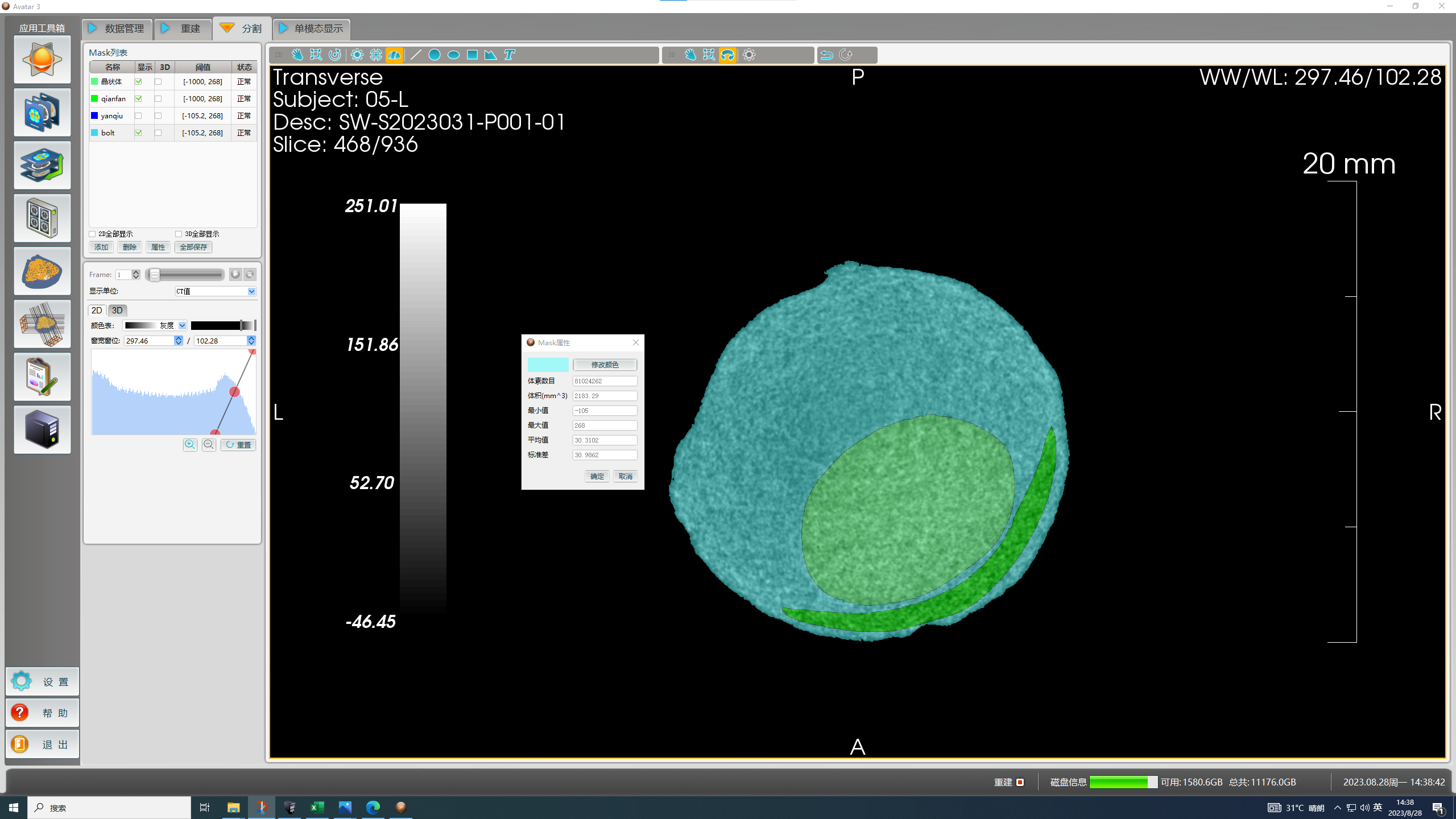

Supplement: S3 Data — (ZIP) [file pone.0310830.s003.zip › CT_rabbits/Vitreous body/05-L.png]

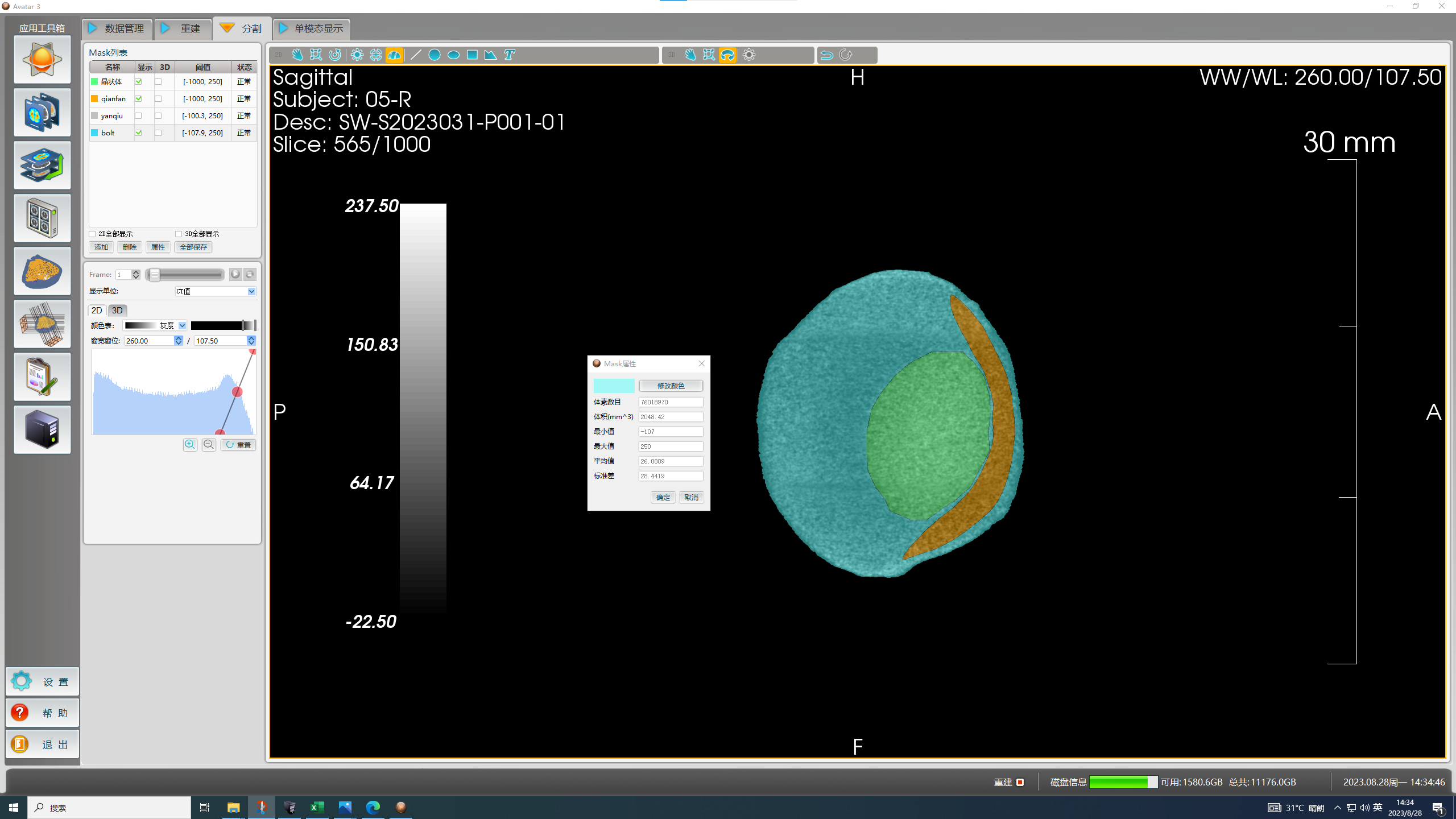

Supplement: S3 Data — (ZIP) [file pone.0310830.s003.zip › CT_rabbits/Vitreous body/05-R.png]

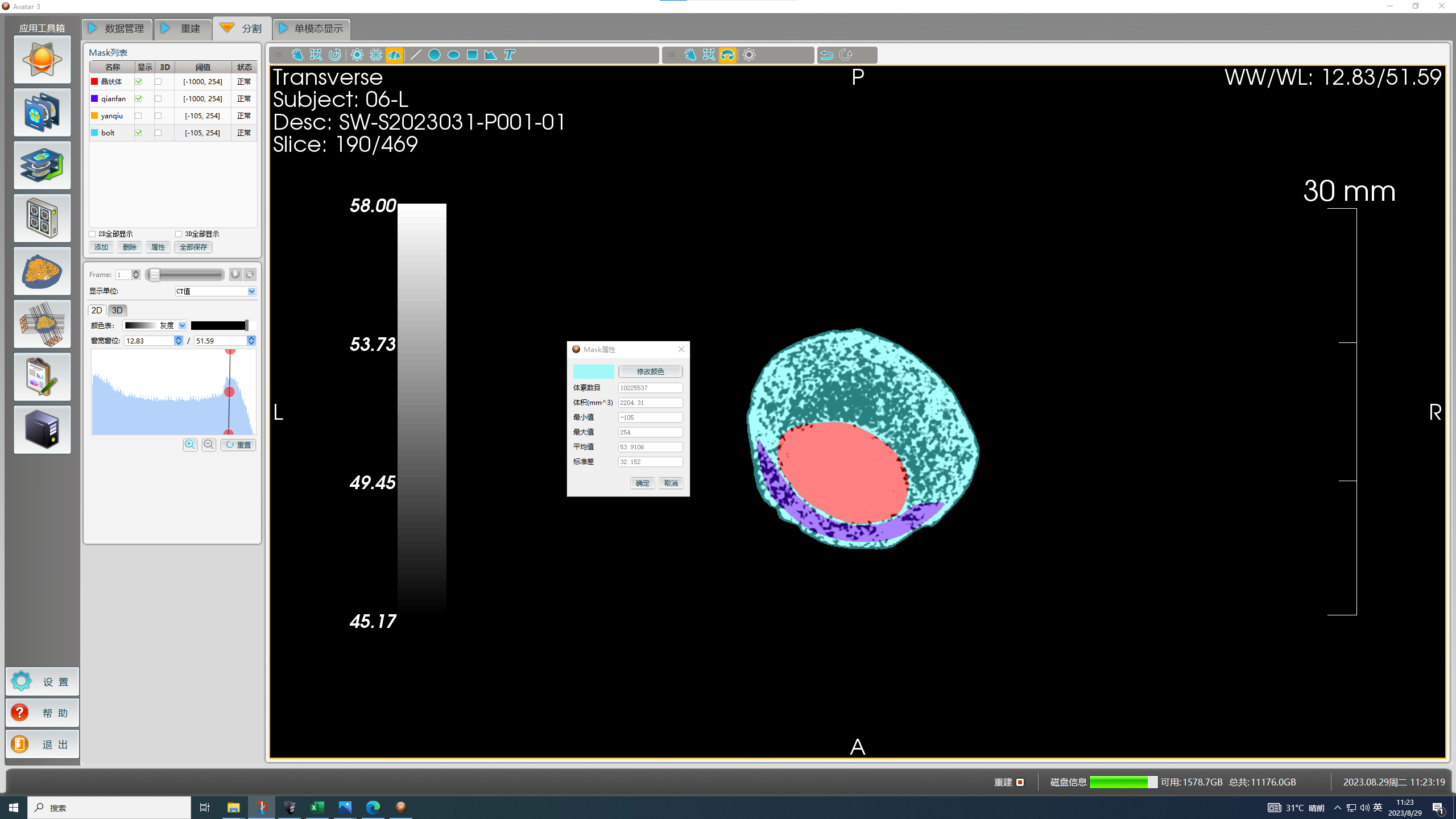

Supplement: S3 Data — (ZIP) [file pone.0310830.s003.zip › CT_rabbits/Vitreous body/06-L.png]

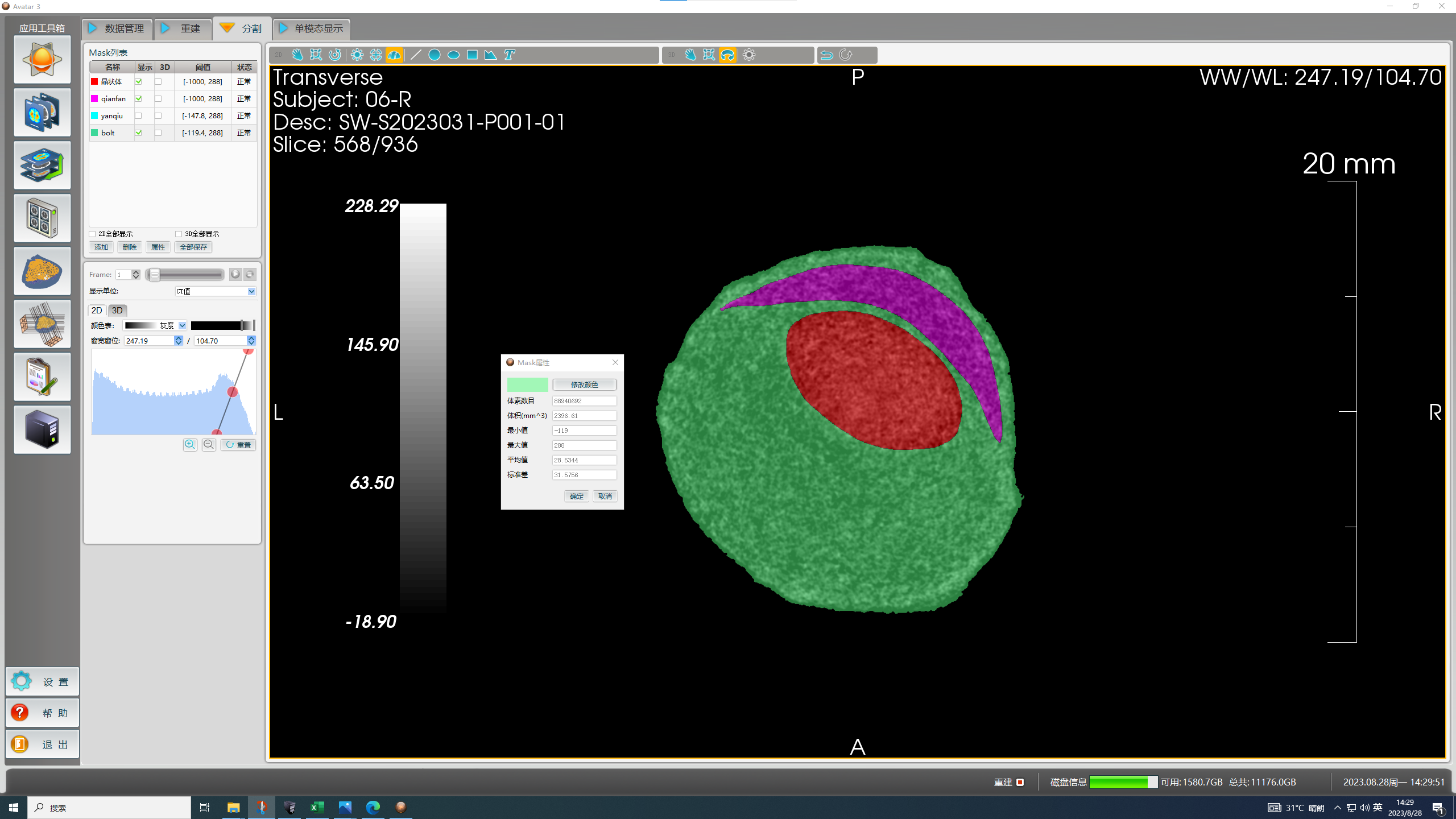

Supplement: S3 Data — (ZIP) [file pone.0310830.s003.zip › CT_rabbits/Vitreous body/06-R.png]

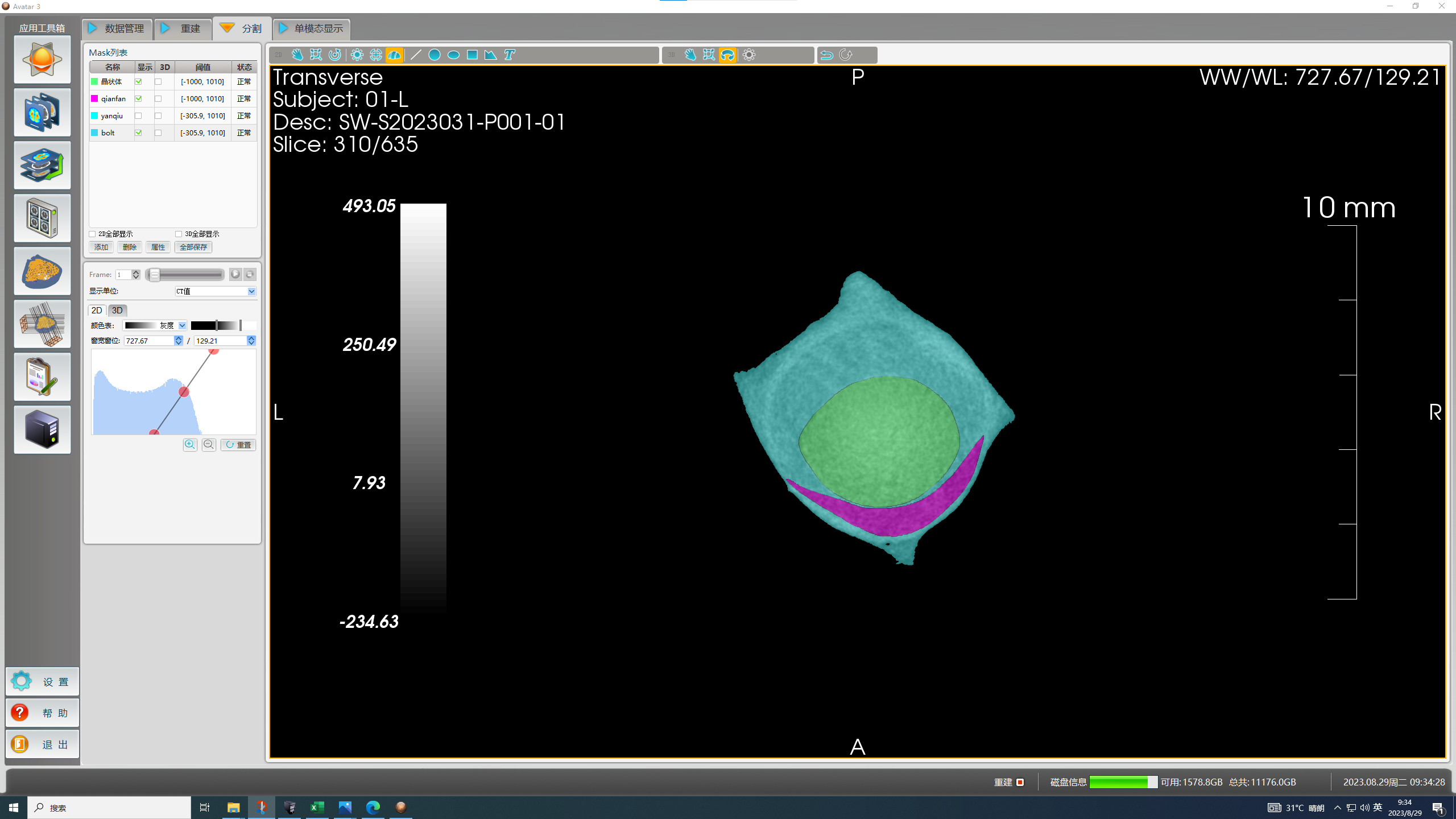

Supplement: S4 Data — (ZIP) [file pone.0310830.s004.zip › CT_SDrats/01-L.png]

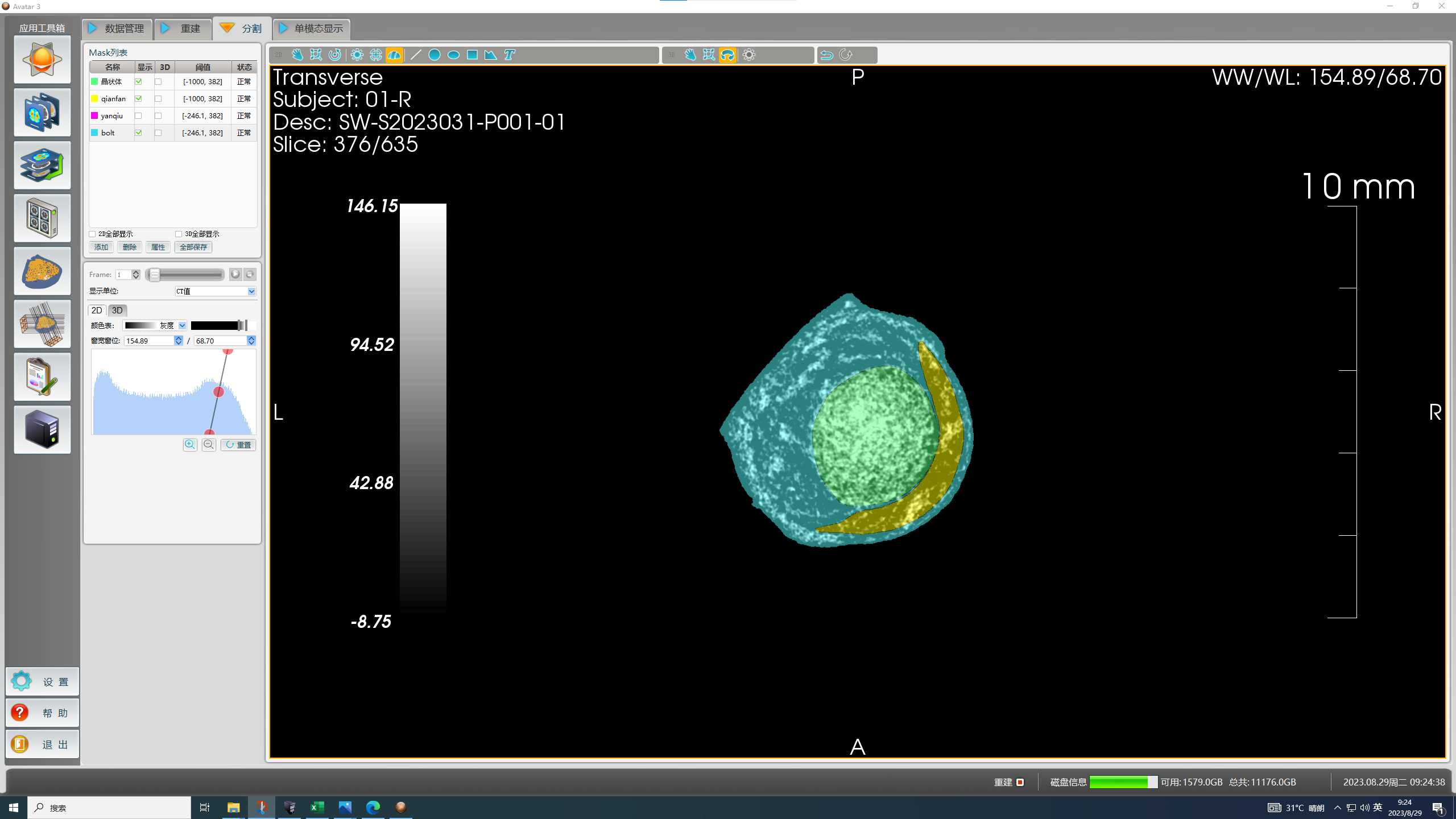

Supplement: S4 Data — (ZIP) [file pone.0310830.s004.zip › CT_SDrats/01-R.png]

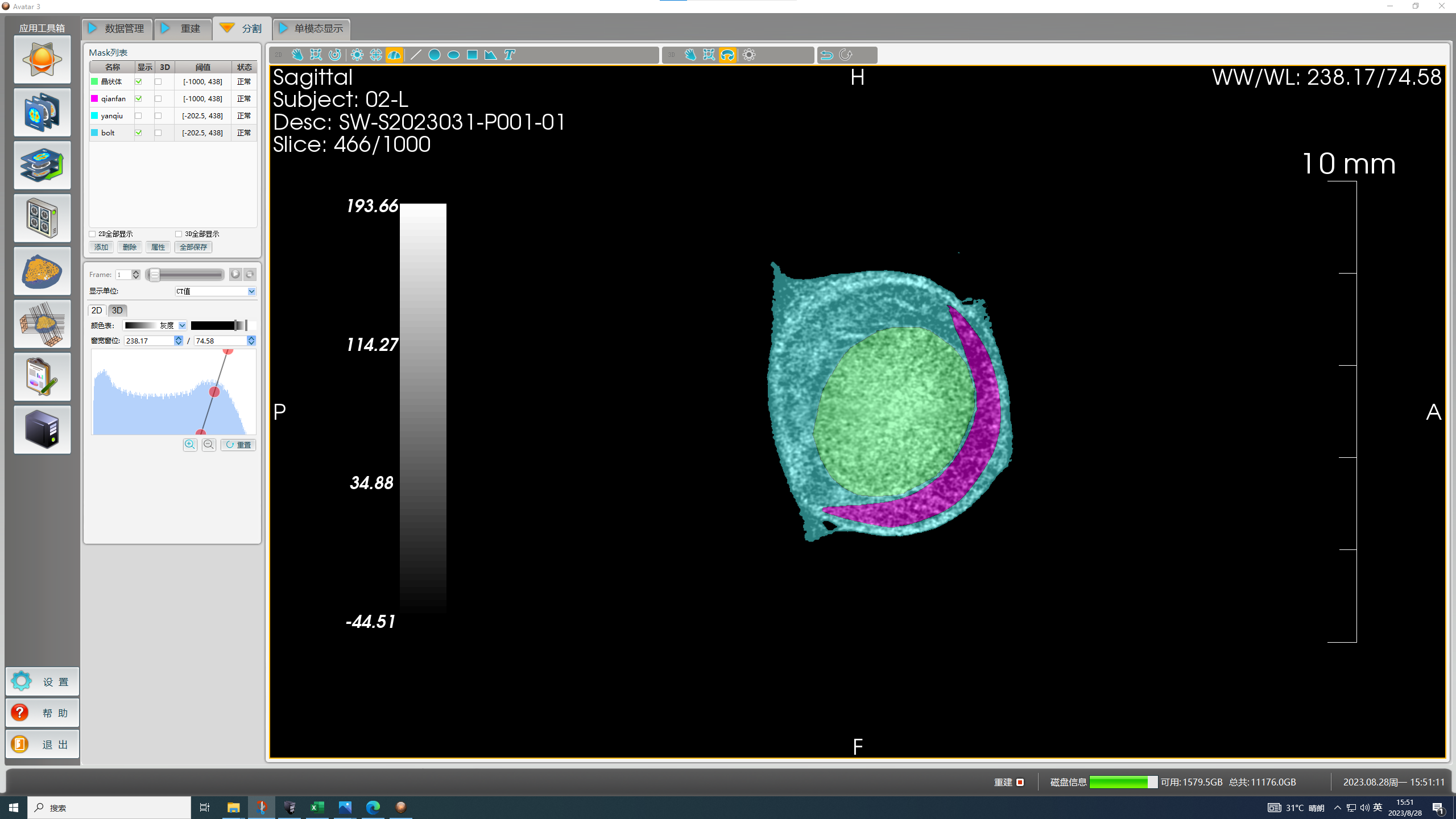

Supplement: S4 Data — (ZIP) [file pone.0310830.s004.zip › CT_SDrats/02-L.png]

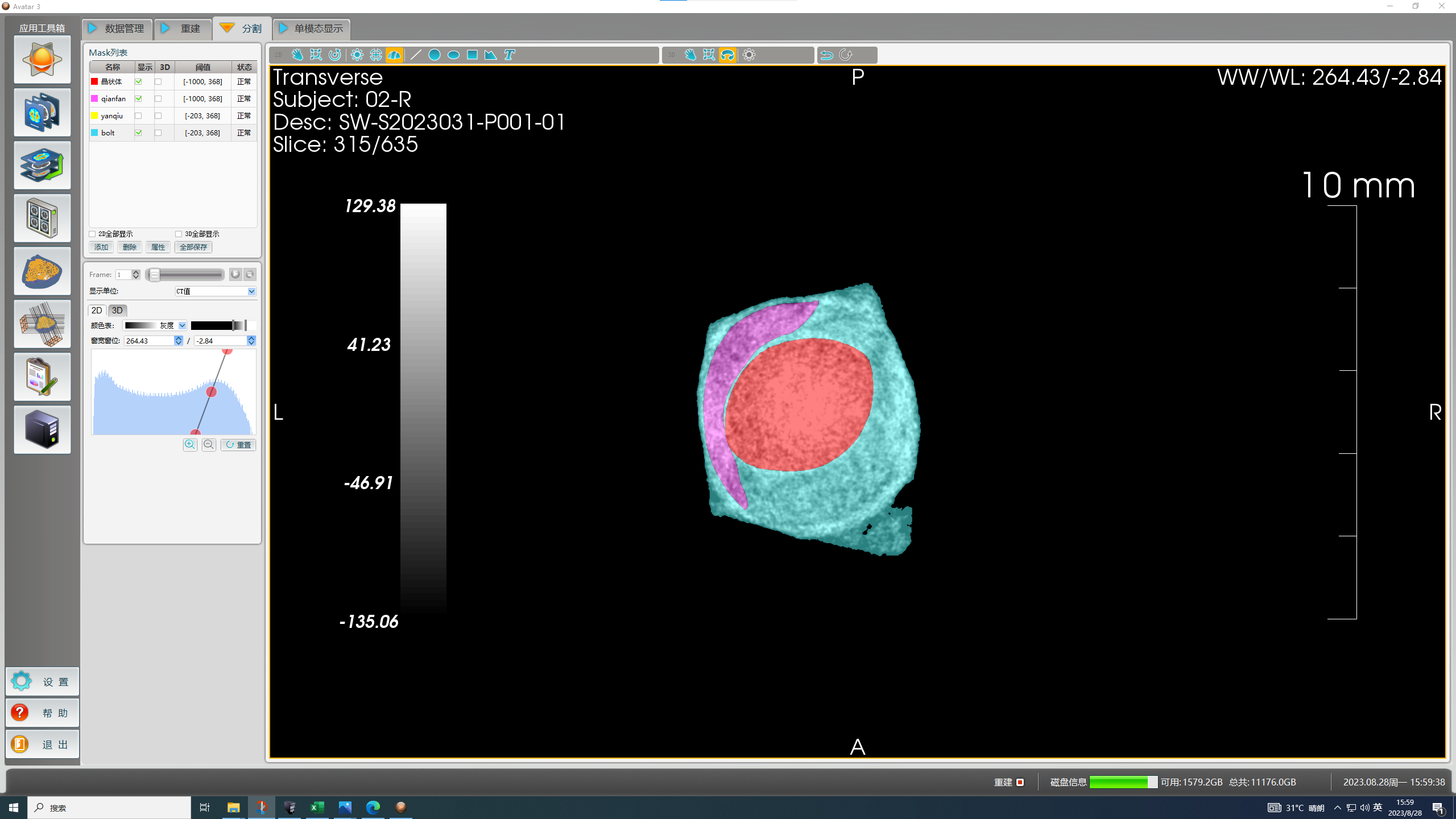

Supplement: S4 Data — (ZIP) [file pone.0310830.s004.zip › CT_SDrats/02-R.png]

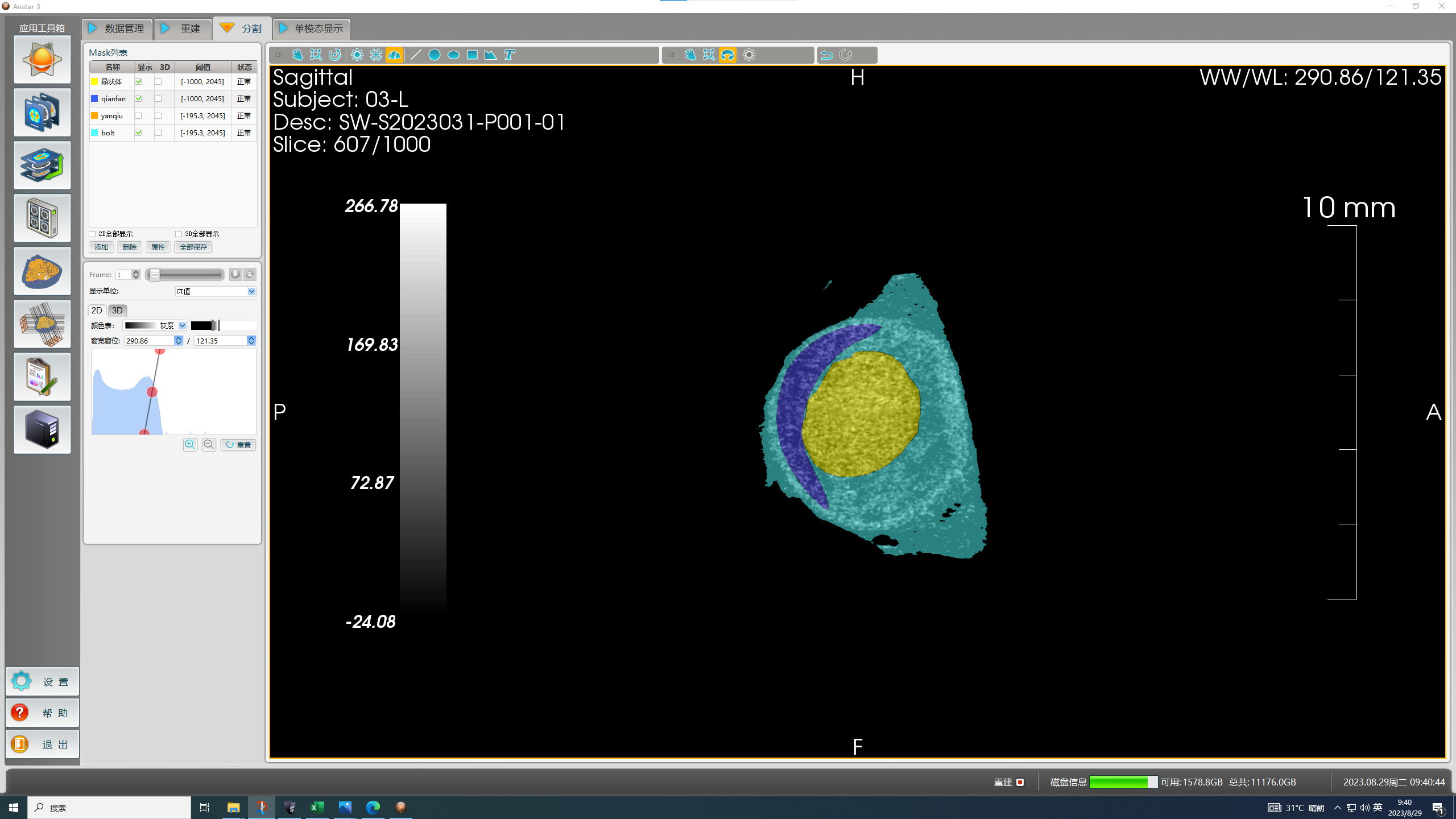

Supplement: S4 Data — (ZIP) [file pone.0310830.s004.zip › CT_SDrats/03-L.png]

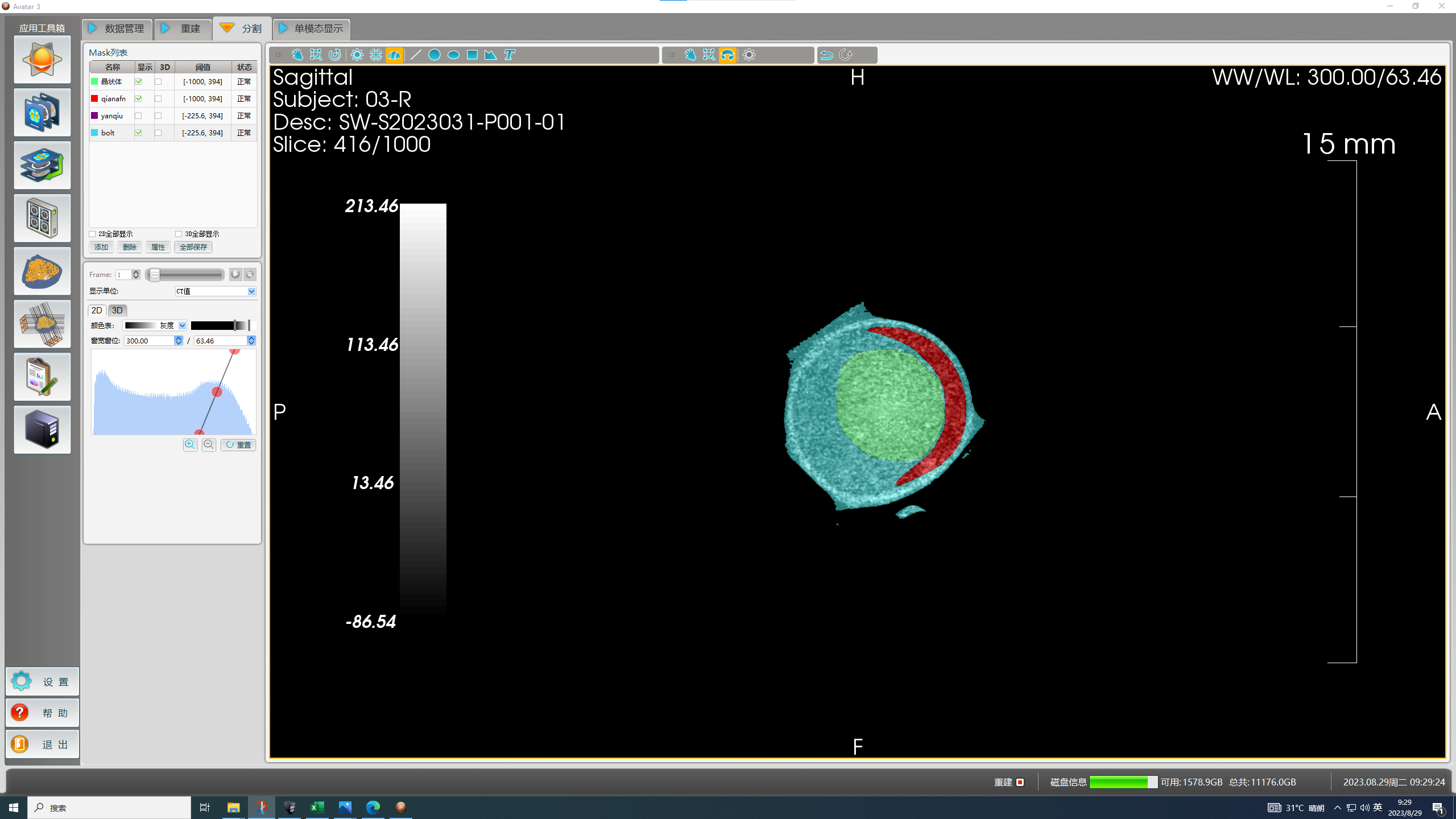

Supplement: S4 Data — (ZIP) [file pone.0310830.s004.zip › CT_SDrats/03-R.png]

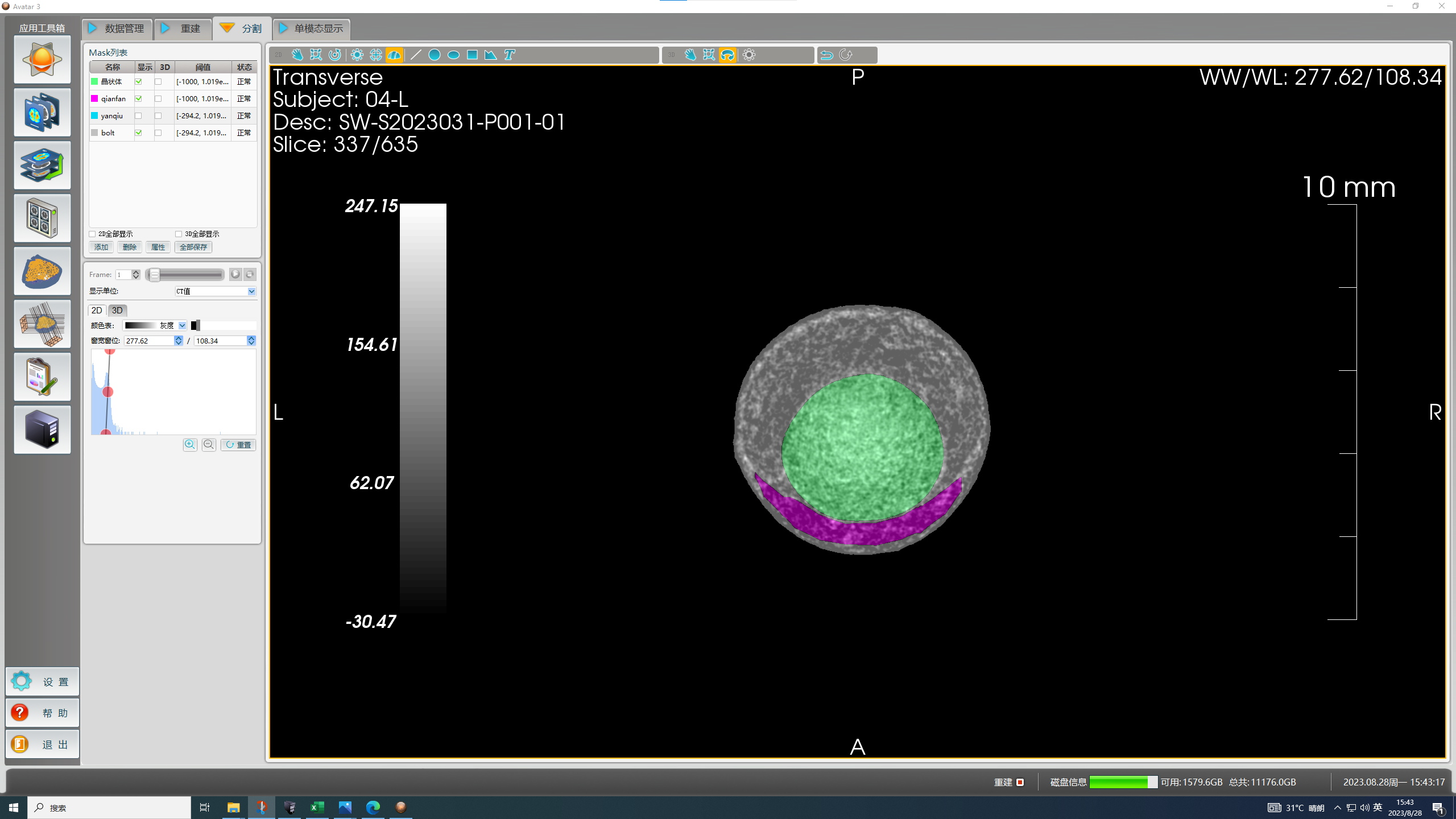

Supplement: S4 Data — (ZIP) [file pone.0310830.s004.zip › CT_SDrats/04-L.png]

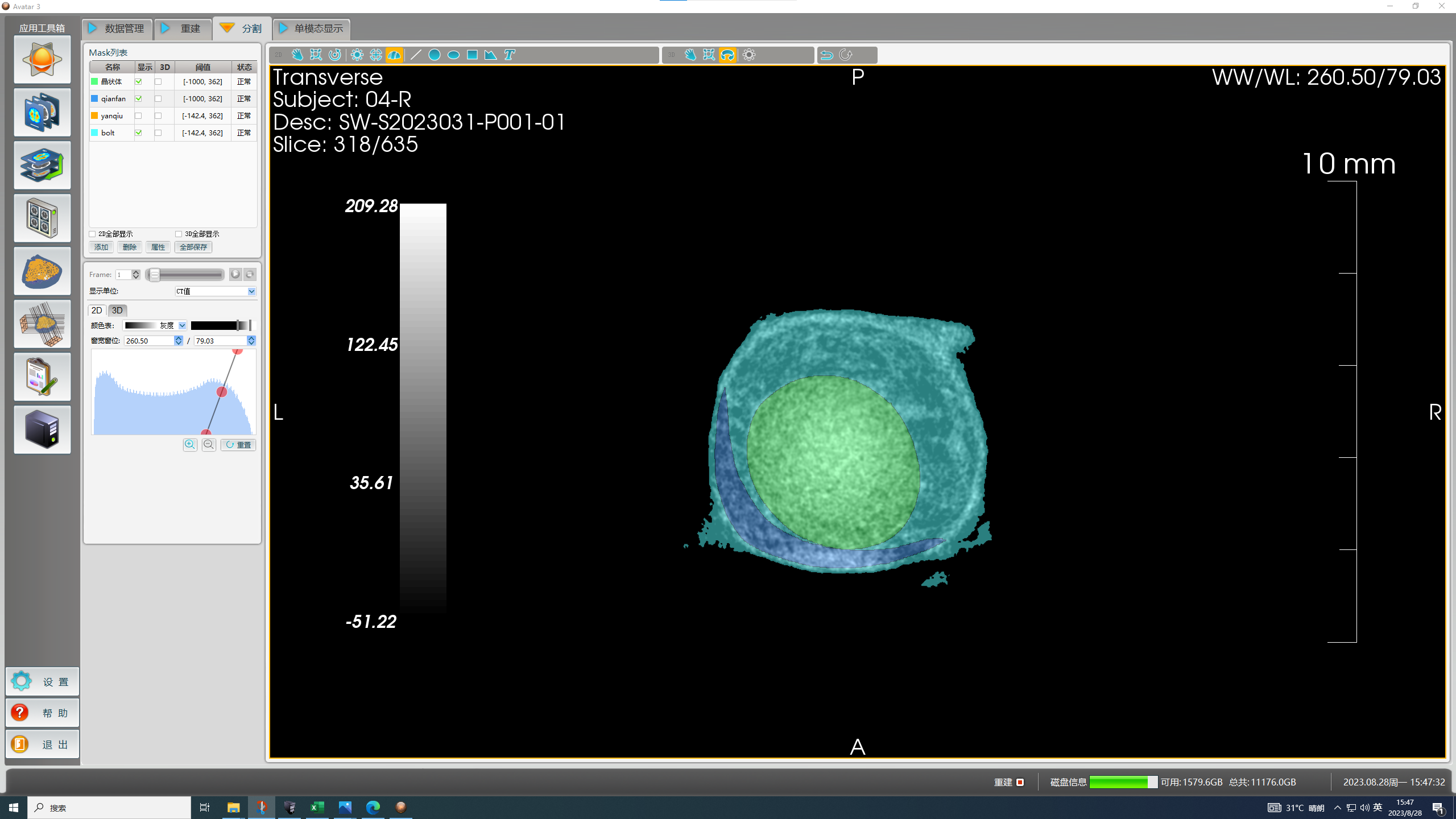

Supplement: S4 Data — (ZIP) [file pone.0310830.s004.zip › CT_SDrats/04-R.png]

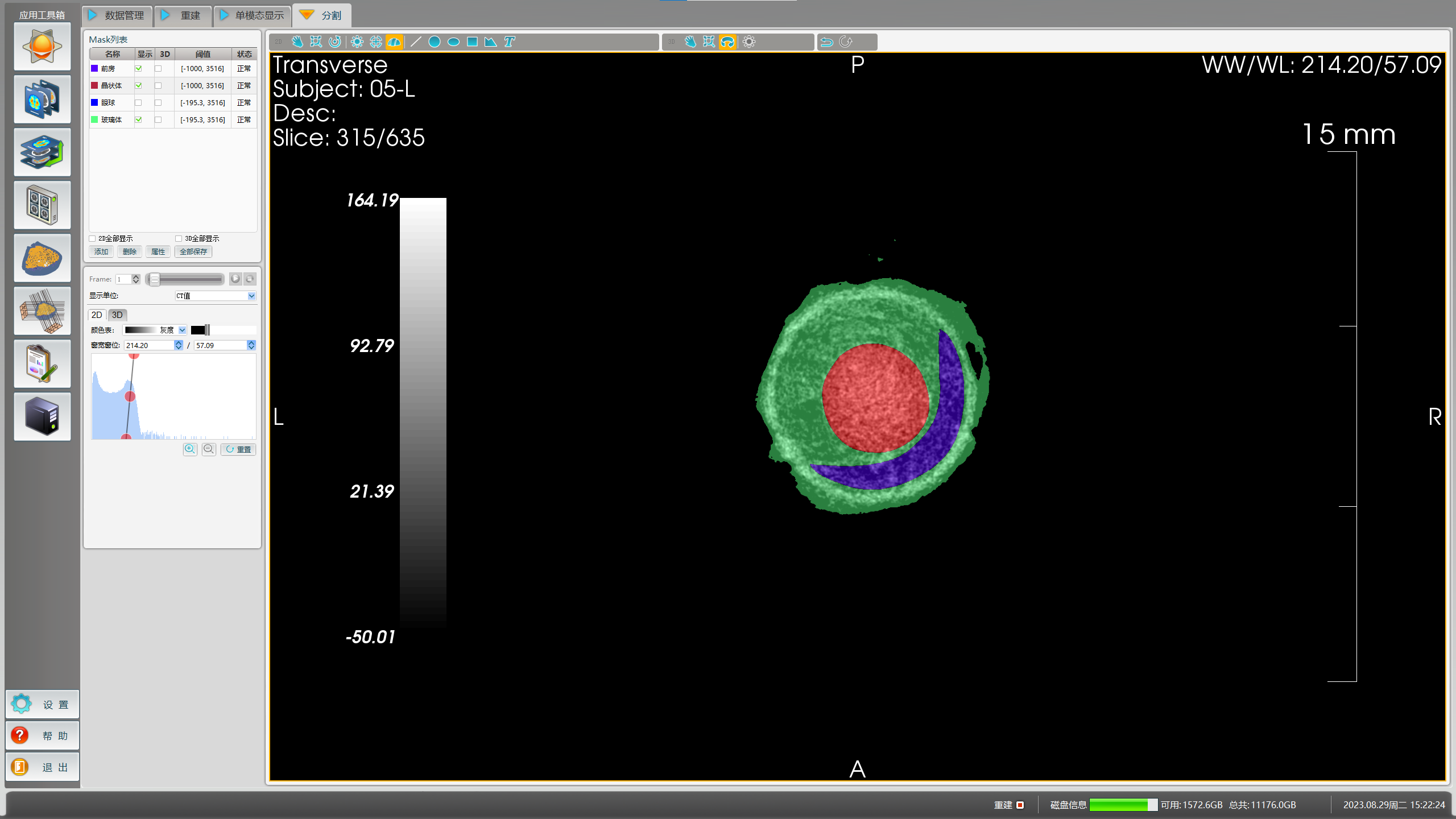

Supplement: S4 Data — (ZIP) [file pone.0310830.s004.zip › CT_SDrats/05-L.png]

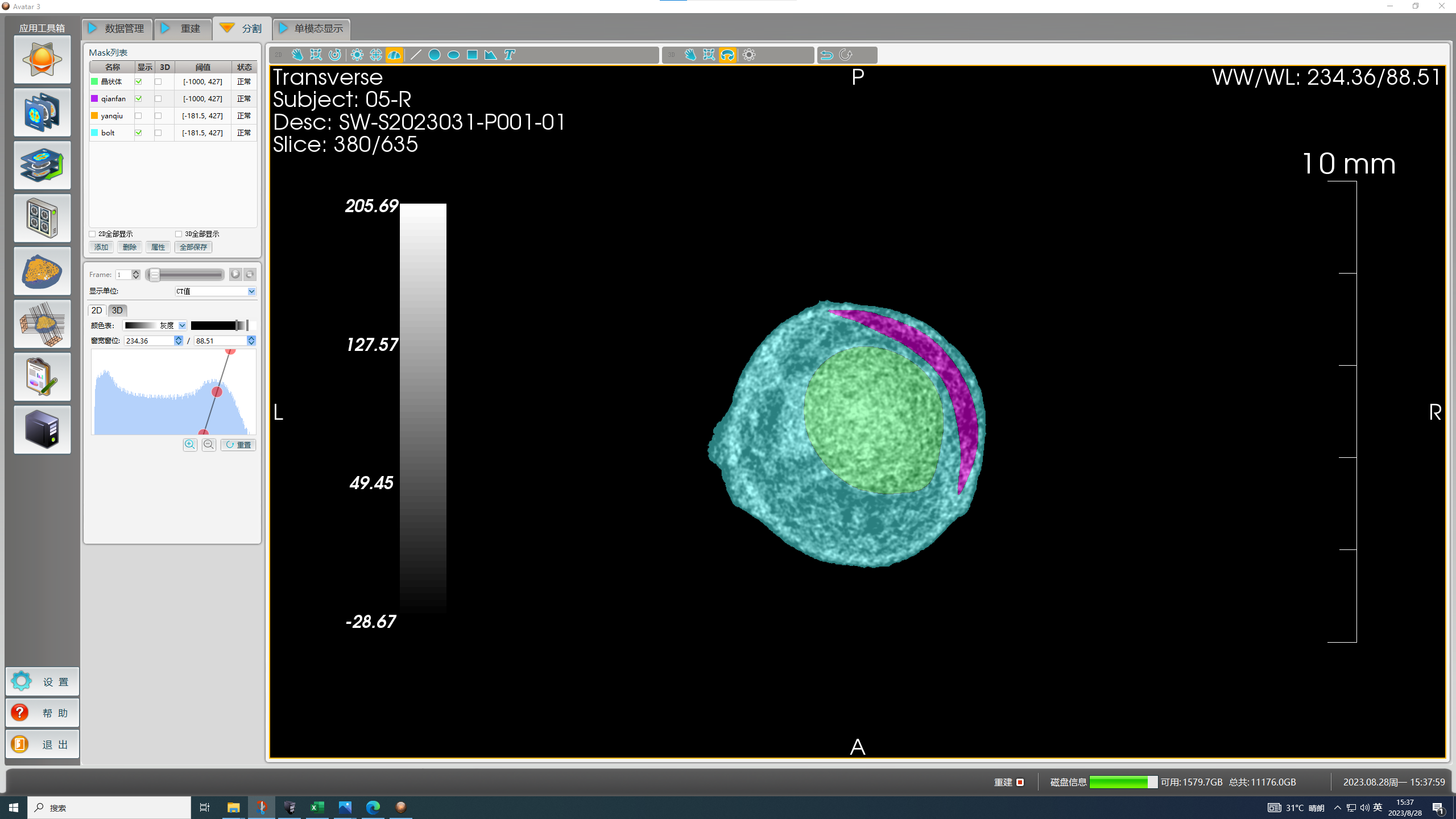

Supplement: S4 Data — (ZIP) [file pone.0310830.s004.zip › CT_SDrats/05-R.png]

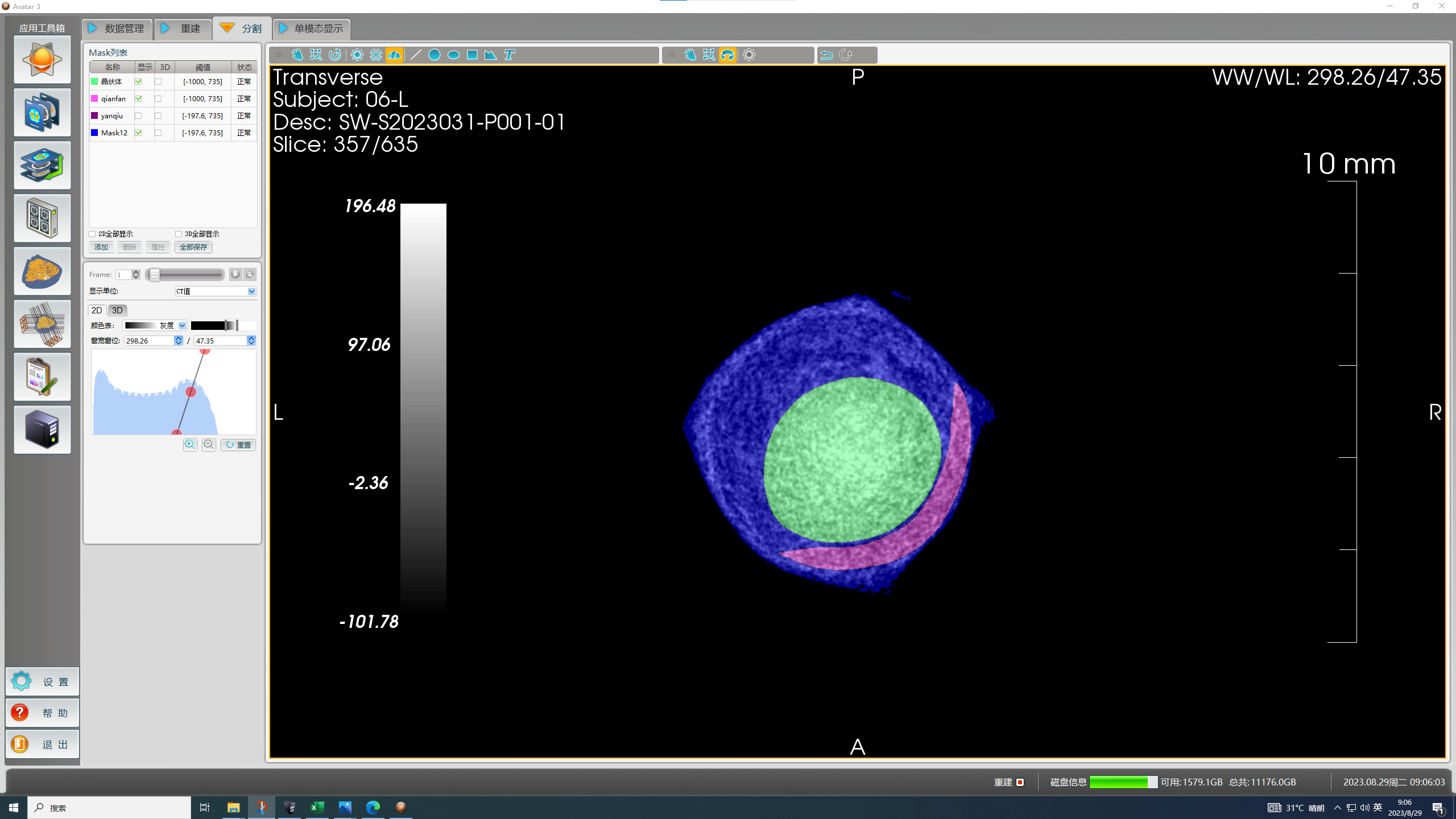

Supplement: S4 Data — (ZIP) [file pone.0310830.s004.zip › CT_SDrats/06-L.png]

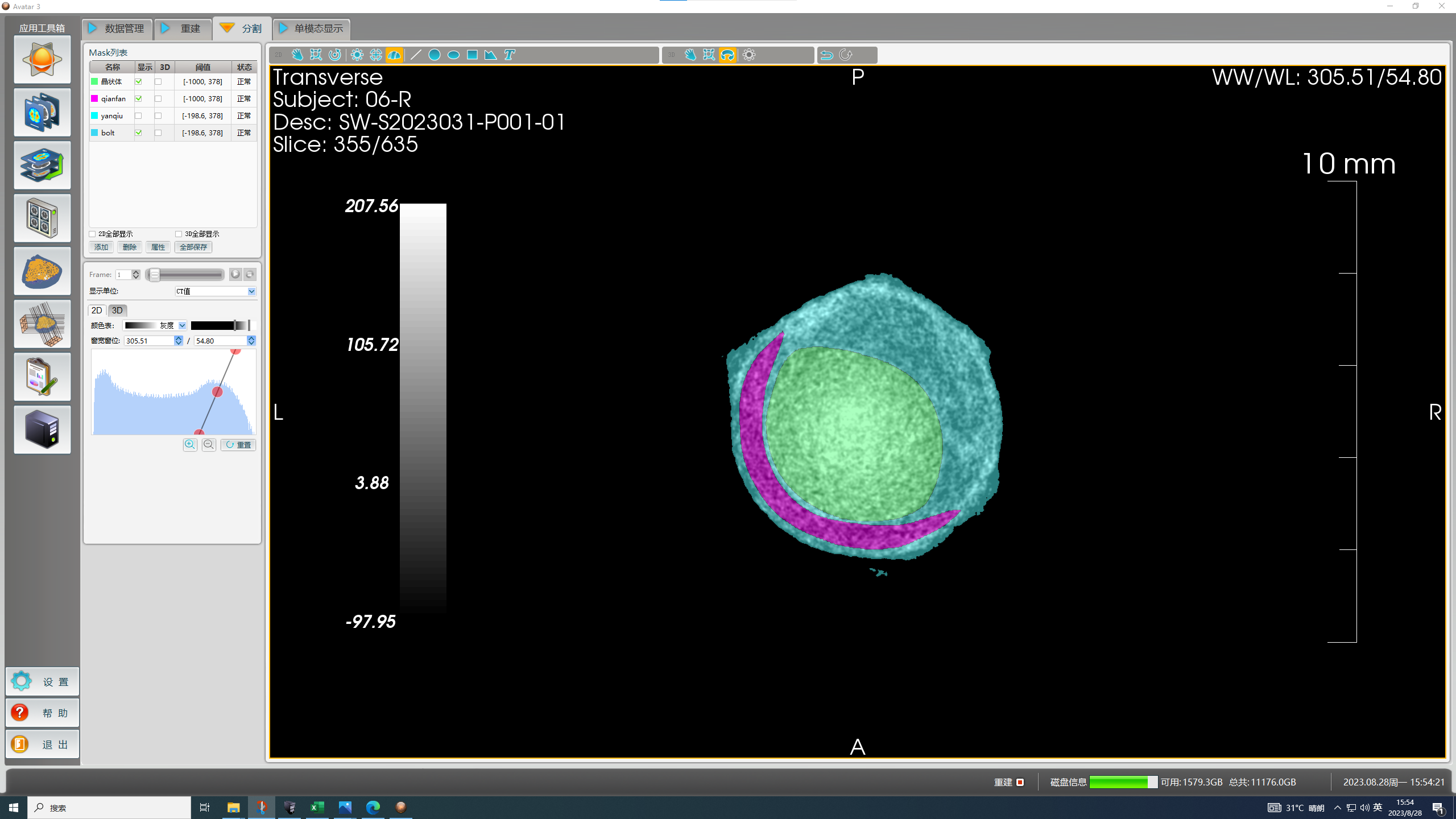

Supplement: S4 Data — (ZIP) [file pone.0310830.s004.zip › CT_SDrats/06-R.png]

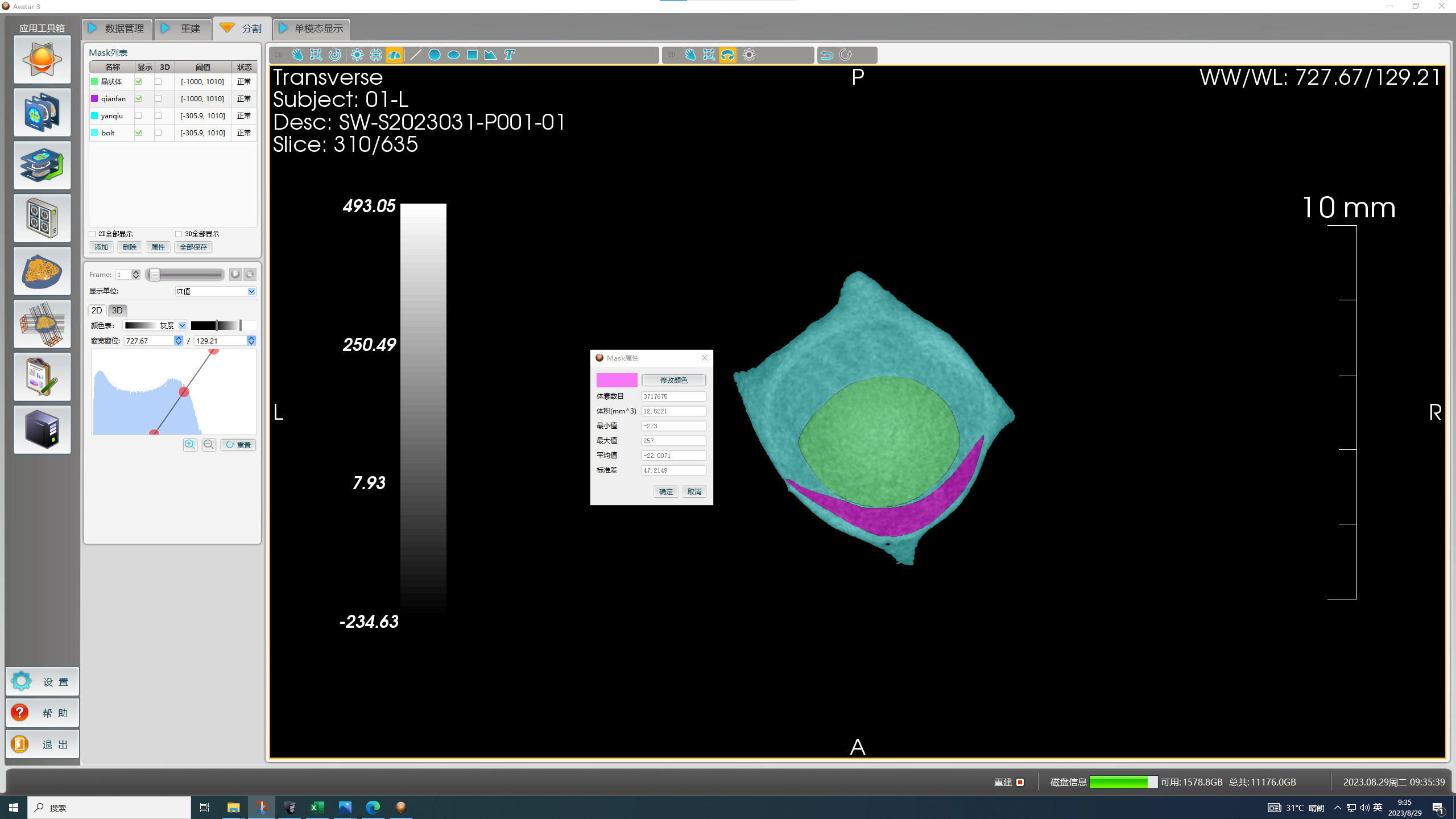

Supplement: S4 Data — (ZIP) [file pone.0310830.s004.zip › CT_SDrats/Anterior chamber/01-L.png]

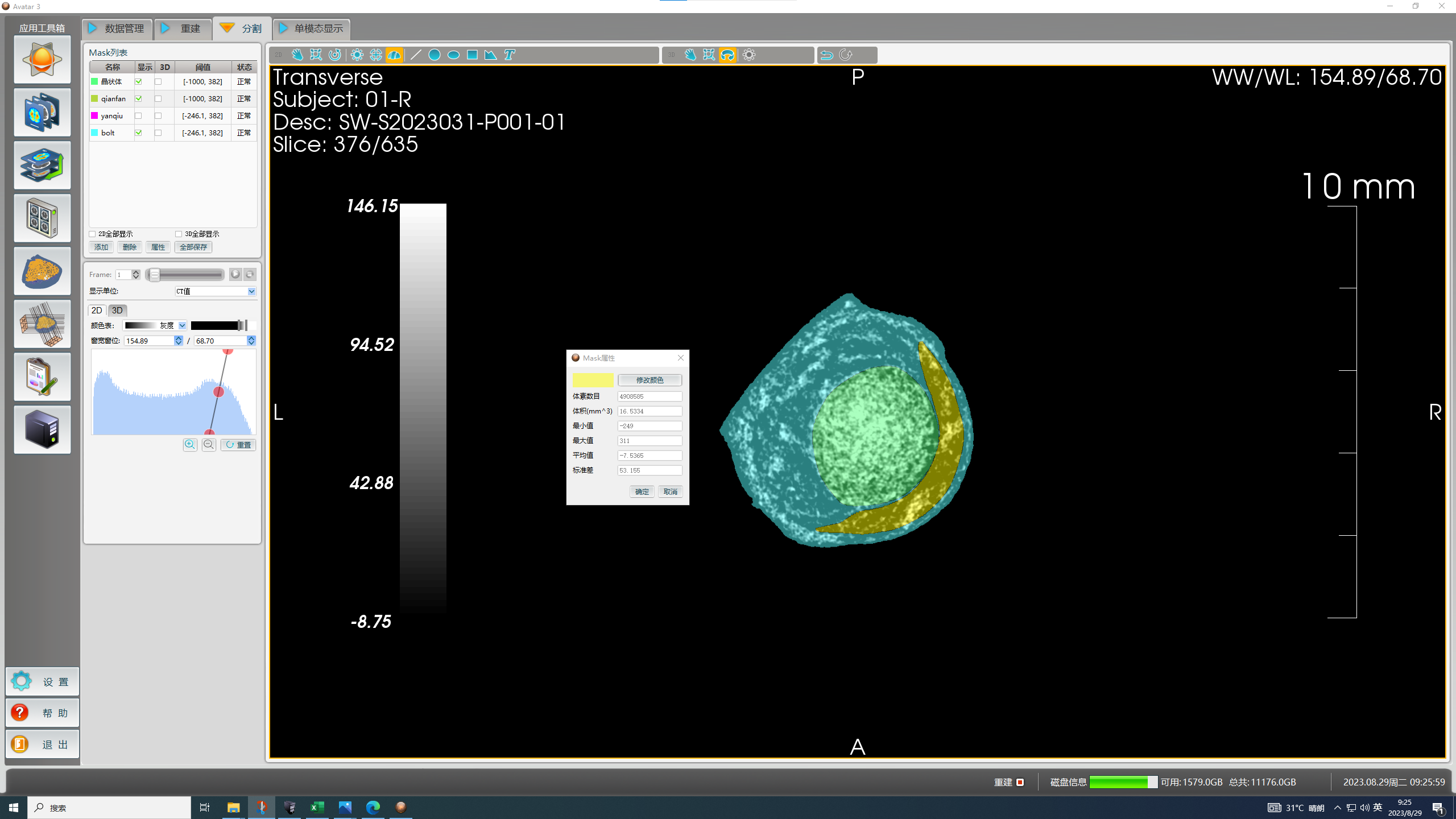

Supplement: S4 Data — (ZIP) [file pone.0310830.s004.zip › CT_SDrats/Anterior chamber/01-R.png]

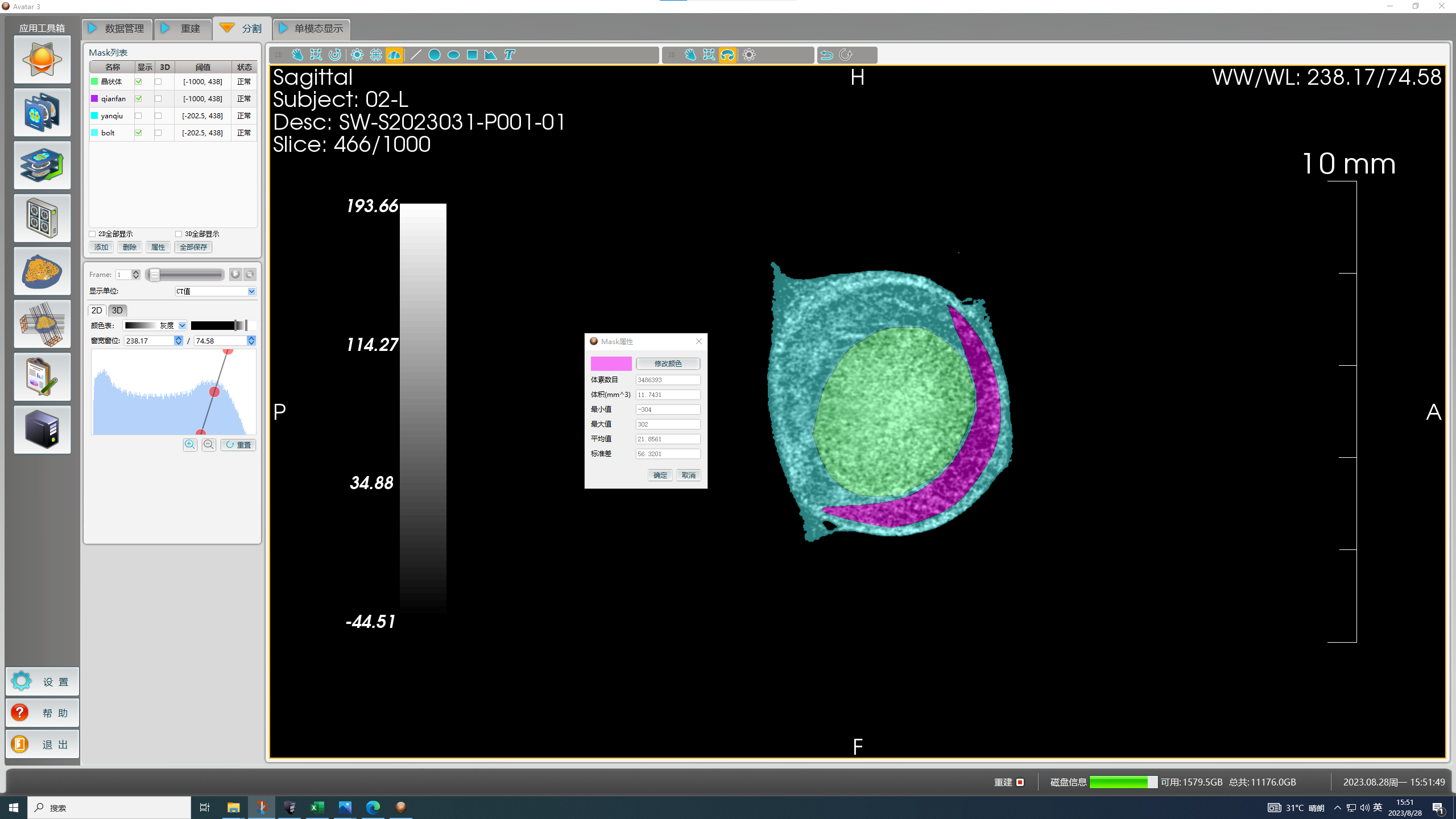

Supplement: S4 Data — (ZIP) [file pone.0310830.s004.zip › CT_SDrats/Anterior chamber/02-L.png]

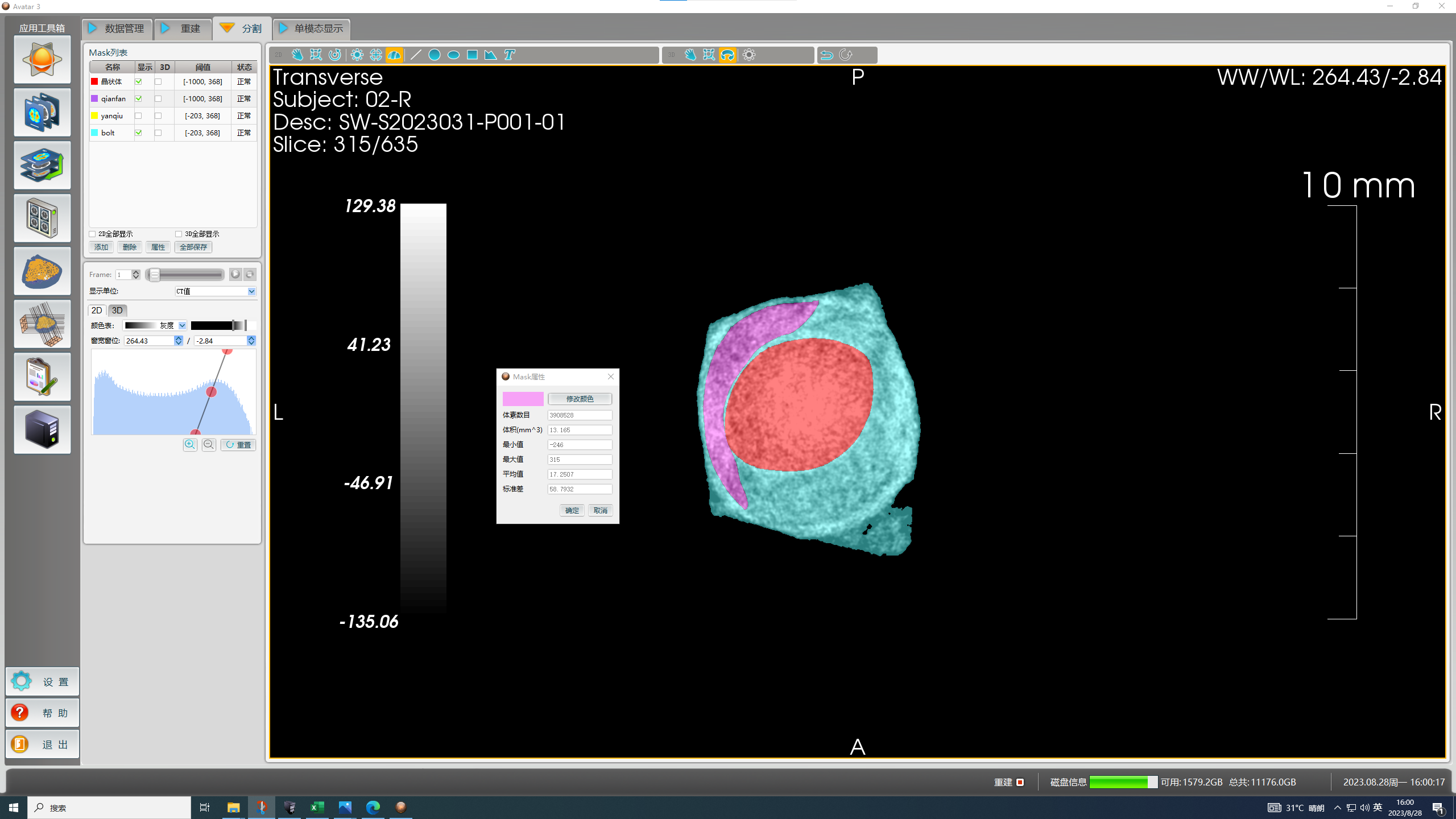

Supplement: S4 Data — (ZIP) [file pone.0310830.s004.zip › CT_SDrats/Anterior chamber/02-R.png]

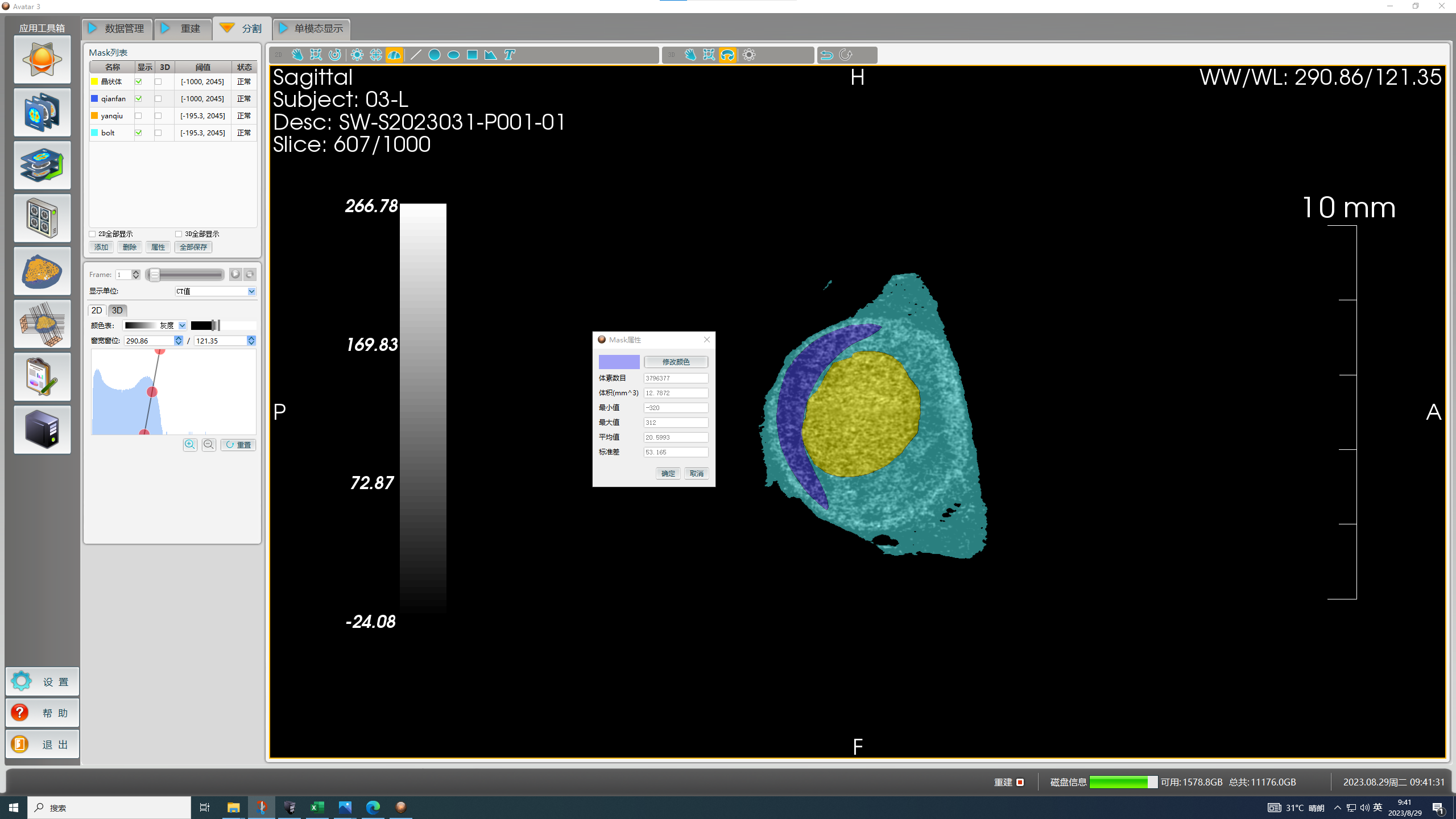

Supplement: S4 Data — (ZIP) [file pone.0310830.s004.zip › CT_SDrats/Anterior chamber/03-L.png]

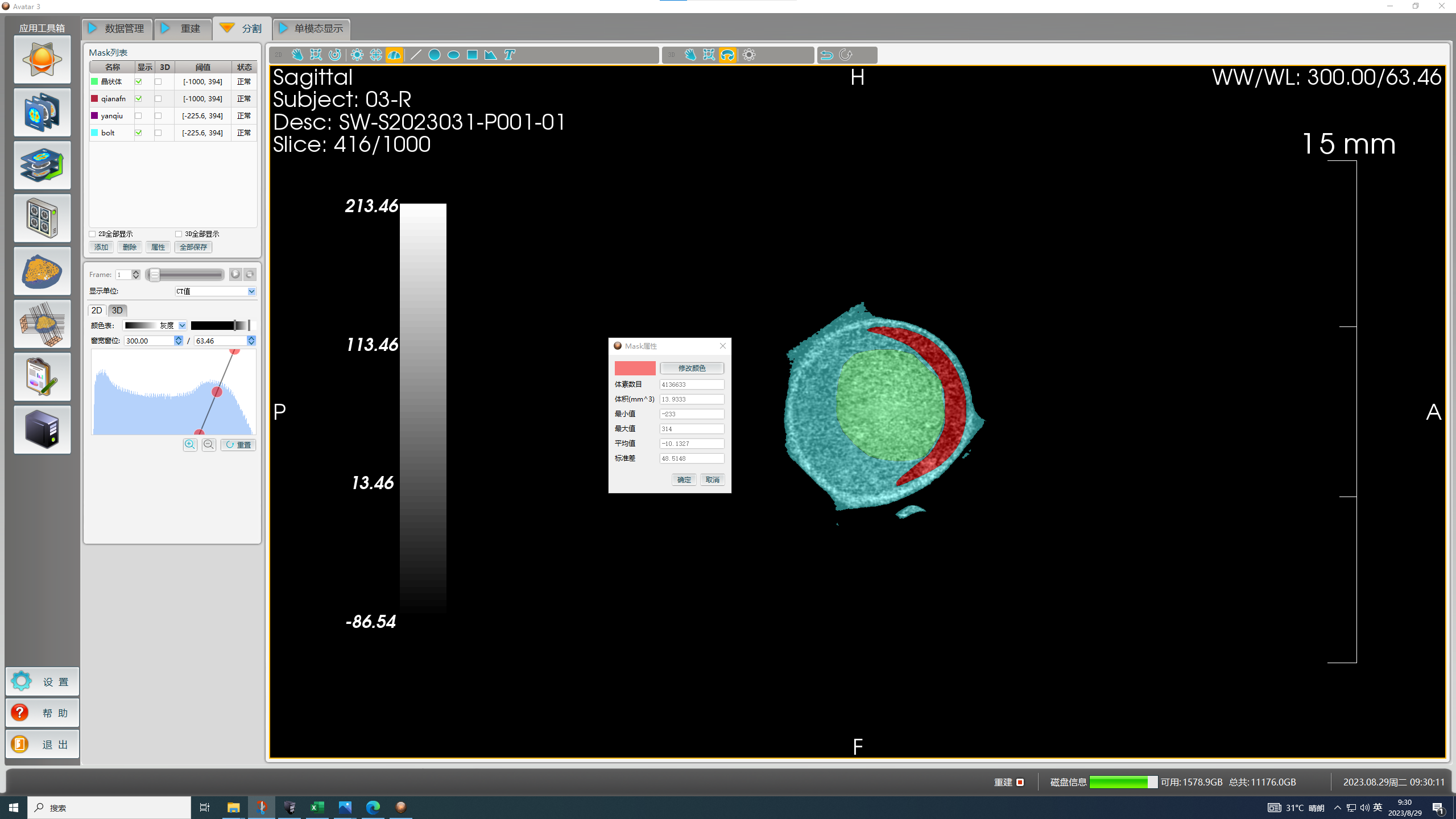

Supplement: S4 Data — (ZIP) [file pone.0310830.s004.zip › CT_SDrats/Anterior chamber/03-R.png]

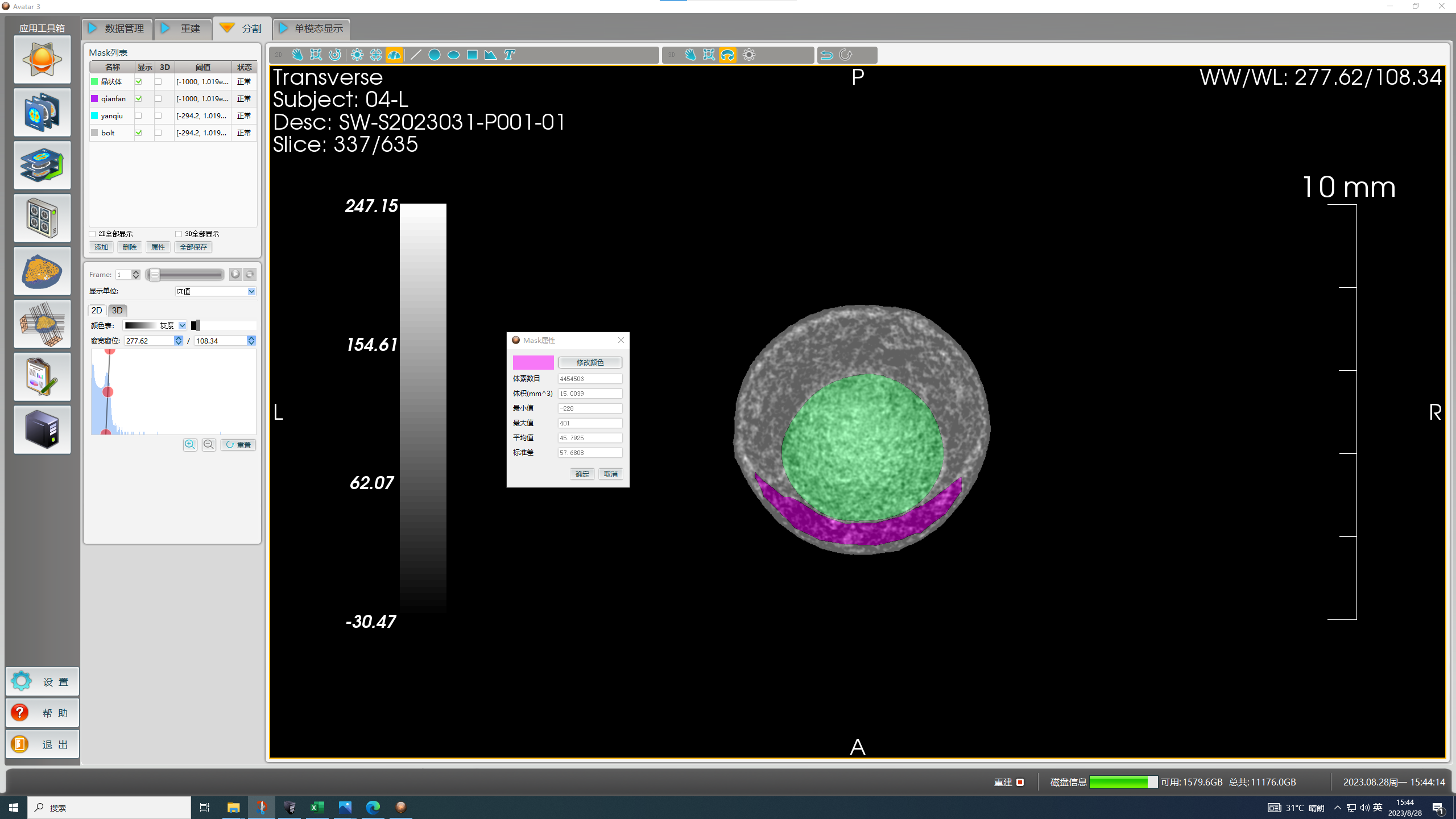

Supplement: S4 Data — (ZIP) [file pone.0310830.s004.zip › CT_SDrats/Anterior chamber/04-L.png]

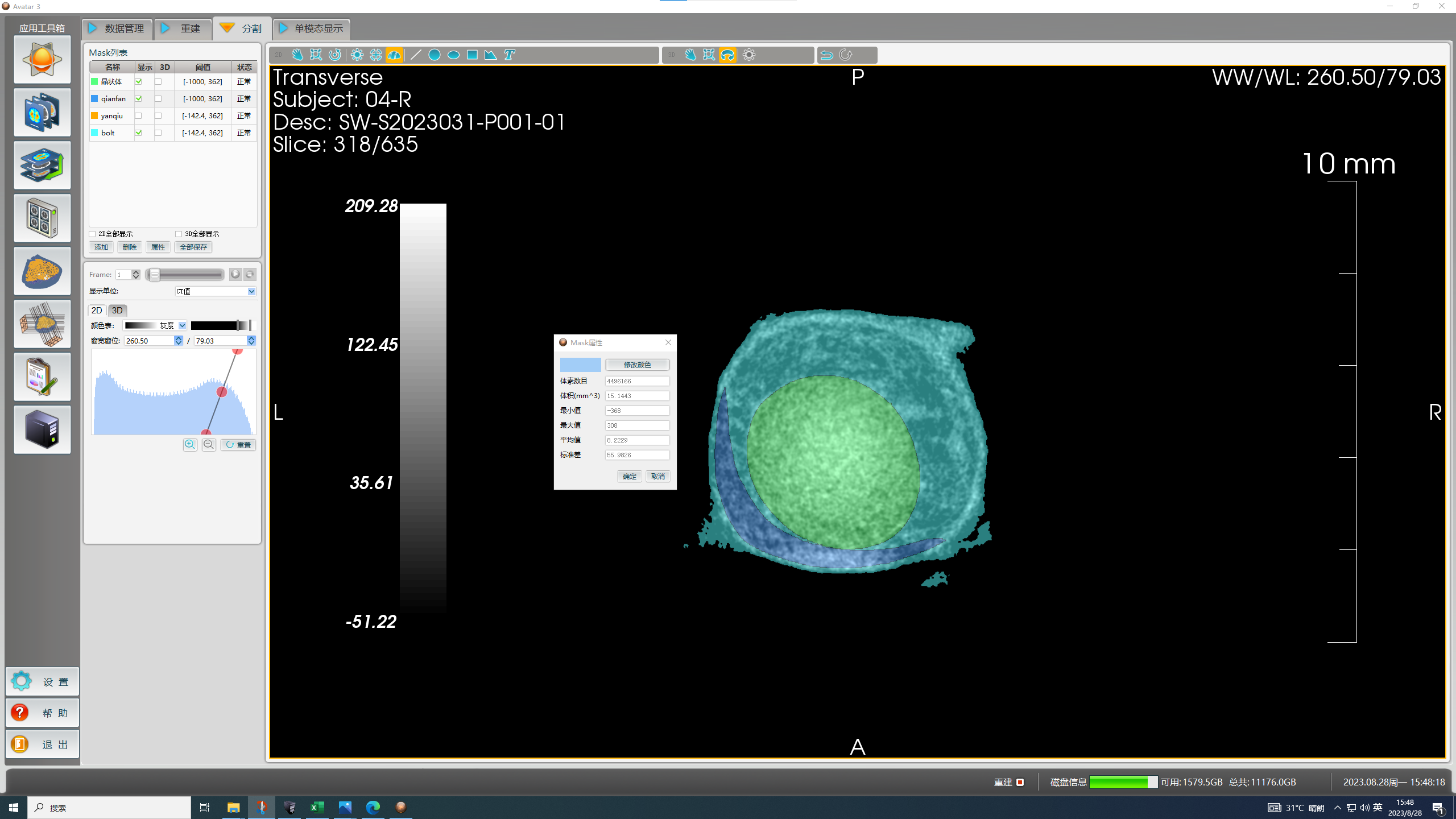

Supplement: S4 Data — (ZIP) [file pone.0310830.s004.zip › CT_SDrats/Anterior chamber/04-R.png]

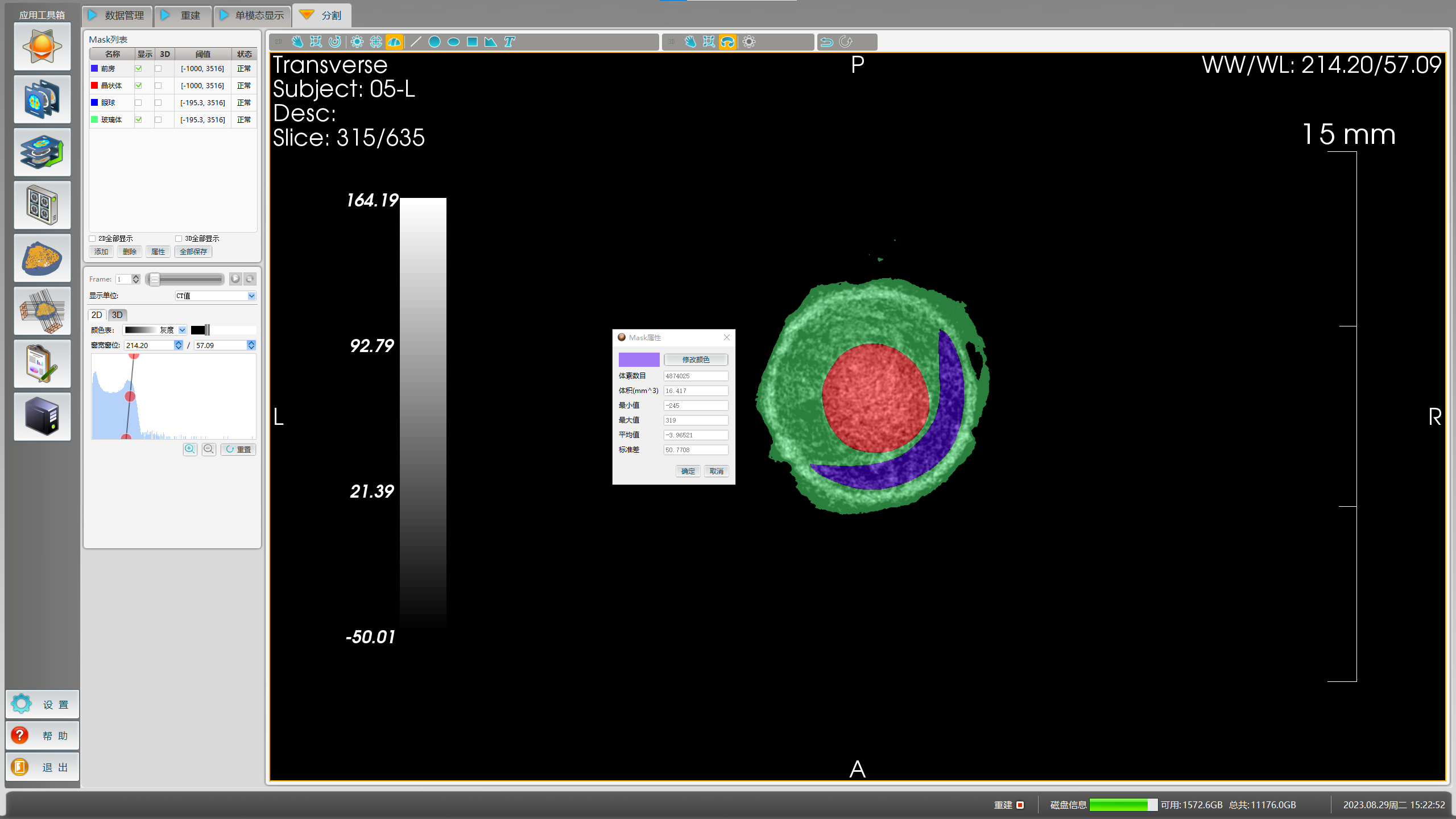

Supplement: S4 Data — (ZIP) [file pone.0310830.s004.zip › CT_SDrats/Anterior chamber/05-L.png]

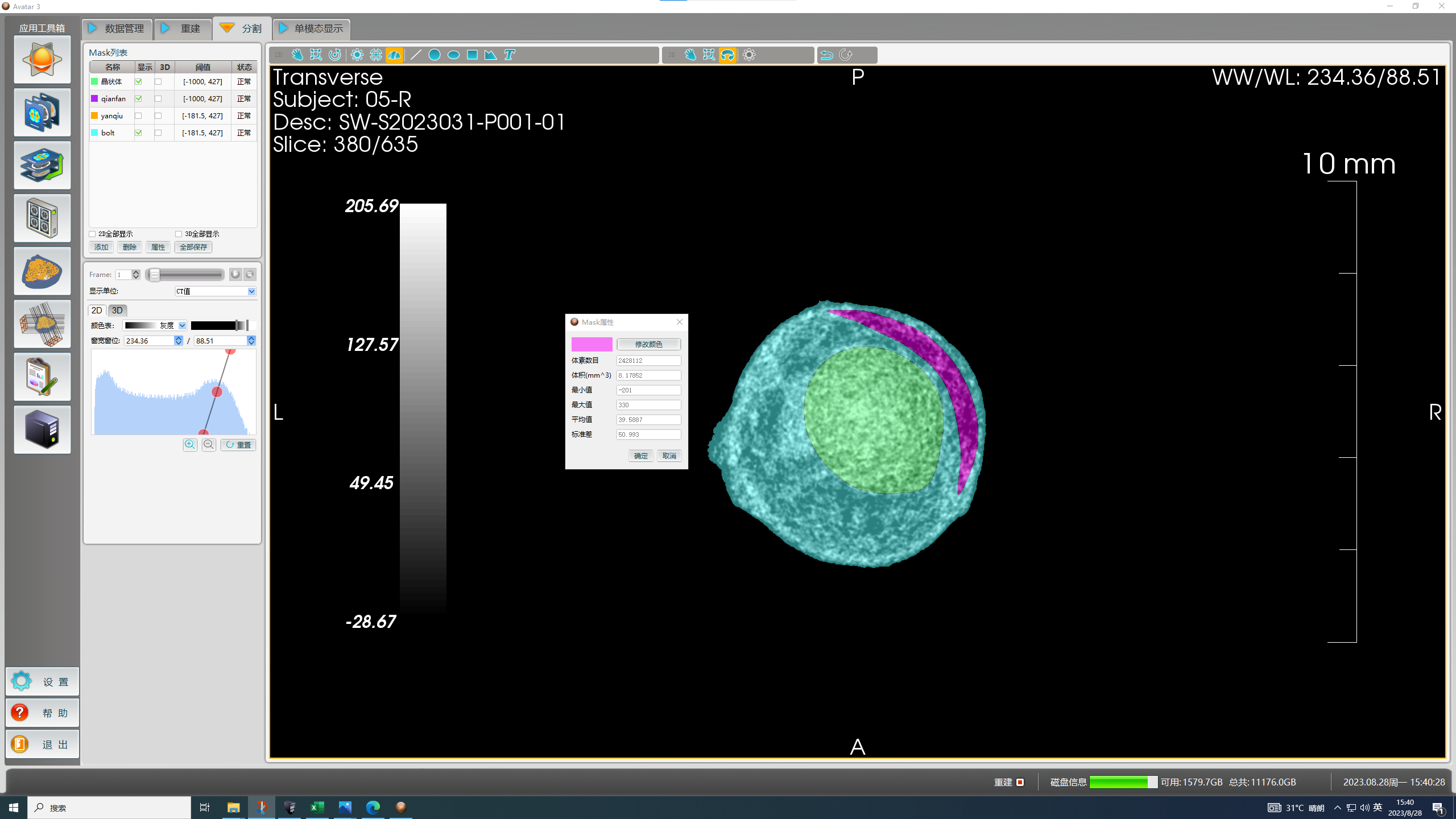

Supplement: S4 Data — (ZIP) [file pone.0310830.s004.zip › CT_SDrats/Anterior chamber/05-R.png]

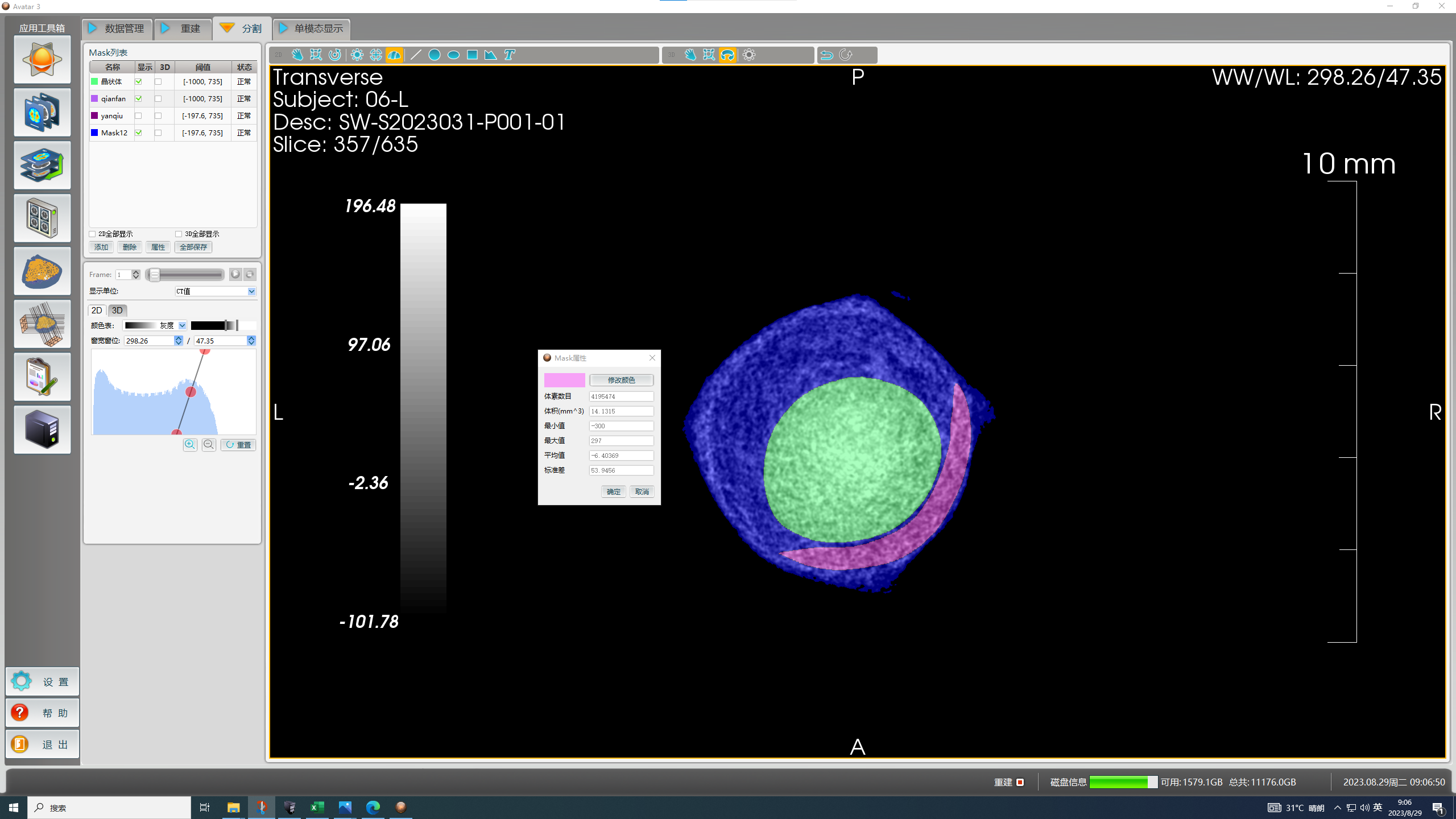

Supplement: S4 Data — (ZIP) [file pone.0310830.s004.zip › CT_SDrats/Anterior chamber/06-L.png]

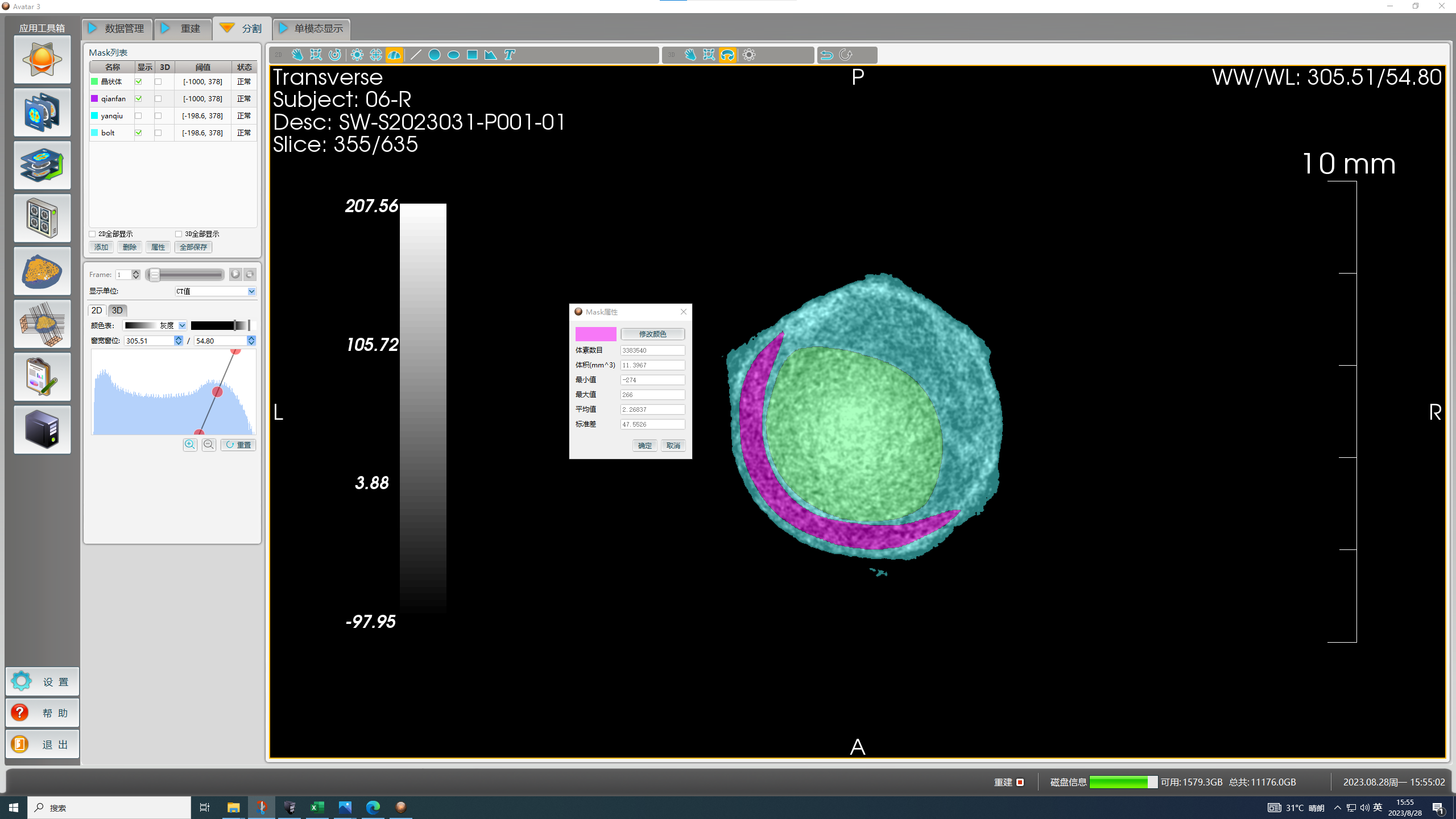

Supplement: S4 Data — (ZIP) [file pone.0310830.s004.zip › CT_SDrats/Anterior chamber/06-R.png]

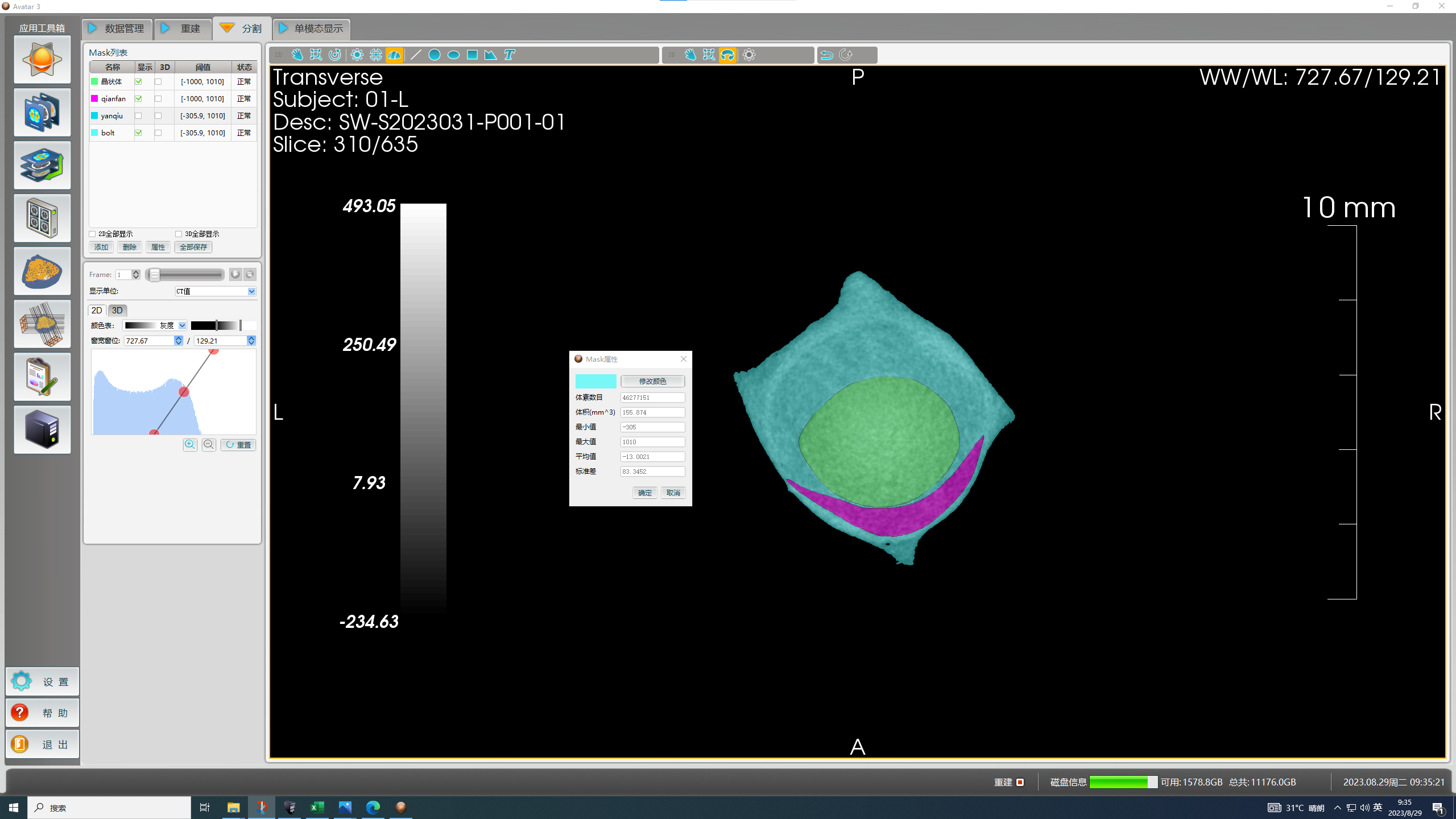

Supplement: S4 Data — (ZIP) [file pone.0310830.s004.zip › CT_SDrats/Eyeball volume/01-L.png]

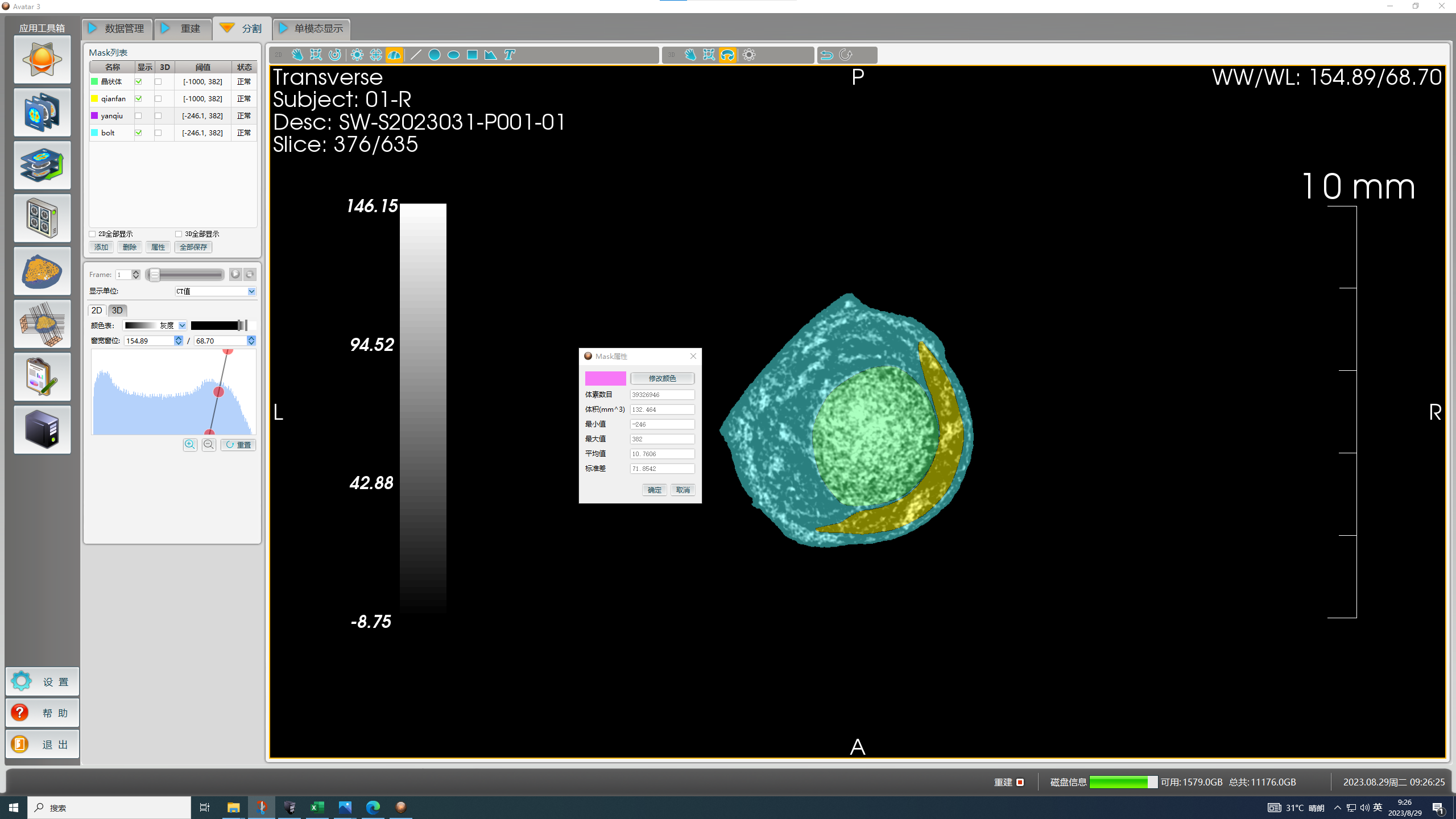

Supplement: S4 Data — (ZIP) [file pone.0310830.s004.zip › CT_SDrats/Eyeball volume/01-R.png]

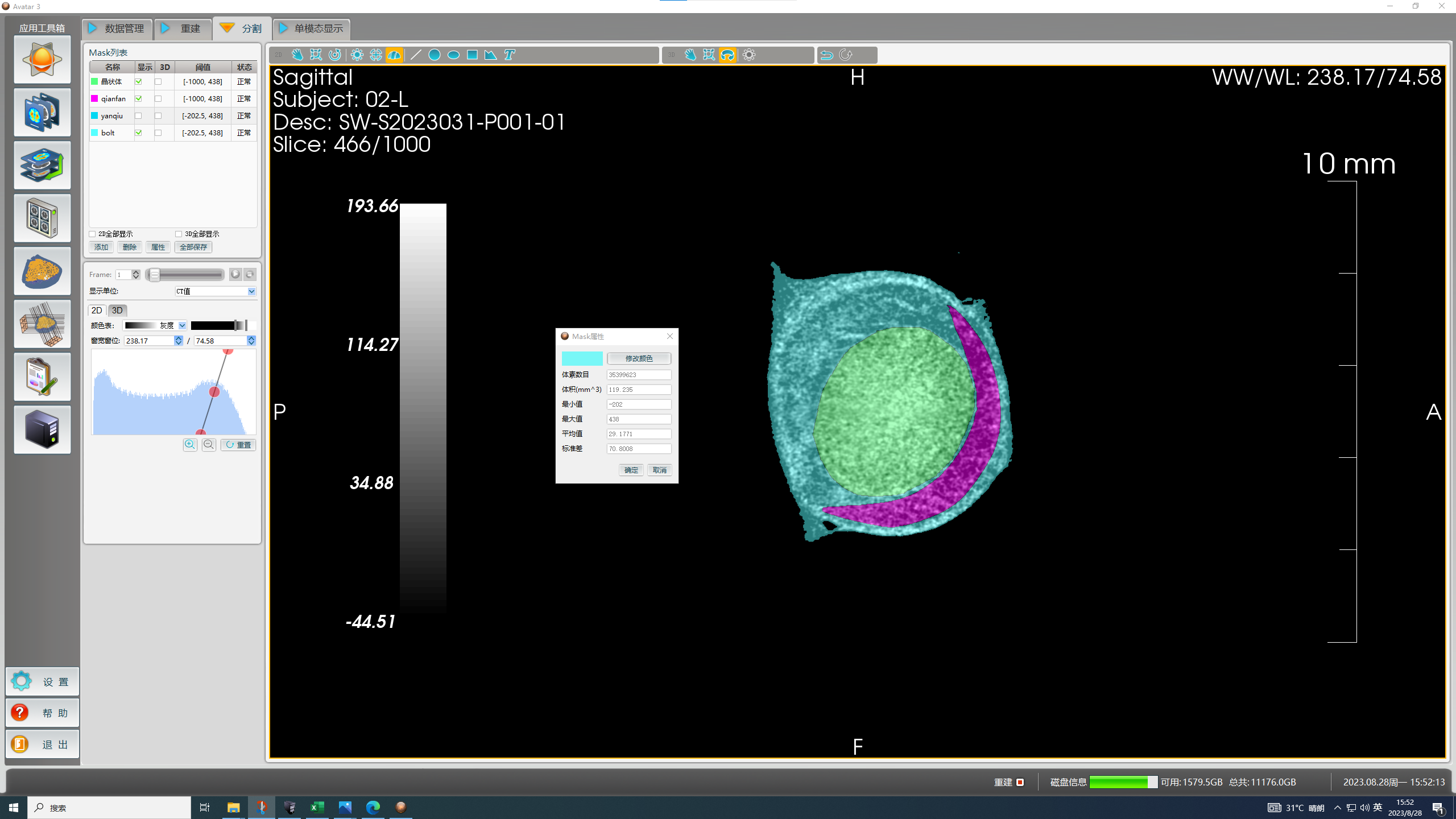

Supplement: S4 Data — (ZIP) [file pone.0310830.s004.zip › CT_SDrats/Eyeball volume/02-L.png]

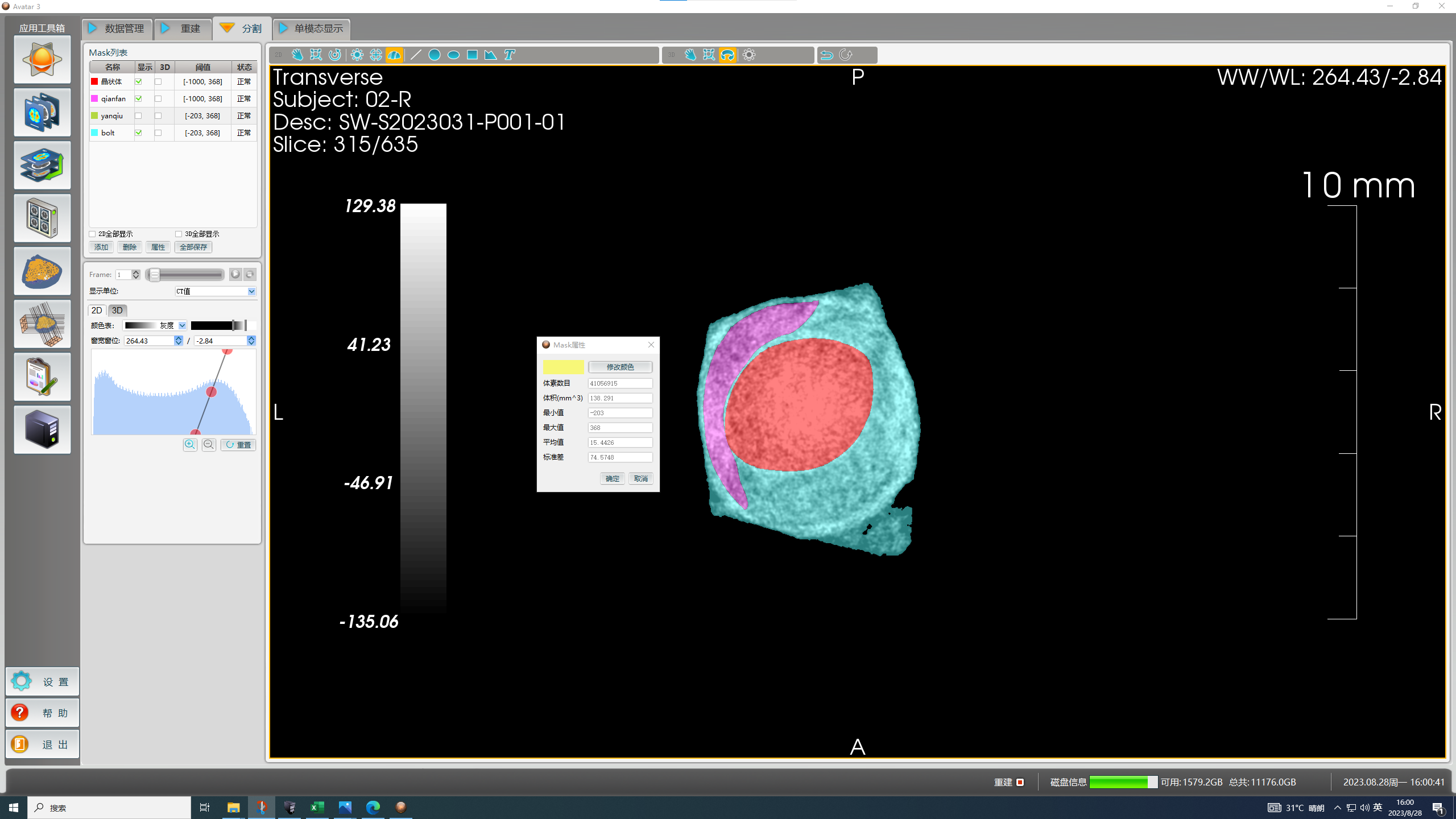

Supplement: S4 Data — (ZIP) [file pone.0310830.s004.zip › CT_SDrats/Eyeball volume/02-R.png]

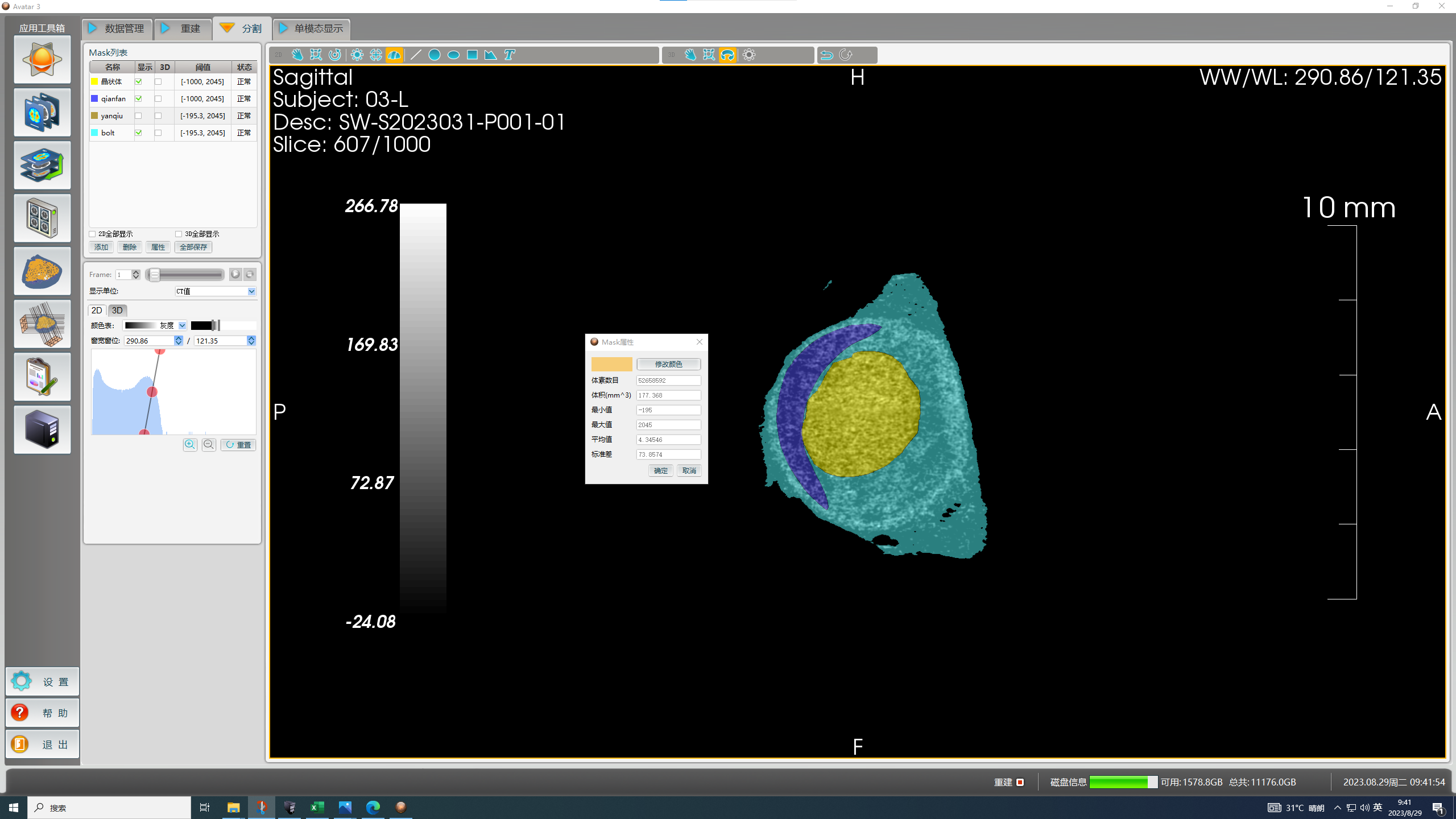

Supplement: S4 Data — (ZIP) [file pone.0310830.s004.zip › CT_SDrats/Eyeball volume/03-L.png]

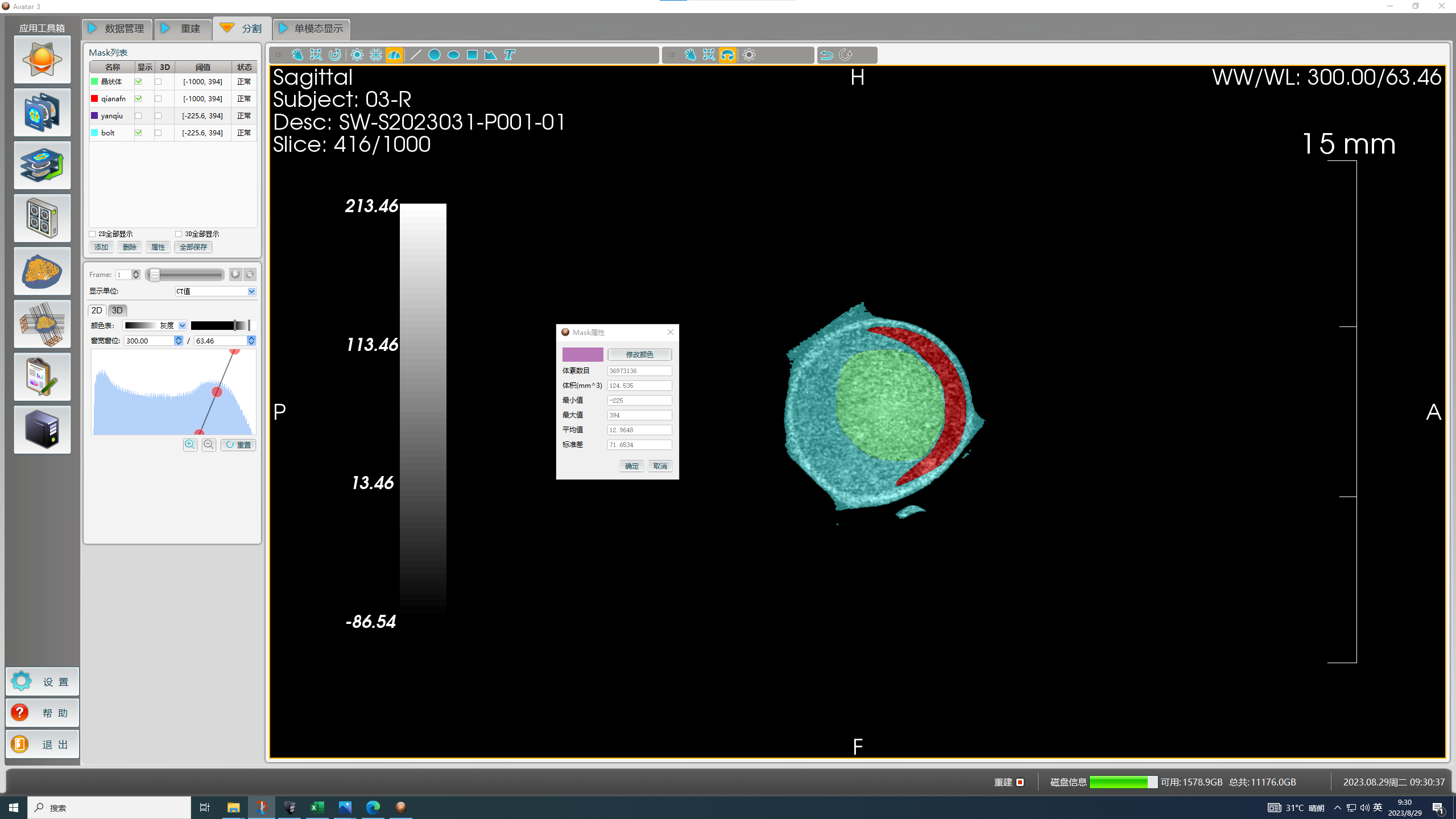

Supplement: S4 Data — (ZIP) [file pone.0310830.s004.zip › CT_SDrats/Eyeball volume/03-R.png]

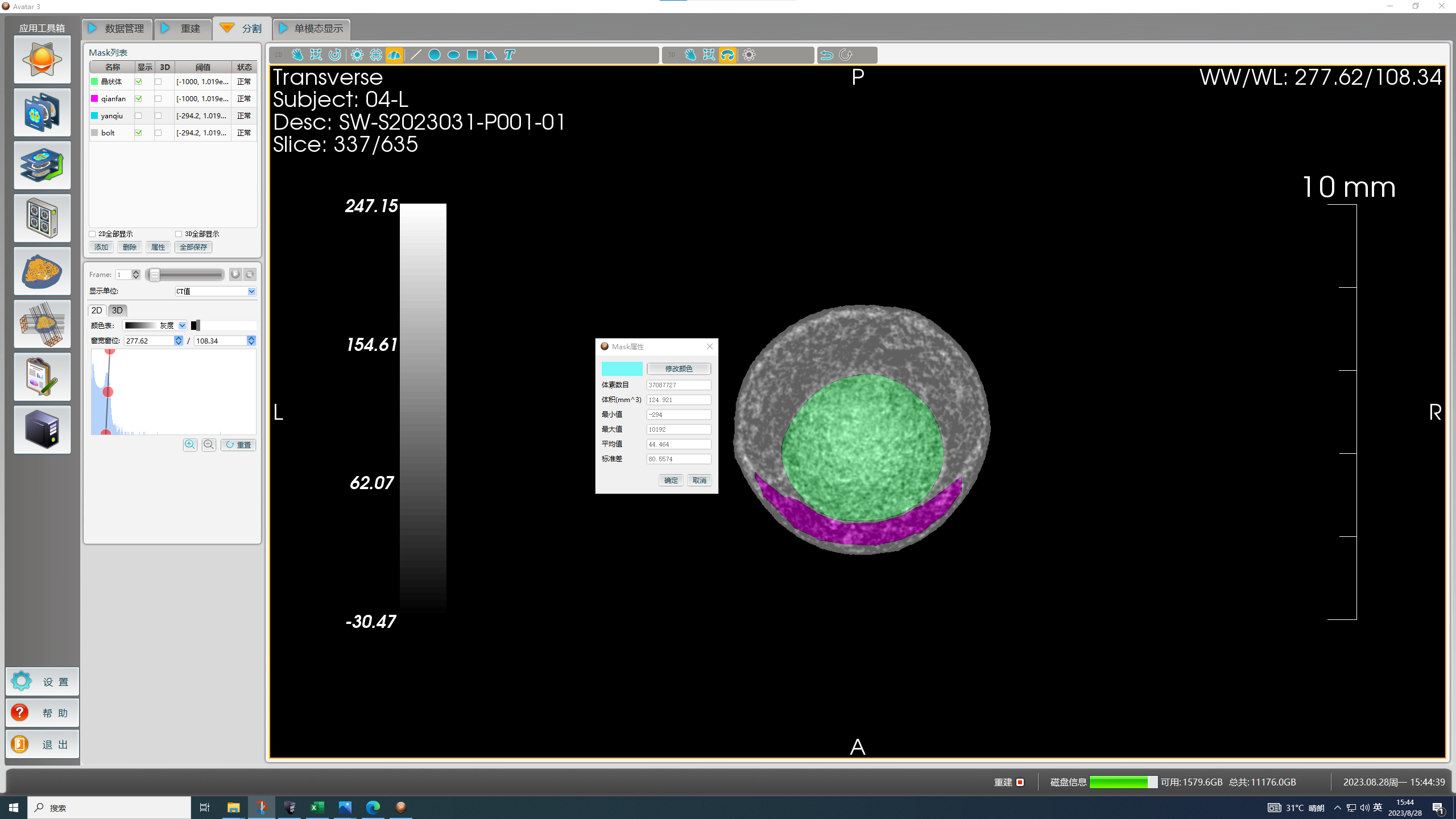

Supplement: S4 Data — (ZIP) [file pone.0310830.s004.zip › CT_SDrats/Eyeball volume/04-L.png]

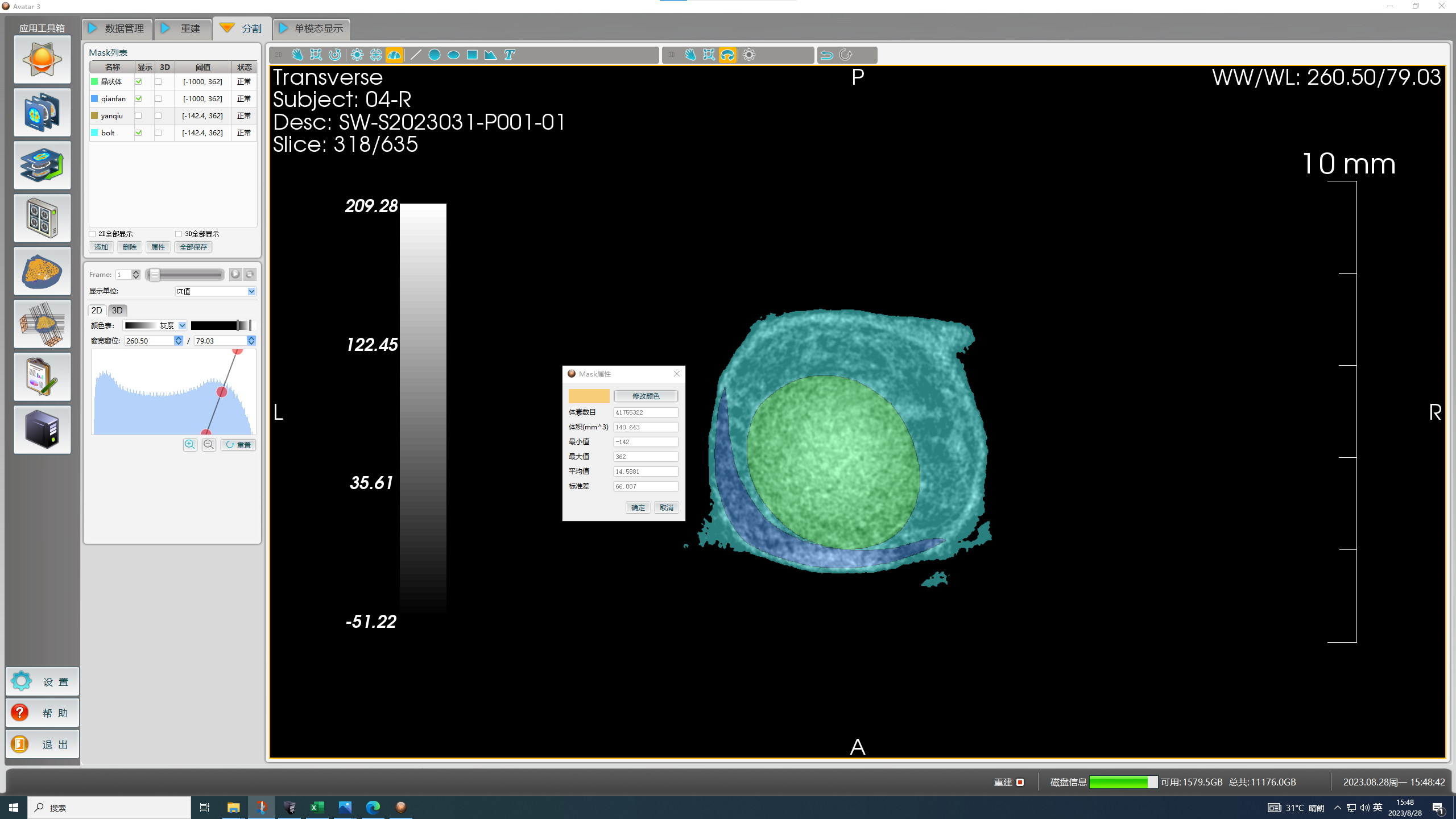

Supplement: S4 Data — (ZIP) [file pone.0310830.s004.zip › CT_SDrats/Eyeball volume/04-R.png]

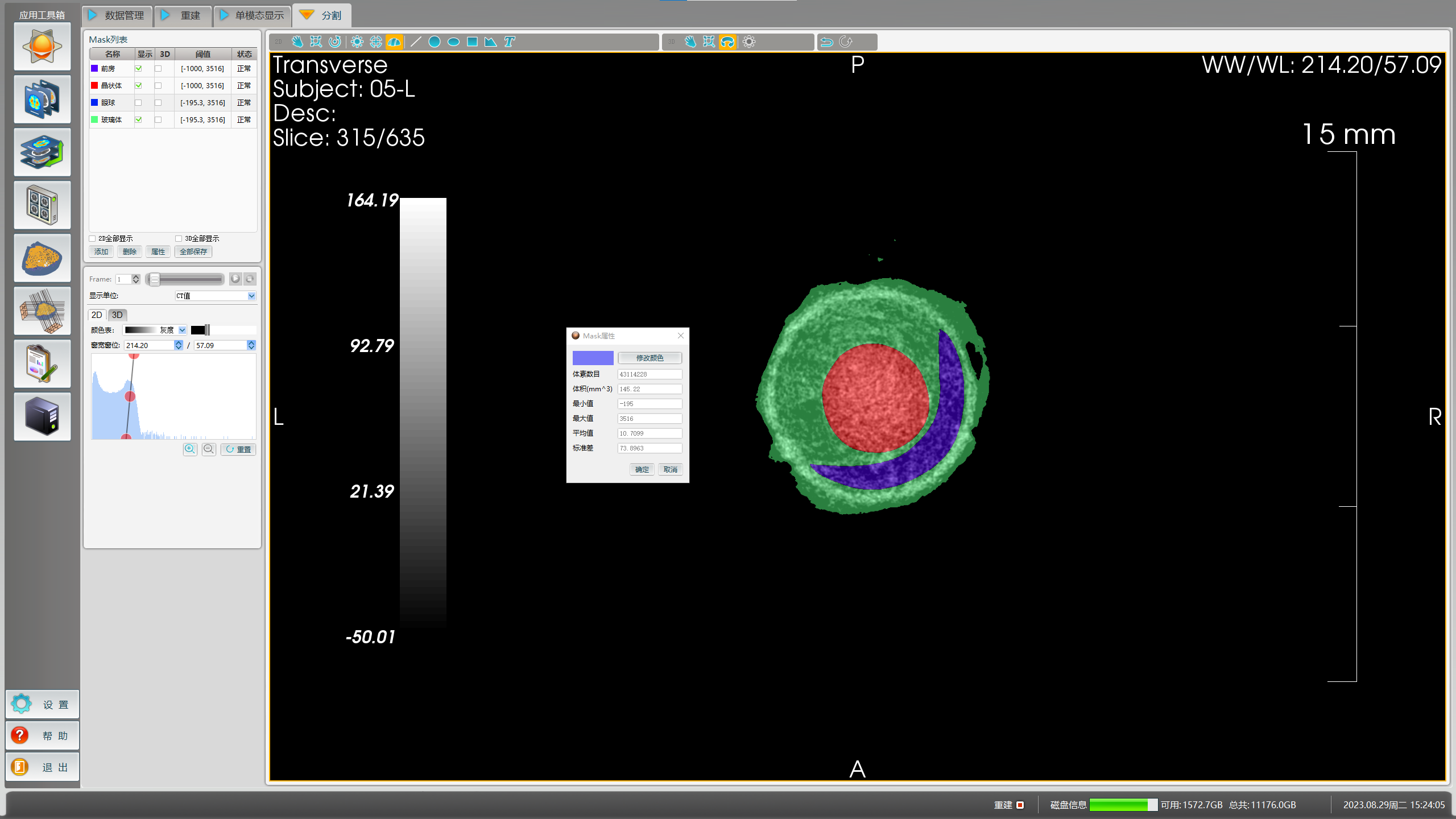

Supplement: S4 Data — (ZIP) [file pone.0310830.s004.zip › CT_SDrats/Eyeball volume/05-L.png]

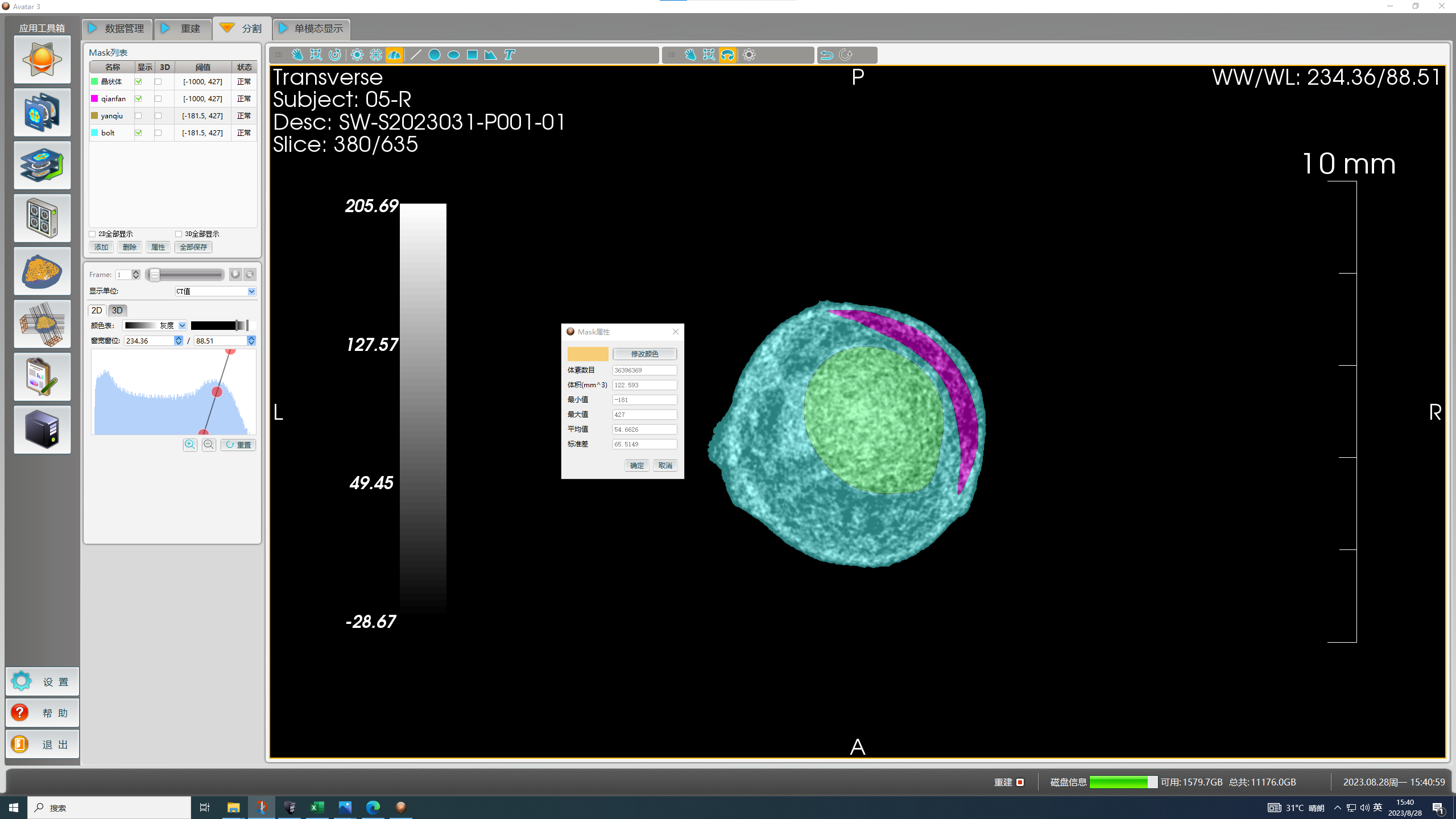

Supplement: S4 Data — (ZIP) [file pone.0310830.s004.zip › CT_SDrats/Eyeball volume/05-R.png]

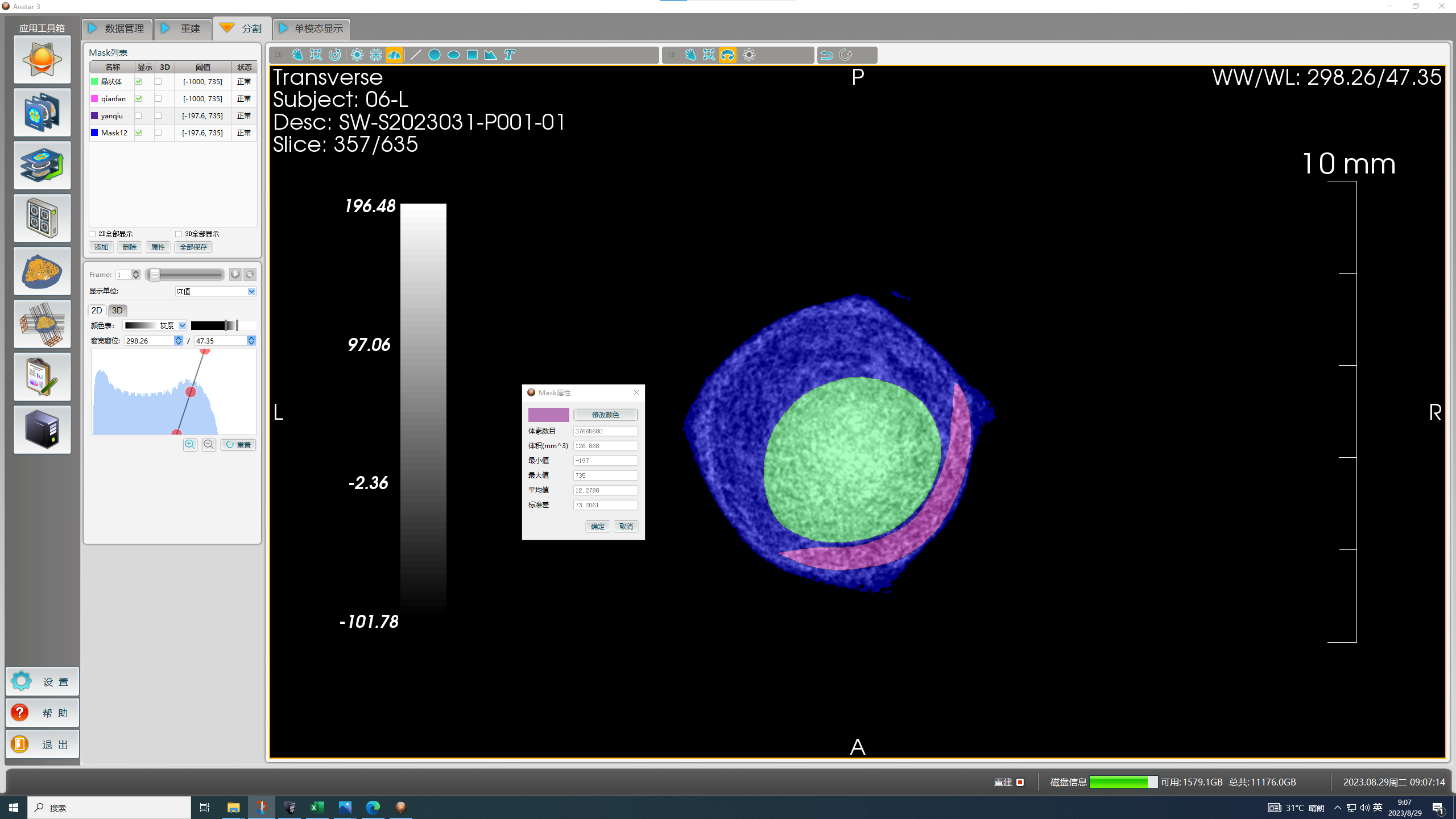

Supplement: S4 Data — (ZIP) [file pone.0310830.s004.zip › CT_SDrats/Eyeball volume/06-L.png]

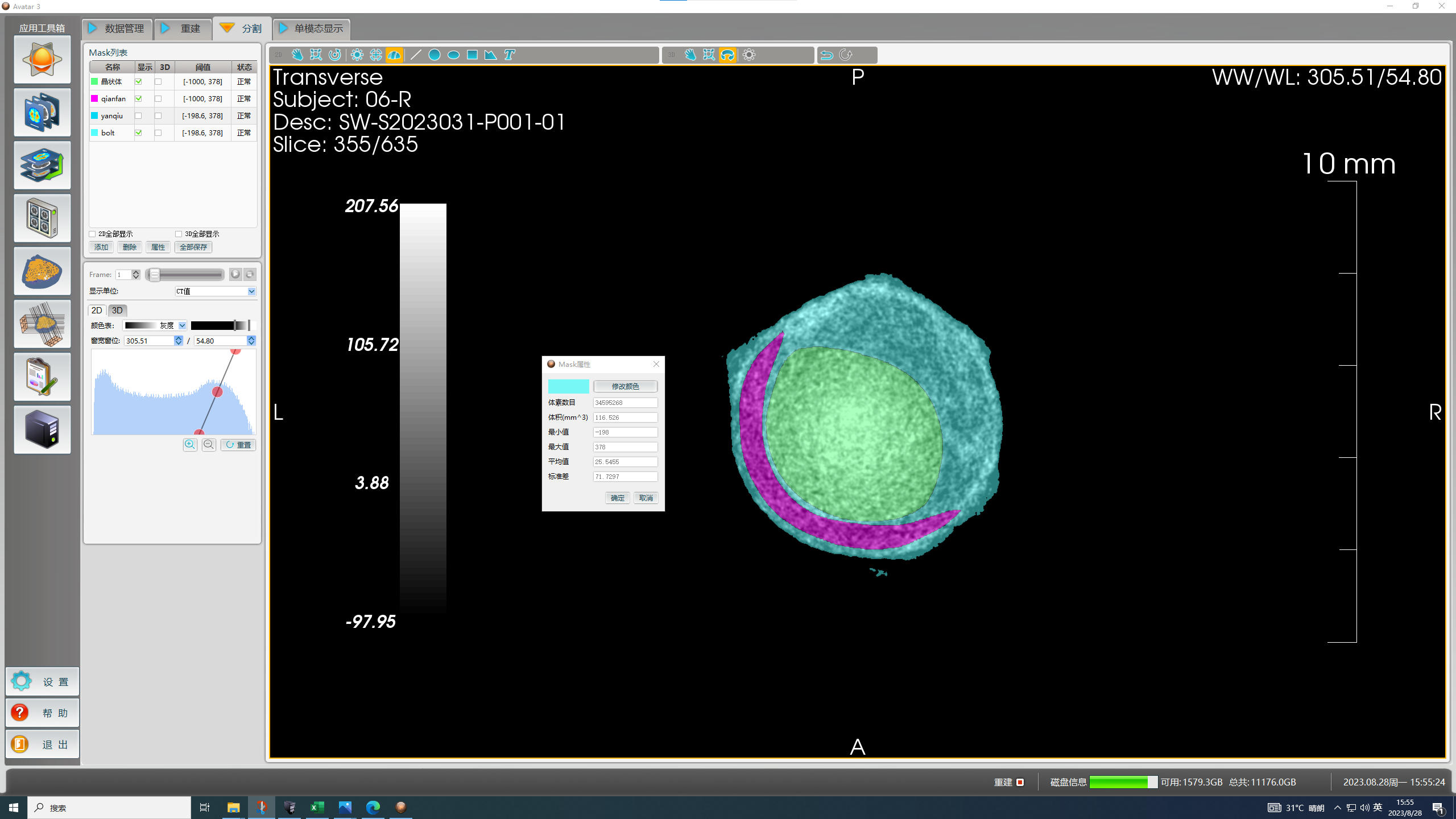

Supplement: S4 Data — (ZIP) [file pone.0310830.s004.zip › CT_SDrats/Eyeball volume/06-R.png]

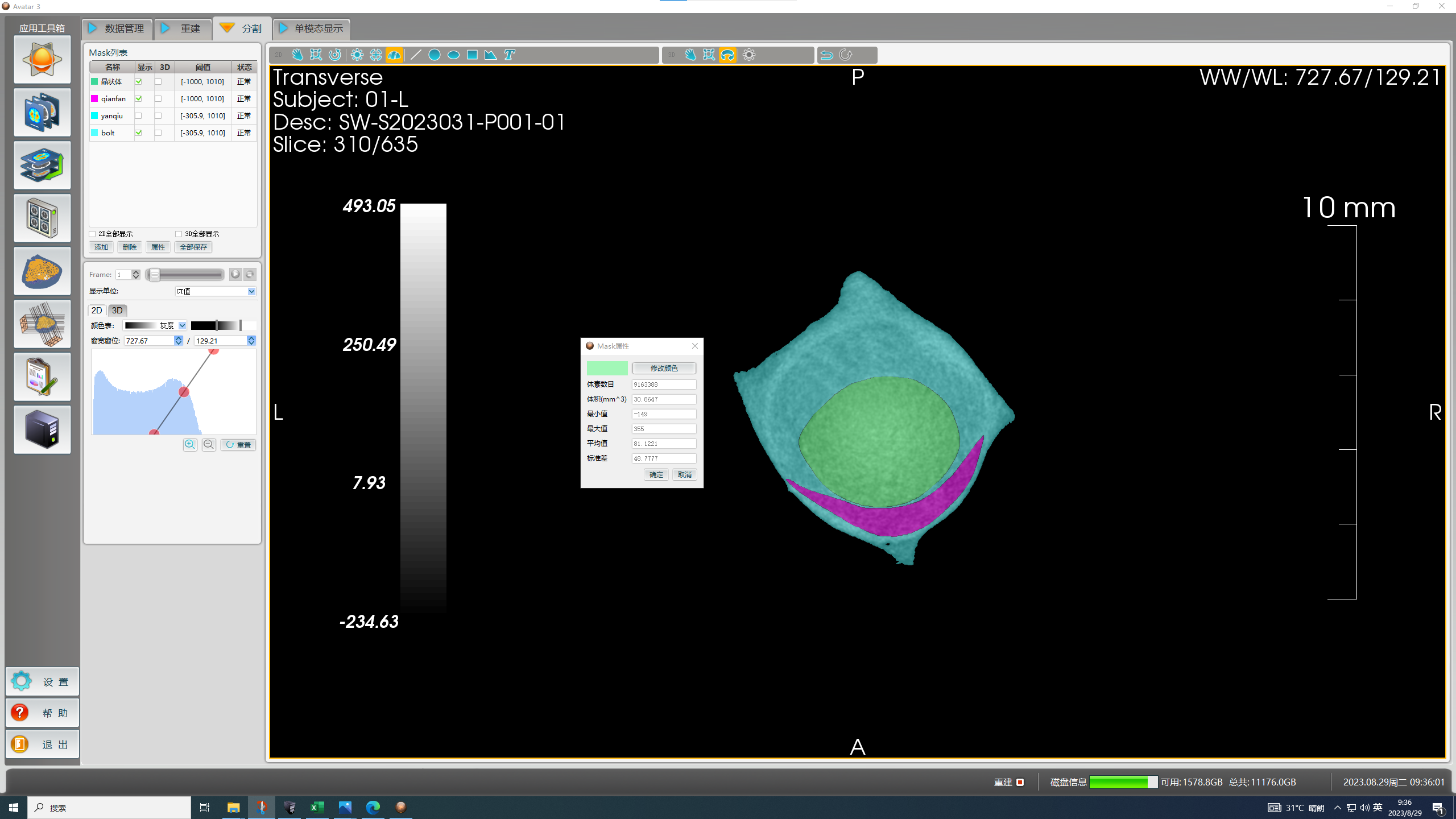

Supplement: S4 Data — (ZIP) [file pone.0310830.s004.zip › CT_SDrats/lens/01-L.png]

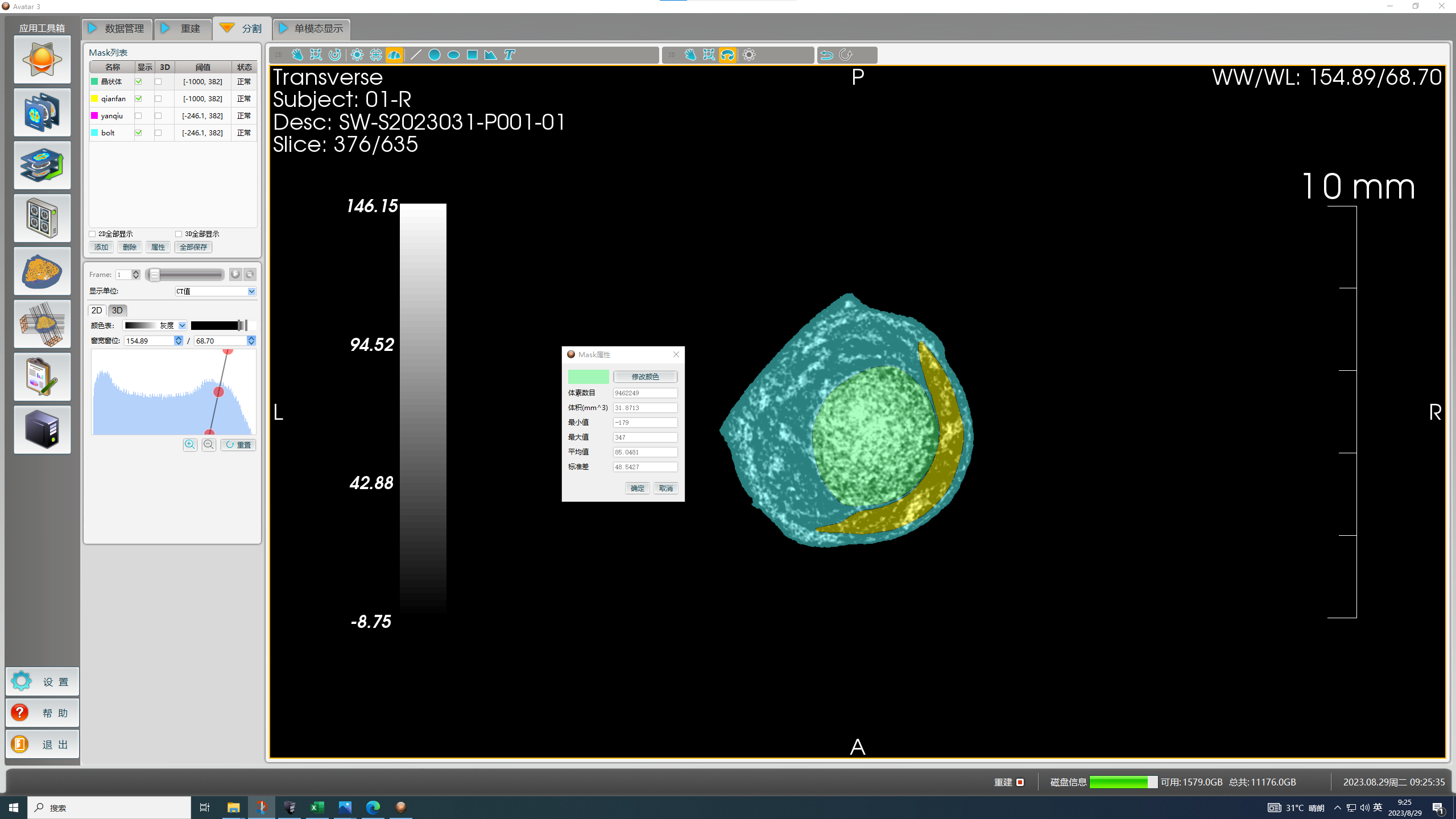

Supplement: S4 Data — (ZIP) [file pone.0310830.s004.zip › CT_SDrats/lens/01-R.png]

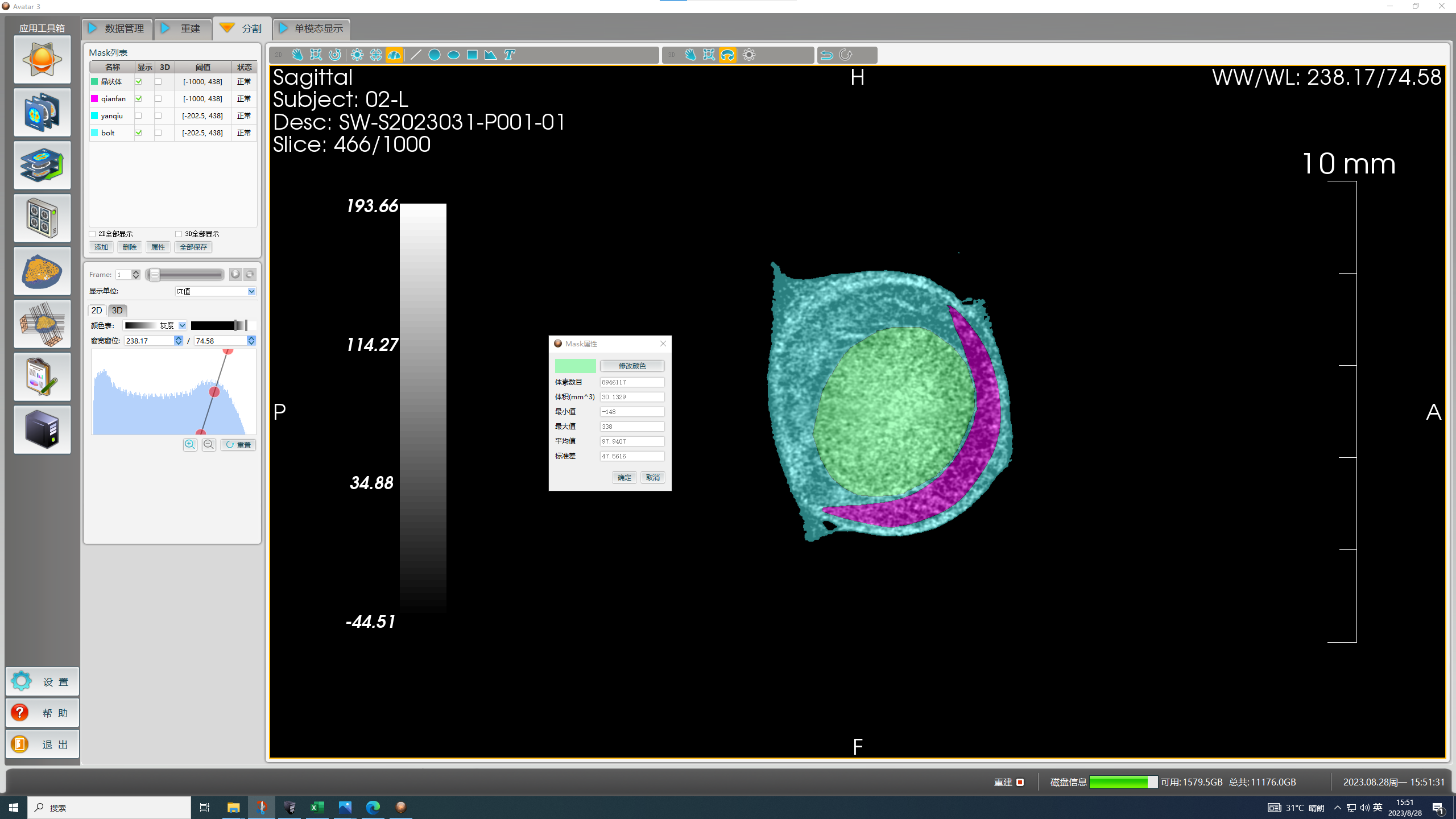

Supplement: S4 Data — (ZIP) [file pone.0310830.s004.zip › CT_SDrats/lens/02-L.png]

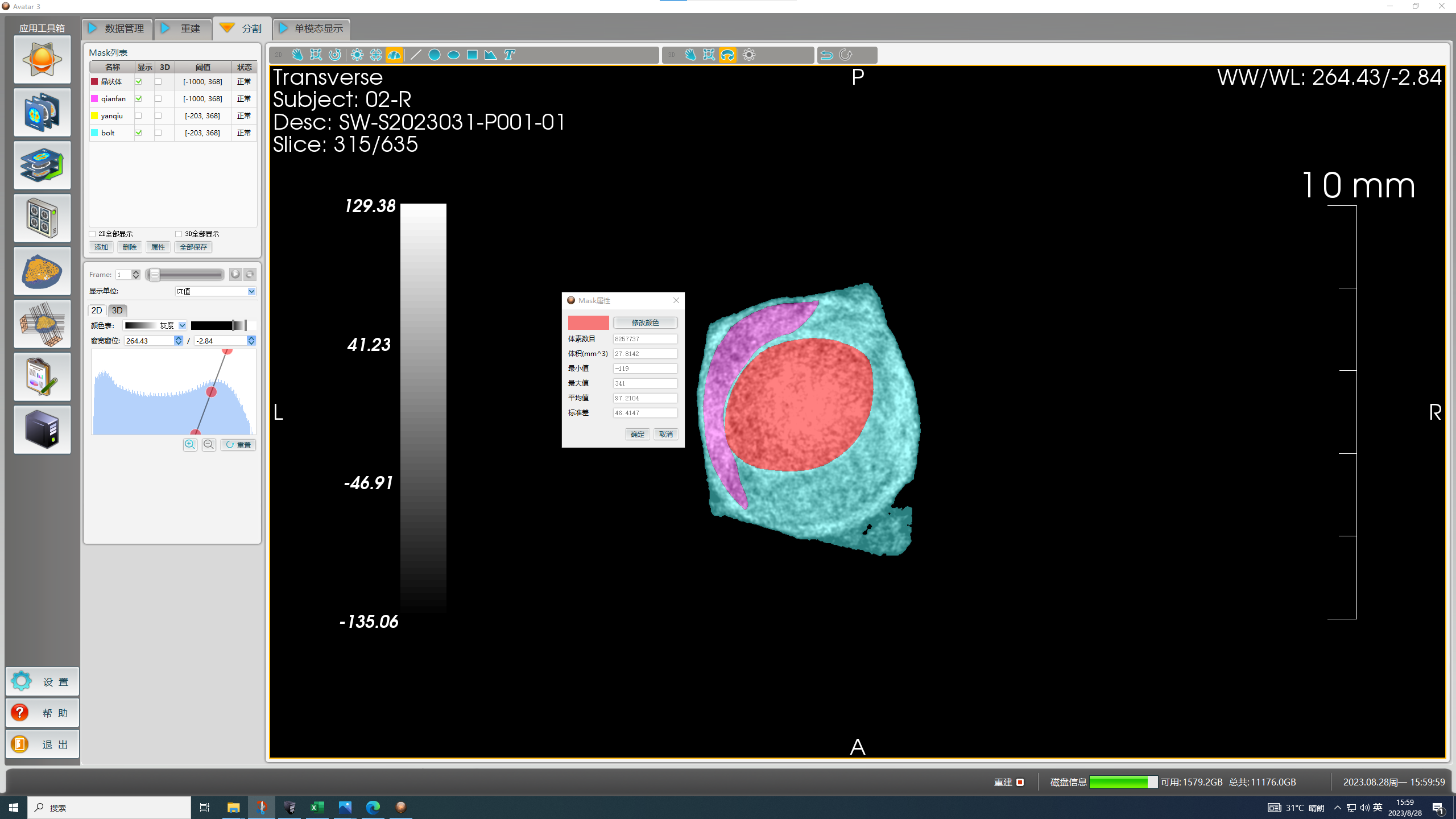

Supplement: S4 Data — (ZIP) [file pone.0310830.s004.zip › CT_SDrats/lens/02-R.png]

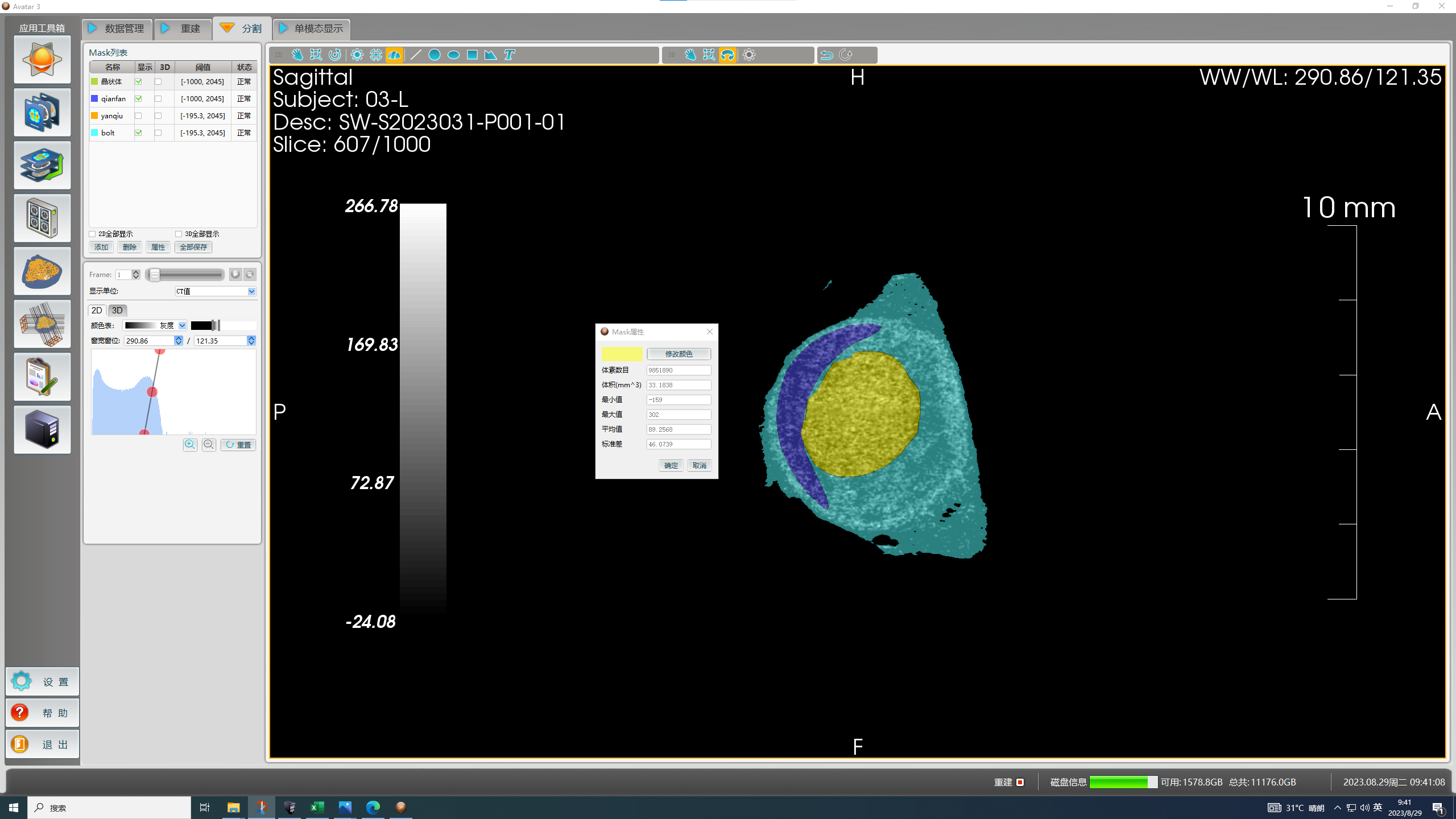

Supplement: S4 Data — (ZIP) [file pone.0310830.s004.zip › CT_SDrats/lens/03-L.png]

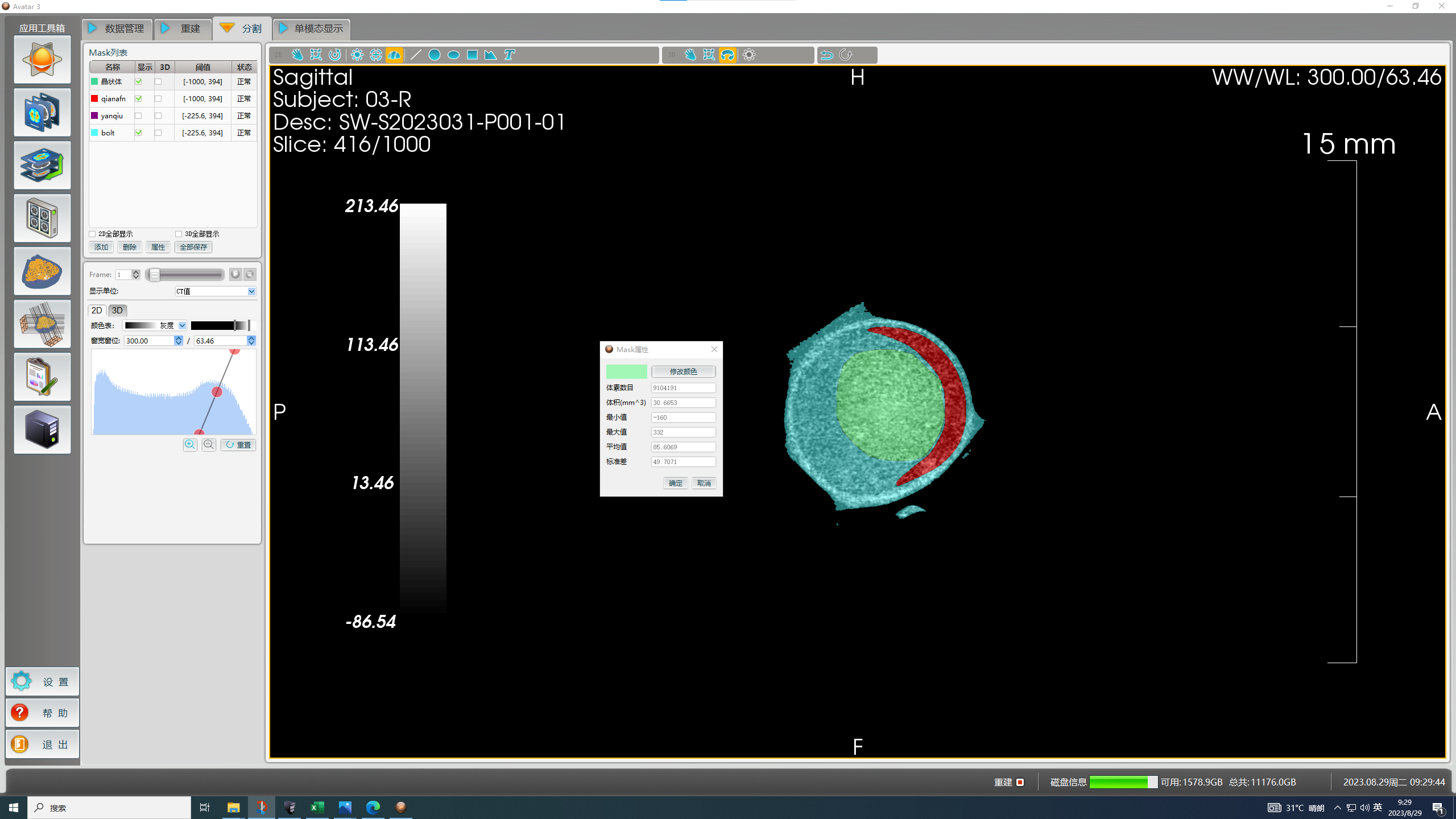

Supplement: S4 Data — (ZIP) [file pone.0310830.s004.zip › CT_SDrats/lens/03-R.png]

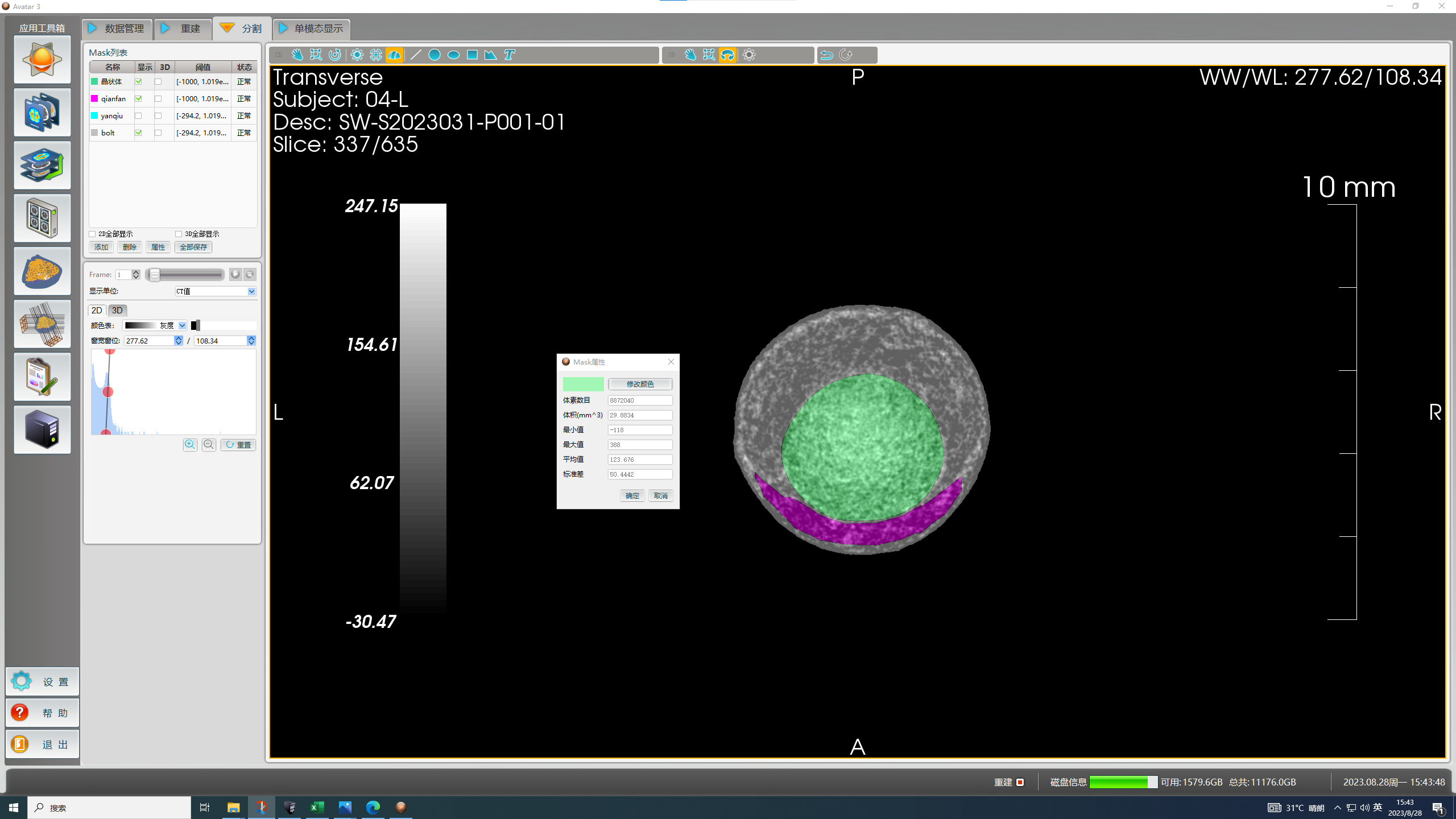

Supplement: S4 Data — (ZIP) [file pone.0310830.s004.zip › CT_SDrats/lens/04-L.png]

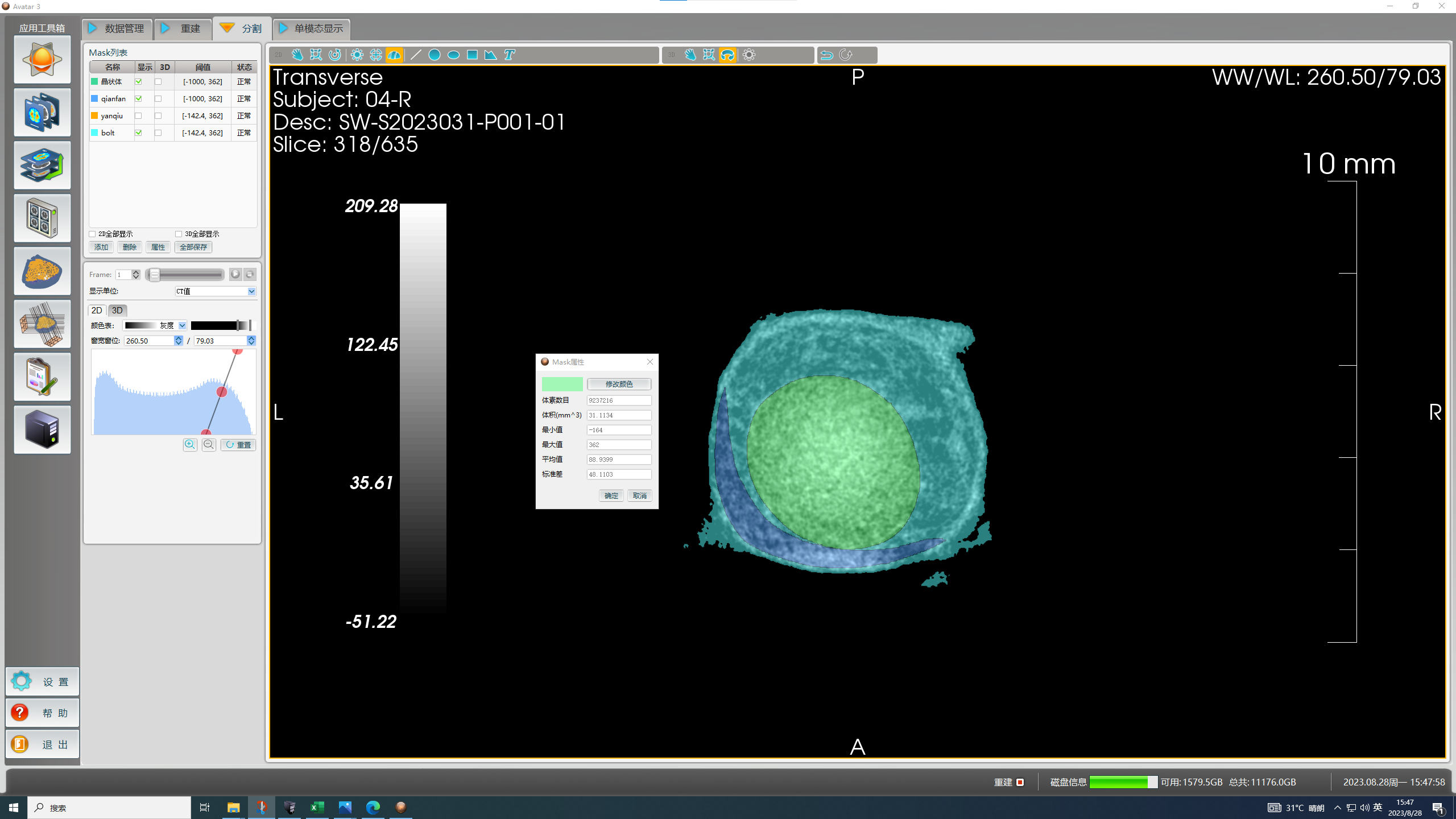

Supplement: S4 Data — (ZIP) [file pone.0310830.s004.zip › CT_SDrats/lens/04-R.png]

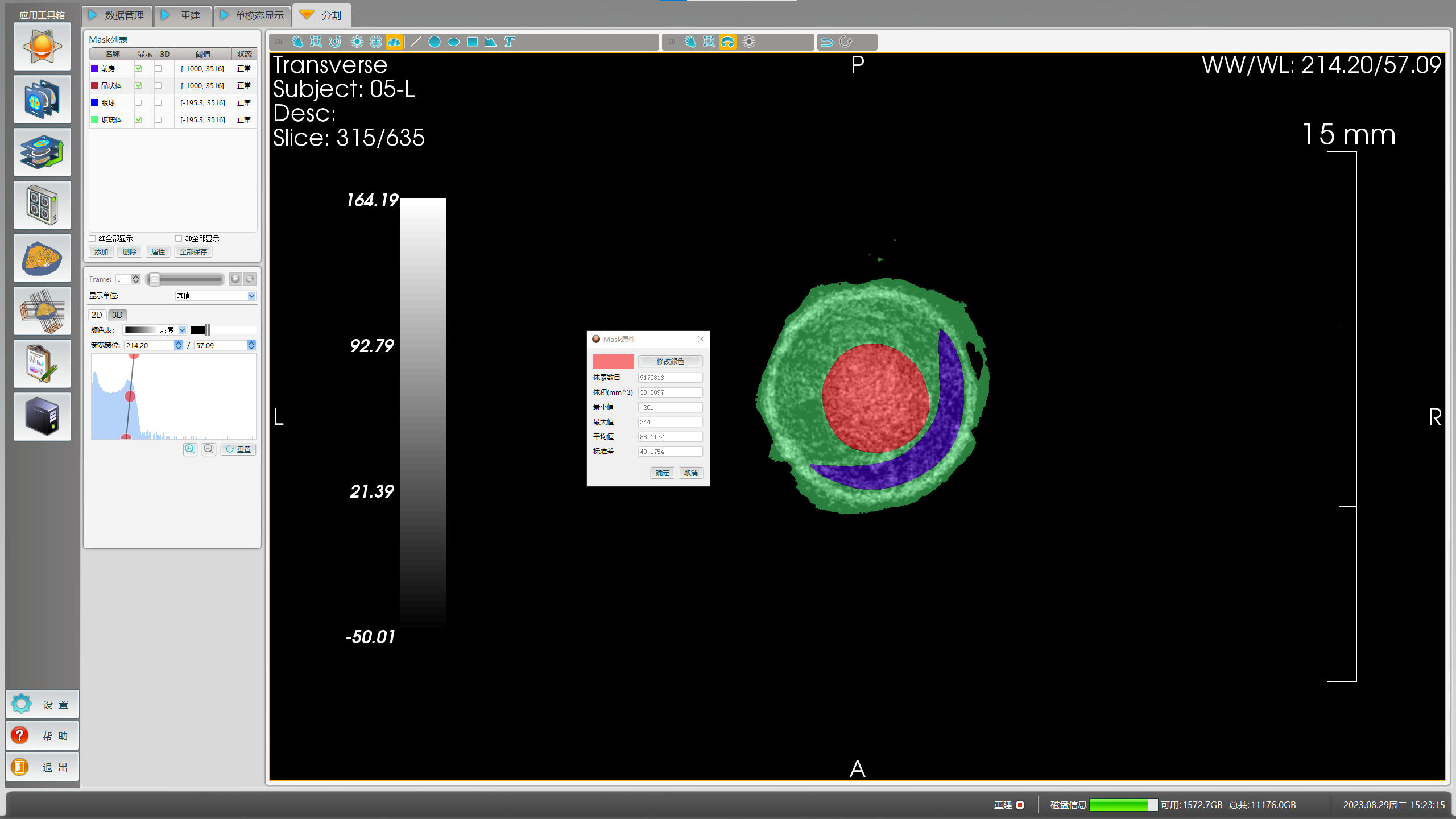

Supplement: S4 Data — (ZIP) [file pone.0310830.s004.zip › CT_SDrats/lens/05-L.png]

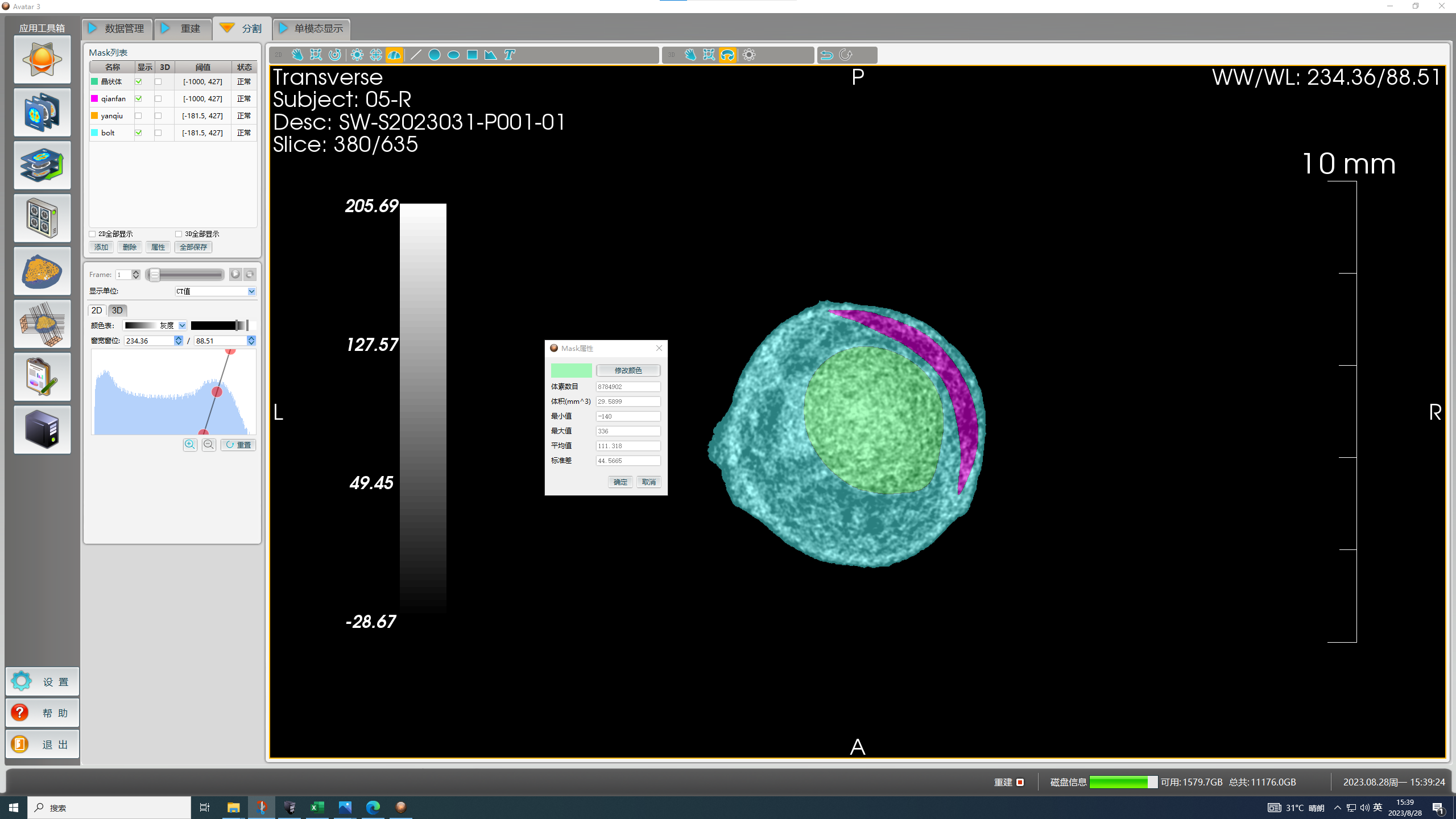

Supplement: S4 Data — (ZIP) [file pone.0310830.s004.zip › CT_SDrats/lens/05-R.png]

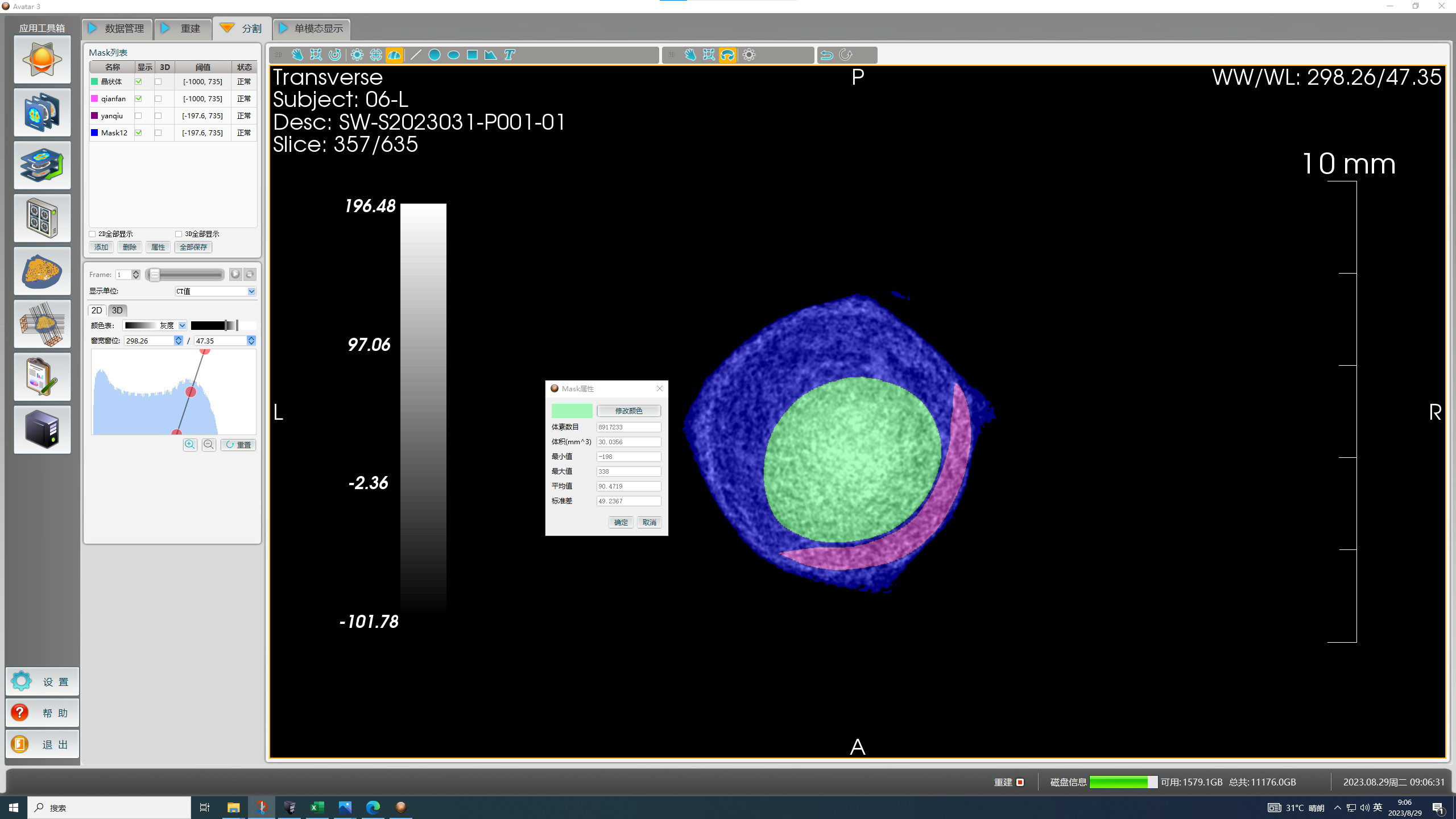

Supplement: S4 Data — (ZIP) [file pone.0310830.s004.zip › CT_SDrats/lens/06-L.png]

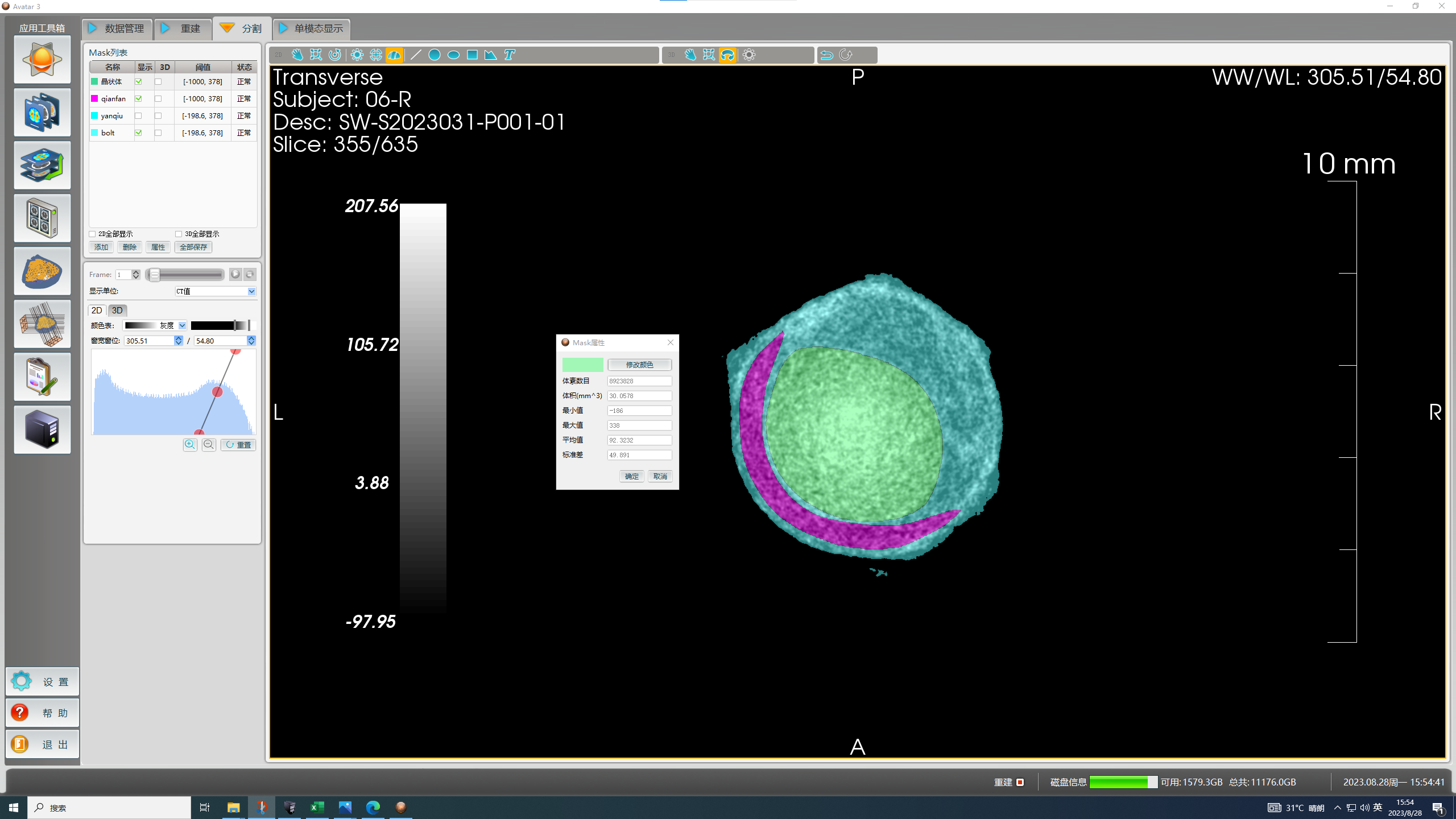

Supplement: S4 Data — (ZIP) [file pone.0310830.s004.zip › CT_SDrats/lens/06-R.png]

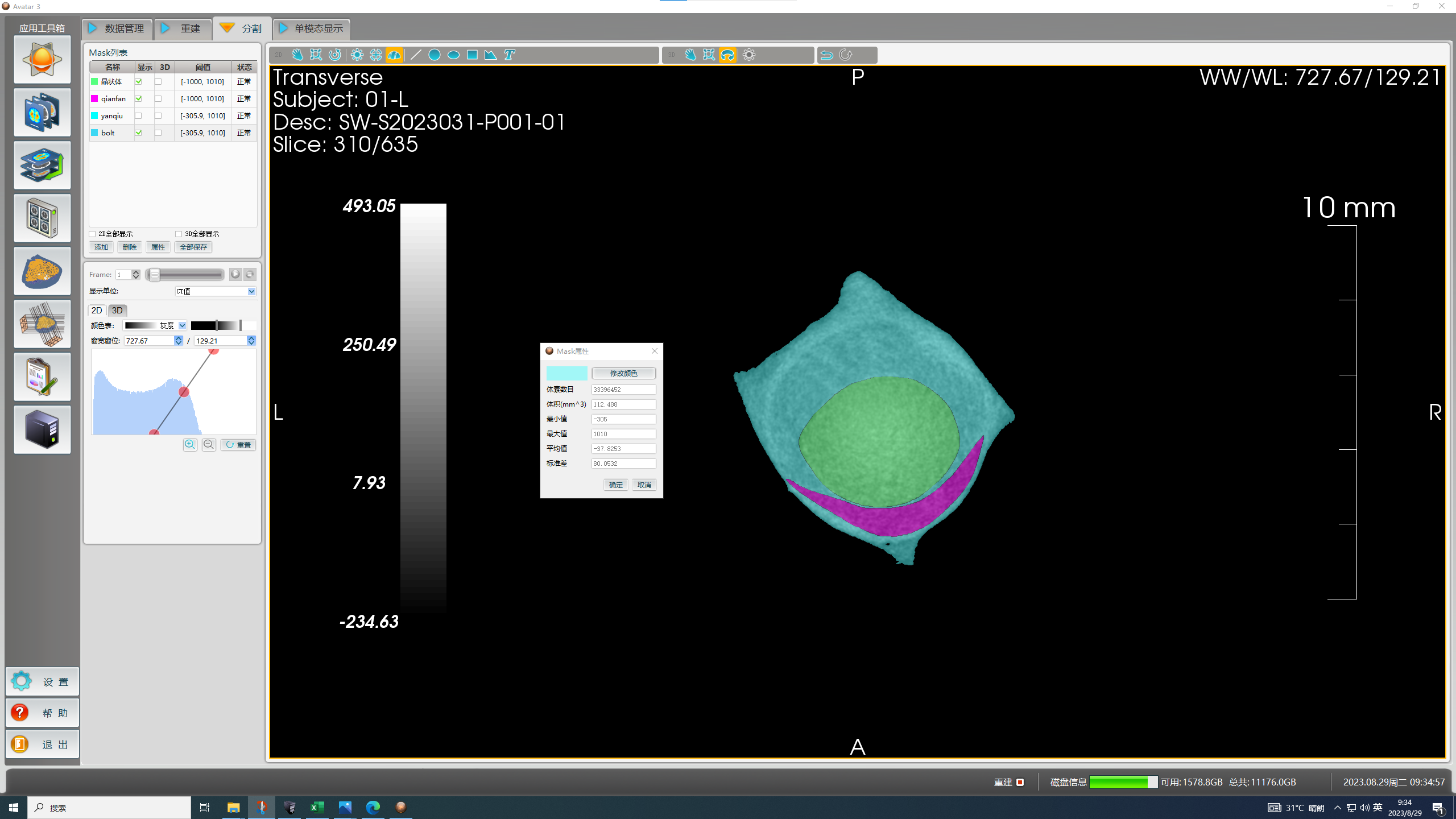

Supplement: S4 Data — (ZIP) [file pone.0310830.s004.zip › CT_SDrats/Vitreous body/01-L.png]

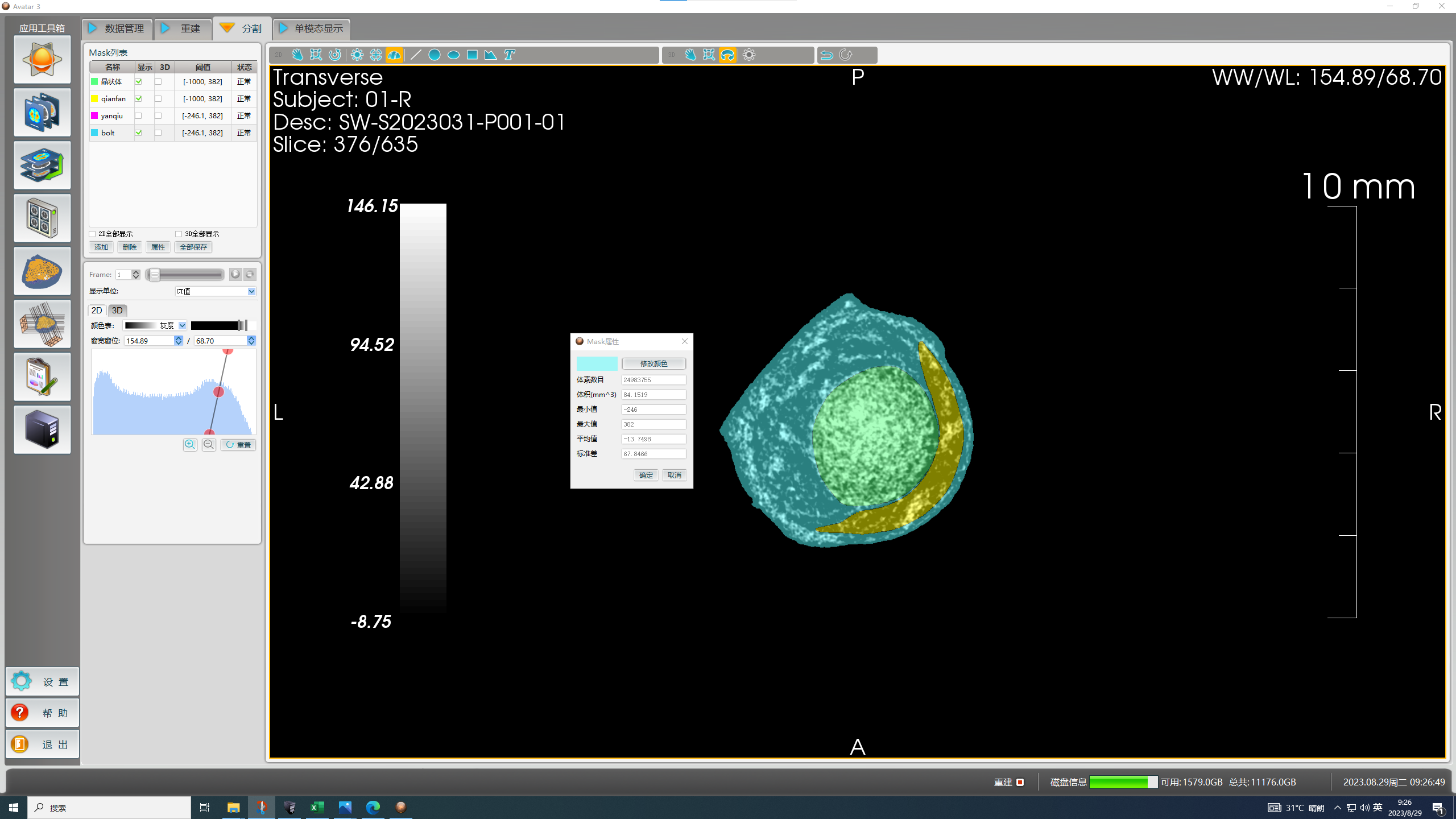

Supplement: S4 Data — (ZIP) [file pone.0310830.s004.zip › CT_SDrats/Vitreous body/01-R.png]

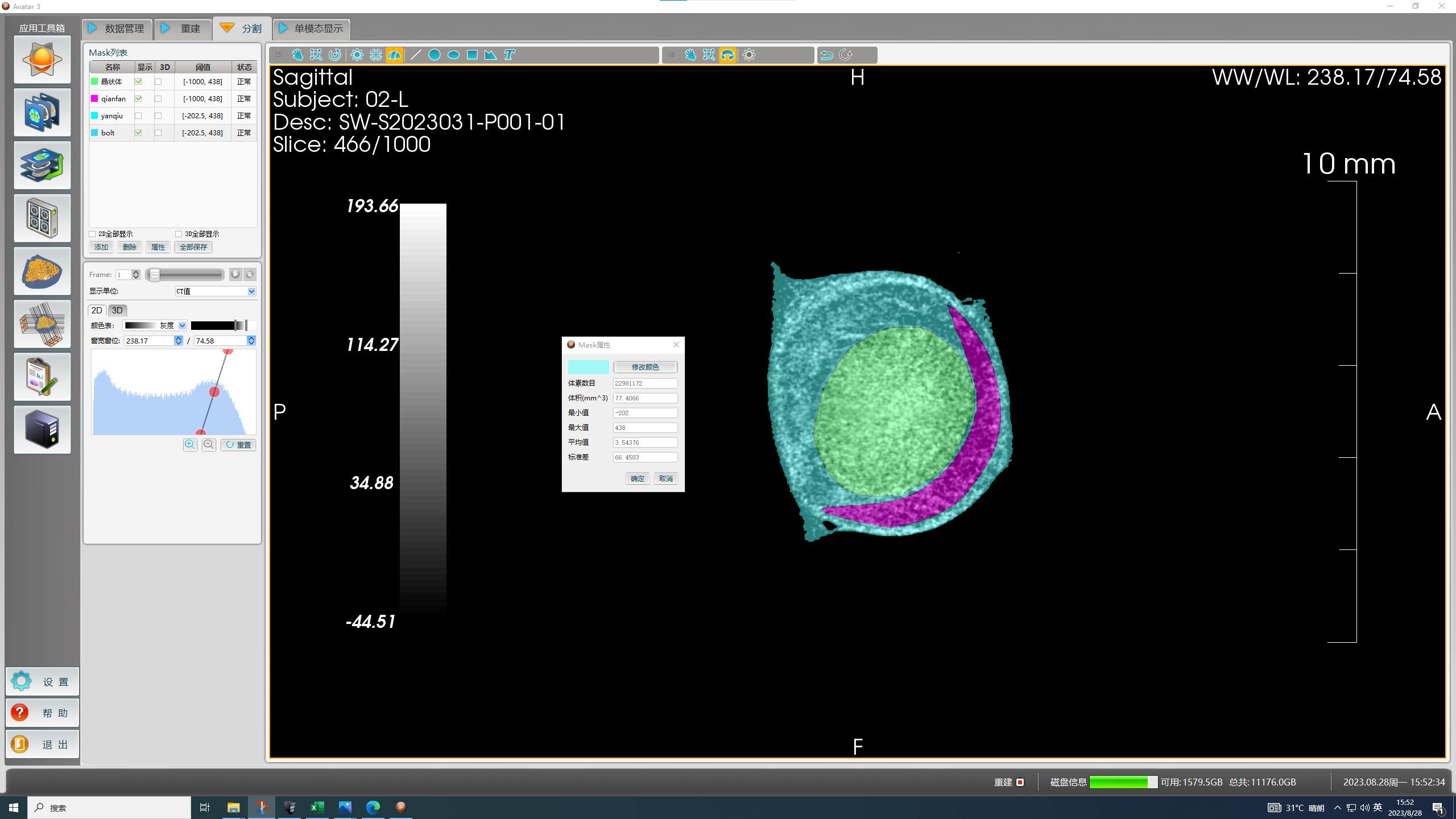

Supplement: S4 Data — (ZIP) [file pone.0310830.s004.zip › CT_SDrats/Vitreous body/02-L.png]

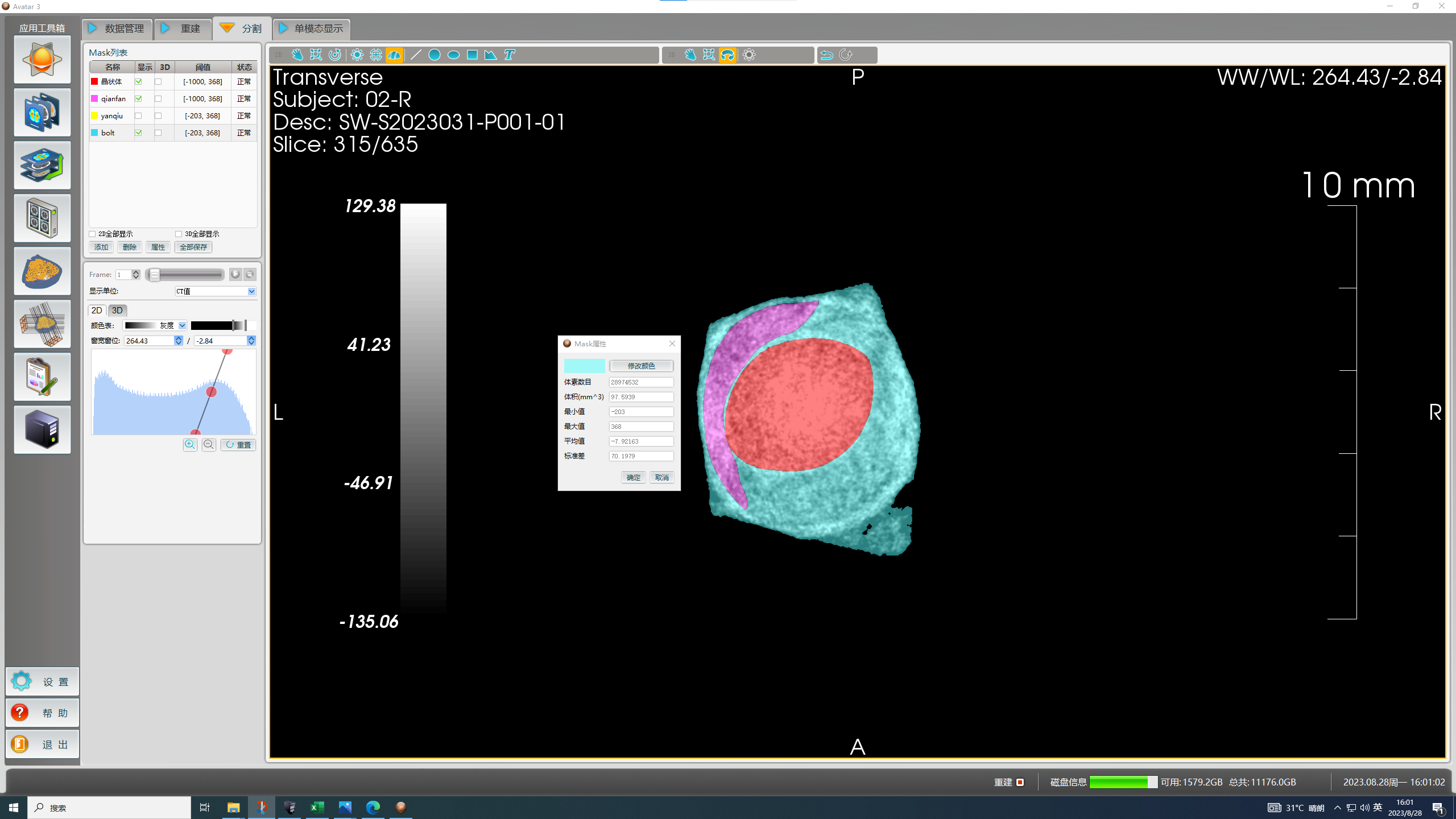

Supplement: S4 Data — (ZIP) [file pone.0310830.s004.zip › CT_SDrats/Vitreous body/02-R.png]

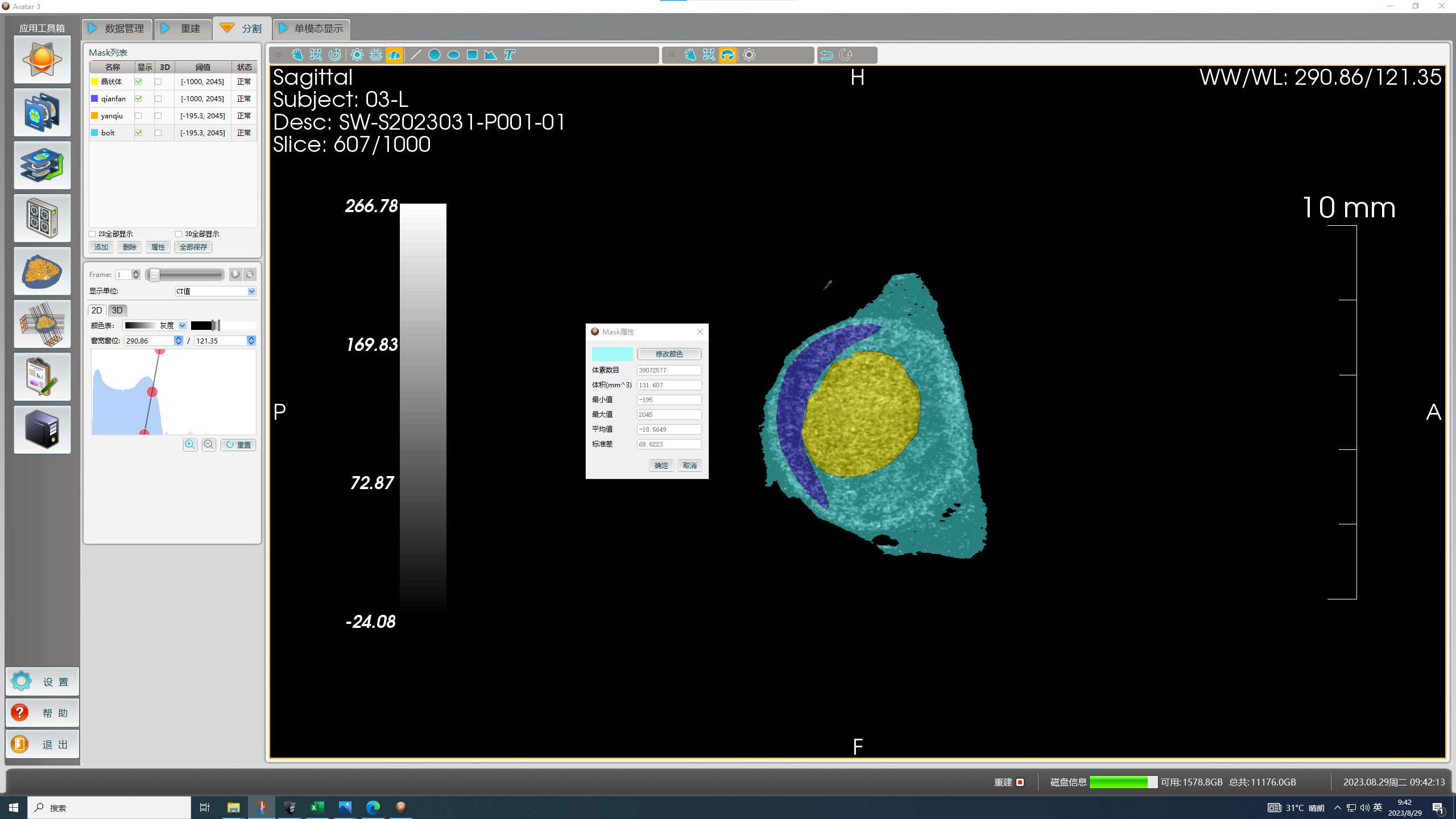

Supplement: S4 Data — (ZIP) [file pone.0310830.s004.zip › CT_SDrats/Vitreous body/03-L.png]

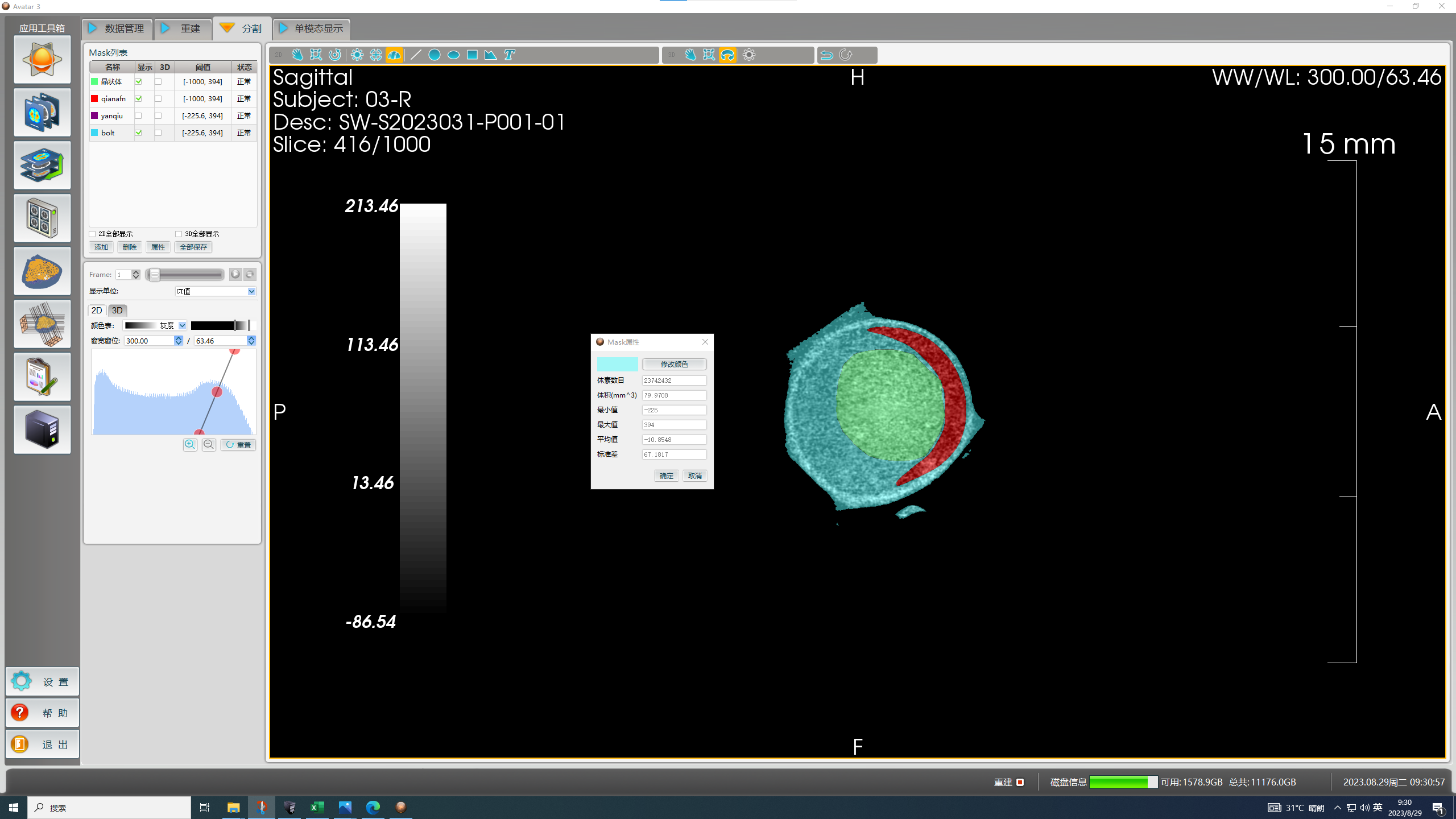

Supplement: S4 Data — (ZIP) [file pone.0310830.s004.zip › CT_SDrats/Vitreous body/03-R.png]

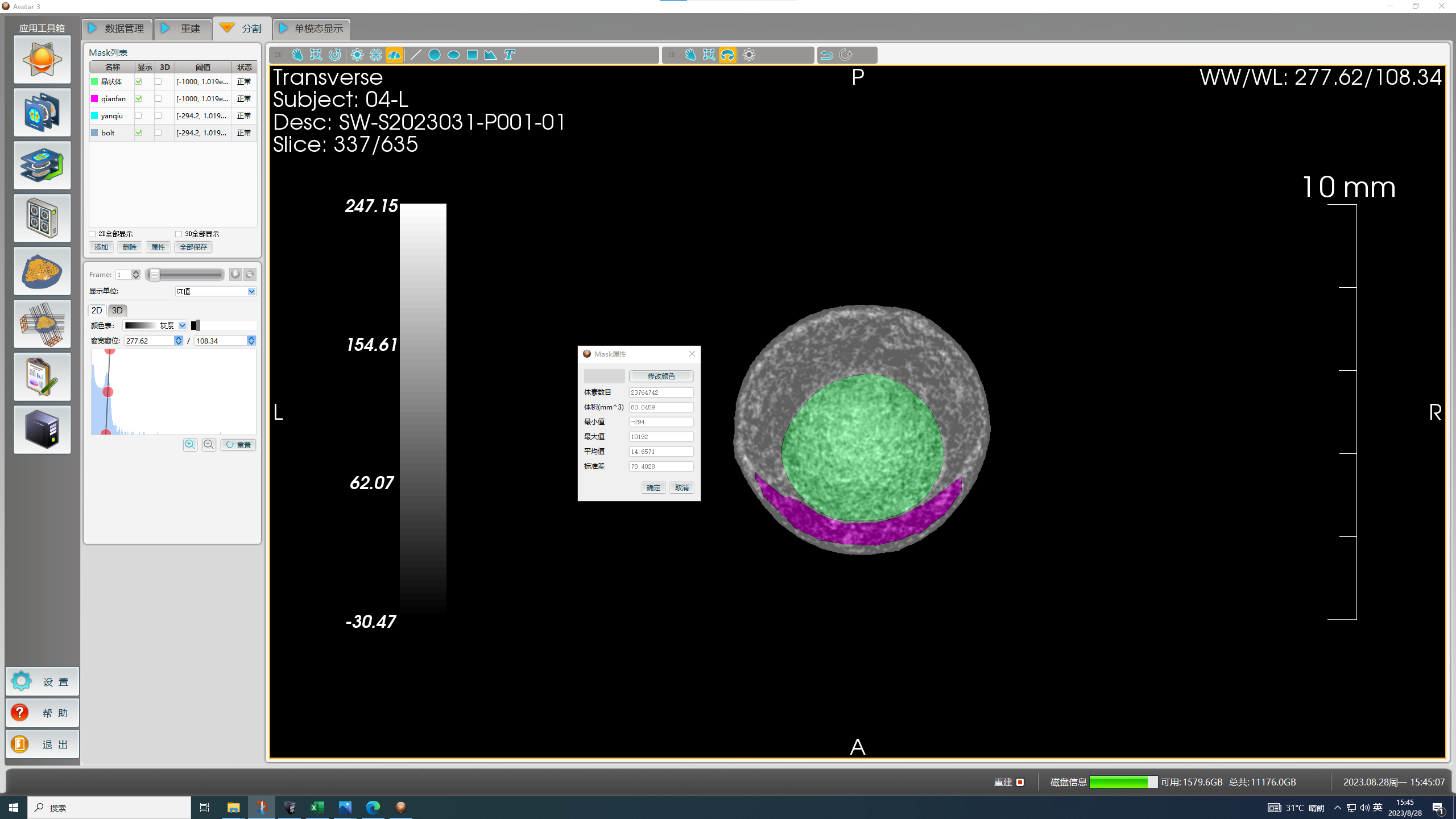

Supplement: S4 Data — (ZIP) [file pone.0310830.s004.zip › CT_SDrats/Vitreous body/04-L.png]

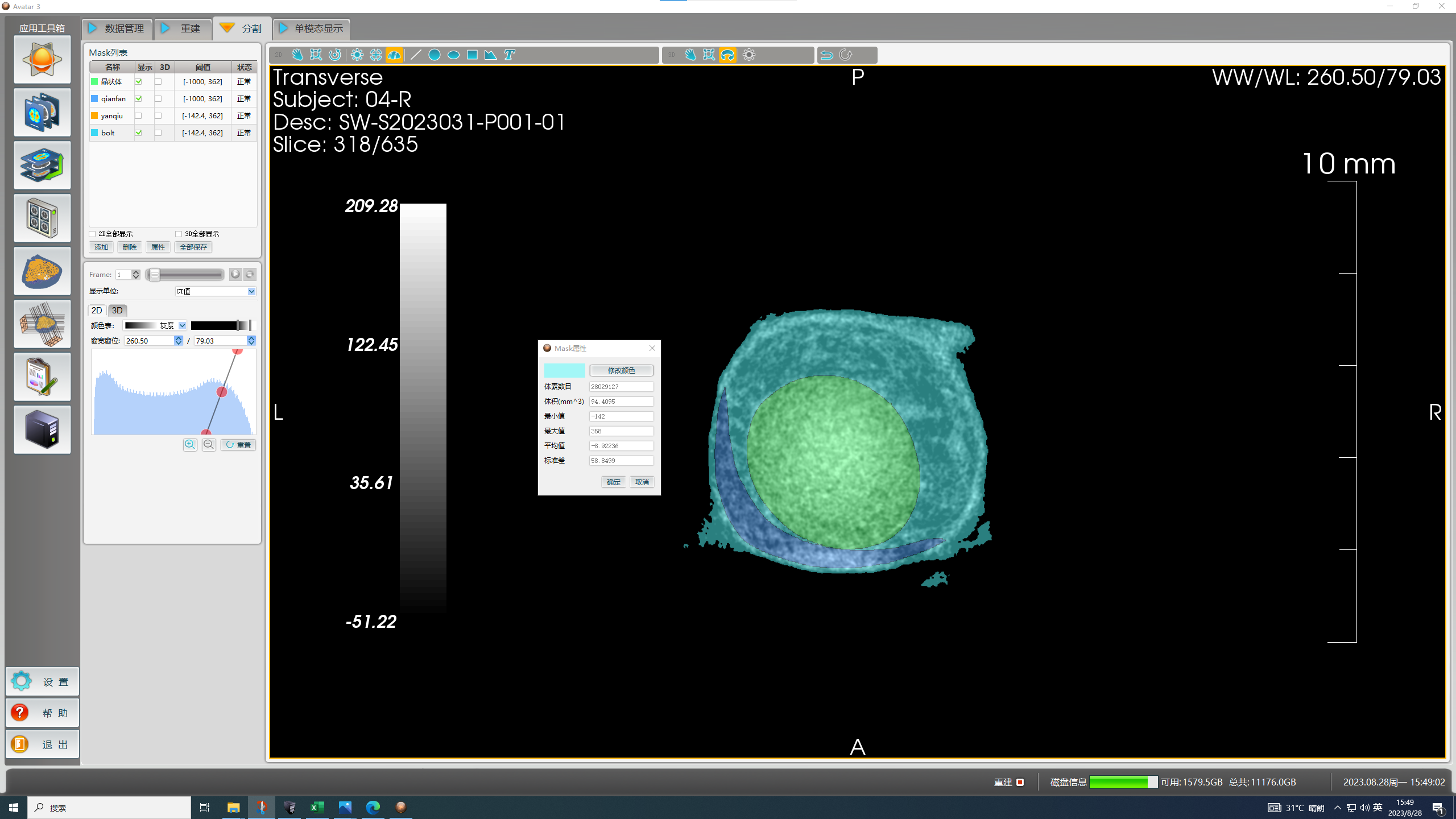

Supplement: S4 Data — (ZIP) [file pone.0310830.s004.zip › CT_SDrats/Vitreous body/04-R.png]

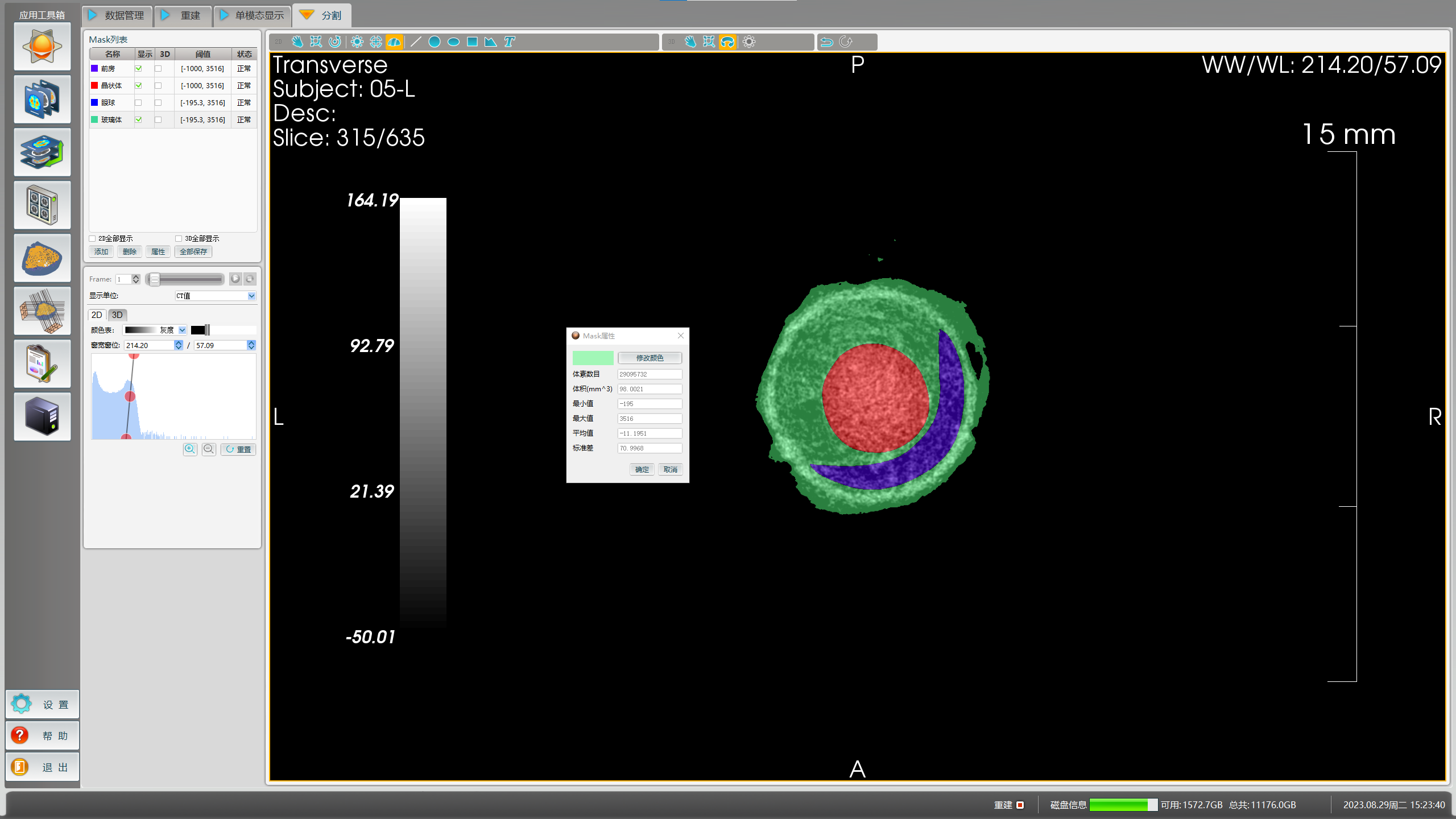

Supplement: S4 Data — (ZIP) [file pone.0310830.s004.zip › CT_SDrats/Vitreous body/05-L.png]

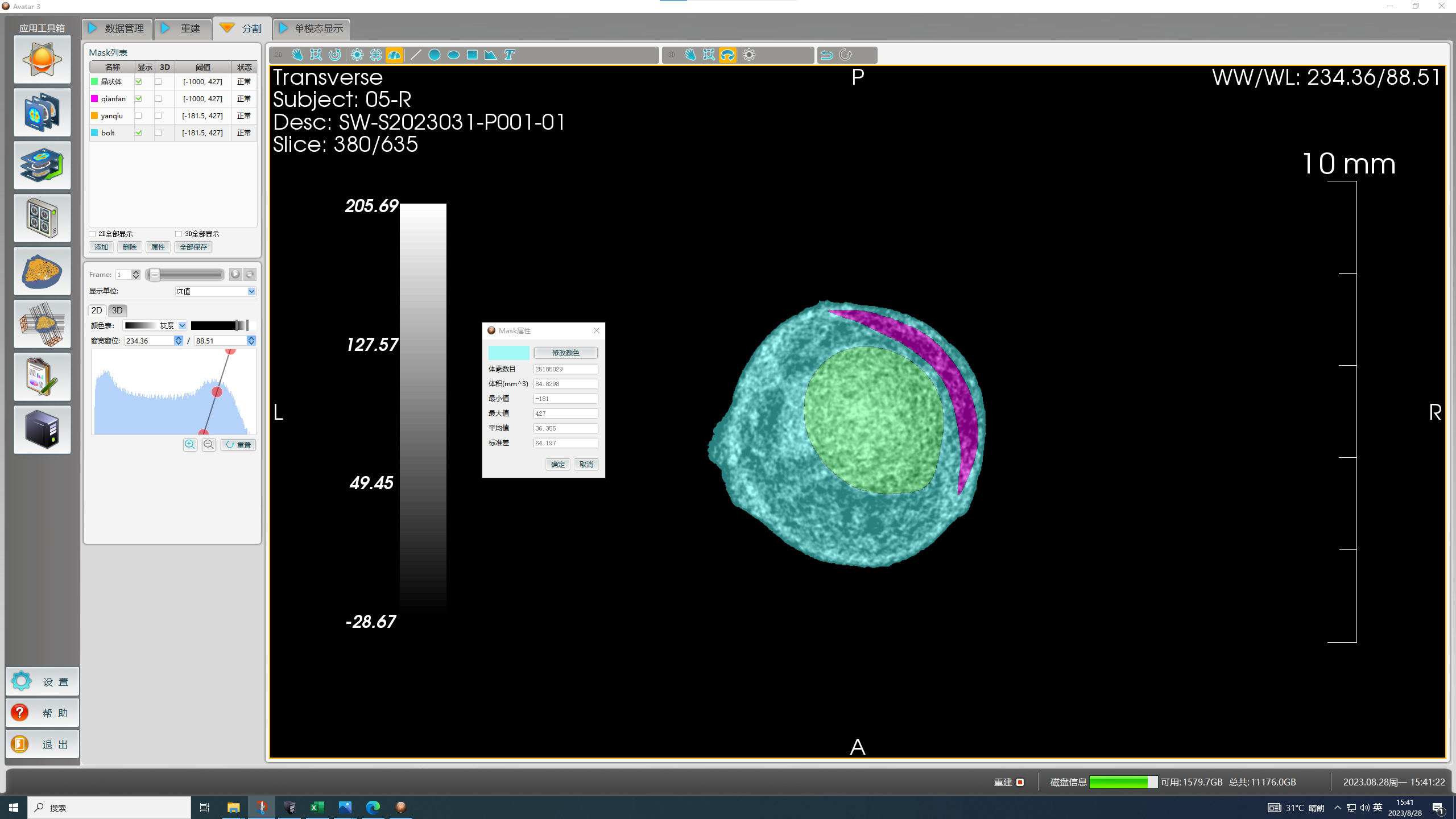

Supplement: S4 Data — (ZIP) [file pone.0310830.s004.zip › CT_SDrats/Vitreous body/05-R.png]

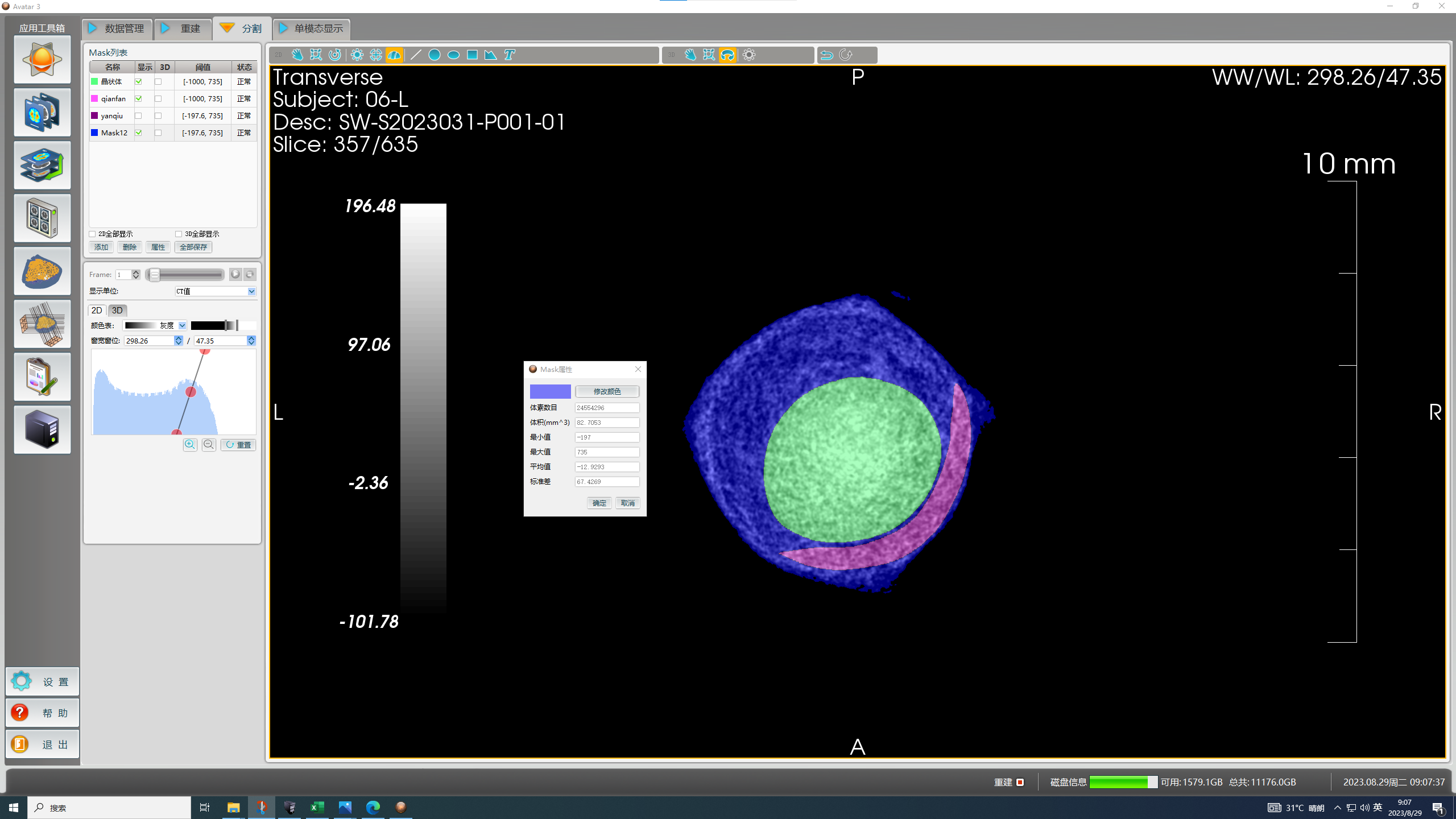

Supplement: S4 Data — (ZIP) [file pone.0310830.s004.zip › CT_SDrats/Vitreous body/06-L.png]

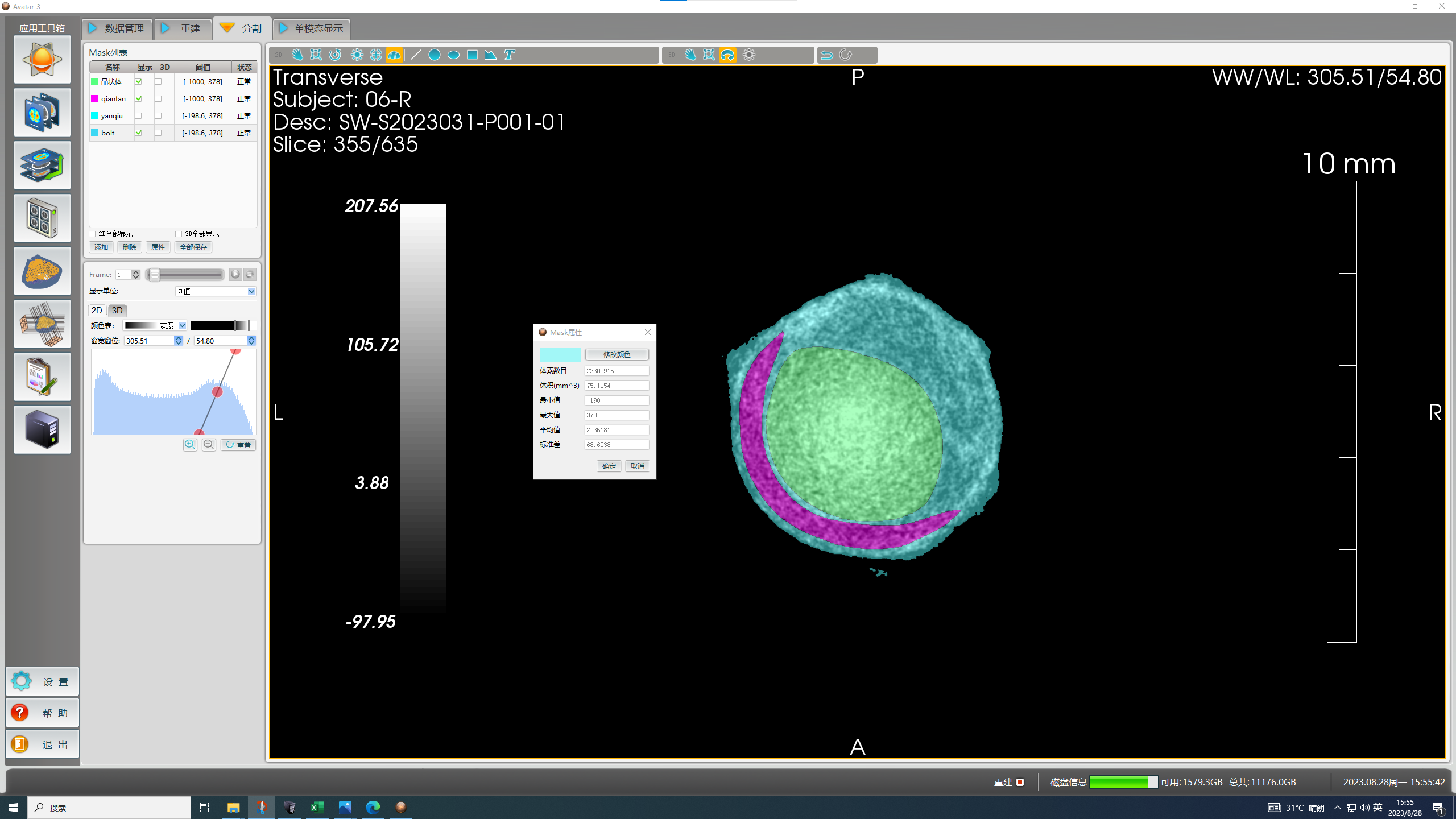

Supplement: S4 Data — (ZIP) [file pone.0310830.s004.zip › CT_SDrats/Vitreous body/06-R.png]
