# Supplementary material for: Anatomical and Micro-CT measurement analysis of ocular volume and intraocular volume in adult Bama Miniature pigs, New Zealand rabbits, and Sprague-Dawley rats
Source: PLoS One. 2024 Sep 20;19(9):e0310830. doi: 10.1371/journal.pone.0310830 (PMC11414937; doi:10.1371/journal.pone.0310830)
Supplement: Attachment — Submitted filename: Response to Reviewers.docx [file pone.0310830.s005.docx]

Dear Editor and reviewers, thank you very much for giving us the opportunity to revise this manuscript. We have carefully revised the manuscript according to your requirements, and the relevant response is as follows. Once again, we sincerely thank you!

**To academic editor and journal:**

1. **Please ensure that your manuscript meets PLOS ONE's style requirements, including those for file naming.**

**Reply:**

Dear editor, thank you again for providing us with this opportunity to revise the manuscript. We have changed the revised manuscript into the correct format as required by the journal, please check it!

**2. Thank you for stating the following financial disclosure:**

**“2023 Research Fund of Aier Eye Research Institute(No.AEI202310LC01); Science Research Foundation of Aier Eye Hospital Group (No.AR2201D3).” Please state what role the funders took in the study. If the funders had no role, please state: "The funders had no role in study design, data collection and analysis, decision to publish, or preparation of the manuscript." If this statement is not correct you must amend it as needed. Please include this amended Role of Funder statement in your cover letter; we will change the online submission form on your behalf.**

**Reply:**

Dear editor, thank you very much for your comment, we are very sorry for not stating the role of funders in the cover letter, we have added relevant information in the cover letter, thank you very much for your comments again!

**3.Thank you for stating the following in the Acknowledgments Section of your manuscript:**

**“Thanks to Aier Eye Group for its funding support; Thanks to WestChina-Frontier PharmaTech Co., Ltd. for providing laboratory facilities and animals for this study.”We note that you have provided funding information that is currently declared in your Funding Statement. However, funding information should not appear in the Acknowledgments section or other areas of your manuscript. We will only publish funding information present in the Funding Statement section of the online submission form.**

**Reply:**

Dear editor, thank you very much for your comment. We have deleted the funding information from the manuscript and added it in the cover letter, please check it!

4. **In the online submission form you indicate that your data is not available for proprietary reasons and have provided a contact point for accessing this data. Please note that your current contact point is a co-author on this manuscript. According to our Data Policy, the contact point must not be an author on the manuscript and must be an institutional contact, ideally not an individual. Please revise your data statement to a non-author institutional point of contact, such as a data access or ethics committee, and send this to us via return email. Please also include contact information for the third party organization, and please include the full citation of where the data can be found.**

**Reply:**

Dear editor, thank you very much for your comment. We wanted to submit the original data when submitting the manuscript, but the original data was too large to be submitted. That's why we wrote in the data statement that we can obtain the original data from the corresponding author. In this revised draft, we have split the original data and uploaded it, please check, thank you!

5. **Please review your reference list to ensure that it is complete and correct. If you have cited papers that have been retracted, please include the rationale for doing so in the manuscript text, or remove these references and replace them with relevant current references. Any changes to the reference list should be mentioned in the rebuttal letter that accompanies your revised manuscript. If you need to cite a retracted article, indicate the article’s retracted status in the References list and also include a citation and full reference for the retraction notice.**

**Reply:**

Dear editor, thank you very much for your comment. The references cited in this manuscript are complete and correct, and there are no references to the retracted manuscript.

**To additional editor:**

**Based on the Reviewers` comment the current manuscript needs some minor revision.**

**Reply:**

Dear editor, thank you again for providing us with this opportunity to revise the manuscript. We have made corresponding revisions to the manuscript as required by the reviewers, please check it! Thanks again for this opportunity！

**To reviewers:**

**To Reviewer #1:**

**The study aimed to measure the eyeball and its components in Bama Miniature Pigs, New Zealand rabbits, and Sprague-Dawley rats using micro-CT and anatomical techniques. They included six animals from each species and measured the volumes of the eyeball, anterior chamber, lens, and vitreous cavity. The conclusion was that combining micro-CT and anatomical measurements is effective for determining eyeball volumes in these animals. This information could be useful for future ophthalmology-related drug research.**

**Reply:**

Dear reviewer, thank you very much for your detailed review of this manuscript. Your comments are an important recognition and support for our work. Thank you again for your comments.

**To Reviewer #2:**

**1.Title:**

**- Replace “Pig” with “pig”.**

**Abstract:**

**- You can report your results using 2 decimal points.**

**Introduction:**

**- Line 78: “Salguero R et al.”…replace with “Salguero et al.”**

**- “length of each line in normal dog”….explain what do you mean by each line.**

**Reply:**

Dear reviewer, thank you very much for your comments. We have made corresponding modifications in the manuscript. Besides, we are very sorry for the confusion caused by our writing, the "length of each line in normal dog" refers to the distance between certain structures of the dog's eyeball, including the average axial length of the sphere, the average anterior posterior distance of the anterior chamber, the average anterior posterior distance of the vitreous chamber, etc. For better understanding, we have replaced this sentence with “and the length of the normal dog eye structures (Including the average axial length of the sphere, the average anterior posterior distance of the anterior chamber, the average anterior posterior distance of the vitreous chamber, etc) with CT”, please check it.

**2.Method:**

**- Lone 89: “This is an in vitro experiment”…..Do you believe that this is an in vitro experiment?! ….. please replace by “ an in vivo study”.**

**- Line 103: “The tool was then used to draw the ROI (region of interest)” could be replaced by “The tool was then used to draw the region of interest (ROI)”**

**Reply:**

Dear reviewer, thank you very much for your comments. We have made corresponding modifications in the manuscript. Besides, due to our inability to perform micro CT scans on live pigs and rabbits, the scanning chamber for small animal CT is very small and can only accommodate mice and rats. In order to standardize the measurement method, we removed the eyeball and conducted separate CT and anatomical measurements. We euthanize animals in order to extract their eyeballs for measurement of in vitro parameters. Strictly speaking, we should not consider it as an in vivo experiment. To avoid misunderstandings, we have removed the mention of in vivo and in vitro experiments. We have provided corresponding descriptions of the measurement methods in our methodology.please check it.

**3.Line 110: “All operations were performed by a single experimenter” … Do you mean “All CT scans and measurements were made by the same examiner (add Name abbreviations)?? ….Did you made measurements at the same time of examination or you made it on saved scans….please clarify. Did you make your measurements only one time or multiple times and get your mean for each eye measurements.**

**Reply:**

Dear reviewer, thank you very much for your comment. In order to reduce errors, our CT scans and eye measurements under CT were performed by the same professional imaging technician (Zongtao Shu). Due to the time-consuming process of manual delineation and reconstruction of eye structure involved in the measurement process, our technicians promptly saved all eye image data after scanning, and then uniformly measured eye volume. In addition, we also referred to other literature and did not have similar imaging data for multiple measurements. Therefore, the CT measurement data for this part was only reconstructed by outlining the eyeball structure once to obtain the required parameter results. The entire process took nearly 2 months and was quite cumbersome and complex.

4. **Line 111: Anatomical results/anatomic measurements all over the text could be replaced by “Gross volumetric estimation of ocular volume”.**

**Reply:**

Dear reviewer, thank you very much for your comments. We have made corresponding modifications in the manuscript, please check it.

**5.- Line 112-113:**

**- “intravenous injection of pentobarbital sodium (30mg/kg)” at which vein did you inject in each animal?**

**- please add concentration, trade name, manufacturer and country of origin of the anesthetic drugs.**

**Reply:**

Dear reviewer, thank you for your detailed comments on this manuscript. Both Bama miniature pigs and New Zealand rabbits were injected via ear vein after skin preparation, while SD rats were injected intraperitoneally with 30mg/kg pentobarbital sodium. We are very sorry that we did not provide detailed information on the concentration, product name, manufacturer, and country of origin of the anesthetic drug in the manuscript. Our reagents are 2mg/kg diazepam (Sigma-Aldrich, USA) and 30mg/kg pentobarbital sodium (Sigma-Aldrich, USA), which we have supplemented in the manuscript. Please check. Thank you!

**6.“all animals were euthanized through abdominal artery bleeding” ….Which abdominal artery? How did you approach it? What is your justification and reference for this method of euthanasia? How did you ensure euthanasia?**

**Rep**ly:

Dear reviewer, thank you very much for your comments. All animals in this manuscript were euthanized with diazepam and anesthetized with 30mg/kg pentobarbital sodium before cutting the abdominal aorta and bleeding to death. We have supplemented this information in the manuscript. We ensure that all animals have a safe, effective, painless, and pain free death experience, following the AVMA Animal Euthanasia Guidelines. In addition, we confirmed whether the signs of life had completely disappeared by observing the disappearance of the animal's breathing and heartbeat, as well as pinching its toe reflex. After confirming that the animal had completely died, we proceeded to remove the eyeball, which we also supplemented in the manuscript. Finally, thank you again for your comments!

**7.Statistical analysis:**

**- Did you consider running correlation and regression analysis to your measurements?**

**Reply:**

Dear reviewer, thank you very much for your valuable comment. We have added relevant statistics to the manuscript, please check it!

**8.Results:**

**- Results could be presented using 2 decimal points.**

**Reply:**

Dear reviewer, thank you very much for your comments. We have made corresponding modifications in the manuscript, please check it.

**9.Discussion:**

**- Line 162-163: “In our study, due to the large size of the pigs, in vivo, eye scanning could not be performed. In order to control the uniform conditions, ex vivo CT scanning was performed on the eyeballs of pigs, rabbits, and rats”…..this is really very confusing. For the first time here in the discussion section to include that you did not make your CT scans on life animals??!!! You have mentioned that animals were euthanized for the anatomic study not for the CT scans.**

**Then how did you position the eyes balls for scanning? How did you manage post-mortem changes of ocular dimensions during scanning??**

**Please clarify in details.**

**Reply:**

Dear reviewer, thank you very much for your valuable comments. We are very sorry that our manuscript has caused you inconvenience. In fact, we have written about the specific process in the measurement of eye volume in methodology: we euthanize all animals by bleeding the abdominal aorta after anesthesia, and it takes less than 2 minutes to completely kill the animals after cutting the abdominal aorta. Our procedure is to quickly transfer the extracted eyeballs into the CT room for scanning, with each eyeball scan lasting approximately 3-5 minutes. The scanned eyeball is first placed into the anatomical measurement volume for measurement and recording of the eyeball volume, which takes about 1-2 minutes. Then, the aqueous humor is immediately collected, which takes about tens of seconds. The eyeball that has been completely drained of aqueous humor is quickly frozen in liquid nitrogen, and the lens and vitreous body are removed and placed in the anatomical measurement container. After the tissue is thawed, readings are taken. We try to extract the anterior chamber as dry as possible during the collection of aqueous humor, and evaluate all obtained parameters based on anatomical measurements and CT scans.

We are unable to perform micro-CT scans on live pigs and rabbits because the scanning chamber for small animal-CT is very small and can only accommodate mice and rats. In order to standardize the measurement method, we removed the eyeball and conducted separate CT and anatomical measurements. We euthanize animals in order to extract their eyeballs for measurement of in vitro parameters. Strictly speaking, we should not consider it as an in vivo experiment. To avoid misunderstandings, we have removed the mention of in vivo and in vitro experiments. We have provided corresponding descriptions of the measurement methods in our methodology. In addition, we mentioned that the entire scanning process was very brief and there was almost no change in eye size, which is evidenced by the lack of statistical differences in the total eye volume of the three animals in CT and anatomical measurements.

Sorry, we did not provide a detailed explanation of the specific method for locating the eyeball for scanning. We have added it to the methodology ***(The specific way we locate the eyeball for scanning is to ensure that the entire eyeball is located within the scanning interval, manually select the lens to start drawing from the tip of the ciliary body, move towards the other end until the lens structure disappears, and then use this tool to draw the region of interest (ROI) of the lens until the anatomical structure disappears; Using the same method, select any starting surface of the eyeball or anterior chamber structure and draw its ROI until the anatomical structure disappears,the vitreous body and posterior chamber cannot be located due to the lack of a clear boundary line)*,** please check it. Finally, thank you again for your valuable comments. Thank you!

**10.Line 173: you are suggesting that the micro-CT measurements are more reliable than anatomic measurements “we posit that CT measurement results may be more reliable”……So what was your study objective and hypothesis??!! What is your gold standard to judge/prove that micro CT is reliable in determination of volumetric eye measurement?**

**Reply:**

Dear reviewer, thank you very much for your detailed review of this manuscript. We are pleased with your professional feedback. We are very sorry that we have not actually recognized micro CT as the gold standard for measuring eye volume. We have also mentioned in our discussion that MRI is actually a more accurate measurement method, but small animal MRI is a very luxurious existence in our laboratory, and currently we do not have the conditions to implement it. The main reason why we believe CT results are more reliable is as follows: we found that there were differences between the anterior chamber volume of the eyeballs of three animals and the CT results. For example, the anatomical measurement of the anterior chamber volume in pigs was 0.28±0.04ml, while the CT result was 0.19±0.05ml. We can only combine the results of the two methods to conclude that the anterior chamber volume in pigs is approximately 0.19±0.05-0.28±0.04ml. We are not sure if time will have a certain impact on the amount of room water, but there are indeed differences between these two measurement methods on room water. In fact, we believe that CT measurement results are more reliable because only the anterior chamber was delineated when measuring the anterior chamber volume, and the anatomical measurement may have extracted a small amount of posterior chamber volume. We will explain and discuss this in the manuscript.

In addition, compared to SD rats, the eyeballs of Bama miniature pigs and New Zealand rabbits are much larger, especially the eyeball volume of pigs, while the eyeball of New Zealand rabbits is slightly smaller than that of pigs. We found that the error sources of the eye contents volume of pigs and rabbits obtained by two measurement methods mainly lie in the anterior chamber volume and lens volume. Micro CT mainly uses scanning and manual delineation to obtain these two types of intraocular contents volume, reconstructing and calculating the volume of the anterior chamber and lens completely and comprehensively. Anatomical measurement, on the other hand, measures the volume of the anterior chamber by suctioning the aqueous humor in one go and measuring the volume of the anterior chamber through the scale of the syringe. Therefore, we believe that CT measurement results may be more reliable because only the anterior chamber is outlined in the measurement of anterior chamber volume, and the reason why anatomical measurement is too large may be due to the extraction of a small amount of posterior chamber volume. As for the fact that the anterior chamber volume measured anatomically in SD rats is smaller than that measured by CT, compared to pigs and rabbits, SD rats have very small eyeballs and require higher accuracy in anatomical measurements. Their aqueous humor content is also very low, which may cause losses during the transfer of aqueous humor to the measuring instrument. This may be the main reason why the anatomical measured aqueous humor volume is smaller than the CT result. The reason why the vitreous volume is larger is likely because this vitreous volume is mainly the sum of eyeball volume minus the volume of the anterior and crystalline lens. A smaller anterior chamber volume results in a larger volume in this part. The loss error caused by the larger eyeball structure and contents of rabbits and pigs can be ignored, so there is no difference in vitreous volume between the two methods.

In addition, the lens volume of pigs and rabbits measured by dissection is slightly larger than that of micro CT. We do not believe that the main error comes from CT, because there is no difference between the eye volume results obtained by CT and anatomical measurements without separating the intraocular contents. This indicates that we have no significant problems in delineating the eye structure, 3D reconstruction, and automatic measurement of volume. We speculate that the main source of error lies in anatomical measurements. As the crystalline lens is quickly frozen in liquid nitrogen and then completely removed from the eyeball, it is placed in an anatomical measurement container for volume reading after thawing. This process involves freezing, separation, thawing, and data reading, and each step may result in errors, especially the separation process which may obtain a small amount of surrounding tissue, resulting in slightly larger measurement results. However, each crystalline lens we obtain is in a frozen state and is read only after it has completely melted. The crystalline lens of SD rats is very small, and even if there is a small amount of surrounding tissue attached, it can be ignored, so there is no difference in their crystalline lens volume between the two measurement methods. We still believe that anatomical measurements are necessary because micro CT cannot clearly identify the boundary between the vitreous body and the posterior chamber. We need to rely on anatomical measurements to evaluate vitreous volume. In fact, we are not comparing the two different measurement methods, but combining them to obtain the volume of the eyeball and its contents of Bama miniature pigs, New Zealand rabbits, and SD rats. We believe that the results obtained need to be referenced by combining the two, which are complementary experimental techniques rather than a comparative relationship.

Measurement errors, especially in anatomical measurements, are relatively traditional. Despite our efforts to make each measurement result more accurate, errors cannot be avoided, especially in the measurement of anterior chamber and lens volume. We believe that future advances in measurement technology may solve this problem.

Our research goal is to accurately measure the eyeball and contents volume of Bama miniature pigs, New Zealand rabbits, and SD rats. We do not compare CT and anatomical measurements as good or bad. We believe that the two are complementary in this study and hope that our results have practical reference value. Given that there are currently almost no articles reporting on this topic, we hope that our research will provide useful value for future research on ophthalmic drugs.

We have described and explained the above content in our discussion. Finally, thank you very much for your valuable feedback. We will improve our research methods in future studies to make the results more accurate. Thank you!

**11.Figures:**

**- Figure 1: please add reference to panels a,b, c within “Figure caption”. You may need to include description of volumetric measurements in the CT scan.**

**Reply:**

Dear reviewer, we are very sorry for the trouble caused to your review due to our lack of writing the panels a,b, c within Figure 1, we have modified the corresponding content in the manuscript according to your comments. Besides, we have added a description in the methodology that includes volume measurement in CT scans ***(After the ROI of each eye structure such as anterior chamber and lens was drawn, Avatar software provides mask of the lens and anterior chamber, and automatically calculates its volume as the physiological volume of the corresponding structure (see Fig 1), without secondary or manual calculation.),*** please check. it. Thank you again for your comments!
